# Mapping human resources for eye health in 21 countries of sub-Saharan Africa: current progress towards VISION 2020

**DOI:** 10.1186/1478-4491-12-44

**Published:** 2014-08-15

**Authors:** Jennifer J Palmer, Farai Chinanayi, Alice Gilbert, Devan Pillay, Samantha Fox, Jyoti Jaggernath, Kovin Naidoo, Ronnie Graham, Daksha Patel, Karl Blanchet

**Affiliations:** 1 International Centre for Eye Health Clinical Research Department, Faculty of Infectious & Tropical Diseases, London School of Hygiene & Tropical Medicine, Keppel St., London WC1B 7HT, UK; 2 African Vision Research Institute, 172 Umbilo Road Umbilo, Durban 4001, South Africa; 3 International Agency for the Prevention of Blindness (Africa Region), 172 Umbilo Road Umbilo, Durban 4001, South Africa

**Keywords:** Human resources, Eye health, sub-Saharan Africa, Cataract, Low vision, Blindness, Ophthalmology, Optometry, Nursing, Vision 2020

## Abstract

**Background:**

Development of human resources for eye health (HReH) is a major focus of the Global Action Plan 2014 to 2019 to reduce the prevalence of avoidable visual impairment by 25% by the year 2019. The eye health workforce is thought to be much smaller in sub-Saharan Africa than in other regions of the world but data to support this for policy-making is scarce. We collected HReH and cataract surgeries data from 21 countries in sub-Sahara to estimate progress towards key suggested population-based VISION 2020 HReH indicators and cataract surgery rates (CSR) in 2011.

**Methods:**

Routinely collected data on practitioner and surgery numbers in 2011 was requested from national eye care coordinators via electronic questionnaires. Telephone and e-mail discussions were used to determine data collection strategies that fit the national context and to verify reported data quality. Information was collected on six practitioner cadres: ophthalmologists, cataract surgeons, ophthalmic clinical officers, ophthalmic nurses, optometrists and ‘mid-level refractionists’ and combined with publicly available population data to calculate practitioner to population ratios and CSRs. Associations with development characteristics were conducted using Wilcoxon rank sum tests and Spearman rank correlations.

**Results:**

HReH data was not easily available. A minority of countries had achieved the suggested VISION 2020 targets in 2011; five countries for ophthalmologists/cataract surgeons, four for ophthalmic nurses/clinical officers and two for CSR. All countries were below target for optometrists, even when other cadres who perform refractions as a primary duty were considered. The regional (sample) ratio for surgeons (ophthalmologists and cataract surgeons) was 2.9 per million population, 5.5 for ophthalmic clinical officers and nurses, 3.7 for optometrists and other refractionists, and 515 for CSR. A positive correlation between GDP and CSR as well as many practitioner ratios was observed (CSR *P* = 0.0042, ophthalmologists *P* = 0.0034, cataract surgeons, ophthalmic nurses and optometrists 0.1 > *P* > 0.05).

**Conclusions:**

With only a minority of countries in our sample having reached suggested ophthalmic cadre targets and none having reached targets for refractionists in 2011, substantially more targeted investment in HReH may be needed for VISION 2020 aims to be achieved in sub-Saharan Africa.

## Abstract (fr)

**Résumé:**

**Contexte**

Les ressources humaines en santé oculaire semblent être moindres en Afrique subsaharienne que dans les autres régions du monde, mais peu de données sont disponibles. Nous avons collecté des données sur les ressources humaines et le taux de chirurgie de la cataracte dans 21 pays de l’Afrique subsaharienne afin d’estimer pour 2011 les progrès réalisés concernant les indicateurs suggérés de ressources humaines et de chirurgie de la cataracte de VISION 2020.

**Méthodes**

Les données sur les six catégories de cadre de santé et les opérations de chirurgie régulièrement collectées en 2011 ont été demandées aux coordinateurs des programmes nationaux par questionnaire envoyé électroniquement. Des discussions par courrier électronique et téléphone ont permis de vérifier la qualité des données. Les associations avec les caractéristiques de développement ont été menées en utilisant le test de la somme des rangs de Wilcoxon et les coefficients de corrélation de Spearman.

**Résultats**

Une minorité de pays ont atteint les cibles suggérées de VISION 2020: cinq pays pour les ophtalmologistes et les opérateurs de cataracte, quatre pays pour les infirmiers en ophtalmologie et les techniciens supérieurs en ophtalmologie et deux pays pour les opérations de cataracte. Tous les pays n’ont pas assez d’optométristes. Le ratio régional (échantillon) pour les chirurgiens (ophtalmologistes et operateurs de cataracte) était de 2,9 par million de population, 5,5 pour les infirmiers en ophtalmologie et les techniciens supérieurs en ophtalmologie, 3,7 pour les optométristes et autres réfractionnistes, et 515 pour le taux de chirurgie de cataracte. Il existe une corrélation positive entre PNB et le taux de chirurgie de cataracte et le PNB et le nombre de la plupart des professionnels (opération de cataracte *P* = 0.0042, ophtalmologistes *P* = 0.0034, opérateurs de cataracte, infirmiers en ophtalmologie et optométristes 0.1 > *P* > 0.05).

Etant donné que seulement une minorité de pays d’Afrique subsaharienne dans notre échantillon ont atteint en 2011 les cibles suggérées VISION 2020 en termes de nombre de professionnels de la santé oculaire et qu’aucun pays n’a un nombre suffisant de réfractionnistes, il est nécessaire et urgent d’investir dans les ressources humaines de la santé afin de pouvoir atteindre les objectifs VISION 2020.

## Background

### The importance of evidence on human resources for eye health (HReH)

Development of human resources for eye health (HReH) has been consistently recognized as central to eye health service delivery in global initiatives, reports and resolutions on visual impairment over the last two decades [1-5]. Global action plans for the prevention of avoidable blindness and visual impairment recommend that national programmes train and maintain an eye health workforce whose size and composition is proportionate to the eye care needs in a population [2-4]. As key indicators of eye health system development, regular measurement of HReH to population ratios is suggested to help programmes monitor progress towards overall programme goals, the most recent version being: reduction in the prevalence of avoidable visual impairment by 25% between 2010 and 2019 [3]. The global initiative known as ‘VISION 2020: the Right to Sight’, which is an established partnership between the World Health Organization (WHO) and the International Agency for the Prevention of Blindness (IAPB), has furthermore suggested regional HReH targets which are thought to be needed for substantial reductions in avoidable visual impairment by the year 2020 [2,4].

Since the World Health Report was published in 2006 [6], there is greater awareness amongst the public health community of the need to strengthen the collection and analysis of human resources data and to develop evidence-based strategies and policies for workforce training, retention and distribution. Global studies of selected eye health cadres have noted a deficit of information in sub-Saharan Africa, in particular [1,7]. From the little information that is available, the eye health workforce in Africa is thought to be much smaller and less dense than other regions of the world and unevenly distributed within countries, making VISION 2020 programme goals more difficult to achieve in poorer, rural areas [1,2,8,9]. There is little analysis of the reasons for this deficit of practitioners in the eye care market (for example, is it due to low numbers of professionals trained?; a lack of capacity in the labour market to absorb new graduates?; low levels of retention and high migration?; or some combination of these and other factors?) [10]. Strategies to potentially address these gaps in services delivery such as through task-shifting in a team approach also lack evidence [2,11]. Information in all of these areas would help develop appropriate and specific HReH strategies which countries are expected to design under the new Global Action Plan [3].

### HReH cadres in service delivery

Globally, 191 million people were estimated to be moderately or severely visually impaired in 2010 [12]. Around 80% of visual impairment is thought to be preventable or curable through the delivery of cost-effective eye care services [13]. Global HReH indicators have therefore been selected [2-4] to reflect the skills needed to address the two main preventable causes of visual impairment, estimated to represent three quarters of vision loss globally: cataract (33%) and uncorrected refractive errors (42%) [3].

Treatment of cataract (cataract extraction with implantation of an intraocular lens) is safe and efficacious to restore sight, as well as cost-effective [14]. Annual cataract surgical rates (CSR) measure the delivery of cataract surgical services in a health system as a ratio of operations per million population per year. Although the current WHO global action plan suggests no specific global or regional targets for cataract surgery, suggested target annual CSR for sub-Saharan Africa have been selected [4] and revised [2] before. At 1,000 in 1997 and 2,000 a decade later, CSR targets were originally selected as rough estimates of service delivery need based on several assumptions including a uniform prevalence of cataract blindness in sub-Saharan Africa and an assumption that mostly blind eyes would be operated (assumptions laid out in [15,16]). The appropriateness of these assumptions has since been challenged [17,18]. Nonetheless, for planning purposes a suggested CSR target of 2,000 is often used.

Suggested VISION 2020 ophthalmic HReH targets have furthermore been justified in terms of this CSR target, although the evidence base for this is also weak. For a population of one million people in sub-Saharan Africa, surgical programmes were thought to be able to achieve 2,000 cataract surgeries per year using four teams, each operating on 2 to 3 cataract cases/hour, one day per week through static and outreach services [19]. With surgical teams normally led by one surgeon, this approximated to a suggested VISION 2020 target of four ophthalmologists per million population [2]. Non-physician clinical officers and nurses can also be trained to share this key task to compensate for the lack of ophthalmologists in Africa (especially in rural areas), although this strategy is debated [9,20-23]. With subsequent research suggesting that cataract surgery productivity varies greatly by surgeon and by setting [21,24,25], this also calls the universality of a four surgeons per million population target into question. If programmes achieved higher average productivity per team, for example, HReH targets could be reduced.

Each surgeon is ideally supported by three to five mid-level personnel including ophthalmic nurses and ophthalmic clinical officers (OCOs) who also provide inpatient care and run outpatient clinics. These and other allied eye health cadres provide the bulk of eye care (including preventive, diagnostic and referral services) in most rural and remote areas [9,11]. It is notable, however, that, for both ophthalmic service HReH categories, lower VISION 2020 targets have been chosen for the sub-Saharan Africa region given the limited pool from which trainees for eye care can be drawn, despite this region having the greatest need for HReH [2]. Suggested VISION 2020 targets therefore estimate that at least one worker from this HReH category is needed for a typical district population of 100,000 [19], which is equivalent to 10 per million in Africa [2,16].

Refractive errors can be corrected in a convenient, cost-effective manner by a wide range of personnel such as optometrists, ophthalmic assistants, opticians and other cadres [2,26,27]. Cadres involved in refraction are also often the first point of contact for people with eye diseases and thus can refer to and receive referrals from ophthalmic services in a team approach [3]. Although no specific target was set in the original global initiative [4], a global suggested target of 20 refractionists per million population has since been chosen by VISION 2020, with no distinction for the African region where evidence on incidence of refractive error and the productivity of refractionists is limited [2,26]. This target was selected following a service delivery model for eye care developed in India, whereby ‘Vision Centres’ provide primary eye care and first-level refractive error services to a population of 50,000 people [28]. Depending on the resource and legal context, they can be staffed to address uncorrected refractive error by someone with either one or four years’ optometric training, through flexible training schemes considered appropriate to an African context [27-29].

Suggested facility-based targets have also been chosen to reflect the need for other personnel who support eye care service delivery such as eye team managers and equipment technicians as well as personnel who are essential for the integration of eye care at primary health care level such as community-based and integrated eye care workers [2]. Questions remain about how this HReH structure proposed by VISION 2020 can respond to chronic disease conditions such as glaucoma and diabetic retinopathy and the need for preventive activities linked to primary care.

### Available information on HReH in sub-Saharan Africa

The most recent estimate of the burden of visual impairment in 48 countries of sub-Saharan Africa for 2010 indicates that 21.4 million people are visually impaired, including 4.8 million who are blind [12]. The only publicly-available assessment of HReH comparing multiple cadres in sub-Saharan Africa was conducted during a global survey by the Human Resources Working Group of the International Agency for the Prevention of Blindness (IAPB) in 2006 [1]. Using data from 45 countries in the region, 2,210 ophthalmologists were identified yielding a regional ratio of 3.1 ophthalmologists per million population (using pooled data; country mean 3.8, country median 1.8; estimates calculated by us to reflect current VISION 2020 definitions using data available in [1] and using approach outlined in the Methods section), suggesting that sub-Saharan Africa as a whole has met three quarters of the suggested VISION 2020 target. An additional 674 ophthalmologists were needed to meet the suggested VISION 2020 target in 2006, corresponding to an increase of one third of the existing number. A more recent global survey of ophthalmologists (using data from 2010) similarly estimated there were 2.7 ophthalmologists per million population in sub-Saharan Africa, a figure nearly 20 times lower than in Latin America and 30 times lower than former socialist and established market economies [7].

Data on ophthalmic nurses/medical assistants (presumed to include cataract surgeons) and on optometrists (not including other types of refractionists) was collected from fewer countries and estimated at 11.4 (using pooled data from 40 countries; country mean 17.8, median 6.6) and 3.7 (using pooled data from 21 countries; country mean 2.4, country median 0.4) per million population, respectively [1]. Large differences in practitioner to population ratios, particularly for optometrists, were noted across countries sampled. No subnational analyses were undertaken, although these have since been completed in individual countries [30-32].

Relative to HReH target performance, sub-Saharan Africa is thought to be underperforming in terms of suggested VISION 2020 target cataract surgical rates [16,33]. In 2006, the median CSR reported from 45 countries was 387 (mean 662, calculated using data available in [2], pooled data to calculate regional ratio not available), well below the suggested 2,000 target for year 2020, especially in Francophone and Lusophone countries. This underperformance needs to be better understood and related to HReH ratios and their distribution.

We undertook a study of the eye health workforce in sub-Saharan Africa to inform programme strategies and policies for HReH in the region. Specifically, we sought to (i) provide up-to-date and accurate data on the active eye health workforce between and within countries, (ii) assess current progress towards key HReH and cataract surgical targets as set out in the VISION 2020 global strategy [2,3], and (iii) determine associations between VISION 2020 progress and national development characteristics. In a related study, we build on this work to predict future VISION 2020 target performance and identify associations between predicted performance and HReH structure [34].

## Methods

### National questionnaires

National-level eye health services data was collected by researchers at the International Centre for Eye Health (ICEH) and African Vision Research Institute (AVRI) via questionnaires, which were electronically circulated to key informants in all 33 countries of sub-Saharan Africa with more than 4 million population as of the year 2010, as well as in 3 countries with less than 4 million population where research collaborations already existed (Botswana, Gambia, Guinea-Bissau). Effort was focused in these countries only, given the difficulties faced by earlier studies attempting to access HReH data in this region [1,7].

Developed in collaboration with IAPB and the Human Resources Taskforce Programme of the World Health Organization, the questionnaire was pretested in four countries. English and French versions of the final questionnaire (see Additional files 1 and 2) were sent electronically to key informants in the eye health system (usually national eye care coordinators) who were also notified and followed up by telephone between December 2011 and May 2013. This period included an extensive phase of qualitative discussions with respondents to suggest data collection strategies that fit the national context to verify that all definitions were understood and to investigate internal data discrepancies and non-response fields.

In each country, national eye care coordinators were requested to coordinate data collection and reporting. Where national coordinators could not be contacted or were unable to assist in data collection, other key informants were recruited through ICEH alumni, AVRI and Sightsavers networks of contacts. Within countries, coordinators were encouraged to use several sources of data including state/district eye care coordinators, professional networks (ophthalmologists and optometrists’ associations), HReH training institutions and eye care nongovernmental organizations (NGOs) or researchers who had conducted recent surveys. In two countries, with especially large populations and decentralized information systems (Nigeria and Ethiopia), in-country surveys were conducted with paid data collectors to work with national coordinators. Informants were requested to provide information on the entire HReH workforce and surgical services, including those in non-public facilities.

The questionnaire was designed to collect information on six HReH cadres identified in VISION 2020 plans and appropriate to the sub-Saharan African context:

(i) ophthalmologists (physicians (MD or equivalent degree) who specialize in the eye and visual system),

(ii and iii) OCOs/medical assistants and ophthalmic nurses (non-physician practitioners with an advanced (minimum one year) qualification in ophthalmology, including ‘*techniciens supérieurs en ophtalmologie*’ in Francophone countries),

(iv) cataract surgeons (non-physician ophthalmic practitioners or non-specialist physicians additionally trained in cataract surgery),

(v) optometrists (personnel with a BSc or diploma in optometry (normally three to four years)) and

(vi) mid-level refractionists (all other mid-level personnel with refraction training including: refractionists, ophthalmic assistants/technicians, low vision specialists, opticians and equivalent).

In VISION 2020 documents, cadres (ii) OCOs, (iii) ophthalmic nurses, and (iv) cataract surgeons are combined as ‘mid-level personnel’ and share a single suggested target [2]. In our analyses, we collected and reported separate data on all three cadres and in some cases combined cataract surgeons with ophthalmologists in a new ‘surgeons’ category to highlight human resource needs associated with cataract surgical performance. In two countries (Democratic Republic of Congo and Madagascar), ‘cataract surgeons’ were defined locally to mean non-ophthalmologist general physicians who have been trained to conduct cataract surgery and may have received some training in ophthalmology. Similarly, optometrists and mid-level refractionists were combined in some instances in the analyses as a new ‘refractionists’ category. Data was not collected on: community-based eye care educators, cataract case-finders, integrated eye care workers and equivalents, or on non-ophthalmic surgical and inpatient ward staff, including general nurses or clinical officers with ‘on-the-job’ training in ophthalmology or refractive error services but without formal qualifications.

For all cadres in the active workforce, information on their location (capital city or outside capital) and type of employer (government, NGO/mission or private-for-profit) was collected.

When information on the number of cataract surgeries performed in 2011 could not be provided, informants were asked to provide data for another year. When national data on cataract surgeries performed could not be provided by informants in-country (Malawi and Zambia), the most recent estimate available in the IAPB Africa database was used (Daniel Etya’ale, personal communication). National coordinators were asked to use personal local knowledge to estimate the proportion of cataract surgeries performed by ophthalmologists, as, in most countries, this data is not routinely collected.

### Data management and analysis

For consistency, all questionnaire data was reviewed by a single researcher (JP) as it was received, to identify inconsistencies and likely reporting errors in the data requiring follow-up. Data was then entered directly into a Stata 12.1 (StataCorp, College Station, TX, USA 2009) database and outputs cross-checked with questionnaires by a second researcher. Stata 12.1 was used for all statistical analyses described below.

#### National comparisons to suggested V2020 targets

HReH practitioner to population ratios were calculated for all six cadres and three HReH practitioner categories corresponding to VISION 2020 classifications (‘Surgeons’ consisting of: ophthalmologists and cataract surgeons, ‘OCOs/nurses’: ophthalmic clinical officers and ophthalmic nurses, and ‘Refractionists’: optometrists and mid-level refractionists) using 2011 questionnaire data and national population data estimates for 2011 (calculated using annual population growth and 2010 populations from [35]; estimates for Sudan and South Sudan provided separately from [36] and the South Sudan National Bureau of Statistics, personal communication, respectively). Specific practitioner to population ratios were also calculated for capital cities using data available from [37]. Cataract surgeries per population (which are cataract surgery rates, CSR) and per surgeon (considering ophthalmologists and cataract surgeons) were similarly calculated.

In countries where the national capital city was smaller than another city in the country (Benin, Nigeria, Tanzania), the larger city was taken to mean ‘capital’ in our analyses. Data was counted as ‘missing’ in any sub-category (for example, practitioner or surgery location or sector) if informants estimated that > 5% of the confirmed total active workforce could not be categorized.

As reliable surgeries data in Nigeria could only be provided from 13/37 states, we calculated a national estimate based on the proportional CSR in these states for use in all analyses. There was substantial variation between CSR data provided by informants from this study and the existing IAPB database which provided estimates for Malawi and Zambia: a mean difference of 122 surgeries per million population (range 2 to 610), or 22% of our figures. These data were not necessarily directly comparable, however, as we could not assess whether data from all sectors was included in the IAPB estimates. According to our mapping criteria using one third of the suggested VISION 2020 target as a boundary to assess performance (see below), using IAPB CSR data instead would have reclassified only one country above or below the 500 surgeries per million population boundary.

Analyses of the eye health human resources workforce of individual countries are available in Additional file 3 and will be made available on the IAPB web site. This paper reports findings from our multi-country analysis of HReH in sub-Saharan Africa represented in our sample.

#### Multi-country comparisons

Following previous work [7], national surgeon and OCOs/nurse practitioner-population ratios were categorized using one quarter of the VISION 2020 target for the Africa region, the VISION 2020 Africa target, and the Asia region target as the first, second and third category boundaries, respectively, and mapped using ArcGIS software**,** Redlands, California, USA. Since only global targets exist for refractionists, the first and third categories selected were one quarter of and four times the global target, respectively.

HReH and surgical target performance across the sample was assessed in three ways: by pooling the total number of practitioners or surgeries from all countries and dividing this numerator by the total population of all countries reporting data (regional ratio), by calculating the average practitioner to population ratio or CSR across countries (country mean) and by calculating the median practitioner to population ratio or CSR across countries (country median). All three are normally reported for comparison.

To explore significant statistical differences in population and surgical ratios between national development characteristic categories of the sample, median ratios were selected as the measure of central tendency and Wilcoxon rank sum tests were used. The data was categorized as follows: UN African subregion (with South Sudan considered as part of eastern Africa), language of education and whether a training school for the cadre exists in the country (using questionnaire data). Associations or relationships between these ratios and the following continuous variables were explored using both Spearman rank correlations and locally weighted scatter plot smoothing (LOWESS) curves: gross domestic product (GDP, based on purchasing-power-parity (PPP) per capita in current international dollars, 2012, from [38]), government expenditure on health (per capita PPP, 2011, from [39]), human development index values (HDI, 2012 from [40] and [41] for South Sudan), population size (2010, as above) and geographic size (km^2^ from [42], and [43,44] for Sudan and South Sudan). Non-parametric tests were employed in the analysis because of the limited number of observations and because the data did not fit normal distributions.

### Ethics statement

This study was approved by the London School of Hygiene & Tropical Medicine’s Ethics Review Committee.

## Results

Sufficient HReH data to include in analyses was received from 21 countries, giving a response rate of 58%, but higher in Anglophone countries (13/14 or 87%, compared to 7/16 or 44% in Francophone, 1/3 or 33% in Horn of Africa and 0/3 or 0% in Lusophone countries approached). Insufficient data was received from three countries (Burkina Faso, Guinea-Bissau and South Africa); in all other cases, contact could not be made with national coordinators (seven countries: four Francophone, one Lusophone, two Horn of Africa) or forms were not returned after a minimum of three approaches to follow-up (six countries: five Francophone, one Lusophone). The total population of countries represented in the study in 2011 was 633 million, accounting for 72% of the population of sub-Saharan Africa.

Amongst the 21 countries sampled, the survey identified a total of 1,444 ophthalmologists, 363 cataract surgeons (including 98 physician cataract surgeons from Democratic Republic of Congo and Madagascar), 456 OCOs and 2,997 ophthalmic nurses in 2011. Complete information on optometrists and mid-level refractionists was only available from 18 countries each (17 when data is combined, representing 46% of the sub-Saharan population); 696 and 925 were identified from this sample, respectively.

### HReH distribution by country

#### Surgeons

Three countries (Nigeria, Sudan and Ethiopia) employed almost two thirds of the ophthalmologists (64.5%) in the sample. Kenya, Democratic Republic of Congo and Ethiopia were the biggest employers of cataract surgeons (54.5%) while Nigeria, Sudan and Botswana (representing nearly a third of the study population) had none.

**Figure 1:**
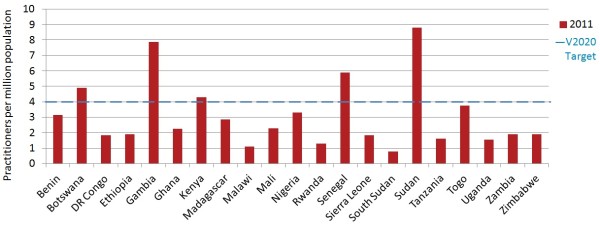
Surgeons per million population in 2011.

**Figure 2:**
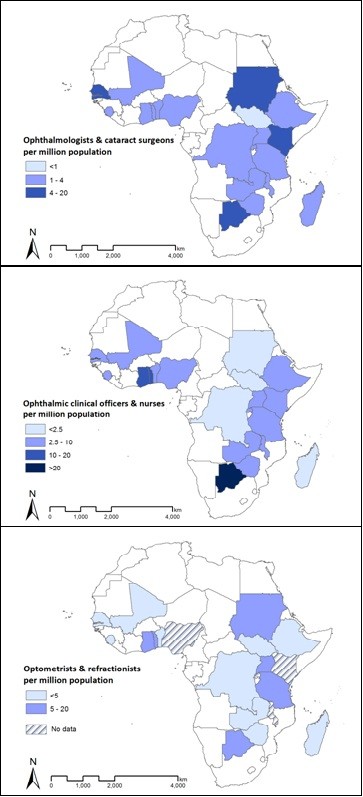
Distribution of VISION 2020 human resources for eye health (HReH) cadres per million population in 21 countries of sub-Saharan Africa in 2011.

When data is pooled across the sample, the regional ratio of ophthalmologists was 2.3 per million population. When all surgeons are considered (ophthalmologists and cataract surgeons combined), the regional practitioner to population ratio was 2.9 (country mean 3.1, median 2.2, range 0.8 to 8.8), suggesting that sub-Saharan Africa as a whole may currently be three quarters of the way to the suggested 4.0 target (see Table 1 for summary results and Additional file 4 for practitioner to population ratios for each country). Five countries met or exceeded the suggested V2020 target for surgeons (Figures 1, 2). When only ophthalmologists are considered, only three countries (Botswana, Senegal and Sudan) met or exceeded this target; an additional two countries (Gambia and Kenya) met it when cataract surgeons were also considered. Ten countries, representing nearly half of the population in the sample (47.5%), were less than half way to meeting the target. South Sudan currently has the greatest need for surgeons.

**Table 1:** Practitioners per million population in 2011, by cadre, in sub-Saharan African countries included in the study

**Eye care cadre**	**n countries included**	**Regional ratio**	**Country mean**	**Country median**	**Minimum**	**Maximum**
Ophthalmologists	21	2.3	2.2	1.3	0.3	8.8
Cataract surgeons	21	0.6	0.9	0.4	0.0	7.3
All surgeons	**21**	**2.9**	**3.1**	**2.2**	**0.8**	**8.8**
OCOs	21	0.7	1.0	0	0.0	6.2
Ophthalmic nurses	21	4.7	7.4	3.2	0.0	45.9
All OCOs/Nurses	**21**	**5.5**	**8.4**	**6.2**	**0.0**	**45.9**
Optometrists	18	1.7	1.7	0.4	0.0	8.8
Mid-level refractionists	18	2.1	2.2	1.1	0.0	8.9
All refractionists	**17**	**3.7**	**4.0**	**1.8**	**0.3**	**14.8**

Table legend: figures in bold indicate ratios for combined categories of practitioners.

#### OCOs/nurses

**Figure 3:**
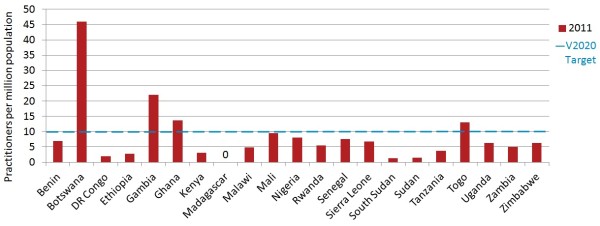
Ophthalmic clinical officers (OCOs)/nurses per million population in 2011.

Nigeria, representing 25.7% of the study population, contributed 44.1% of the sample’s ophthalmic nurses. Only seven countries employed OCOs.When data is pooled across the sample, the regional ratio of OCOs/nurses per million population was 5.5 (country mean 8.4, median 6.2, range 0.0 to 45.9), suggesting that sub-Saharan Africa as a whole may currently be only half way to the suggested 10.0 target, but there was a substantial range in suggested target performance between countries. For example, Madagascar had no practitioners in either of these cadres; Malawi and Rwanda had no ophthalmic nurses; Botswana employed no OCOs but had more than four times the suggested target number of ophthalmic nurses. Four countries (none of which employed OCOs) exceeded the suggested V2020 target, but they represented only 5.5% of the population in the sample (Figure 3).

#### Refractionists

**Figure 4:**
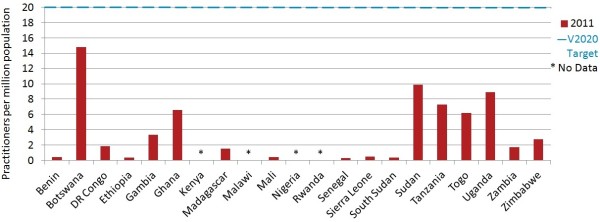
Refractionists per million population in 2011.

Although data submitted on optometrists was incomplete for Nigeria, based on analysis of partial data provided, this country would probably be the largest employer of optometrists, followed by Sudan. Uganda employed one third of mid-level refractionists in the sample (32.9%). Tanzania was the only country that employed high numbers of both cadres. Mid-level refractionists represented 57.1% of this practitioner workforce.When data is pooled across the 17 countries in the sample where data on both cadres of refractionists was available, progress towards this suggested V2020 target appeared to be poorer than for the other HReH categories. The combined regional ratio of all refractionists per million population was 3.7 (country mean 4.0, median 1.8, range 0.3 to 14.8), suggesting that sub-Saharan Africa as a whole was less than one quarter of the way to the suggested 20.0 global target in 2011. No countries had reached this practitioner to population ratio, although Botswana was close (mean 14.8, Figure 4).

### HReH distribution by sector

**Figure 5:**
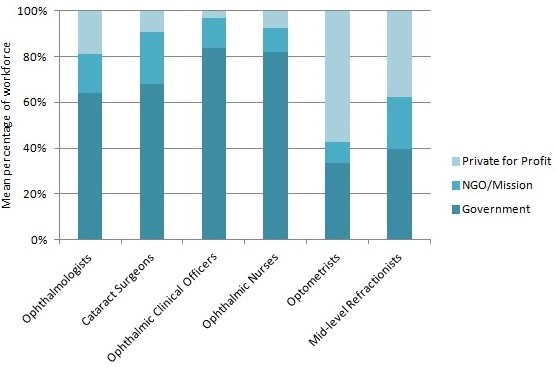
**Distribution of human resources for eye health (HReH) by sector.** Data comes from Table 2.

Within countries, 81.9% of surgeons (both types, combined country mean) worked in the government or NGO/mission sectors (Table 2, Figure 5). Whereas 18.7% of ophthalmologists mainly worked in the private-for-profit sector, only 9.4% of cataract surgeons did (mainly in Democratic Republic of Congo and Madagascar where cataract surgeons are physicians). OCOs and nurses also tended to work in public facilities, with only 7.3% in private employment. Optometrists were most commonly (57.2%) in the private sector; mid-level refractionists were commonly employed in both the private (37.6%) and government sectors (39.6%). Proportionately, cataract surgeons and mid-level refractionists were the cadres most commonly employed in the NGO/mission sector (22.5% and 22.8%, respectively).

**Table 2:** Proportional distribution of eye health practitioners by sector

	**Country mean% workforce by sector**			
**Eye care cadre**	**n countries**	**Government**	**NGO/Mission**	**Private-for-profit**
Ophthalmologists	20	63.9	17.4	18.7
Cataract surgeons	16	68.1	22.5	9.4
All surgeons	**19**	**63.4**	**18.5**	**18.1**
OCOs	7	83.8	13.0	3.3
Ophthalmic nurses	17	82.2	10.2	7.6
All OCOs/Nurses	**18**	**81.7**	**11.1**	**7.3**
Optometrists	14	33.5	9.2	57.2
Mid-level refractionists	13	39.6	22.8	37.6
All refractionists	**15**	**41.4**	**16.7**	**44.9**

Table legend: data on the sector in which practitioners worked was not available for all sectors in all countries. In analyses of individual cadres, countries with ≤ one practitioner in the country were considered not to employ this cadre and excluded from analyses. In analyses of combined cadres which sum practitioners of two types, however, countries with none of an individual cadre were counted as having ‘zero’, so the number (‘n’) of countries considered to have contributed data may be different. Figures in bold indicate ratios for combined categories of practitioners.

### HReH distribution within countries

While ophthalmologists and optometrists were more likely to be employed in national capital cities (67.2% and 66.3%, respectively), all other cadres were more frequently employed outside the capital in smaller cities or rural areas (Table 3). All rural populations were larger than urban populations within countries. When location-specific practitioner per population ratios were calculated, ratios were uniformly worse in areas outside of capital cities. Differences between ratios inside and outside capital cities were particularly large for ophthalmologists (mean 10.8 inside versus 0.7 outside capitals), optometrists (10.6 versus 0.9) and mid-level refractionists (8.4 versus 1.8) (Table 4).

**Table 3:** Proportional distribution of eye health practitioners within countries

	**Mean% practitioners by location**		
**Eye care cadre**	**n countries**	**Inside capital**	**Outside capital**
Ophthalmologists	20	67.2	32.6
Cataract surgeons	18	38.3	61.7
All surgeons	**20**	**54.1**	**45.5**
OCOs	7	29.4	70.6
Ophthalmic nurses	17	33.7	66.0
All OCOs/Nurses	**19**	**30.0**	**69.6**
Optometrists	15	66.3	33.7
Mid-level refractionists	16	41.4	58.6
All refractionists	**16**	**49.8**	**50.2**

Table legend: ‘0’ practitioners in countries where individual cadres do not exist have been excluded in analyses of mean proportions for individual cadres and considered to be zero when cadres are combined. Botswana: location data for ophthalmic nurses comes from the government sector only. Ghana: data on ophthalmologists and optometrists does not include practitioners in the private sector. Malawi: ‘capital’ was interpreted as the three largest cities in the country. Uganda: data for mid-level refractionists does not include practitioners in the private sector. Figures in bold indicate ratios for combined categories of practitioners.

**Table 4:** Practitioners per million population in 2011, by cadre, inside and outside capital cities

	**n countries**	**Country mean**	**Inside capital**		**Outside capital**	
			**mean**	**(range)**	**mean**	**(range)**
Ophthalmologists	20	1.8	10.8	(2.0 to 29.7)	0.7	(0.0 to 3.2)
Cataract Surgeons	18	1.1	2.3	(0.0 to 12.1)	1.0	(0.0 to 9.5)
All surgeons	**20**	**2.8**	**12.9**	**(4.0 to 29.7)**	**1.7**	**(0.2 to 9.5)**
OCOs	7	3.0	8.5	(0.0 to 19.9)	2.4	(0.3 to 6.5)
Ophthalmic Nurses	17	9.0	19.3	(1.2 to 69.3)	7.6	(0.0 to 39.5)
All OCOs/Nurses	**19**	**9.2**	**20.5**	**(1.2 to 69.3)**	**7.7**	**(0.7 to 39.5)**
Optometrists	15	2.0	10.6	(0.0 to 51.8)	0.9	(0.0 to 3.8)
Mid-level refractionists	16	2.6	8.4	(0.0 to 39.6)	1.8	(0.0 to 8.6)
All refractionists	**16**	**4.2**	**16.7**	**(0.0 to 64.4)**	**2.5**	**(0.0 to 9.3)**

Table legend: ‘0’ practitioners in countries where individual cadres do not exist have been excluded in analyses of mean proportions for individual cadres and considered to be zero when cadres are combined. Botswana: location data for ophthalmic nurses comes from the government sector only. Ghana: data on ophthalmologists and optometrists does not include practitioners in the private sector. Malawi: ‘capital’ was interpreted as the three largest cities in the country. Uganda: data for mid-level refractionists does not include practitioners in the private sector. Figures in bold indicate ratios for combined categories of practitioners.

### CSR suggested V2020 target performance

**Figure 6:**
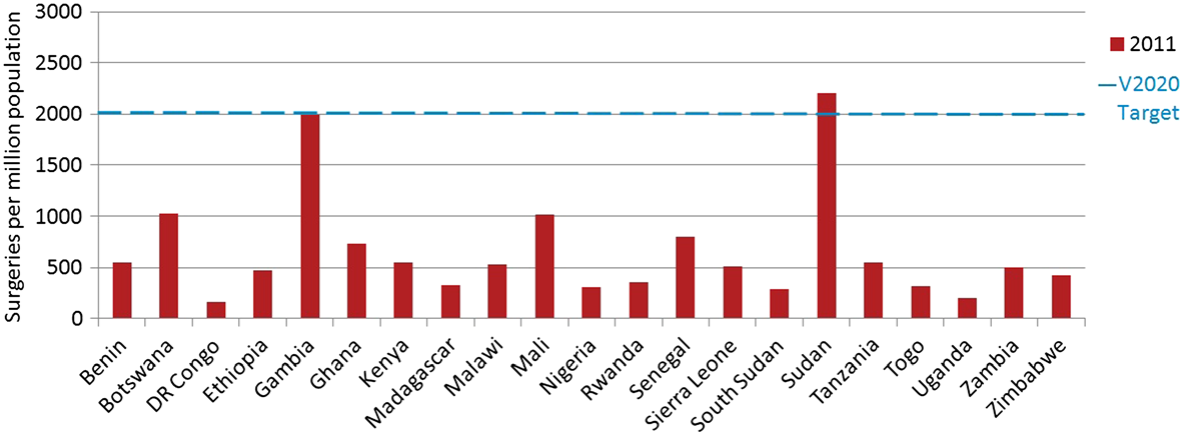
**Cataract surgeries per million population in 2011.** Estimates of surgeries for ‘2011’ come from 2008 for Malawi, from 2010 for Benin, Botswana, Democratic Republic (DR) of Congo, Ethiopia, Gambia, Ghana, Kenya, Madagascar, Rwanda, Senegal, Sierra Leone, Sudan, Uganda and Zimbabwe and from 2012 for South Sudan.

In 2011, 2/21 countries (Gambia at 1,993 and Sudan at 2,210) had met (or nearly met) the suggested VISION 2020 CSR target of 2,000 surgeries per million population. The regional CSR based on pooled populations was 515 overall (country mean 659, country median 509, range 163 to 2,210), with the region as a whole only a quarter of the way to the V2020 target. Democratic Republic of Congo had the lowest CSR in 2011, at 163 (Figure 6, Additional file 4).

The regional ratio of surgeries performed per surgeon in 2011 was 180 (country mean 235, country median 252, n = 21), with a minimum of 85 in Togo and a maximum of 483 in Malawi. No countries reached either of the alternate VISION 2020 model targets of 500 or 1,000 surgeries per annum per surgical team.

### V2020 progress associated with development

Strong evidence for an association between geographical regions and certain practitioner to population ratios was found, with higher ophthalmologist (regional median 2.8 versus 1.2, *P* value = 0.0063) and ophthalmic nurse (8.8 versus 2.4, *P* = 0.0012) ratios in western Africa compared to eastern (Table 5). There was borderline evidence for an association between language of education and practitioner ratios for optometrists only, with more optometrists in Anglophone compared to Francophone countries (median 3.8 versus 0.5, *P* = 0.0588). No evidence was found for an association between practitioner ratios and whether or not countries had at least one national training institute for any cadre (all *P*-values > 0.15).

**Table 5:** Mean practitioner to population ratios according to development characteristics

	**Ophthalmologists**				**Cataract surgeons**				**OCOs**				**Ophthalmic nurses**				**Optometrists**				**ML refractionists**			
	**n**	**Mdn**	**min to max**	***P*-value**	**n**	**Mdn**	**min to max**	***P*-value**	**n**	**Mdn**	**min to max**	***P*-value**	**n**	**Mdn**	**min to max**	***P*-value**	**n**	**Mdn**	**min to max**	***P-*** **value**	**n**	**Mdn**	**min to max**	***P*-value**
African region (UN)																								
Northern	1	8.8		**0.0063**^**a**^	0			0.3545	0			-	1	1.5		**0.0012**^**a**^	1	8.8		0.8312	1	1.1		0.6605
Western	7	2.8	0.8 to 4.3		4	1.3	0.3 to 7.3		0				8	8.8	6.8 to 22.0		4	0.6	0.3 to 4.5		5	2.1	0.3 to 5.4	
Middle	1	1.0			1	0.8			0				1	1.9			0				1	1.8		
Eastern	10	1.2	0.3 to 2.1		9	0.6	0.3 to 2.2		7	2.6	0.6 to 6.2		7	2.4	0.1 to 6.2		6	1.0	0.1 to 3.8		8	1.0	0.1 to 8.8	
Southern	1	4.9			0				0				1	45.9			1	5.9			1	8.9		
Language of education																								
English	12	1.5	0.3 to 8.8	0.4469	9	0.6	0.3 to 7.3	0.7576	6	1.8	0.6 to 6.2	0.3173	12	4.7	0.1 to 45.9	0.4606	7	3.8	0.1 to 8.8	**0.0588**	11	2.1	0.2 to 8.9	0.8961
French	7	2.2	0.8 to 4.3		4	1.2	0.3 to 2.0		1	5.4			5	7.6	1.9 to 13.0		4	0.5	0.3 to 0.8		4	1.6	0.3 to 5.4	
Horn of Africa	1	1.3			1	0.6			0				1	2.8			1	0.3			1	0.1		
National training school for cadre exists																								
0	4	1.5	0.3 to 4.9	0.5708	5	1.0	0.3	0.5582	1	0.6		0.1573	2	4.5	2.0 to 6.9	0.7656	6	1.1	0.1 to 5.9	0.6310	4	1.0	0.1 to 5.4	0.6015
≥ 1	16	1.5	0.5 to 8.8		8	0.7	0.3 to 7.3		4	5.1	0.8 to 6.2		15	6.2	0.1 to 45.9		6	2.2	0.3 to 8.8		11	1.5	0.2 to 8.8	

Table legend: countries with ≤ one practitioner in any cadre have been excluded from analyses. Regional comparisons tested were western versus eastern and language comparisons were English versus French, because of small numbers in other categories. Median ratios were used rather than means because data was not normally distributed. *P*-values in bold indicate significance at the 10% level, ^a^at 5%, using the Wilcoxon rank sum test. CO: clinical officer; max, maximum; Mdn: median; min, minimum; ML: mid-level; OCOs, ophthalmic clinical officers.

**Figure 7:**
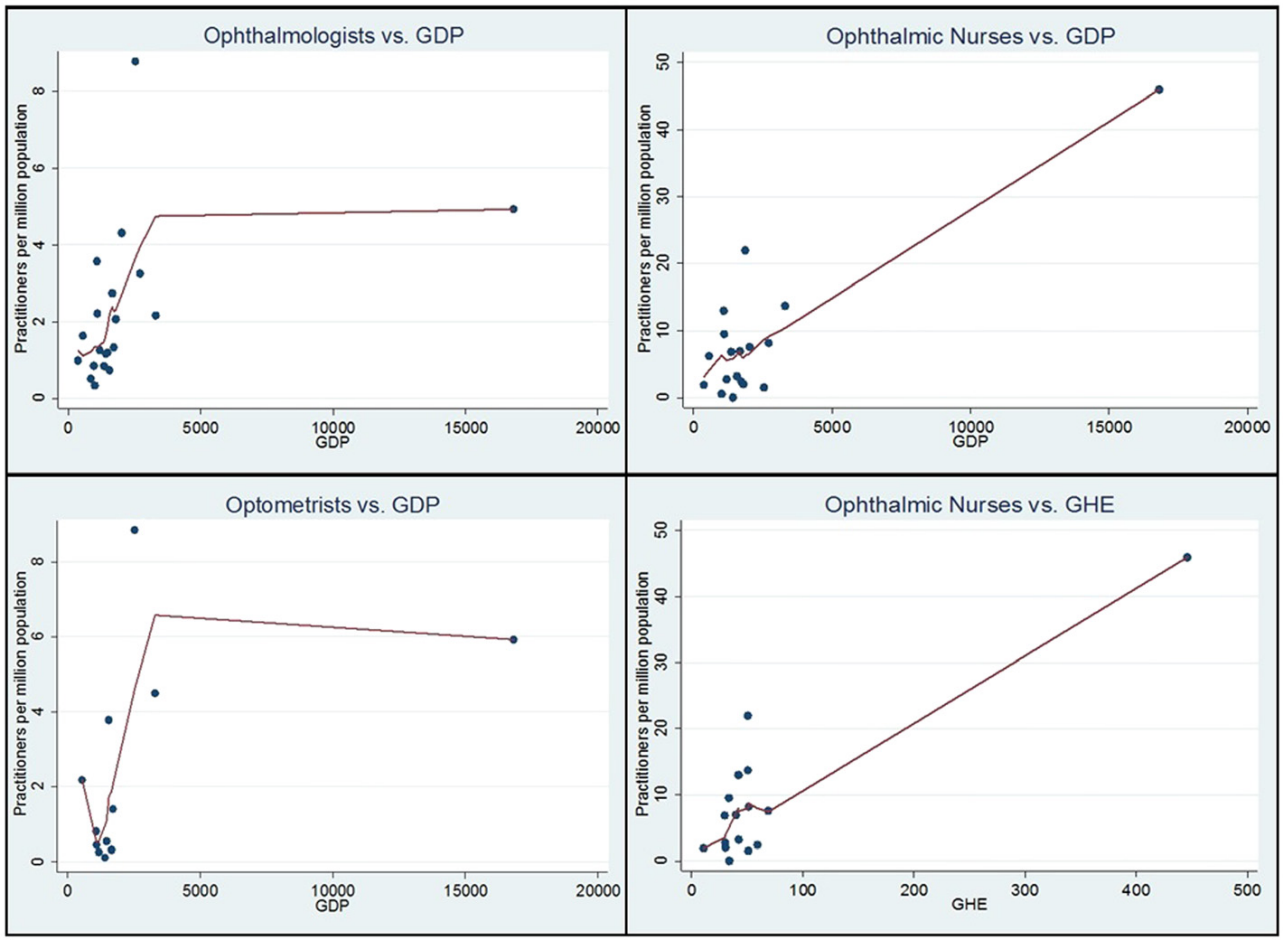
**Association between practitioner ratios and gross domestic product (GDP) and government health expenditure (GHE).** Using locally weighted scatter plot smoothing to visualize associations, increasing monotonic relationships are apparent between practitioner to population ratios presented and GDP/GHE (strongly/almost ever-increasing for ophthalmologists versus GDP at *P* < 0.05 and weakly increasing for the other observations at 0.1 > *P* > 0.05). Botswana is an outlier in all graphs, with both a high GDP and GHE.

Across many cadres, there appeared to be a positive correlation between a country’s practitioner to population ratio and its national GDP (ophthalmologists *P* = 0.0034, cataract surgeons, ophthalmic nurses and optometrists 0.1 > *P* > 0.05) and government expenditure on health (optometrists *P* = 0.0098, ophthalmologists 0.1 > *P* > 0.05) (Table 6, Figure 7). For mid-level ophthalmic cadres, a smaller population and geographic size also appeared associated with higher practitioner to population ratios.

**Table 6:** Correlations between density of eye care practitioners and development characteristics

	**Ophthalmologists**			**Cataract surgeons**			**Ophthalmic COs**			**Ophthalmic nurses**			**Optometrists**			**ML refractionists**		
	**n**	**R**	***P*-value**	**n**	**R**	***P*-value**	**n**	**R**	***P*-value**	**n**	**R**	***P*-value**	**n**	**R**	***P*-value**	**n**	**R**	***P*-value**
GDP	20	0.6226	**0.0034**^**a**^	14	0.4637	**0.0949**	7	−0.2857	0.5345	18	0.4118	**0.0895**	12	0.5594	**0.0586**	16	0.2794	0.2946
GHE	18	0.4159	**0.0861**	12	−0.0699	0.8290	6	0.2571	0.6228	16	0.4147	0.1102	11	0.7364	**0.0098**^**a**^	14	0.1033	0.7253
HDI	20	0.3263	0.1603	14	0.4286	0.1263	7	−0.1429	0.7599	18	0.3746	0.1256	12	0.4126	0.1826	16	0.5853	0.0172*
Population size	20	−0.1203	0.6134	14	−0.0198	0.9465	7	−0.2143	0.6445	18	−0.4799	**0.0439**^**a**^	12	−0.1678	0.6021	16	−0.0706	0.7950
Geographic size	20	0.0421	0.8601	14	−0.0901	0.7593	7	−0.7857	**0.0362**^**a**^	18	−0.4159	**0.0861**	12	0.3007	0.3423	16	−0.2676	0.3163

Table legend: countries with ≤ one practitioner in any cadre have been excluded from analyses. R = Spearman’s rho. *P*-values in bold indicate significance at the 10% level, ^a^at 5%, tested using Spearman rank correlations. CO: clinical officer; GDP: gross domestic product; GHE: government health expenditure; HDI: human development index; ML: mid-level.

National GDP was the only development characteristic (positively) associated with cataract surgical rates (*P* = 0.0042, data not shown).

## Discussion

This study evaluated the performance of eye health human resources development in 2011 using key population-based indicators of the global VISION 2020 programme in 21 countries across sub-Saharan Africa. Our estimates suggest that a minority of countries had achieved the suggested VISION 2020 targets for ophthalmologists, ophthalmic nurses and clinical officers (the latter two being cadres encompassed by the ‘allied ophthalmic personnel’ indicator in the 2014 to 2019 action plan [3]) and for cataract surgery rates in 2011. All countries were below the suggested target for optometrists, even when other cadres who perform refractions as a primary duty were also considered.

Practitioner to population ratio performance looks worse, in addition to being inequitable, when analyzed according to practitioner location, with ratios for all cadres higher in capital cities. This concentration of resources in cities means that suggested VISION 2020 target ratios for the ophthalmic cadres have been exceeded for most urban African populations, at the expense of rural service users. Similar domestic geographic imbalances have been observed for other types of health cadres in sub-Sahara [44,45]. This geographic misdistribution is particularly important for ophthalmologists, optometrists and other refractionists who are also more likely to work mainly in the private sector, suggesting that market dynamics play a role in this imbalance. Geographic and sectoral imbalances in the health workforce can be attributed to several factors: poor working conditions in the public sector, particularly in rural areas (for example, because of low salaries, lack of dedicated positions, poor maintenance of facilities, inadequate provision of appropriate equipment and supervision by managers) [46-48], health systems factors (for example, the structure and dynamics of the labour market and the role of the private sector as an employer) [49,50] and a lack of innovation in national human resources policies regarding retention and task-shifting (for example, through development of attractive career maps, purposive recruitment of students from rural areas or shifting certain responsibilities to trained lower cadres) [51-53].

Given that we identified a fairly consistent correlation between a country’s wealth (GDP) and the ratio of practitioners to population in its workforce, it appears that economic growth generates demand for health spending in eye care, resulting in higher training or higher retention of professionals in the workforce, or both. Accordingly, we identified a similar correlation with government health spending (GHE) and some practitioner cadres which would be expected since GDP is a major predictor of public health expenditure (mainly on salaries) [54]. Different policies have been suggested for countries experiencing different demand- or needs-based human resource shortages [54], and form a complex dynamic that deserves further study.

Shared regional and/or linguistic historical human resource policy decisions also are likely to strongly underlie trends in workforce mix across the continent and may confound financial associations (the low response rate from Francophone Africa should also be noted). Optometrists are worryingly scarce in Francophone countries (maximum ratio 0.8 compared to the suggested 20.0 target), with ophthalmic cadres likely being diverted into refraction work. Ophthalmologist ratios are particularly low in eastern (median ratio 1.2) and Anglophone (1.5) countries of Africa. In regional analyses, cataract surgeons largely do not appear to be making up for this shortage, but do contribute notably to the 2011 achievement of the suggested surgeon target in Anglophone Kenya and Gambia. Interestingly, while sub-Sahara meets three quarters of the HReH VISION 2020 requirements for surgeons, CSR performance is only one quarter of the way towards the suggested target. We further explore the potential impact of cataract surgery task-shifting on VISION 2020 performance in this sample in our companion paper (Palmer *et al*. submitted). Since CSR targets were selected based on the surgical human resources required to meet a population’s cataract needs, this suggests that further research is needed to examine this discrepancy, either by examining surgeon productivity or re-visiting the assumptions used in target selection.

Like others before us [1], we found mid-level refractionists and optometrists to be the cadres most difficult to collect data on. This was partly due to the complexity of identifying a common definition of ‘refractionist’ for cross-country comparisons, with major variations in educational background, job title and responsibilities noted within and between countries [3]. Harmonization of training and qualification standards in this cadre has recently been identified as a key HReH strategy for IAPB Africa as its scope and function is currently poorly understood by policy-makers [9]. It may also be due to the fact that most refractionists work in the private sector which governments have less incentive to monitor. In some countries, professional optometry societies were able to support data collection on practitioners in the active workforce. A lack of private sector data on all cadres, particularly ophthalmologists and refractionists, necessitated exclusion of South Africa, one of the more populous countries in the region, from this study. This approach used by us and previous studies which relied on obtaining data from a small number of ‘key informants’ in the public sector [1,26] may therefore be insufficient to map and understand the dynamics of this particular refractionist workforce; more in-depth field studies may instead be needed in some places.

Comparing our findings to previous HReH surveys in Africa using similar methodology (Table 7), our regional estimates indicate substantially lower V2020 target performance than expected. While it is possible that this indicates decreasing performance over time, our analysis of practitioner entry and exit trends 2008 to 2010 elsewhere (Palmer *et al*. submitted) suggests that the practitioner workforce is growing faster than the general population in most countries, which would not support this hypothesis. It is more likely that differences between surveys reflect differences in survey design. Our survey collected more HReH data from a smaller number of countries than the other surveys (21 versus 40), with a focus on the most populous countries in sub-Saharan Africa. Because these surveys are concerned with relatively rare, specialist health human resources over a relatively small number of countries, small differences in the way that surveys are conducted and data is included can have seemingly large influences on the regional picture of performance. For this reason, and as this report has done, it is most informative to examine performance across the sample, for example, using graphs and figures which plot as much country-specific data as possible, rather than single regional estimates. This multi-country analysis is also written to be read alongside analyses of performance in individual countries (Additional file 3). Further within-country research verifying data reported here and examining performance over time can also be conducted to contextualize our quantitative findings for policy-making.

**Table 7:** Regional human resources for eye health (HReH) practitioner to population ratios in sub-Saharan Africa, estimated from different surveys

	**V2020 target per million population**	**V2020 survey 2006**	**Resnikoff *et al*. survey **[12]** 2010**	**This survey 2011**
Ophthalmologists	4	3.1	2.7	2.3
Cataract surgeons	10	11.4	-	6.0
OCOs			-	
Ophthalmic nurses			-	
Optometrists	20	3.7	-	3.7
Mid-level refractionists		-	-	
CSR	2,000	662	-	515
Cataract surgeries per surgeon	500	-	-	180

Table legend: estimates presented in each survey and for each cadre come from different numbers and samples of countries [1,7]. Practitioner ratios for individual cadres estimated in this study are available in Table 1.

Finally, our study highlights the difficulty of collecting human resources data in the eye care sector. In most countries contributing data to this study, data on the number and distribution of eye care professionals was not centralized and easily available; in large countries such as Ethiopia and Nigeria, a specific additional survey was required. It is troubling that, despite the presence of national prevention of blindness or eye health committees in most African countries, fifteen years into the VISION 2020 initiative it is still so difficult to access this type of data. The eye care sector is not alone in this; very little data exists on the dynamics of human resources for health generally, and even when it does exist, it is rarely used for planning purposes [55,56]. As the Word Health Report emphasizes [6], we need to collect, disseminate and analyze more data on HReH. This will require greater and sustained investment from national authorities and their partners to identify appropriate tools that can be integrated into national information systems.

## Conclusions

The VISION 2020 campaign emphasizes the key role that eye care human resources development plays in reducing vision loss globally. However, comprehensive data on human resources for eye health is not easily available in sub-Saharan Africa and should be improved to assist VISION 2020 planning. Substantially more and more targeted investment in training and deployment of HReH may be needed for the aims of the VISION 2020 campaign to be achieved in sub-Saharan Africa. Only a minority of countries in sub-Saharan Africa have so far achieved the suggested VISION 2020 targets suggested for ophthalmic cadres and for cataract surgeries. The relative number of refractionists to populations who need services to correct refractive error is currently very low and reflects a lack of investment from health systems in sub-Saharan countries to address this key eye health morbidity. A major imbalance in the distribution of the eye health workforce between urban and rural areas makes a critical analysis of domestic eye health system dynamics even more pressing if V2020 goals are to be achieved in the poorest populations in rural areas. The development of a new intermediate cadre, non-physician cataract surgeons, to compensate for the concentration of ophthalmologists in urban areas may be one solution. Further research is needed to test innovative recruitment, retention and task-shifting solutions to improve universal eye health coverage in sub-Saharan Africa.

## Abbreviations

AVRI: African Vision Research Institute; CO: clinical officer; CSR: cataract surgery rate; DR: Democratic Republic; GDP: gross domestic product; GHE: government health expenditure; HDI: human development index; HReH: human resources in eye health; IAPB: International Agency for the Prevention of Blindness; ICEH: International Centre for Eye Health; LOWESS: locally weighted scatter plot smoothing; MD: medical doctor; ML: mid-level; NGO: nongovernmental organization; OCO: ophthalmic clinical officer; PPP: purchasing-power-parity; WHO: World Health Organization; UN: United Nations.

## Competing interests

The authors declare that they have no competing interests. This study was funded by Sightsavers International.

## Authors’ contributions

JP, SF, JJ, DPatel and KB designed the methodology and the data collection tools. JP, SF, DPillay and FC collected data. JP, FC and AG analyzed data. JP drafted the article. JJ, KK, RG, DPatel and KB reviewed the draft version. All authors read and approved the final manuscript.

## Authors’ information

JP, MSc, PhD, is a Research Fellow at the International Centre for Eye Health in the Clinical Research Department at the London School of Hygiene & Tropical Medicine.

FC, BSc, MPhil, is the Statistician for the African Vision Research Institute and the Brien Holden Vision Institute.

AG, BSc, MSc, was a Research Assistant at the International Centre for Eye Health in the Clinical Research Department at the London School of Hygiene & Tropical Medicine.

DPillay, IMM Dip (Marketing Management), was the African Vision Research Institute Research Coordinator on the study.

SF, BSc, MSc, is a Public Health Registrar and was a Research Assistant at the International Centre for Eye Health in the Clinical Research Department at the London School of Hygiene & Tropical Medicine.

JJ, MA, PhD, is the Research Manager of the African Vision Research Institute.

KSN, OD, MPh, PhD, is the CEO of the African Vision Research Institute, the Global Programmes Director of the Brien Holden Vision Institute and the Africa Chair of the International Agency for the Prevention of Blindness.

RG, MA, Dip. Ed., MA, is the Director of HRH Programmes, IAPB Africa.

DPatel, MD, MMed (Ophth), MSc (CEH), Lecturer in Public Health Ophthalmology at the International Centre for Eye Health in the Clinical Research Department at the London School of Hygiene & Tropical Medicine.

KB, MMgt, MScPH, PhD, is a Lecturer in Health Systems Research at the International Centre for Eye Health in the Clinical Research Department at the London School of Hygiene & Tropical Medicine.

## Supplementary Material

Additional file 1 HReH questionnaire in English. Click here for file

Additional file 2 HReH questionnaire in English. Click here for file

Additional file 3 Country HReH analyses. Click here for file

Additional file 4 **Practitioner and surgical population ratios in 2011 by country.** Table legend: “.” refers to no available data. Figures in bold indicate ratios for combined categories of practitioners (columns) or for the sub-Saharan Africa region (row). Click here for file
