# Supplementary material for: Mapping human resources for eye health in 21 countries of sub-Saharan Africa: current progress towards VISION 2020
Source: Hum Resour Health. 2014 Aug 15;12:44. doi: 10.1186/1478-4491-12-44 (PMC4237800; doi:10.1186/1478-4491-12-44)
Supplement: Additional file 1 — HReH questionnaire in English. [file 1478-4491-12-44-S1.doc]

**Data Collection Tool - Human Resources for Eye Care**

This tool and the data it collects are part of a collaborative study being undertaken by the International Centre for Eye Health (ICEH), the African Vision Research Institute (AVRI) and the International Agency for the Prevention of Blindness (IAPB). This study is looking at eye care human resources in countries in sub Saharan Africa with populations of at least 4 million people.

This tool collects information about the volume and distribution of eye care workers, how workers enter and exit the workforce, key characteristics of service delivery and related outcomes.

This data will be analysed to provide information to inform government and non-governmental organisation policy and funding decisions for eye care human resources.

**Guidance**

Thank you for taking the time to complete this questionnaire. Please fill in the tables presented below. We would appreciate accurate numbers, however if this is not possible, we would be really grateful if you could provide estimates, indicating where these have been used.

Data sources could include your country’s Vision2020 monitoring submissions, the Ministry of Health (national and/or district level), eye care training institutions or eye care professional organisations. Where possible we have filled the tables in for you with publically available data. If this information is incorrect, please delete the data and replace it with your accurate information.

If you have any questions please contact Jennifer Palmer at the London School of Hygiene and Tropical Medicine, [jennifer.palmer@lshtm.ac.uk](mailto:jennifer.palmer@lshtm.ac.uk).

Please could you return the questionnaire by XXX. Results of the study will be fedback to you.

Definitions of terms used can be found at appendix 1.

**Questionnaire**

**Part 1** - General Questions

| **1.1** | **Country** | |  | | | | |
| --- | --- | --- | --- | --- | --- | --- | --- |
|  |  | |  | | | | |
| **1.2** | **Name of person coordinating response** | |  | | | | |
|  |  | |  | | | | |
| **1.3** | **Job title of person coordinating response** | |  | | | | |
|  |  | |  | | | | |
| **1.4** | **Contact details of person coordinating response address/phone/email** | |  | | | | |
|  | | | | |
|  | | | | |
|  |  | |  | | | | |
| **1.5** | **How many people are in your population, and how many are over the age of 50 (please note the year this estimate is from)?** | | Total population | | Population over 50 | | Year of estimate and source |
|  | |  | |  |
|  | | | | | | | |
| **1.6** | **What is the percentage of people in the population estimated to have cataracts?** | | | | | | |
|  |  | Under 50 | | 50 and over | | All ages | |
| a | Cataract |  | |  | |  | |
| b | Blindness caused by cataract |  | |  | |  | |
| c | Visual impairment caused by cataract (<6/18) |  | |  | |  | |
| If possible use population based prevalence estimates. Please give source of estimates. If you have data from a RAAB, please attach the RAAB report. | | | | | | | |
| Source of information | |  | | | | | |

**Part 2 – Entry into the workforce – internal and external factors**

| **2.1** | **In the table below, please list the names of all training centres in your country which lead to a qualification as an:** | | | | | | | | |
| --- | --- | --- | --- | --- | --- | --- | --- | --- | --- |
|  |  | **Type of qualification** | | | | | | | |
|  |  | Diploma | | | | Other qualification - MMed; MD; Membership/Fellowship | | | |
| a | Ophthalmologist |  | | | |  | | | |
|  | | | | | | | | | |
|  |  | BSc | Diploma | | | Certificate | | | Other |
| b | Ophthalmic nurse |  |  | | |  | | |  |
|  | | | | | | | | | |
|  |  | BSc | | | | Other | | | |
| c | Optometrist |  | | | |  | | | |
|  | | | | | | | | | |
|  |  | Diploma | | | Certificate | | | Other | |
| d | Cataract surgeon or equivalent |  | | |  | | |  | |
|  |  |  | |  | | |  | | |
|  |  | Diploma | | | Certificate | | | Other | |
| e | Other mid level eye care workers |  | | |  | | |  | |
| Source of information | |  | | | | | | | |
|  | | | | | | | | | |
| **2.2** | **Please complete the following table:** | | | | | | | | |
|  |  | **How many people (nationals of your country only) graduated/qualified from academic and educational institutions in the last three years (2008 -2010) as the following?** | | | **How many eye care workers (any nationality) started working in your country as the following in the last three years (2008 -2010)?** | | | **How many eye care workers started working for the government only as the following in the last three years (2008 -2010)?** | |
| a | Ophthalmologist |  | | |  | | |  | |
| b | Ophthalmic nurse |  | | |  | | |  | |
| c | Optometrist |  | | |  | | |  | |
| d | Cataract surgeon or equivalent |  | | |  | | |  | |
| e | Other mid level eye care workers |  | | |  | | |  | |
| Source of information | |  | | | | | | | |
| *Note: If institutions in your country also train students from other countries, please only report the numbers of nationals from your country trained. If your country sends students to other countries to be trained, please report these numbers, as well. | | | | | | | | | |

**Part 3** – Active workforce

| **3.1** | **Which sector do eye care workers mainly work in? Please complete the following table, providing information about the numbers of different types of eye care workers currently working in each healthcare sector** | | | | | | | |
| --- | --- | --- | --- | --- | --- | --- | --- | --- |
|  |  | | Primarily government administered facilities | Primarily mission/NGO | | Primarily private for profit | | Total |
| a | Ophthalmologist | |  |  | |  | |  |
| b | Ophthalmic nurse | |  |  | |  | |  |
| c | Optometrist | |  |  | |  | |  |
| d | Cataract surgeon or equivalent | |  |  | |  | |  |
| e | Other mid level eye care workers | |  |  | |  | |  |
| Source of information | | |  | | | | | |
|  | | | | | | | | |
| **3.2** | **Where do the Ophthalmologists mainly work? Please complete the table filling in the numbers of Ophthalmologists in each category:** | | | | | | | |
|  |  | Capital City | | | Outside capital city | | Total | |
| a | Primary |  | | |  | |  | |
| b | Secondary |  | | |  | |  | |
| c | Tertiary |  | | |  | |  | |
| Source of information | |  | | | | | | |
|  | | | | | | | | |
| **3.3** | **Where do the Ophthalmic nurses mainly work? Please complete the table filling in the numbers of Ophthalmic nurses in each category:** | | | | | | | |
|  |  | Capital City | | | Outside capital city | | Total | |
| a | Primary |  | | |  | |  | |
| b | Secondary |  | | |  | |  | |
| c | Tertiary |  | | |  | |  | |
| Source of information | |  | | | | | | |
|  | | | | | | | | |
| **3.4** | **Where do the Optometrists mainly work? Please complete the table filling in the numbers of Optometrists in each category:** | | | | | | | |
|  |  | Capital City | | | Outside capital city | | Total | |
| a | Primary |  | | |  | |  | |
| b | Secondary |  | | |  | |  | |
| c | Tertiary |  | | |  | |  | |
| Source of information | |  | | | | | | |
|  | | | | | | | | |

| **3.5** | **Where do cataract surgeons mainly work? Please complete the table filling in the numbers of cataract surgeons in each category:** | | | | |
| --- | --- | --- | --- | --- | --- |
|  |  | Capital City | | Outside capital city | Total |
| a | Primary |  | |  |  |
| b | Secondary |  | |  |  |
| c | Tertiary |  | |  |  |
| Source of information | |  | | | |
|  |  |  | |  |  |
| **3.6** | **Where do other mid level eye care workers mainly work? Please complete the table filling in the numbers of mid level eye care workers in each category:** | | | | |
|  |  | Capital City | | Outside capital city | Total |
| a | Primary |  | |  |  |
| b | Secondary |  | |  |  |
| c | Tertiary |  | |  |  |
| Source of information | |  | | | |
|  |  |  | |  |  |
| **3.7** | **How many eye care workers active in the workforce originate from your country?** | | | | |
| a | Ophthalmologist | |  | | |
| b | Ophthalmic nurse | |  | | |
| c | Optometrist | |  | | |
| d | Cataract surgeon or equivalent | |  | | |
| e | Other mid level eye care workers | |  | | |
| Source of information |  | | | | |

**Part 4 –** Exiting from the workforce

| **4.1** | **How many people in the following groups left employment in the last three years, for whatever reason (e.g. retirement, death, emigration)?** | | | | |
| --- | --- | --- | --- | --- | --- |
|  |  | Government | NGO | Private | Total |
| a | Ophthalmologist |  |  |  |  |
| b | Ophthalmic nurse |  |  |  |  |
| c | Optometrist |  |  |  |  |
| d | Cataract surgeon or equivalent |  |  |  |  |
| e | Other mid level eye care workers |  |  |  |  |
| f | No idea? Please tick |  |  |  |  |
| Source of information | |  | | | |
|  | | | | | |

| **4.2** | **How many eye care staff emigrate to another country within 5 years of graduation/qualifying? (please tick)** | | | | | |
| --- | --- | --- | --- | --- | --- | --- |
|  |  | Ophthalmo-logists | Ophthalmic nurses | Optometrists | Cataract surgeons | MLEPs |
| a | None |  |  |  |  |  |
| b | A few (1-20%) |  |  |  |  |  |
| c | Some (21-50%) |  |  |  |  |  |
| d | The majority (51-81%) |  |  |  |  |  |
| r | All or almost all (81-100%) |  |  |  |  |  |
| f | No idea |  |  |  |  |  |
| Source of information | |  | | | | |

**Part 5 – Service delivery and outcomes**

| **5.1** | **Could you describe the patient pathway that a typical cataract patient would experience from first to last contact with services? This could be one example of the different types of service provision in your country?**  For example:  1. Patient consults outreach worker. Patient referred for cataract surgery.  2. Pre assessment with Ophthalmic nurse in secondary facility  3. Operation carried out by Ophthalmologist and 3 allied eye care workers  4. One night overnight stay  5. One follow up appointment with Ophthalmic nurse. | |
| --- | --- | --- |
|  | Source of information: | |
|  |  | |
| **5.2** | **How many cataract surgeries were undertaken in 2010 (if no data are available for 2010 please note which year this number is from, and the source of the information)** |  |
|  | | |
|  |  |  |
| **5.3** | **Approximately what proportion of cataract surgeries were carried out by Ophthalmologists in 2010 (please note the source of the information)?** |  |

**Thank you for completing this survey**

**Appendix 1**

**Definitions[[1]](#endnote-2),[[2]](#endnote-3)**

**Ophthalmologist, Optometrist, Ophthalmic Nurse**

Occupational groups who have education and training equivalent to a bachelor’s or higher degree with major study in the specialised field of eye care.

**Cataract surgeons**

Eye care workers (for example, clinical officers) who are not doctors but are trained in and practice cataract surgery.

**Ophthalmic clinical officers (non-surgical)**

If your country trains clinical officers with a specialisation in ophthalmology but not cataract surgery, you may create a separate table for this cadre in each section.

**Other mid level eye care workers**

All other personnel, not included in other categories, who have a technical and/or supportive role to the provision of eye care. Training and education for the role is gained on the job and through educational attainment below degree level. Roles that are included in this grouping are refractionists, ophthalmic technicians, low vision specialists/assistants.

**Levels of care**

- Primary care – This is the first eye care contact for the patient. This contact usually takes place in the community.
- Secondary care – Eye care at this level results from a referral from another health professional. Care at this level will be more specialised than primary care. Secondary care takes place at a district level.
- Tertiary care –Eye care at this level results from a referral from another health professional at primary or secondary level. Care at this level will be more specialised that secondary care. Tertiary care is usually provided at a regional/country level in specialist centres.

**Government employed –** this includes the military

**Notes**

Where the tool asks for results in the last year, please state the year the data is from. This should be the most recent data available to you.

1. Human Resource Development Working Group – Vision2020. *Global Human Resource Development Assessment for Comprehensive Eye Care*. [Online] Available from: [http://www.vision2020.org/documents/WHO%20Publications/Global_Human_Resource_Development_Assessment_For_Comprehensive_Eye_Care.pdf](http://www.vision2020.org/documents/WHO Publications/Global_Human_Resource_Development_Assessment_For_Comprehensive_Eye_Care.pdf) [Accessed 24th March 2011]. [↑](#endnote-ref-2)
2. Para IABP HRD Programme Committee. Taxonomy of eye healthy occupations. [↑](#endnote-ref-3)
