# Supplementary material for: Mapping human resources for eye health in 21 countries of sub-Saharan Africa: current progress towards VISION 2020
Source: Hum Resour Health. 2014 Aug 15;12:44. doi: 10.1186/1478-4491-12-44 (PMC4237800; doi:10.1186/1478-4491-12-44)
Supplement: Additional file 2 — HReH questionnaire in English. [file 1478-4491-12-44-S2.doc]

**Outil de collecte de données – Ressources Humaines en soins oculaires**

*Cet outil et les données qu'il recueille font partie d'une étude conjointe menée par le Centre international pour la santé des yeux (ICEH), et l'Agence Internationale pour la Prévention de la Cécité (IAPB). Cette étude se penche sur les ressources humaines de soins oculaires dans 23 pays en Afrique sub-saharienne.

Cet outil recueille des informations sur le volume et la distribution des agents de soins oculaires, la façon dont les travailleurs entrent et sortent de la du marché du travail, les principales caractéristiques de la prestation de services et les résultats connexes.

Ces données seront analysées afin de fournir des informations pour éclairer les politiques des gouvernements et les organisations non-gouvernementales et les décisions de financement pour les ressources humaines en soins oculaires.*

**Instructions**

*Je vous remercie de prendre le temps de remplir ce questionnaire. Nous vous serions reconnaissants de fournir les chiffres, mais si cela n'est pas possible, nous serions reconnaissants si vous pouviez fournir des estimations, en indiquant si les données sont des estimations. Si les réponses à certaines des questions ne sont pas connues ou ne peuvent pas être estimées, s'il vous plaît laissez-les vides, et remplissez les sections que vous pouvez remplir.

Nous prévoyons qu’une personne ne peut pas avoir toutes les réponses, mais à travers vos réseaux, nous espérons que l'information sera connue.

Si vous avez des questions s'il vous plaît contacter Jennifer Palmer à la London School of Hygiene and Tropical Medicine*, jennifer.palmer@ lshtm.ac.uk

*Pourriez-vous retourner le questionnaire avant le 21 novembre 2012 durant l’atelier de Dakar ou par email.

Les définitions des termes utilisés peuvent être trouvées à l'appendice 1.*

**Questionnaire**

**Part 1 – Questions Générales**

| **1.1** | **Pays** | |  | | | | |
| --- | --- | --- | --- | --- | --- | --- | --- |
|  |  | |  | | | | |
| **1.2** | **Nom du répondant** | |  | | | | |
|  |  | |  | | | | |
| **1.3** | **Fonction du répondant** | |  | | | | |
|  |  | |  | | | | |
| **1.4** | **Adresse, tél et email du répondant** | |  | | | | |
|  | | | | |
|  | | | | |
|  |  | |  | | | | |
| **1.5** | **Population totale du pays? Quel est le nombre estimé de la population agée de plus de 50 ans? En quelle année a été faite  cette estimation?** | | Population totale | | Population âgée de +50 ans | | Année et source de cette estimation |
|  | |  | |  |
|  | | | | | | | |
| **1.6** | **Quelle est la prevalence estimée de la cécité en Guinée ? et de la catarcte** | | | | | | |
|  |  | - de 50 ans | | 50 et 50+ ans | | Tout âge confondu | |
| a | Cataracte |  | |  | |  | |
| b | Cécitée suite à une cataracte |  | |  | |  | |
| c | Baisse d’acuité visuelle inf à 3/10 suite à une cataracte |  | |  | |  | |
| Si possible, utilisez la prevalence éstimée. Donnez la source de cette estimation. Veuillez envoyer, en pièce jointe, le RAAB, si disponible | | | | | | | |
| Source d’information | |  | | | | | |

**Part 2 – Entrée dans le marché du travail – Facteurs internes et externes**

| **2.1** | **Veuillez noter, çi dessous, les noms des centres de formation disponibles dans votre pays** | | | | | | | | |
| --- | --- | --- | --- | --- | --- | --- | --- | --- | --- |
|  |  | **Type de qualification** | | | | | | | |
|  |  | Diplome | | | | Autres qualifications | | | |
| a | Ophthalmologiste |  | | | |  | | | |
|  | | | | | | | | | |
|  |  | Licence | Diplôme | | | Certificate | | | Autres |
| b | Infirmier ophthalmo  (TSO, ISO) |  |  | | |  | | |  |
|  | | | | | | | | | |
|  |  | BSc | | | | Autres | | | |
| c | Optometriste |  | | | |  | | | |
|  | | | | | | | | | |
|  |  | Diplome | | | Certificat | | | Autres | |
| d | Opérateur de cataracte ou équivalent |  | | |  | | |  | |
|  |  |  | |  | | |  | | |
|  |  | Diplome | | | Certificat | | | Autres | |
| e | Autres cadres de niveau intermédiaires |  | | |  | | |  | |
| Source d’information | |  | | | | | | | |
|  | | | | | | | | | |
| **2.2** | **Completer la tableau suivant:** | | | | | | | | |
|  |  | **Nombre de personnes qui ont terminé leur formation dans le pays ou à l’étranger en 2008, 2009 et 2010- Donner le total des 3 ans** | | | **Nombre total de personnel de santé oculaire qui ont été embauchés 2008,2009 et 2010-privé et étatique confondu** | | | **Idem, mais embauchés par les institutions étatiques** | |
| a | Ophthalmologiste |  | | |  | | |  | |
| b | Infirmier Ophthalmo |  | | |  | | |  | |
| c | Optometriste |  | | |  | | |  | |
| d | Opérateur de cataracte ou équivalent |  | | |  | | |  | |
| e | Autres cadres de niveau intermédiaires |  | | |  | | |  | |
| Source d’information | |  | | | | | | | |

**Part 3 – Marché du travail (personnels de santé oculaire engagés)**

| **3.1** | **Nomre de personnels engagés dans les différents types d’institutions de soins oculaires** | | | | | | | |
| --- | --- | --- | --- | --- | --- | --- | --- | --- |
|  |  | | Structures sanitaires étatiques | Missionnaires, ONG) | | Privées | | Total |
| a | Ophthalmologiste | |  |  | |  | |  |
| b | Infirmier ophthalmo | |  |  | |  | |  |
| c | Optométriste | |  |  | |  | |  |
| d | Opérateur de cataracte ou équivalent | |  |  | |  | |  |
| e | Autres cadres de niveau intermédiaires | |  |  | |  | |  |
| Source d’information | | |  | | | | | |
|  | | | | | | | | |
| **3.2** | **A quel niveau du système sanitaire et à quel endroit exercent les ophtalmologistes ?** | | | | | | | |
|  |  | La capitale | | | En dehors de la capitale | | Total | |
| a | Niveau primaire |  | | |  | |  | |
| b | Secondaire |  | | |  | |  | |
| c | Tertiaire |  | | |  | |  | |
| Source d’information | |  | | | | | | |
|  | | | | | | | | |
| **3.3** | **A quel niveau du système sanitaire et à quel endroit exercent les infirmiers ophthalmo ?** | | | | | | | |
|  |  | La capitale | | | En dehors de la capitale | | Total | |
| a | Niveau primaire |  | | |  | |  | |
| b | Secondaire |  | | |  | |  | |
| c | Tertiaire |  | | |  | |  | |
| Source of information | | Source d’information | | | | | | |
|  | | | | | | | | |
| **3.4** | **A quel niveau du système sanitaire et à quel endroit exercent les optométristes?** | | | | | | | |
|  |  | La capitale | | | En dehors de la capitale | | Total | |
| a | Primary |  | | |  | |  | |
| b | Secondary |  | | |  | |  | |
| c | Tertiary |  | | |  | |  | |
| Source of information | |  | | | | | | |
|  | | | | | | | | |

| **3.5** | **A quel niveau du système sanitaire et à quel endroit exercent les opérateurs de cataracte ?** | | | | | | |
| --- | --- | --- | --- | --- | --- | --- | --- |
|  |  | | | La capitale | | En dehors de la capitale | Total |
| a | Niveau primaire | | |  | |  |  |
| b | Secondaire | | |  | |  |  |
| c | Tertiaire | | |  | |  |  |
| Source of information | | | |  | | | |
|  | | |  |  | |  |  |
| **3.6** | | | **A quel niveau du système sanitaire et à quel endroit exercent les agents intermediaries?** | | | | |
|  | | |  | La capitale | | En dehors de la capitale | Total |
| a | Niveau primaire | | |  | |  |  |
| b | Secondaire | | |  | |  |  |
| c | Tertiaire | | |  | |  |  |
| Source of information | | | |  | | | |
|  | |  | |  | |  |  |
| **3.7** | | **Parmi le type de personnel suivant, combien sont de la nationalité de votre pays ?** | | | | | |
| a | | Ophthalmologiste | | |  | | |
| b | | Infirmier ophthalmo | | |  | | |
| c | | Optométriste | | |  | | |
| d | | Opérateur de cataracte ou équivalent | | |  | | |
| e | | Autres cadres de niveau intermédiaires | | |  | | |
|  | | Source d’information | | | | | |

**Part 4 – Sorties du marché du travail**

| **4.1** | **Nombre de personnes qui ont quitté leur poste (toute les raisons confondues-maladie, décès, départ à l’étranger, retraite, etc.) durant les 3 dernières années** | | | | |
| --- | --- | --- | --- | --- | --- |
|  |  | Governement | NGO | Privé | Total |
| a | Ophthalmologiste |  |  |  |  |
| b | Infirmier ophthalmo |  |  |  |  |
| c | Optométriste |  |  |  |  |
| d | Opérateur de cataracte ou équivalent |  |  |  |  |
| e | Autres cadres de niveau intermédiaires |  |  |  |  |
| f | Je ne sais pas (metre une croix) |  |  |  |  |
| Source d’ information | |  | | | |
|  | | | | | |

| **4.2** | **Pourcentage du personnel qui ont émigrés durant les 5 dernières années (mettre un croix)** | | | | | |
| --- | --- | --- | --- | --- | --- | --- |
|  |  | Ophthalmologistes | Infirmiers Ophthalmo | Optometristes | Opérateur de cataracte ou équivalent | Autres cadres de niveau intermédiaires |
| a | Aucun |  |  |  |  |  |
| b | Quelques un (1-20%) |  |  |  |  |  |
| c | Un bon nombre (21-50%) |  |  |  |  |  |
| d | La majorité (51-81%) |  |  |  |  |  |
| r | Presque tous (81-100%) |  |  |  |  |  |
| f | Je ne sais pas |  |  |  |  |  |
| Source d’information | |  | | | | |

**Part 5 – Prestation de soins et résultats**

| **5.1** | **Décrire le circuit type d’un patient de cataracte depuis son contact avec un agent de santé au village jusqu’à la sortie de l’hôpital.**  Par exemple:  1. Le patient visit le volontaire. Le patient est réferré pour une operation de cataracte.  2. Diagnostic par l’infimier ophthalmo dans l’hopital de district  3. Opération fatre par le doctor et trios personnels intermédiaires  4. Le patient reste à l’hopital regional pour une nuit  5. Le visite de verification est faite par l’infirmier le lendemain. | |
| --- | --- | --- |
|  | Source d’information: | |
|  |  | |
| **5.2** | **Nombre total de chirurgies de la cataracte en 2010 dans le pays** |  |
|  | | |
|  |  |  |
| **5.3** | **Quelle proportions de la chirurgie de la cataracte en 2010 était faite par les ophtalmologistes eux-mêmes ?** |  |

**Merci pour votre participation**

**Annexe1**

**Definitions[[1]](#endnote-2),[[2]](#endnote-3)**

**Ophthalmologiste, Optometriste, Infirmier ophthalmo**

Groupes de professionnels qui ont au moins reçu un diplome équivalent à la licence dans une des spécialités en santé oculaire.

**Opérateurs de cataracte**

Des personnels qui ne sont pas des docteurs mais ont recu une formation en chirurgie de la cataracte.

**Autres cadres de niveau intermédiaire**

Tout autre personnel pas inclus dans les autres catégories qui ont un rôle technique et d’appui dans l’offre de soins (par ex. infirmiers en bloc opératoire, assistants optomotétristes....).

**Niveau dans la pyramide sanitaire**

- Primaire – C’est le premier contact avec le patient et a généralement lieu au niveau de la communauté.
- Secondaire – Les patients sont référés du niveau secondaire. Soins plus spécialisé qu’au niveau primaire et généralement au niveau du district.
- Tertiaire – Les patients sont référés du niveau secondaire. Il s’agit des hôpitaux régionaux ou généralement se trouve l’ophtalmologiste.

**Employés par le gouvernement –** Ceci inclus le personnel de l’armée

**Note**

Lorsque le questionnaire precise l’année, veuillez fournir les données les plus récentes.

1. Human Resource Development Working Group – Vision2020. *Global Human Resource Development Assessment for Comprehensive Eye Care*. [Online] Available from: [http://www.vision2020.org/documents/WHO%20Publications/Global_Human_Resource_Development_Assessment_For_Comprehensive_Eye_Care.pdf](http://www.vision2020.org/documents/WHO Publications/Global_Human_Resource_Development_Assessment_For_Comprehensive_Eye_Care.pdf) [Accessed 24th March 2011]. [↑](#endnote-ref-2)
2. Para IABP HRD Programme Committee. Taxonomy of eye healthy occupations. [↑](#endnote-ref-3)
