# Supplementary material for: Mapping human resources for eye health in 21 countries of sub-Saharan Africa: current progress towards VISION 2020
Source: Hum Resour Health. 2014 Aug 15;12:44. doi: 10.1186/1478-4491-12-44 (PMC4237800; doi:10.1186/1478-4491-12-44)
Supplement: Additional file 3 — Country HReH analyses. [file 1478-4491-12-44-S3.pdf]

Mapping Human Resources for Eye Health in Sub-Saharan Africa: Progress towards VISION 2020

Country Fact Sheets

This study was funded by: Sightsavers

Index

Country Fact Sheets Methods 1

Benin 3

Botswana 7

Democratic Republic of Congo 11

Ethiopia 15

Gambia 19

Ghana 23

Kenya 27

Madagascar 31

Malawi 35

Mali 39

Nigeria 43

Rwanda 47

Senegal 51

Sierra Leone 55

Republic of South Sudan 59

Sudan 63

United Republic of Tanzania 67

Togo 71

Uganda 75

Zambia........79

Zimbabwe........83

Country Fact Sheet Methods

Information on human resources for eye health (HReH) in 21 Sub-Saharan African countries is presented here from a study conducted by the International Centre for Eye Health and African Visions Research Institute. A brief outline is provided here to describe the methods used in the Country Fact Sheets.

Sources of HReH Data

Questionnaires were sent to national eye care coordinators and other key informants to provide information on three areas:

1. **Active** workforce – practitioners currently in the workforce, including location and sector
2. **Entry** into the eye care workforce in the last three years – practitioners who were recently trained and recruited into the workforce, including recent graduates, immigrants and those who re-entered the sector
3. **Exit** from the workforce over the last three years – practitioners who have recently left the workforce due to retirement, emigration, death and temporary exit to pursue a new field of work

Informants were encouraged to use several sources of data including state/district eye care coordinators, professional networks (ophthalmologists and optometrists' associations), HReH training institutions and eye care NGOs or researchers who had conducted recent surveys. Informants were requested to provide information on the entire HReH workforce, including those in non-public facilities.

Sources of Population Data

Population data were taken from the sources below, using expected rates of annual population increase to estimate populations for specific years (e.g. 2011 population = 2010 population + expected population growth in one year). The expected population increase over the 9-year period of 2011 to 2020 was then calculated for the general population and the population over 50 years of age to show whether a country has an ageing population or not. The same projection was calculated for the capital city population to show the expected effects of urbanisation.

- General population for all countries
- General population for Sudan. 2020 population was projected using a 1.03% annual growth rate
- South Sudan: South Sudan National Bureau of Statistics, personal communication
- Over 50 population data
- Capital City Population

VISION 2020 Targets and HReH Cadres

Countries were tracked against the VISION 2020 targets below:

VISION 2020 targets per 1,000,000 population	
Ophthalmologists	4
Cataract surgeons	
Ophthalmic clinical officers	10
Ophthalmic nurses	
Optometrists	20
Mid-level refractionists	

Each target was graded into three categories to show whether or not the country had met VISION 2020 targets in 2011:

- **Green**: VISION 2020 target has already been met in 2011
- **Orange**: The country was above one quarter of the VISION 2020 target in 2011
- **Red**: The country was at less than one quarter of the VISION 2020 target in 2011

HReH Workforce Dynamics

Practitioner per population ratios were calculated for the overall population and compared with their respective VISION 2020 targets. The target number of practitioners for the country was calculated and compared to the number in the active workforce to estimate the practitioner shortage in 2011.

Practitioner per population ratios were also calculated for those working inside and outside the capital using capital city and outside capital populations. This highlights the geographic distribution of eye care practitioners within each country.

The three-year net change in numbers of practitioners in each cadre over the last three years was calculated as the total number who entered the workforce minus the total who exited. This was multiplied by three to calculate the net change expected over the nine years from 2011 to 2020. This was then added to the number of practitioners in the active workforce in 2011 to calculate the projected numbers in the active workforce in 2020, assuming that current trends in entry and exit will continue over this period. The projected 2020 population was then used to calculate the projected practitioner per population ratios in 2020. The projected practitioner shortage in 2020 was calculated as above.

VISION 2020 Targets and CSR

VISION 2020 targets	
Cataract surgeries performed per 1,000,000 population	2,000
Cataract surgeries performed per surgeon	500

Questionnaires reported the total number of cataract surgeries performed in the country in all sectors in a recent year (varying between 2008 and 2012 per country, as noted on each country fact sheet). The cataract surgical ratio (CSR) was calculated by dividing the total number of surgeries performed in the country by the total population in 2011. The target number of surgeries for the country was then calculated and compared to the number performed to estimate the shortage of cataract surgeries in 2011.

The ratio of cataract surgeries per surgeon (surgical performance ratio) was calculated by dividing the total number of surgeries performed in the country by the total number of ‘surgeons’ (ophthalmologists and cataract surgeons) in the active workforce. National eye care coordinators were asked to use personal knowledge to estimate the proportion of cataract surgeries performed by ophthalmologists and it was assumed that the remainder of cataract surgeries were performed by cataract surgeons. These estimates were then use to calculate specific surgeries per surgeon ratios for each cadre in 2011.

It was assumed that there would be no change in surgical performance between 2011 and 2020, so the ratio of surgeries per surgeon for each cadre would be assumed to remain the same in 2020. The projected number of cataract surgeries that will be performed in 2020 was calculated by multiplying the 2011 surgical performance rate of each cadre by the projected number of practitioners in each cadre in the active workforce in 2020. This number was then divided by the projected 2020 population to calculate the CSR in 2020 and hence the projected shortage in 2020 to meet the VISION 2020 target, as calculated above.

Country Profile: Benin

Key Messages

- **Surgeons:** The surgeon to population ratio is projected to decrease in Benin between 2011 and 2020. Unless this trend changes, Benin will never meet this target.
- **OCOs/Nurses:** The number of ophthalmic nurses is projected to decrease. Benin is not set to meet this target.
- **Refractionists:** Benin has 4 refractionists, a number which has not changed over the past 3 years. Benin is not set to meet this VISION 2020 target.
- **Cataract Surgeries:** The number of cataract surgeries performed will not increase as fast as population growth. By 2020 Benin is projected to be a quarter of the way to meeting this VISION 2020 target.

VISION 2020 Targets

	Eye Care Practitioners per Million Population			Cataract Surgeries Performed per Million Population	Cataract Surgeries Performed per Surgeon
	Surgeons*	Ophthalmic Clinical Officers /Nurses	Refraction-ists		
VISION2020 Target	4	10	20	2,000	500
2011 Situation	3.1	6.9	0.4	550	179
On Track					

* For the Africa region this includes Ophthalmologists and Cataract Surgeons

Eye Care Practitioners: % Working Inside/Outside Capital

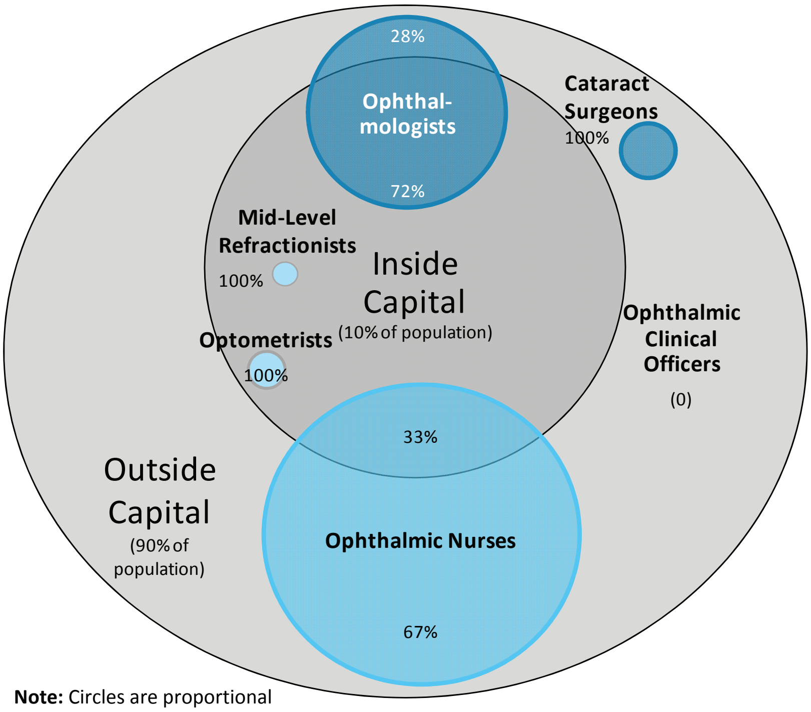

Note: Circles are proportional to numbers of eye care practitioners

In-Country Training Programmes

Surgeons	OCOs/Nurses	Refractionists
Total: 1	Total: 0	Total: 0

Distribution of Refractionists

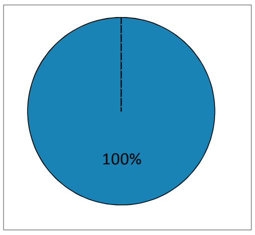

Refractionists based in the capital

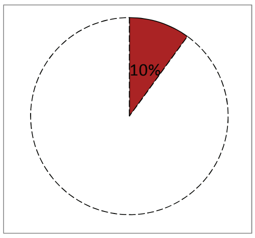

Population living in the capital

100% of Refractionists treat 10% of the population

Eye Care Practitioners: Split between Sectors

Government | NGO/Mission | Private for Profit

Surgeons

Ophthalmologists

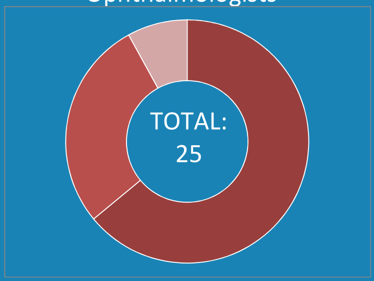

Cataract Surgeons

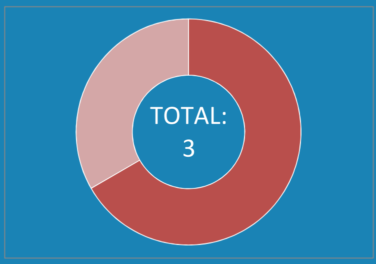

OCOs/Nurses

Ophthalmic Clinical Officers

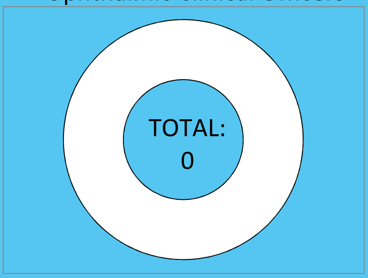

Ophthalmic Nurses

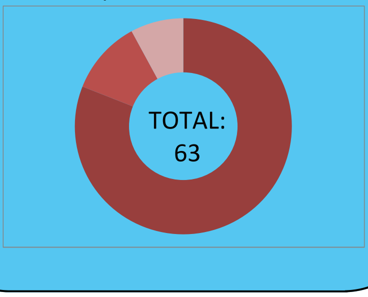

Refractionists

Optometrists

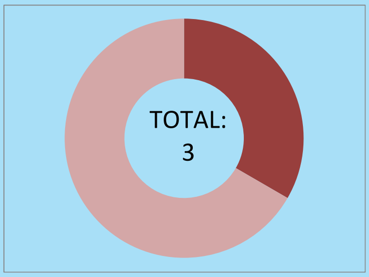

Mid-level Refractionists

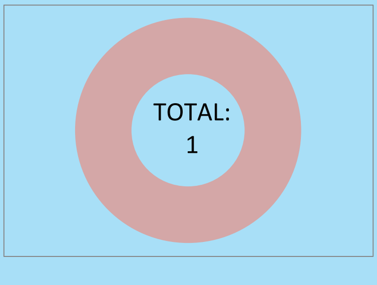

Surgeons: Current & Projected Workforce per Million Population

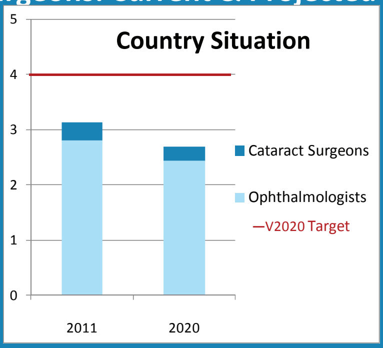

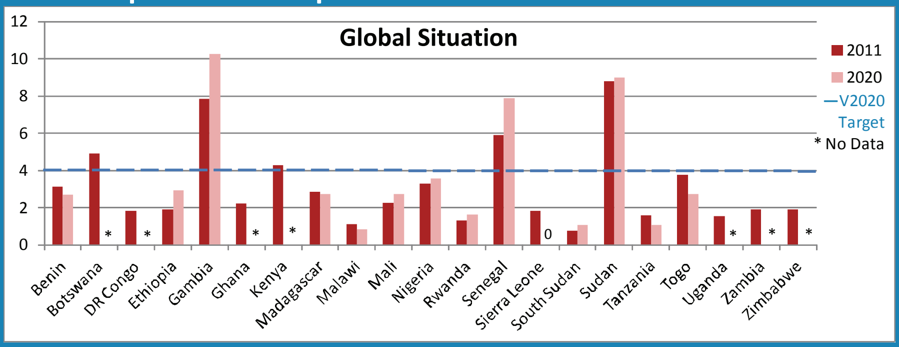

Practitioner Entry vs Exit: No projected change in the numbers of cataract surgeons is expected between 2011 and 2020 and a projected net increase of 1 ophthalmologist every 3 years.

Practitioner Working Location: Although Benin is currently close to meeting the VISION 2020 target, with a practitioner to population ratio of 3.1, this is not the case for practitioners based outside of Cotonou, for which the ratio is 1.2 and projected to decrease to 1.1 by 2020.

Practitioner vs Population Growth: The surgeon practitioner growth rate is expected to be below the general population growth rate, meaning that the practitioner to population ratio will decrease by 2020 if current trends continue.

OCOs/Nurses: Current & Projected Workforce per Million Population

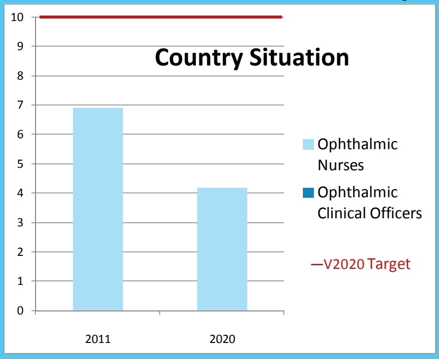

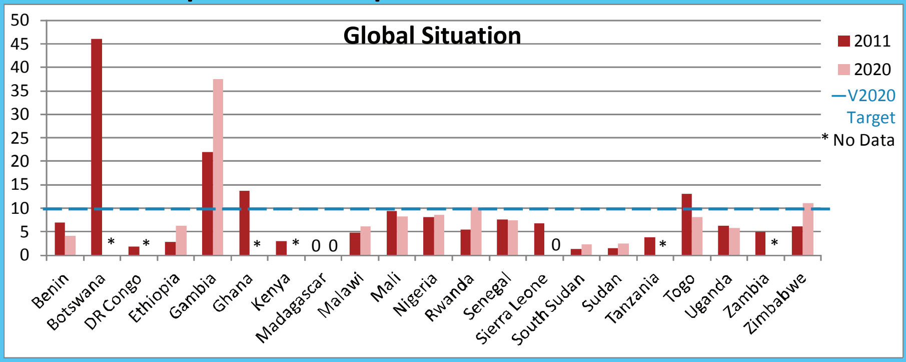

Practitioner Entry vs Exit: Over the previous three years, there have been no ophthalmic nurses entering the workforce, only exiting. If this trend continues, Benin will never meet this VISION 2020 target.

Practitioner Working Location: The overall practitioner to population ratio of 6.9, is 22.7 for those working in Cotonou compared with 5.1 for those working outside .

Practitioner vs Population Growth: With a negative growth in practitioner numbers, combined with an expected 27% population increase over this 9-year period, the practitioner to population ratio is projected to decrease from 6.9 in 2011 to 4.2 in 2020.

Refractionists: Current & Projected Workforce per Million Population

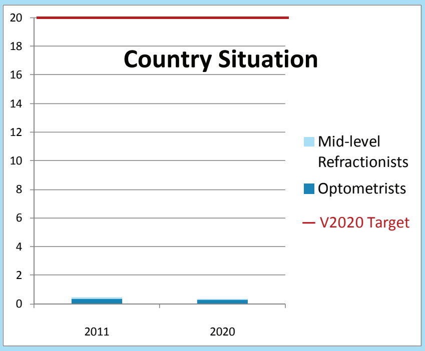

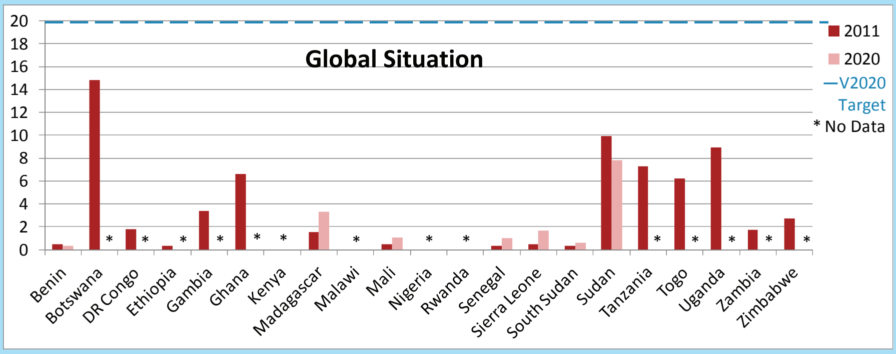

Practitioner Entry vs Exit: Benin has 4 refractionists currently working and over the past 3 years there has been no entry or exit from the workforce. If this trend continues, Benin will never meet the VISION 2020 target, as the country requires 230 refractionists.

Practitioner Working Location: All 4 of the refractionists work in Cotonou, treating 11% of the population.

Practitioner vs Population Growth: Between 2011 and 2020 the population of Benin is expected to increase by 27%, whereas these practitioner numbers are not set to change.

Cataract Surgical Performance: Current & Projected Performance per Million Population

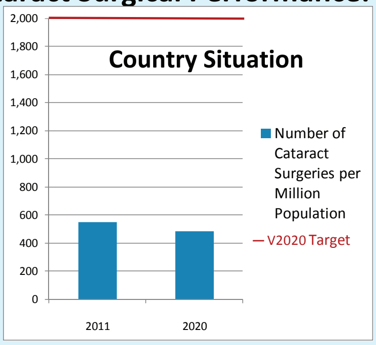

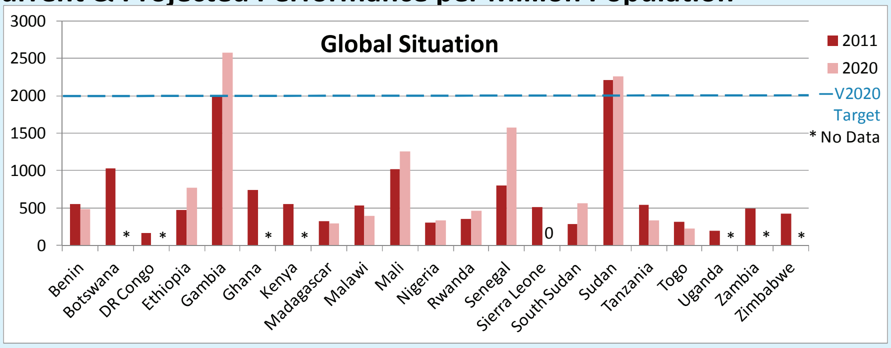

With a surgeon population growing at a slower rate to general population growth, cataract surgeries are projected to decrease between 2011 and 2020, rather than the 260% increase that is needed to meet the VISION 2020 target.

Country Profile: Benin

Current Situation: 2011

Total Population	Population Living in Capital City *	% Population Living in Capital	Population Over 50	% Population Over 50
9,086,624	924,000	10%	934,000	10%

VISION 2020 Targets

Eye Care Cadre	# Practitioners per Million Population
Surgeons	4
OCOs/Nurses	10
Refractionists	20

Characteristics of the Active Eye Care Practitioner Workforce: 2011

Eye Care Cadre	Number of Practitioners in Active Workforce	Sector			Location		# Training Programmes available in Country
		Government	NGO/ Mission	Private for Profit	Capital	Outside Capital	
Ophthalmologists	25	16	7	2	18	7	1
Cataract Surgeons	3	0	2	1	0	3	0
**Surgeons**	**28**	**16**	**9**	**3**	**18**	**10**	**1**
Ophthalmic Clinical Officers	0	0	0	0	0	0	0
Ophthalmic Nurses	63	51	7	5	21	42	0
**OCOs/Nurses**	**63**	**51**	**7**	**5**	**21**	**42**	**0**
Optometrists	3	1	0	2	3	0	0
Mid-level Refractionists	1	0	0	1	1	0	0
**Refractionists**	**4**	**1**	**0**	**3**	**4**	**0**	**0**

Eye Care Practitioner Workforce Dynamics: 2011

Eye Care Cadre	Number of Practitioners in Active Workforce	Practitioners per Million Population			VISION 2020 Country Target # of Practitioners	Shortage in Practitioners to meet Target
		Countrywide	In Capital	Outside Capital		
Ophthalmologists	25	2.8	19.5	0.9		
Cataract Surgeons	3	0.3	0.0	0.4		
**Surgeons**	**28**	**3.1**	**19.5**	**1.2**	**36**	**8**
Ophthalmic Clinical Officers	0	0.0	0.0	0.0		
Ophthalmic Nurses	63	6.9	22.7	5.1		
**OCOs/Nurses**	**63**	**6.9**	**22.7**	**5.1**	**91**	**28**
Optometrists	3	0.3	3.2	0.0		
Mid-level Refractionists	1	0.1	1.1	0.0		
**Refractionists**	**4**	**0.4**	**4.3**	**0.0**	**182**	**178**

Annual Cataract Surgical Performance

Number of Cataract Surgeries Performed (data from 2010)	5,000
Number of Cataract Surgeries per Surgeon (surgical performance ratio)	179
% Surgeries Performed by Ophthalmologists (estimate)	95%
Number of Cataract Surgeries per Million Population (CSR)	550
Target Number of Cataract Surgeries to meet VISION 2020 Target	18,173
Shortage in Cataract Surgeries to meet VISION 2020 Target	13,173

* Cotonou data was used for “Capital City

Projected Situation: 2020

Projected Total Population	Projected Population Living in Capital City	% Projected Population Living in Capital	Projected Population Over 50	% Projected Population Over 50	Expected 9-year Population Increase	Expected 9-year Over 50 Population Increase
11,523,481	1,312,352	11%	1,279,000	11%	27%	37%

Projected Eye Care Practitioner Workforce Dynamics: 2020

Eye Care Cadre	Number of Practitioners in Active Workforce (2011)	Over last 3 years			Projected Net Change over next 9 years	Projected Number of Practitioners in Active Workforce	Projected Practitioners per Million Population			VISION 2020 Country Target # of Practitioners	Projected Shortage in Practitioners to meet VISION 2020
		Number Entered Workforce	Number Exited Workforce	Net Change in Practitioner Numbers			Countrywide	In Capital	Outside Capital		
Ophthalmologists	25	4	3	1	3	28	2.4	15.4	0.8		
Cataract Surgeons	3	0	0	0	0	3	0.3	0.0	0.3		
**Surgeons**	**28**	**4**	**3**	**1**	**3**	**31**	**2.7**	**15.2**	**1.1**	**46**	**15**
Ophthalmic Clinical Officers	0	0	0	0	0	0	0.0	0.0	0.0		
Ophthalmic Nurses	63	0	5	-5	-15	48	4.2	12.2	3.1		
**OCOs/Nurses**	**63**	**0**	**5**	**-5**	**-15**	**48**	**4.2**	**12.2**	**3.1**	**115**	**67**
Optometrists	3	0	0	0	0	3	0.3	2.3	0.0		
Mid-level Refractionists	1	0	0	0	0	1	0.1	0.8	0.0		
**Refractionists**	**4**	**0**	**0**	**0**	**0**	**4**	**0.4**	**3.0**	**0.0**	**230**	**226**

Annual Projected Cataract Surgical Performance: 2020

Eye Care Cadre	% Surgeries Performed (estimate)	Surgical Performance Ratio per Cadre (2011)	Projected Number of Surgeons in Active Workforce	Projected Number of Cataract Surgeries Performed	Projected Number of Cataract Surgeries per Million Population (CSR)	Target Number of Cataract Surgeries to meet VISION 2020 Target	Projected Shortage in Cataract Surgeries to meet VISION 2020 Target
Ophthalmologists	95%	190	28	5,320			
Cataract Surgeons	5%	83	3	250			
**Surgeons**	**100%**	**179**	**31**	**5,570**	**483**	**23,047**	**17,477**

Country Profile: Botswana

Key Messages

- **Surgeons:** Botswana currently meets this VISION 2020 target, due to the presence of ophthalmologists. It is not clear whether this will still be the case by 2020 due to lack of data.
- **OCOs/Nurses:** Botswana is well above this VISION 2020 target, due to the presence of ophthalmic nurses in the workforce. There are no ophthalmic clinical officers in Botswana.
- **Refractionists:** Botswana is three-quarters of the way to reaching this VISION 2020 target. To meet this target, Botswana will need to increase the workforce by 50% by 2020.
- **Cataract Surgeries:** Botswana is half-way to meeting this VISION 2020 target. However, it is not clear whether this target will be met by 2020 due to lack of data.

VISION 2020 Targets

	Eye Care Practitioners per Million Population			Cataract Surgeries Performed per Million Population	Cataract Surgeries Performed per Surgeon
	Surgeons*	Ophthalmic Clinical Officers /Nurses	Refractionists		
VISION2020 Target	4	10	20	2,000	500
2011 Situation	4.9	46.0	14.8	1,027	208
On Track					

* For the Africa region this includes Ophthalmologists and Cataract Surgeons

Eye Care Practitioners: % Working Inside/Outside Capital

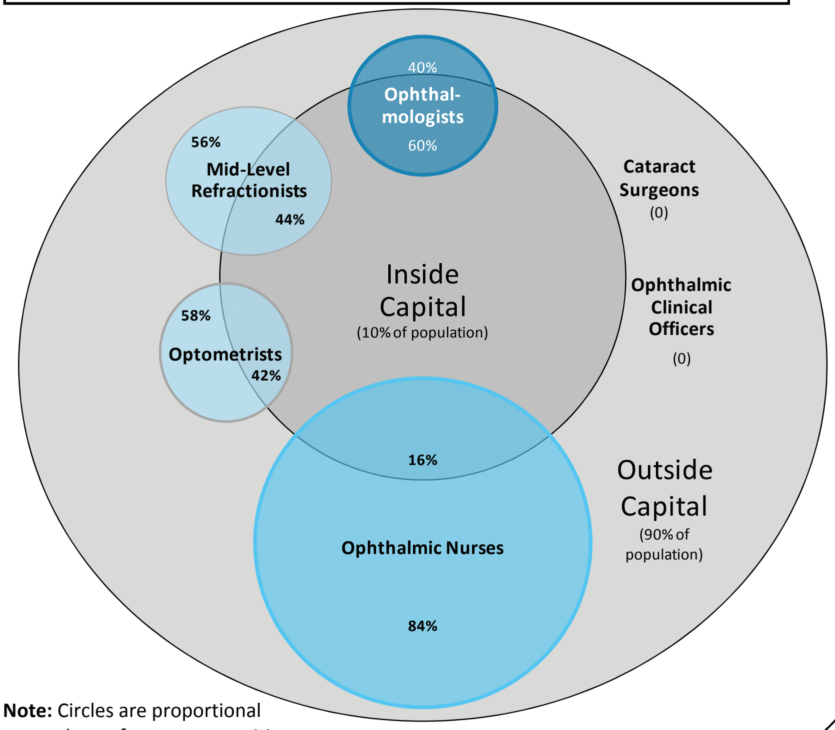

Note: Circles are proportional to numbers of eye care practitioners

In-Country Training Programmes

Surgeons	OCOs/Nurses	Refractionists
Total: 0	Total: 1	Total: 0

Distribution of Ophthalmologists

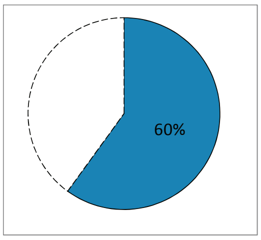

Ophthalmologists based in the capital

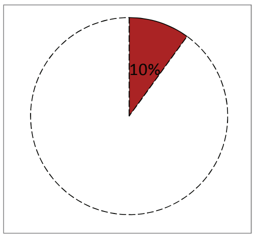

Population living in the capital

60% of Ophthalmologists treat 10% of the population

Eye Care Practitioners: Split between Sectors

Government | NGO/Mission | Private for Profit

Surgeons

Ophthalmologists

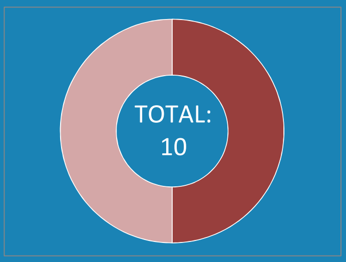

Cataract Surgeons

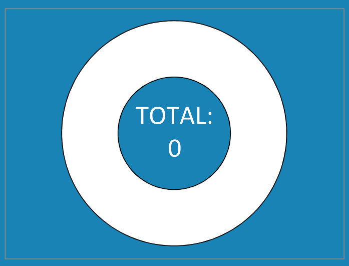

OCOs/Nurses

Ophthalmic Clinical Officers

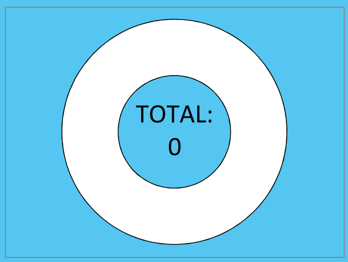

Ophthalmic Nurses

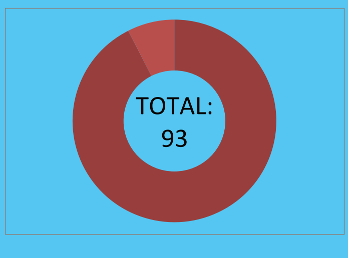

Refractionists

Optometrists

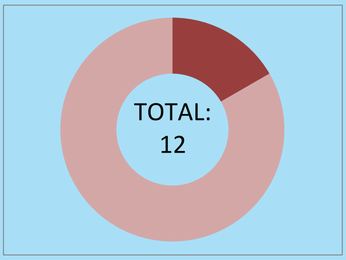

Mid-level Refractionists

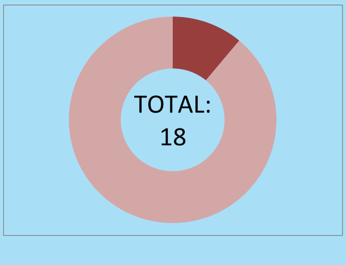

Surgeons: Current & Projected Workforce per Million Population

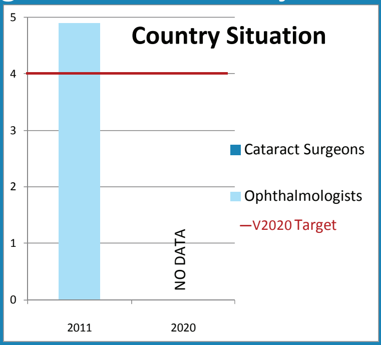

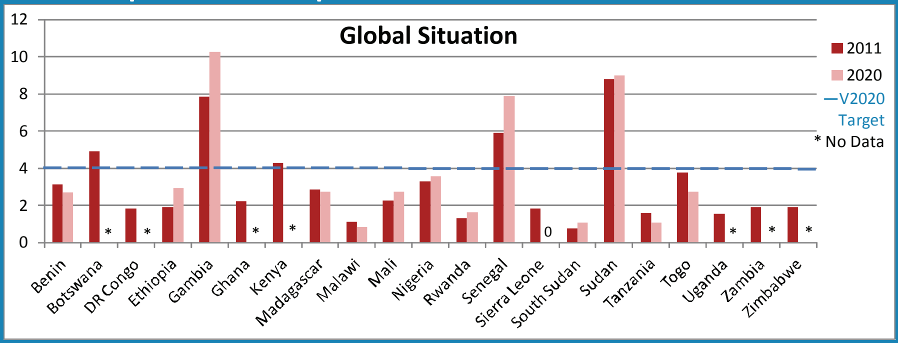

Practitioner Entry vs Exit: Over the past 3 years, 3 government-employed ophthalmologists have exited the workforce. However, there is no information on how many have entered the workforce or exited from the other sectors, so 2020 projections are not available.

Practitioner Working Location: With a ratio of 4.9, Botswana currently exceeds the VISION 2020 target for practitioner to population ratio. The ratio for those working in the capital is 29.7 and 2.2 for those working outside the capital.

Practitioner vs Population Growth: The population of Botswana is expected to increase by 9% between 2011 and 2020. So long as no further ophthalmologists leave the workforce, Botswana will still meet the VISION 2020 target by 2020.

OCOs/Nurses: Current & Projected Workforce per Million Population

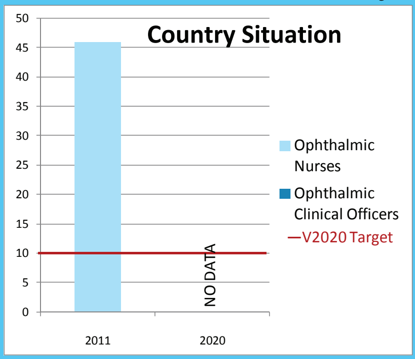

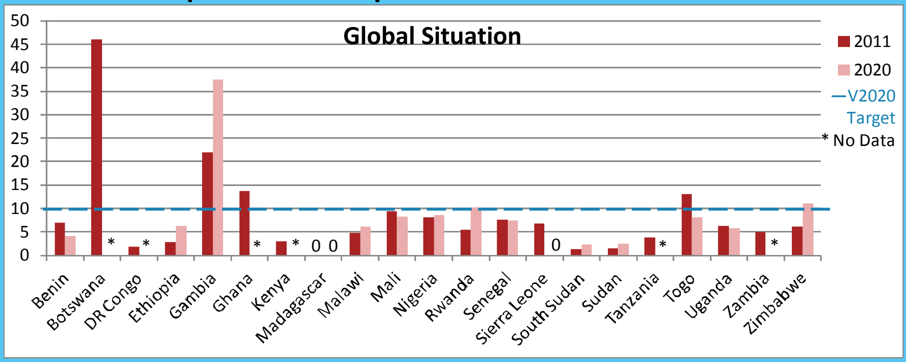

Practitioner Entry vs Exit: Over the past 3 years, 19 government-employed nurses have exited the workforce. However, there is no information on how many have entered the workforce or exited from other sectors, so projections for the situation in 2020 cannot be made.

Practitioner Working Location: 16% of ophthalmic nurses work in the capital, where 10% of the population live. When the practitioner to population ratio is split by location, the VISION 2020 is still far exceeded in both areas.

Practitioner vs Population Growth: Botswana currently has over four times as many ophthalmic nurses as required by the VISION 2020 target.

Refractionists: Current & Projected Workforce per Million Population

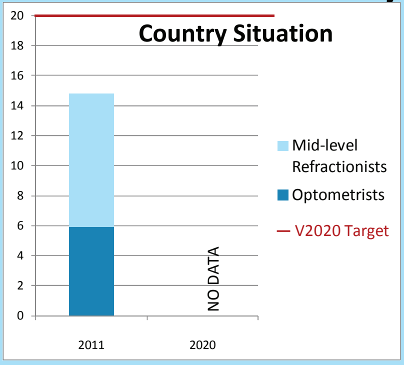

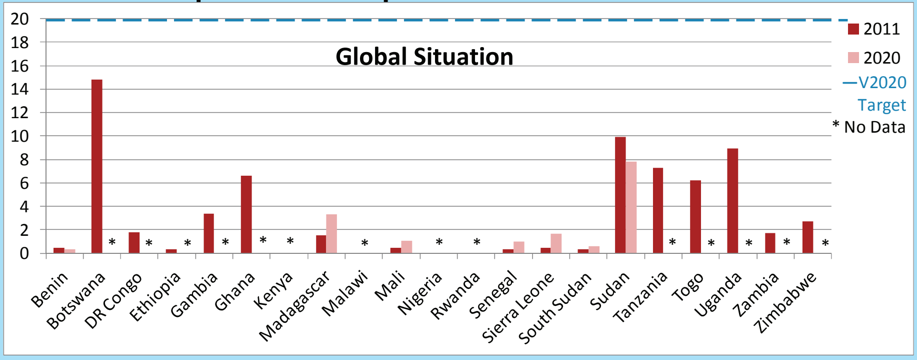

Practitioner Entry vs Exit: No data is available on entry and exit of the workforce.

Practitioner Working Location: The practitioner to population ratio for those working in the capital is 64.4 compared to 9.3 for those working outside the capital.

Practitioner vs Population Growth: Botswana will need to recruit a further 14 refractionists to meet this target by 2020, representing a 47% increase.

Cataract Surgical Performance: Current & Projected Performance per Million Population

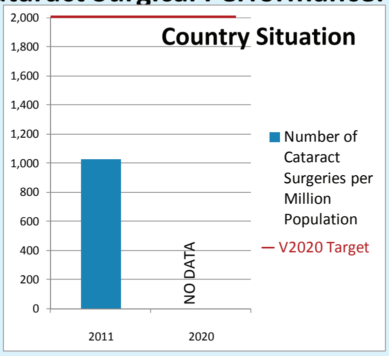

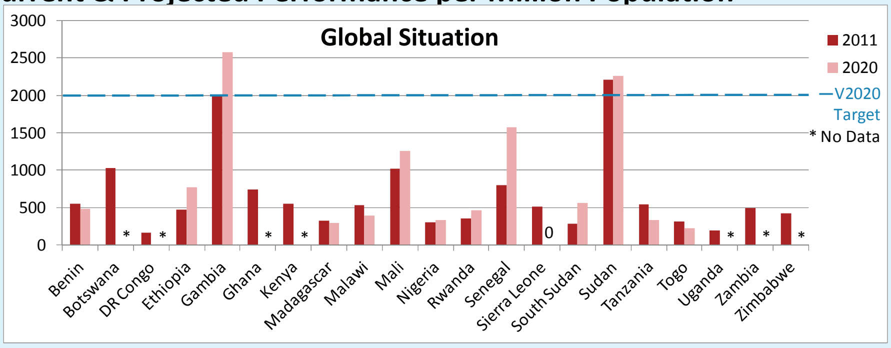

Taking into account the increase in population, Botswana will need to more than double the annual number of cataract surgeries performed in order to meet this target by 2020.

Country Profile: Botswana

Current Situation: 2011

Total Population	Population Living in Capital City	% Population Living in Capital	Population Over 50	% Population Over 50
2,025,916	202,000	10%	226,000	11%

VISION 2020 Targets

Eye Care Cadre	# Practitioners per Million Population
Surgeons	4
OCOs/Nurses	10
Refractionists	20

Characteristics of the Active Eye Care Practitioner Workforce: 2011

Eye Care Cadre	Number of Practitioners in Active Workforce	Sector			Location		# Training Programmes available in Country
		Government	NGO/ Mission	Private for Profit	Capital	Outside Capital	
Ophthalmologists	10	5	0	5	6	4	0
Cataract Surgeons	0	0	0	0	0	0	0
**Surgeons**	**10**	**5**	**0**	**5**	**6**	**4**	**0**
Ophthalmic Clinical Officers	0	0	0	0	0	0	0
Ophthalmic Nurses*	93	86	7	0	14	72	1
**OCOs/Nurses**	**93**	**86**	**7**	**0**	**14**	**72**	**1**
Optometrists	12	2	0	10	5	7	0
Mid-level Refractionists	18	2	0	16	8	10	0
**Refractionists**	**30**	**4**	**0**	**26**	**13**	**17**	**0**

Eye Care Practitioner Workforce Dynamics: 2011

Eye Care Cadre	Number of Practitioners in Active Workforce	Practitioners per Million Population			VISION 2020 Country Target # of Practitioners	Shortage in Practitioners to meet Target
		Countrywide	In Capital	Outside Capital		
Ophthalmologists	10	4.9	29.7	2.2		
Cataract Surgeons	0	0	0.0	0.0		
**Surgeons**	**10**	**4.9**	**29.7**	**2.2**	**8**	**Target Met**
Ophthalmic Clinical Officers	0	0	0.0	0.0		
Ophthalmic Nurses	93	46	69.3	39.5		
**OCOs/Nurses**	**93**	**46**	**69.3**	**39.5**	**20**	**Target Met**
Optometrists	12	5.9	24.8	3.8		
Mid-level Refractionists	18	8.9	39.6	5.5		
**Refractionists**	**30**	**14.8**	**64.4**	**9.3**	**41**	**11**

Annual Cataract Surgical Performance

Number of Cataract Surgeries Performed (data from 2010)**	2,081
Number of Cataract Surgeries per Surgeon (surgical performance ratio)	208
% Surgeries Performed by Ophthalmologists (estimate)	100%
Number of Cataract Surgeries per Million Population (CSR)	1,027
Target Number of Cataract Surgeries to meet VISION 2020 Target	4,052
Shortage in Cataract Surgeries to meet VISION 2020 Target	1,971

* Location data for Ophthalmic Nurses comes from the Government Sector only.

** Cataract Surgery Data from Government Facilities only

Projected Situation: 2020						
Projected Total Population	Projected Population Living in Capital City	% Projected Population Living in Capital	Projected Population Over 50	% Projected Population Over 50	Expected 9-year Population Increase	Expected 9-year Over 50 Population Increase
2,204,932	238,299	11%	254,000	12%	9%	12%

Projected Eye Care Practitioner Workforce Dynamics: 2020											
Eye Care Cadre	Number of Practitioners in Active Workforce (2011)	Over last 3 years			Projected Net Change over next 9 years	Projected Number of Practitioners in Active Workforce	Projected Practitioners per Million Population			VISION 2020 Country Target # of Practitioners	Projected Shortage in Practitioners to meet VISION 2020
		Number Entered Workforce	Number Exited Workforce+	Net Change in Practitioner Numbers			Countrywide	In Capital	Outside Capital		
Ophthalmologists	10	ND	3	ND	ND	ND	ND	ND	ND		
Cataract Surgeons	0	0	0	0	0	0	0.0	0.0	0.0		
**Surgeons**	**10**	**ND**	**3**	**ND**	**ND**	**ND**	**ND**	**ND**	**ND**	**9**	**ND**
Ophthalmic Clinical Officers	0	0	0	0	0	0	0.0	0.0	0.0		
Ophthalmic Nurses	93	ND	19	ND	ND	ND	ND	ND	ND		
**OCOs/Nurses**	**93**	**ND**	**19**	**ND**	**ND**	**ND**	**ND**	**ND**	**ND**	**22**	**ND**
Optometrists	12	ND	ND	ND	ND	ND	ND	ND	ND		
Mid-level Refractionists	18	ND	ND	ND	ND	ND	ND	ND	ND		
**Refractionists**	**30**	**ND**	**ND**	**ND**	**ND**	**ND**	**ND**	**ND**	**ND**	**44**	**ND**

Annual Projected Cataract Surgical Performance: 2020							
Eye Care Cadre	% Surgeries Performed (estimate)	Surgical Performance Ratio per Cadre (2011)	Projected Number of Surgeons in Active Workforce	Projected Number of Cataract Surgeries Performed	Projected Number of Cataract Surgeries per Million Population (CSR)	Target Number of Cataract Surgeries to meet VISION 2020 Target	Projected Shortage in Cataract Surgeries to meet VISION 2020 Target
Ophthalmologists	100%	208	ND	ND			
Cataract Surgeons	0	0	0	0			
**Surgeons**	**100%**	208	**ND**	**ND**	**ND**	**4,410**	**ND**

+ Exit data for all Practitioners comes from the Government Sector only

ND: No Data

Country Profile: Democratic Republic of Congo

Key Messages

- **Surgeons:** DR Congo is less than half way to meeting this VISION 2020 target. To meet this target, the surgeon workforce will need to nearly triple.
- **OCOs/Nurses:** DR Congo is less than 20% towards meeting this target and will need to recruit more than 700 OCOs and ophthalmic nurses by 2020 to meet the target.
- **Refractionists:** DR Congo has one optometrist and 120 mid-level refractionists. To meet this VISION 2020 target, DR Congo will need to recruit more than 1,500 refractionists by 2020.
- **Cataract Surgeries:** DR Congo is less than a tenth of the way towards meeting this target and needs to perform 12 times as many cataract surgeries in order to meet this target by 2020.

VISION 2020 Targets

	Eye Care Practitioners per Million Population			Cataract Surgeries Performed per Million Population	Cataract Surgeries Performed per Surgeon
	Surgeons*	Ophthalmic Clinical Officers /Nurses	Refraction-ists		
VISION2020 Target	4	10	20	2,000	500
2011 Situation	1.8	1.9	1.8	163	90
On Track					

* For the Africa region this includes Ophthalmologists and Cataract Surgeons

Eye Care Practitioners: % Working Inside/Outside Capital

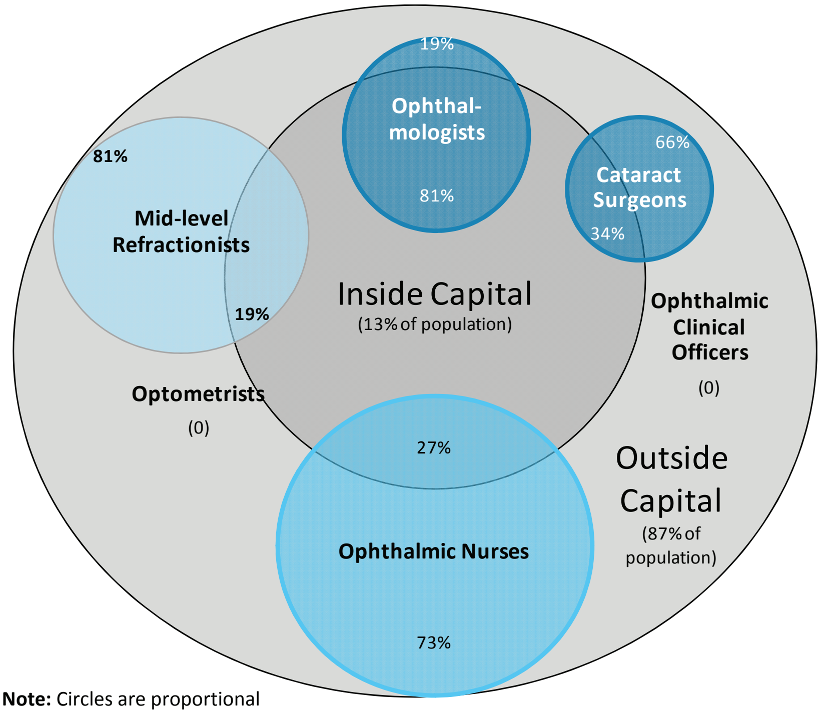

Note: Circles are proportional to numbers of eye care practitioners

In-Country Training Programmes

Surgeons	OCOs/Nurses	Refractionists
Total: 2	Total: 2	Total: 0

Distribution of Ophthalmologists

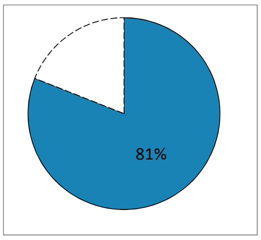

Ophthalmologists based in the capital

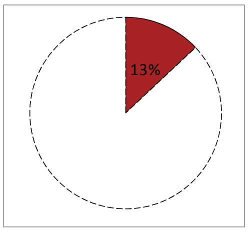

Population living in the capital

81% of Ophthalmologists treat 13% of the population

Eye Care Practitioners: Split between Sectors

Government | NGO/Mission | Private for Profit

Surgeons

Ophthalmologists

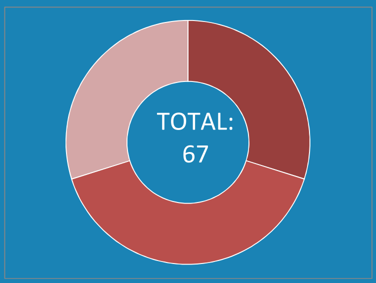

Cataract Surgeons

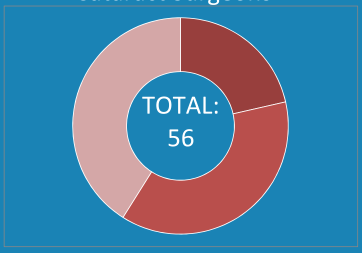

OCOs/Nurses

Ophthalmic Clinical Officers

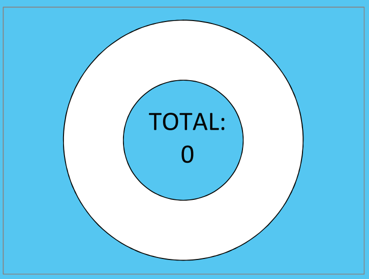

Ophthalmic Nurses

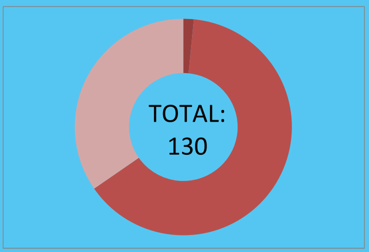

Refractionists

Optometrists

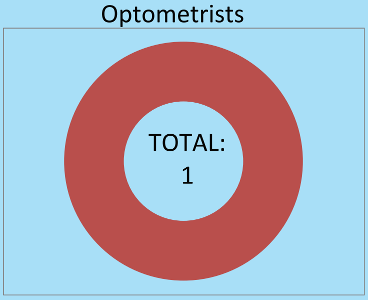

Mid-level Refractionists

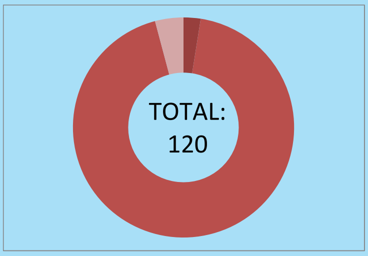

Surgeons: Current & Projected Workforce per Million Population

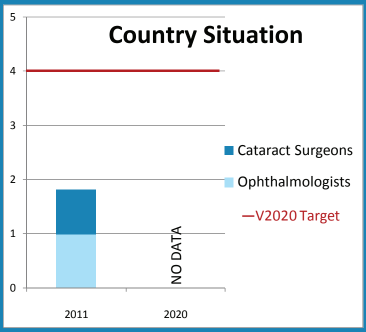

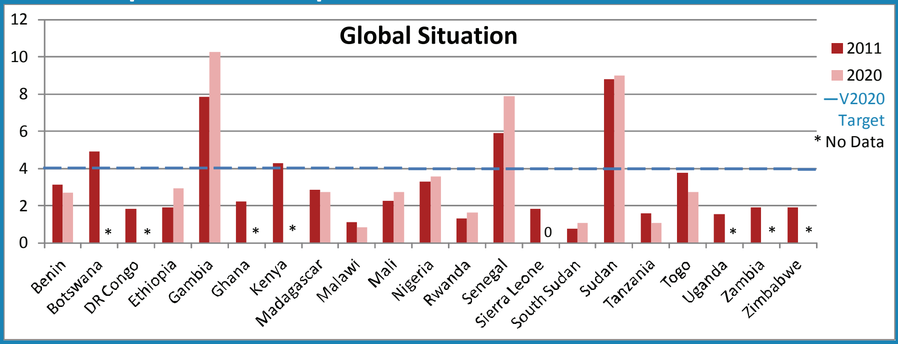

Practitioner Entry vs Exit: Over the past 3 years, 28 surgeons have entered the workforce. However, there is no information on how many have exited the workforce, so projections for the situation in 2020 cannot be made.

Practitioner Working Location: The practitioner to population ratio for those working in the capital is 8.3 compared to 0.8 for those working outside the capital.

Practitioner vs Population Growth: Taking into account the increase in population, the number of surgeons will need to nearly triple to meet the VISION 2020 target by 2020.

OCOs/Nurses: Current & Projected Workforce per Million Population

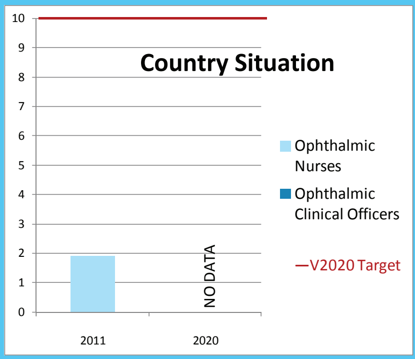

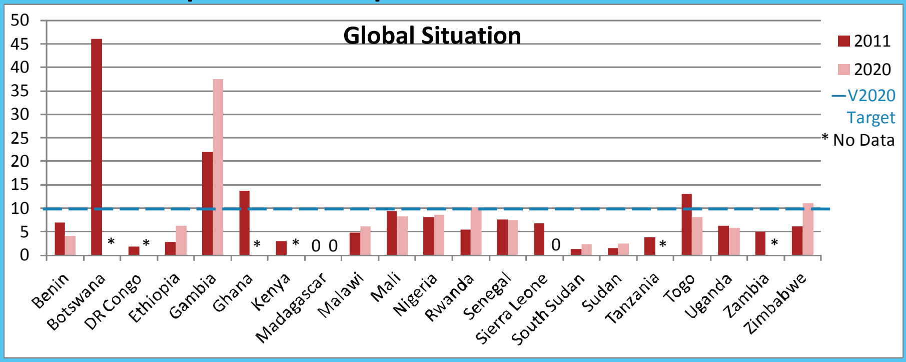

Practitioner Entry vs Exit: Over the past 3 years, 52 nurses have entered the workforce. However, there is no information on how many have exited the workforce, so projections for the situation in 2020 cannot be made.

Practitioner Working Location: The practitioner to population ratio for those working in the capital is 4.0 compared to 1.6 for those working outside the capital.

Practitioner vs Population Growth: Taking into account the expected 26% population increase between 2011 and 2020, there will need to be a 550% increase in practitioner numbers to meet the VISION 2020 target by 2020.

Refractionists: Current & Projected Workforce per Million Population

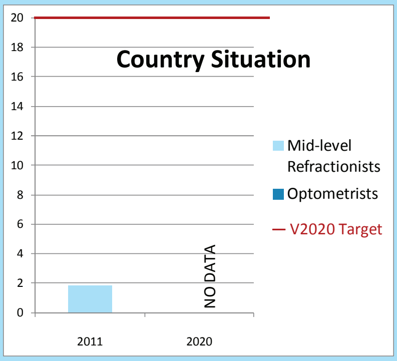

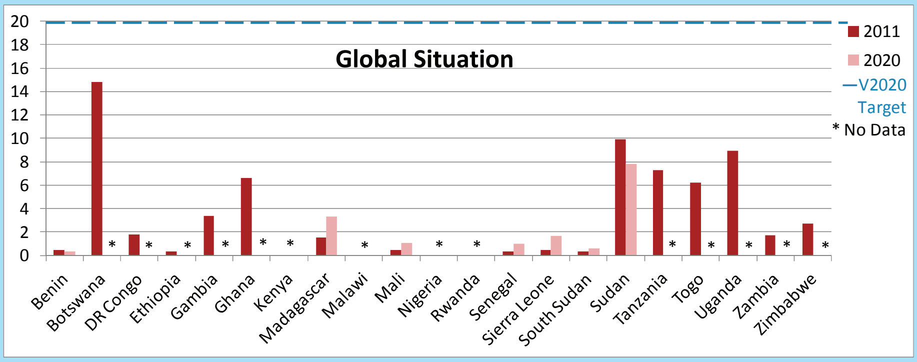

Practitioner Entry vs Exit: Over the past 3 years, 32 refractionists have entered the workforce. However, there is no information on how many have exited the workforce, so projections for the situation in 2020 cannot be made.

Practitioner Working Location: The 1 optometrist in DR Congo works outside the capital. Only 3 of the 120 mid-level refractionists are employed by the Government.

Practitioner vs Population Growth: DR Congo needs 14 times as many refractionists in order to meet the VISION 2020 target by 2020 and account for population growth.

Cataract Surgical Performance: Current & Projected Performance per Million Population

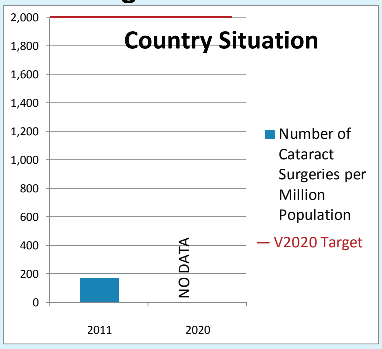

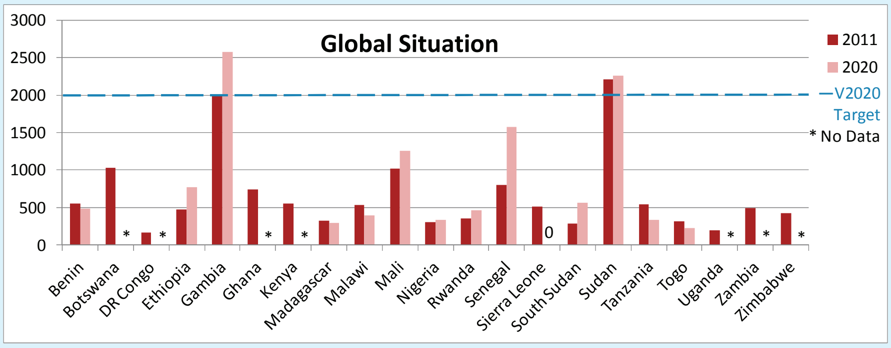

Taking into account population increase, DR Congo will need to carry out more than 15 times the 2011 annual number of cataract surgeries in order to meet this target by 2020.

Country Profile: Democratic Republic of the Congo

VISION 2020 Targets

Eye Care Cadre	# Practitioners per Million Population
Surgeons	4
OCOs/Nurses	10
Refractionists	20

Current Situation: 2011

Total Population	Population Living in Capital City	% Population Living in Capital	Population Over 50	% Population Over 50
67,663,883	8,798,000	13%	5,942,000	9%

Characteristics of the Active Eye Care Practitioner Workforce: 2011

Eye Care Cadre	Number of Practitioners in Active Workforce	Sector			Location		# Training Programmes available in Country
		Government	NGO/ Mission	Private for Profit	Capital	Outside Capital	
Ophthalmologists	67	20	27	20	54	13	1
Cataract Surgeons	56	12	21	23	19	37	1
**Surgeons**	**123**	**32**	**48**	**43**	**73**	**50**	**2**
Ophthalmic Clinical Officers	0	0	0	0	0	0	0
Ophthalmic Nurses	130	2	83	45	35	95	2
**OCOs/Nurses**	**130**	**2**	**83**	**45**	**35**	**95**	**2**
Optometrists	1	0	1	0	0	1	0
Mid-level Refractionists	120	3	112	5	23	97	0
**Refractionists**	**121**	**3**	**113**	**5**	**23**	**98**	**0**

Eye Care Practitioner Workforce Dynamics: 2011

Eye Care Cadre	Number of Practitioners in Active Workforce	Practitioners per Million Population			VISION 2020 Country Target # of Practitioners	Shortage in Practitioners to meet Target
		Countrywide	In Capital	Outside Capital		
Ophthalmologists	67	1.0	6.1	0.2		
Cataract Surgeons	56	0.8	2.2	0.6		
**Surgeons**	**123**	**1.8**	**8.3**	**0.8**	**271**	**148**
Ophthalmic Clinical Officers	0	0.0	0.0	0.0		
Ophthalmic Nurses	130	1.9	4.0	1.6		
**OCOs/Nurses**	**130**	**1.9**	**4.0**	**1.6**	**677**	**547**
Optometrists	1	0.0	0.0	0.0		
Mid-level Refractionists	120	1.8	2.6	1.6		
**Refractionists**	**121**	**1.8**	**2.6**	**1.7**	**1,353**	**1,232**

Annual Cataract Surgical Performance

Number of Cataract Surgeries Performed (data from 2010)	11,063
Number of Cataract Surgeries per Surgeon (surgical performance ratio)	90
% Surgeries Performed by Ophthalmologists (estimate)	65%
Number of Cataract Surgeries per Million Population (CSR)	163
Target Number of Cataract Surgeries to meet VISION 2020 Target	135,328
Shortage in Cataract Surgeries to meet VISION 2020 Target	124,265

Projected Situation: 2020

Projected Total Population	Projected Population Living in Capital City	% Projected Population Living in Capital	Projected Population Over 50	% Projected Population Over 50	Expected 9-year Population Increase	Expected 9-year Over 50 Population Increase
85,055,016	12,595,875	15%	7,649,000	9%	26%	29%

Projected Eye Care Practitioner Workforce Dynamics: 2020

Eye Care Cadre	Number of Practitioners in Active Workforce (2011)	Over last 3 years			Projected Net Change over next 9 years	Projected Number of Practitioners in Active Workforce	Projected Practitioners per Million Population			VISION 2020 Country Target # of Practitioners	Projected Shortage in Practitioners to meet VISION 2020
		Number Entered Workforce	Number Exited Workforce	Net Change in Practitioner Numbers			Countrywide	In Capital	Outside Capital		
Ophthalmologists	67	5	ND	ND	ND	ND	ND	ND	ND		
Cataract Surgeons	56	23	ND	ND	ND	ND	ND	ND	ND		
**Surgeons**	**123**	**28**	**ND**	**ND**	**ND**	**ND**	**ND**	**ND**	**ND**	**340**	**ND**
Ophthalmic Clinical Officers	0	0	0	0	0	0	0.0	0.0	0.0		
Ophthalmic Nurses	130	52	ND	ND	ND	ND	ND	ND	ND		
**OCOs/Nurses**	**130**	**52**	**ND**	**ND**	**ND**	**ND**	**ND**	**ND**	**ND**	**851**	**ND**
Optometrists	1	0	ND	ND	ND	ND	ND	ND	ND		
Mid-level Refractionists	120	32	ND	ND	ND	ND	ND	ND	ND		
**Refractionists**	**121**	**32**	**ND**	**ND**	**ND**	**ND**	**ND**	**ND**	**ND**	**1,701**	**ND**

Annual Projected Cataract Surgical Performance: 2020

Eye Care Cadre	% Surgeries Performed (estimate)	Surgical Performance Ratio per Cadre (2011)	Projected Number of Surgeons in Active Workforce	Projected Number of Cataract Surgeries Performed	Projected Number of Cataract Surgeries per Million Population (CSR)	Target Number of Cataract Surgeries to meet VISION 2020 Target	Projected Shortage in Cataract Surgeries to meet VISION 2020 Target
Ophthalmologists	65%	107	ND	ND			
Cataract Surgeons	35%	69	ND	ND			
**Surgeons**	**100%**	**90**	**ND**	**ND**	**ND**	**170,110**	**ND**

ND: No Data

Country Profile: Ethiopia

Key Messages

- **Surgeons:** Ethiopia is currently half-way to meeting this VISION 2020 target. At the current increasing practitioner growth rate they are projected to meet it by 2034.
- **OCOs/Nurses:** Ethiopia has a rapidly growing number of ophthalmic nurses. Although the country is currently less than a third of the way to this target, they are set to meet it by 2034.
- **Refractionists:** With only 29 Refractionists in the country, Ethiopia is less than 2% of the way to meeting this target and will need to recruit a further than 2,000 Refractionists by 2020
- **Cataract Surgeries:** The number of cataract surgeries is increasing faster than population growth but, to meet the VISION 2020 target, these will need to more than double.

VISION 2020 Targets

	Eye Care Practitioners per Million Population			Cataract Surgeries Performed per Million Population	Cataract Surgeries Performed per Surgeon
	Surgeons*	Ophthalmic Clinical Officers /Nurses	Refrac-tionists		
VISION2020 Target	4	10	20	2,000	500
2011 Situation	1.9	2.8	0.3	473	255
On Track					

* For the Africa region this includes Ophthalmologists and Cataract Surgeons

Eye Care Practitioners: % Working Inside/Outside Capital

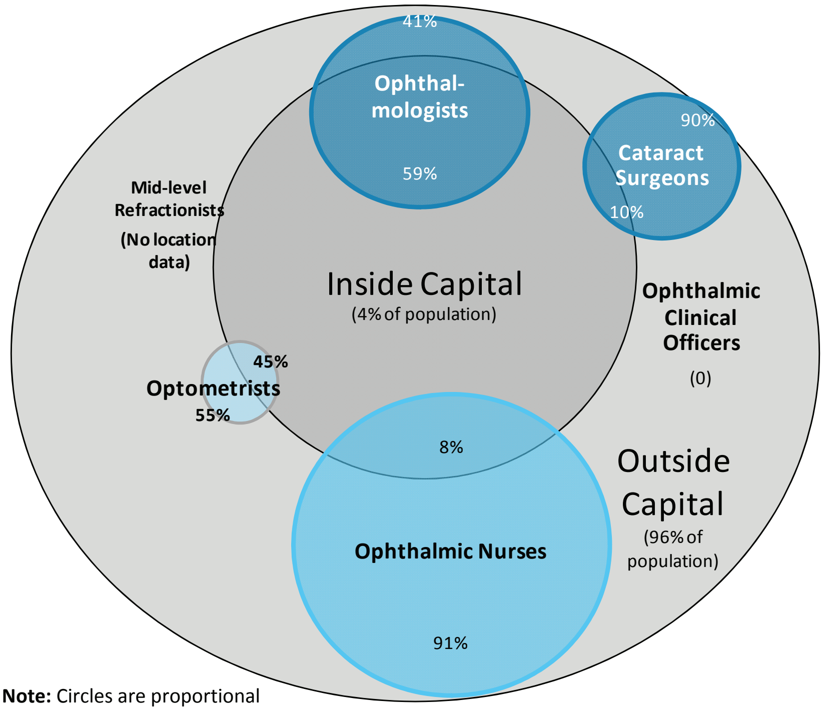

Note: Circles are proportional to numbers of eye care practitioners

In-Country Training Programmes

Surgeons	OCOs/Nurses	Refractionists
Total: 6	Total: 5	Total: 2

Distribution of Optometrists

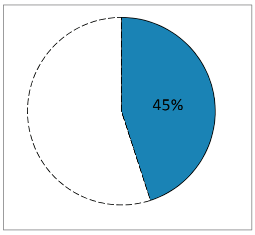

Optometrists based in the capital

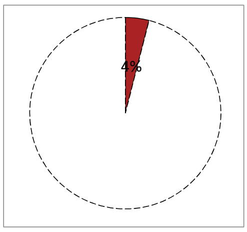

Population living in the capital

45% of Optometrists treat 4% of the population

Eye Care Practitioners: Split between Sectors

Government NGO/Mission Private for Profit

Surgeons

Ophthalmologists

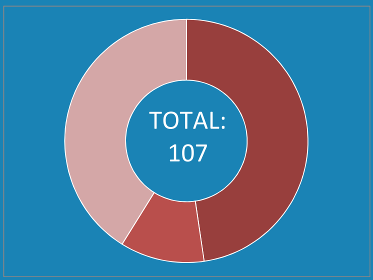

Cataract Surgeons

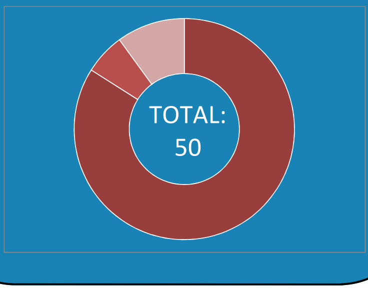

OCOs/Nurses

Ophthalmic Clinical Officers

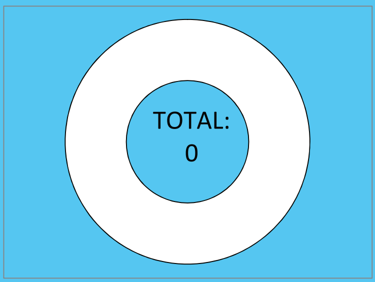

Ophthalmic Nurses

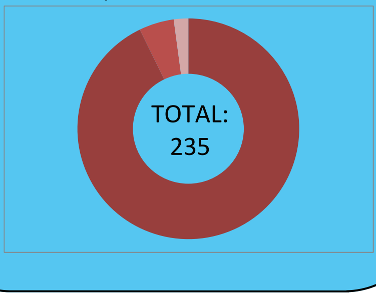

Refractionists

Optometrists

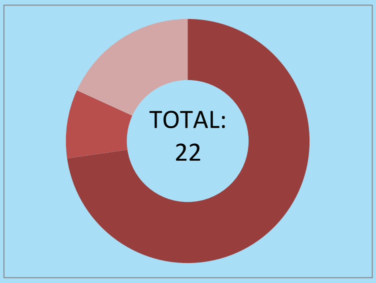

Mid-level Refractionists

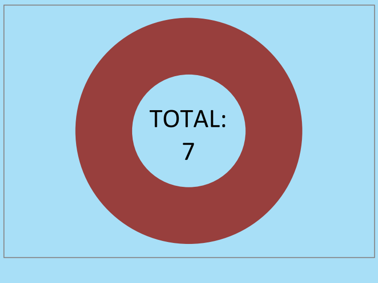

Surgeons: Current & Projected Workforce per Million Population

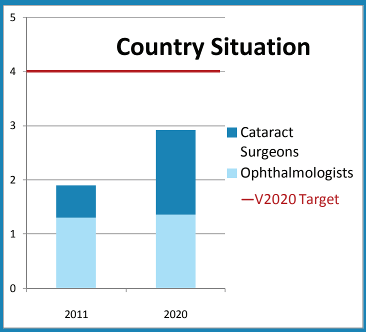

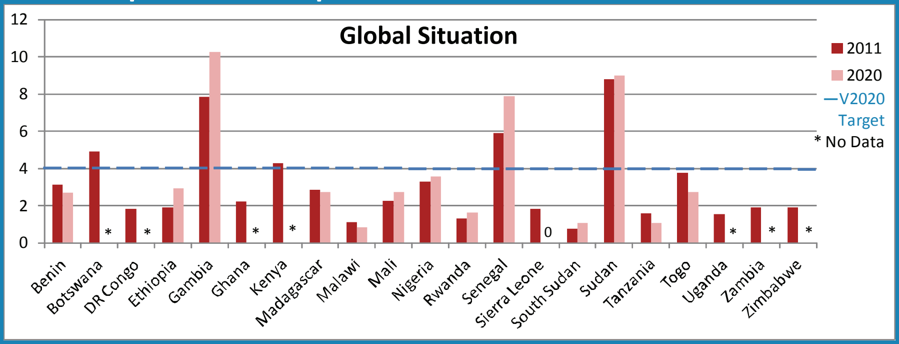

Practitioner Entry vs Exit: Taking into account both surgeon and general population growth rates, Ethiopia is projected to meet this VISION 2020 target by 2034. The number of cataract surgeons is increasing faster than the number of ophthalmologists.

Practitioner Working Location: With a ratio of 1.9, Ethiopia is half-way to meeting the VISION 2020 target for practitioner to population ratio. The ratio for those working in the capital is 21.8 and 1.1 for those working outside the capital.

Practitioner vs Population Growth: The overall surgeon practitioner growth rate of 88% is above the general population growth rate of 19%, although a growth rate of 157% is needed to meet the VISION 2020 target by 2020.

OCOs/Nurses: Current & Projected Workforce per Million Population

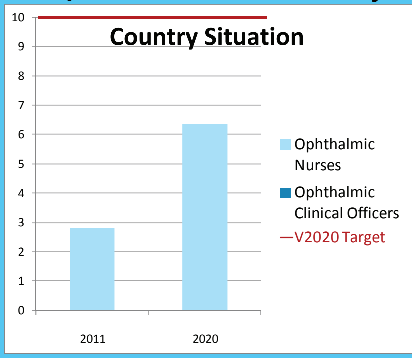

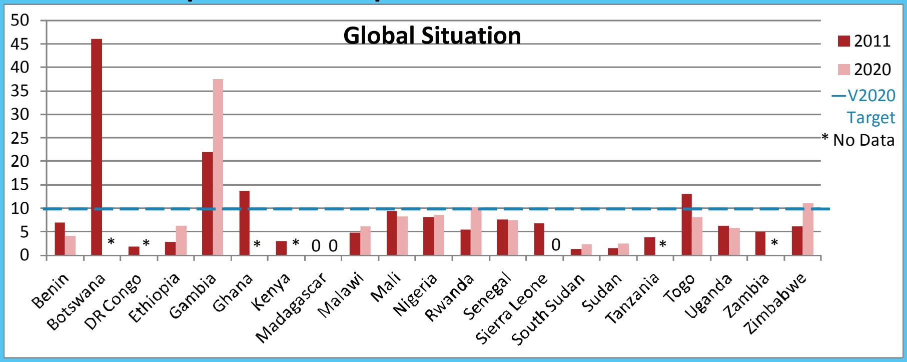

Practitioner Entry vs Exit: Taking into account both ophthalmic nurse and general population growth rates, Ethiopia is projected to meet this VISION 2020 target by 2034.

Practitioner Working Location: The practitioner to population ratio for those working in the capital is 6.4 compared to 2.6 for those working outside the capital. This is projected to change to 12.7 and 6.0 respectively by 2020.

Practitioner vs Population Growth: The number of practitioners needs to increase 50% faster than it is currently projected to, in order to meet the target by 2020.

Refractionists: Current & Projected Workforce per Million Population

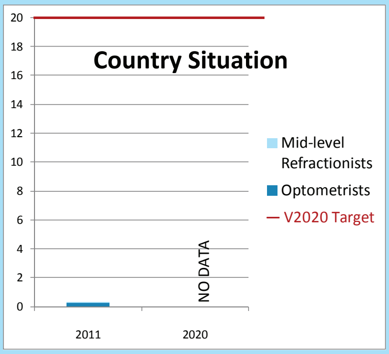

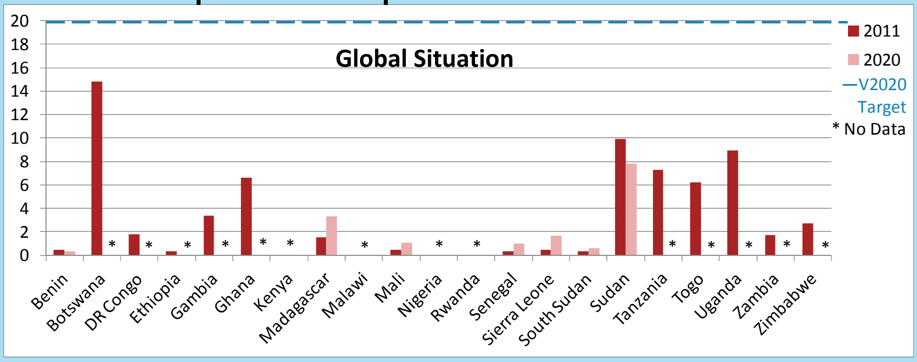

Practitioner Entry vs Exit: There is a projected growth in the number of ophthalmologists, but no data is available on the entry of mid-level refractionists in the workforce.

Practitioner Working Location: The optometrist to population ratio for those working in the capital is 3.4 compared to 0.1 for those working outside the capital. There is no location data for mid-level refractionists.

Practitioner vs Population Growth: There needs to be a 7,000% increase in the number of refractionists by 2020 to meet this target, whilst accounting for the 19% growth in population over this period.

Cataract Surgical Performance: Current & Projected Performance per Million Population

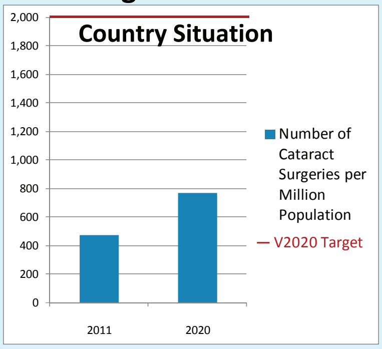

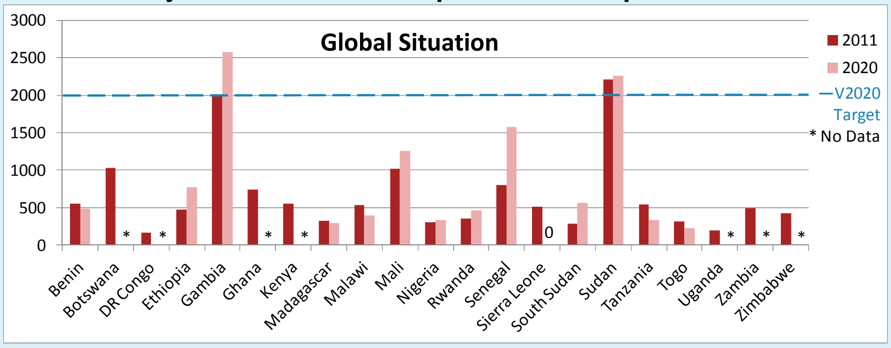

Although Ethiopia has a projected 62% increase in cataract surgeries by 2020, this is still below the required 323% to reach VISION 2020 target by 2020.

Country Profile: Ethiopia

Current Situation: 2011

Total Population	Population Living in Capital City	% Population Living in Capital	Population Over 50	% Population Over 50
84,603,586	2,979,000	4%	8,859,000	10%

VISION 2020 Targets

Eye Care Cadre	# Practitioners per Million Population
Surgeons	4
OCOs/Nurses	10
Refractionists	20

Characteristics of the Active Eye Care Practitioner Workforce: 2011

Eye Care Cadre	Number of Practitioners in Active Workforce	Sector			Location		# Training Programmes available in Country
		Government	NGO/ Mission	Private for Profit	Capital	Outside Capital	
Ophthalmologists *	107	51	12	44	60	42	3
Cataract Surgeons	50	42	3	5	5	45	3
**Surgeons**	**157**	**93**	**15**	**49**	**65**	**87**	**6**
Ophthalmic Clinical Officers	0	0	0	0	0	0	0
Ophthalmic Nurses *	235	218	12	5	19	214	5
**OCOs/Nurses**	**235**	**218**	**12**	**5**	**19**	**214**	**5**
Optometrists	22	16	2	4	10	12	2
Mid-level Refractionists	7	7	0	0	ND	ND	0
**Refractionists**	**29**	**23**	**2**	**4**	**ND**	**ND**	**2**

Eye Care Practitioner Workforce Dynamics: 2011

Eye Care Cadre	Number of Practitioners in Active Workforce	Practitioners per Million Population			VISIOn 2020 Country Target # of Practitioners	Shortage in Practitioners to meet Target
		Countrywide	In Capital	Outside Capital		
Ophthalmologists	107	1.3	20.1	0.5		
Cataract Surgeons	50	0.6	1.7	0.6		
**Surgeons**	**157**	**1.9**	**21.8**	**1.1**	**338**	**181**
Ophthalmic Clinical Officers	0	0.0	0.0	0.0		
Ophthalmic Nurses	235	2.8	6.4	2.6		
**OCOs/Nurses**	**235**	**2.8**	**6.4**	**2.6**	**846**	**611**
Optometrists	22	0.3	3.4	0.1		
Mid-level Refractionists	7	0.1	ND	ND		
**Refractionists**	**29**	**0.3**	**ND**	**ND**	**1,692**	**1,663**

Annual Cataract Surgical Performance

Number of Cataract Surgeries Performed (data from 2010)	40,000
Number of Cataract Surgeries per Surgeon (surgical performance ratio)	255
% Surgeries Performed by Ophthalmologists (estimate)	65%
Number of Cataract Surgeries per Million Population (CSR)	473
Target Number of Cataract Surgeries to meet VISION 2020 Target	169,207
Shortage in Cataract Surgeries to meet VISION 2020 Target	129,207

* Location data for 5 NGO Ophthalmologists and 2 NGO Ophthalmic Nurses is missing, but as this represents less than 5% of active workforce it has been used in the analysis

ND: No Data

Projected Situation: 2020

Projected Total Population	Projected Population Living in Capital City	% Projected Population Living in Capital	Projected Population Over 50	% Projected Population Over 50	Expected 9-year Population Increase	Expected 9-year Over 50 Population Increase
101,055,936	4,084,852	4%	11,478,000	11%	19%	30%

Projected Eye Care Practitioner Workforce Dynamics: 2020

Eye Care Cadre	Number of Practitioners in Active Workforce (2011)	Over last 3 years			Projected Net Change over next 9 years	Projected Number of Practitioners in Active Workforce	Projected Practitioners per Million Population			VISION 2020 Country Target # of Practitioners	Projected Shortage in Practitioners to meet VISION 2020
		Number Entered Workforce	Number Exited Workforce	Net Change in Practitioner Numbers			Countrywide	In Capital	Outside Capital		
Ophthalmologists	107	20	10	10	30	137	1.4	18.8	0.6		
Cataract Surgeons	50	36	0	36	108	158	1.6	3.9	1.5		
**Surgeons**	**157**	**56**	**10**	**46**	**138**	**295**	**2.9**	**29.9**	**1.7**	**404**	**109**
Ophthalmic Clinical Officers	0	0	0	0	0	0	0.0	0.0	0.0		
Ophthalmic Nurses	235	138	2	136	408	643	6.4	12.7	6.0		
**OCOs/Nurses**	**235**	**138**	**2**	**136**	**408**	**643**	**6.4**	**12.7**	**6.0**	**1,011**	**368**
Optometrists	22	16	1	15	45	67	0.7	7.5	0.4		
Mid-level Refractionists	7	ND	0	ND	ND	ND	ND	ND	ND		
**Refractionists**	**29**	**ND**	**1**	**ND**	**ND**	**ND**	**ND**	**ND**	**ND**	**2,021**	**ND**

Annual Projected Cataract Surgical Performance: 2020

Eye Care Cadre	% Surgeries Performed (estimate)	Surgical Performance Ratio per Cadre (2011)	Projected Number of Surgeons in Active Workforce	Projected Number of Cataract Surgeries Performed	Projected Number of Cataract Surgeries per Million Population (CSR)	Target Number of Cataract Surgeries to meet VISION 2020 Target	Projected Shortage in Cataract Surgeries to meet VISION 2020 Target
Ophthalmologists	65%	243	137	33,290			
Cataract Surgeons	35%	280	158	44,240			
**Surgeons**	**100%**	**255**	**295**	**77,530**	**767**	**202,112**	**124,582**

ND: No Data

Country Profile: Gambia

Key Messages

- **Surgeons:** Gambia is well above this target, which is projected to increase further by 2020. This is due almost entirely to the presence of cataract surgeons.
- **OCOs/Nurses:** Gambia is also well above this target, also projected to increase further by 2020. This is due to ophthalmic nurses, there are no ophthalmic clinical officers in Gambia.
- **Refractionists:** Gambia is less than a quarter of the way to meeting this VISION 2020 target. To meet this target by 2020, 40 refractionists need to be recruited.
- **Cataract Surgeries:** Cataract surgeries are increasing relative to population growth, meaning that whilst Gambia currently meets this target, they will have surpassed it by 2020.

VISION 2020 Targets

	Eye Care Practitioners per Million Population			Cataract Surgeries Performed per Million Population	Cataract Surgeries Performed per Surgeon
	Surgeons*	Ophthalmic Clinical Officers /Nurses	Refrac- tionists		
VISION2020 Target	4	10	20	2,000	500
2011 Situation	7.9	22.0	3.4	1,993	253
On Track					

* For the Africa region this includes Ophthalmologists and Cataract Surgeons

Eye Care Practitioners: % Working Inside/Outside Capital

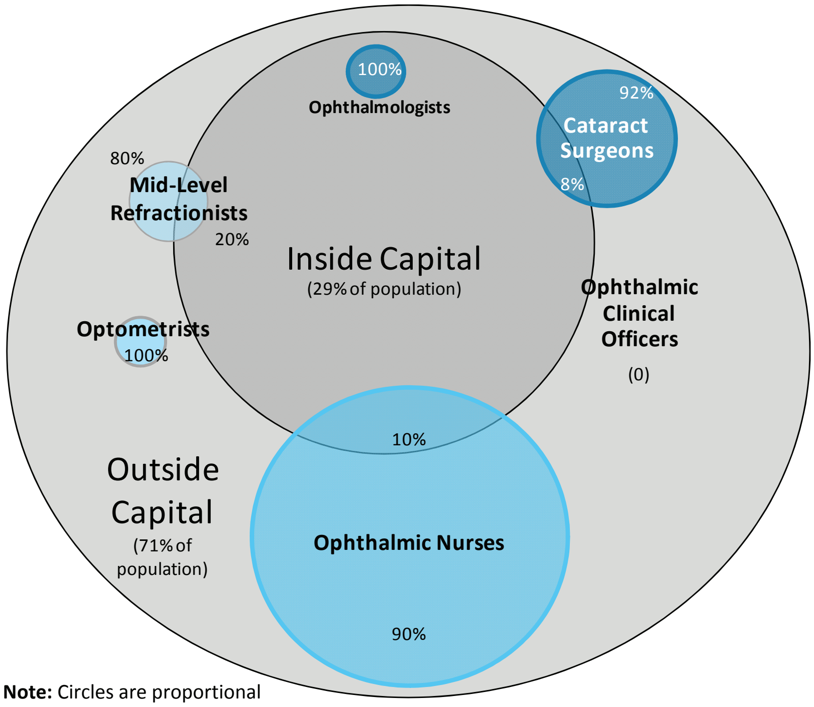

Note: Circles are proportional to numbers of eye care practitioners

In-Country Training Programmes

Surgeons	OCOs/Nurses	Refractionists
Total: 1	Total: 2	Total: 2

Distribution of Ophthalmic Nurses

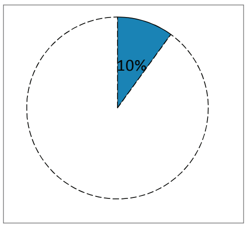

Ophthalmic Nurses based in the capital

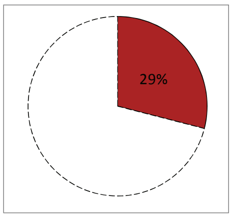

Population living in the capital

10% of Ophthalmic Nurses treat 29% of the population

Eye Care Practitioners: Split between Sectors

Government NGO/Mission Private for Profit

Surgeons

Ophthalmologists

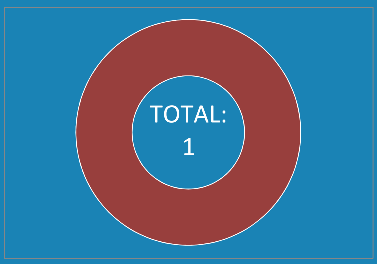

Cataract Surgeons

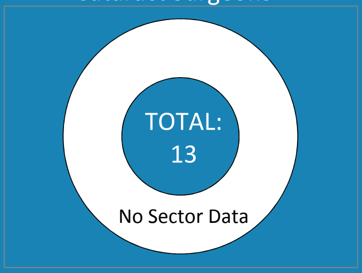

OCOs/Nurses

Ophthalmic Clinical Officers

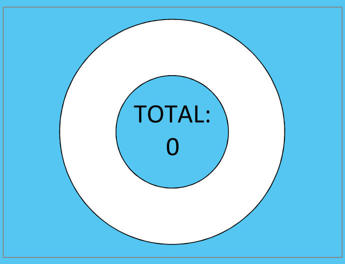

Ophthalmic Nurses

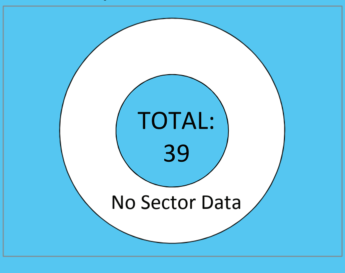

Refractionists

Optometrists

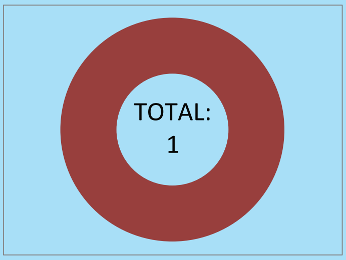

Mid-level Refractionists

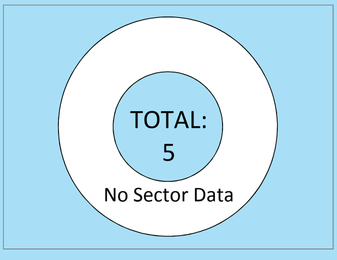

Surgeons: Current & Projected Workforce per Million Population

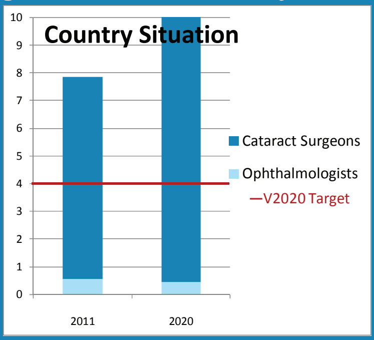

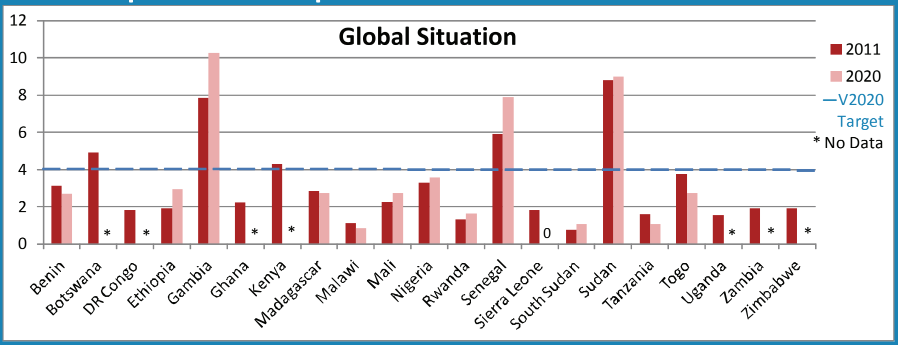

Practitioner Entry vs Exit: The number of cataract surgeons is expected to grow in the coming years, whilst the number of ophthalmologists is projected to remain the same. Gambia will still be above target by 2020.

Practitioner Working Location: The ratio of surgeons to population is 4.0 for those working in the capital and 9.5 for those based outside the capital.

Practitioner vs Population Growth: Surgeon numbers are projected to grow 2.5 times faster than the population between 2011 and 2020.

OCOs/Nurses: Current & Projected Workforce per Million Population

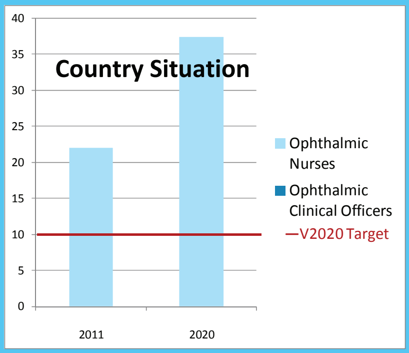

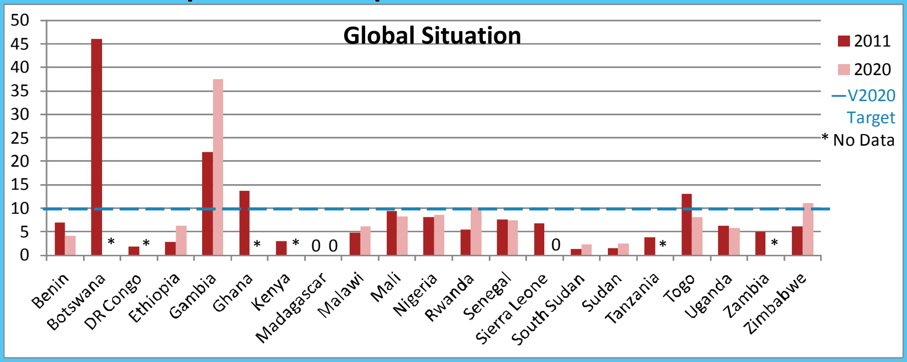

Practitioner Entry vs Exit: The number of ophthalmic nurses is currently growing. By 2020 Gambia is projected to have nearly four times more nurses as required by V2020 target.

Practitioner Working Location: 90% of ophthalmic nurses work outside the capital. The practitioner to population ratios are 7.9 and 27.6 respectively for those working inside and outside the capital, meaning that Gambia is below target for those based in the capital.

Practitioner vs Population Growth: The ophthalmic nurse population is projected to grow faster than the general population between 2011 and 2020.

Refractionists: Current & Projected Workforce per Million Population

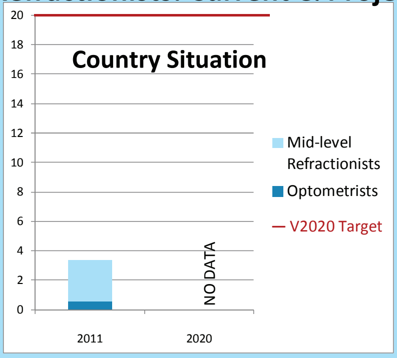

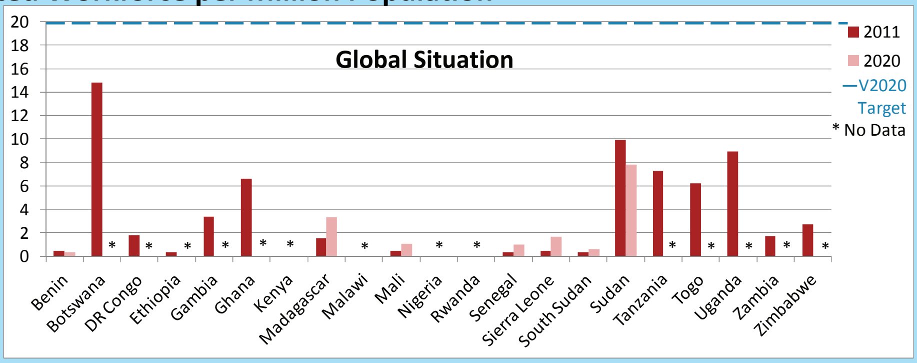

Practitioner Entry vs Exit: Gambia has 1 optometrist currently working and over the past 3 years there has been no entry or exit from the workforce. There is no exit data for the 5 mid-level refractionists.

Practitioner Working Location: 83% of refractionists work inside the capital where 31% of the population live.

Practitioner vs Population Growth: Gambia needs 7 times as many refractionists in order to meet the VISION 2020 target by 2020, whilst accounting for the 26% growth in population over this period.

Cataract Surgical Performance: Current & Projected Performance per Million Population

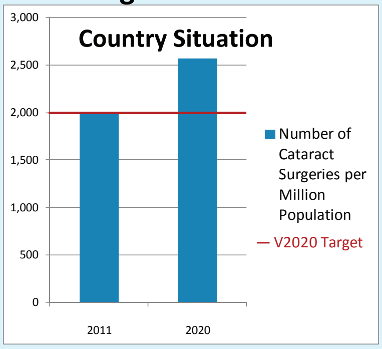

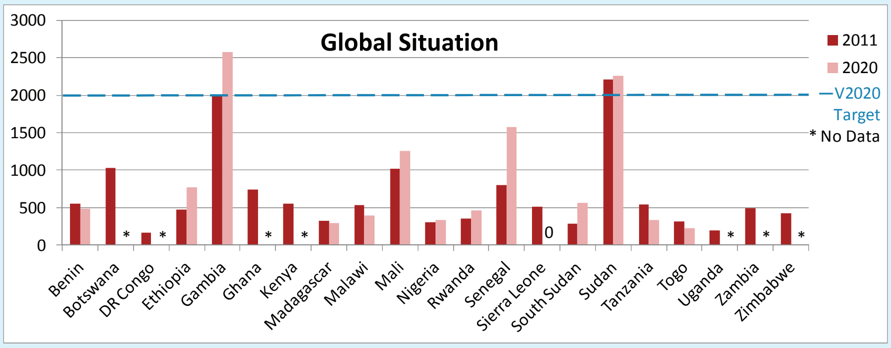

Gambia had almost met the VISION 2020 target in 2011 and if the increasing number of surgeons continues, then the target will be met by 2020.

Country Profile: Gambia

Current Situation: 2011				
Total Population	Population Living in Capital City	% Population Living in Capital	Population Over 50	% Population Over 50
1,774,028	506,000	29%	139,000	8%

VISION 2020 Targets	
Eye Care Cadre	# Practitioners per Million Population
Surgeons	4
OCOs/Nurses	10
Refractionists	20

Characteristics of the Active Eye Care Practitioner Workforce: 2011

Eye Care Cadre	Number of Practitioners in Active Workforce	Sector			Location		# Training Programmes available in Country
		Government	NGO/ Mission	Private for Profit	Capital	Outside Capital	
Ophthalmologists	1	1	0	0	1	0	0
Cataract Surgeons	13	ND	ND	ND	1	12	1
**Surgeons**	**14**	**ND**	**ND**	**ND**	**2**	**12**	**1**
Ophthalmic Clinical Officers	0	0	0	0	0	0	0
Ophthalmic Nurses	39	ND	ND	ND	4	35	2
**OCOs/Nurses**	**39**	**ND**	**ND**	**ND**	**4**	**35**	**2**
Optometrists	1	1	0	0	0	1	0
Mid-level Refractionists	5	ND	ND	ND	1	4	2
**Refractionists**	**6**	**ND**	**ND**	**ND**	**1**	**5**	**2**

Eye Care Practitioner Workforce Dynamics: 2011

Eye Care Cadre	Number of Practitioners in Active Workforce	Practitioners per Million Population			VISION 2020 Country Target # of Practitioners	Shortage in Practitioners to meet Target
		Countrywide	In Capital	Outside Capital		
Ophthalmologists	1	0.6	2.0	0.0		
Cataract Surgeons	13	7.3	2.0	9.5		
**Surgeons**	**14**	**7.9**	**4.0**	**9.5**	**7**	**Target Met**
Ophthalmic Clinical Officers	0	0.0	0.0	0.0		
Ophthalmic Nurses	39	22.0	7.9	27.6		
**OCOs/Nurses**	**39**	**22.0**	**7.9**	**27.6**	**18**	**Target Met**
Optometrists	1	0.6	0.0	0.8		
Mid-level Refractionists	5	2.8	2.0	3.2		
**Refractionists**	**6**	**3.4**	**2.0**	**3.9**	**35**	**29**

Annual Cataract Surgical Performance

Number of Cataract Surgeries Performed (data from 2010)	3,536
Number of Cataract Surgeries per Surgeon (surgical performance ratio)	253
% Surgeries Performed by Ophthalmologists (estimate)	9%
Number of Cataract Surgeries per Million Population (CSR)	1,993
Target Number of Cataract Surgeries to meet VISION 2020 Target	3,548
Shortage in Cataract Surgeries to meet VISION 2020 Target	12

ND: No Data

Projected Situation: 2020

Projected Total Population	Projected Population Living in Capital City	% Projected Population Living in Capital	Projected Population Over 50	% Projected Population Over 50	Expected 9-year Population Increase	Expected 9-year Over 50 Population Increase
2,242,952	684,767	31%	191,000	9%	26%	37%

Projected Eye Care Practitioner Workforce Dynamics: 2020

Eye Care Cadre	Number of Practitioners in Active Workforce (2011)	Over last 3 years			Projected Net Change over next 9 years	Projected Number of Practitioners in Active Workforce	Projected Practitioners per Million Population			VISION 2020 Country Target # of Practitioners	Projected Shortage in Practitioners to meet VISION 2020
		Number Entered Workforce	Number Exited Workforce	Net Change in Practitioner Numbers			Countrywide	In Capital	Outside Capital		
Ophthalmologists	1	0	0	0	0	1	0.5	1.5	0.0		
Cataract Surgeons	13	4	1	3	9	22	9.8	2.5	13.0		
**Surgeons**	**14**	**4**	**1**	**3**	**9**	**23**	**10.3**	**4.8**	**12.7**	**9**	**Target Met**
Ophthalmic Clinical Officers	0	0	0	0	0	0	0.0	0.0	0.0		
Ophthalmic Nurses	39	17	2	15	45	84	37.5	12.6	48.4		
**OCOs/Nurses**	**39**	**17**	**2**	**15**	**45**	**84**	**37.5**	**12.6**	**48.4**	**22**	**Target Met**
Optometrists	1	0	0	0	0	1	0.5	0.0	0.6		
Mid-level Refractionists	5	3	ND	ND	ND	ND	ND	ND	ND		
**Refractionists**	**6**	**3**	**ND**	**ND**	**ND**	**ND**	**ND**	**ND**	**ND**	**45**	**ND**

Annual Projected Cataract Surgical Performance: 2020

Eye Care Cadre	% Surgeries Performed (estimate)	Surgical Performance Ratio per Cadre (2011)	Projected Number of Surgeons in Active Workforce	Projected Number of Cataract Surgeries Performed	Projected Number of Cataract Surgeries per Million Population (CSR)	Target Number of Cataract Surgeries to meet VISION 2020 Target	Projected Shortage in Cataract Surgeries to meet VISION 2020 Target
Ophthalmologists	9%	318	1	318			
Cataract Surgeons	91%	248	22	5,445			
**Surgeons**	**100%**	**253**	**23**	**5,764**	**2,570**	**4,486**	**Target Met**

ND: No Data

Country Profile: Ghana

Key Messages

- **Surgeons:** Ghana is more than half-way to meeting the VISION 2020 target and will need to recruit 66 surgeons to meet it.
- **OCOs/Nurses:** Ghana has already exceeded this VISION 2020 target, due entirely to the presence of ophthalmic nurses.
- **Refractionists:** Ghana is a third of the way to meeting this target. The current growth rate of optometrists looks promising, lack of data on mid-level refractionists means that it is not clear whether this target will be met by 2020.
- **Cataract Surgeries:** Ghana is a third of the way to meeting this target. An additional 13,000 surgeries, representing a 70% increase, need to be carried out annually in order to meet this target by 2020.

VISION 2020 Targets

	Eye Care Practitioners per Million Population			Cataract Surgeries Performed per Million Population	Cataract Surgeries Performed per Surgeon
	Surgeons*	Ophthalmic Clinical Officers /Nurses	Refrac-tionists		
VISION2020 Target	4	10	20	2,000	500
2011 Situation	2.2	13.7	6.6	738	335
On Track					

* For the Africa region this includes Ophthalmologists and Cataract Surgeons

Eye Care Practitioners: % Working Inside/Outside Capital

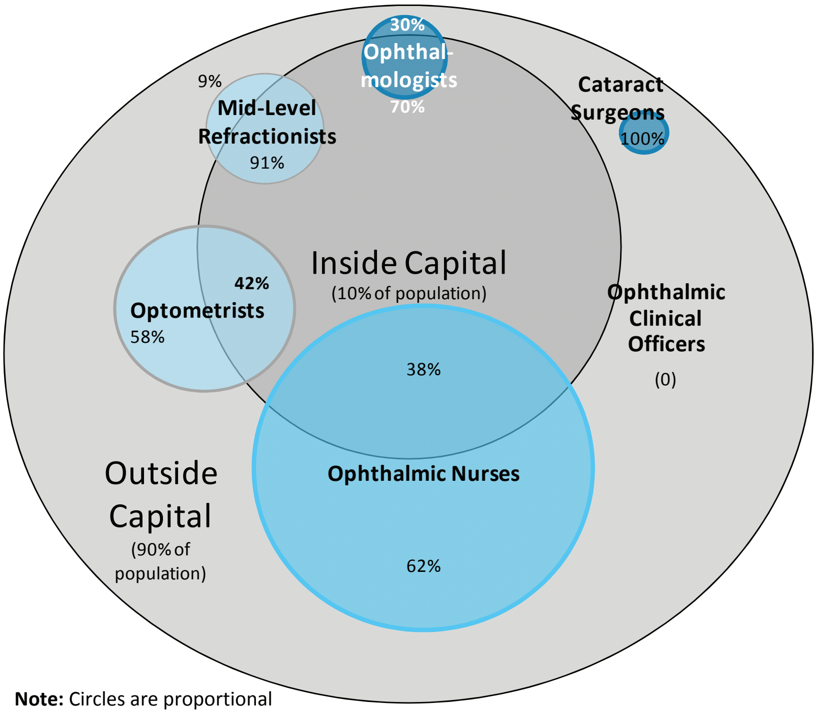

Note: Circles are proportional to numbers of eye care practitioners

In-Country Training Programmes

Surgeons	OCOs/Nurses	Refractionists
Total: 2	Total: 2	Total: 3

Distribution of Ophthalmologists

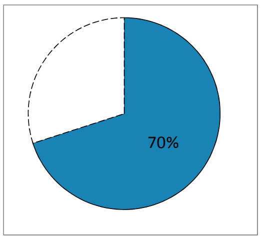

Ophthalmologists based in the capital

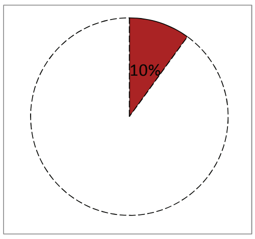

Population living in the capital

70% of Ophthalmologists treat 10% of the population

Data below does not include private sector for all but mid-level refractionists

Eye Care Practitioners: Split between Sectors

Government | NGO/Mission | Private for Profit

Surgeons

Ophthalmologists

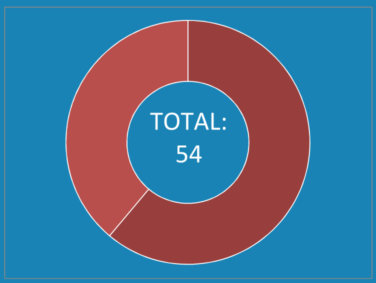

Cataract Surgeons

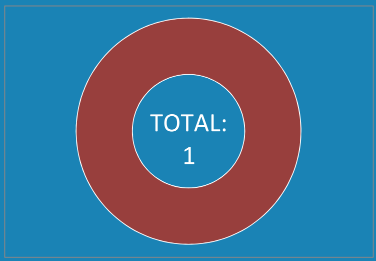

OCOs/Nurses

Ophthalmic Clinical Officers

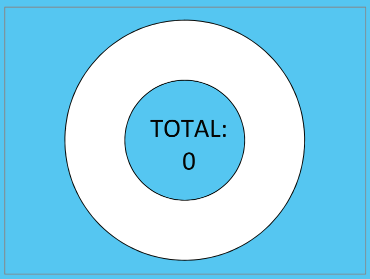

Ophthalmic Nurses

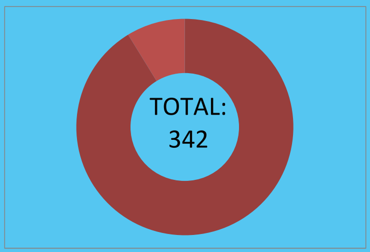

Refractionists

Optometrists

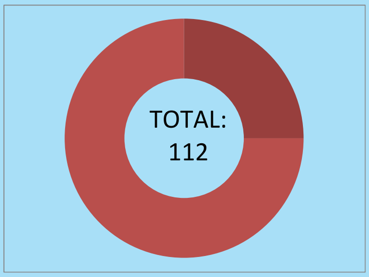

Mid-level Refractionists

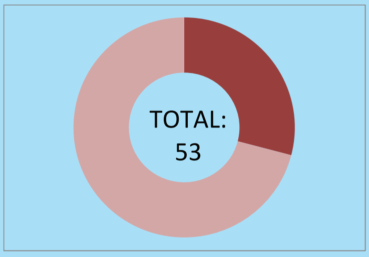

Surgeons: Current & Projected Workforce per Million Population

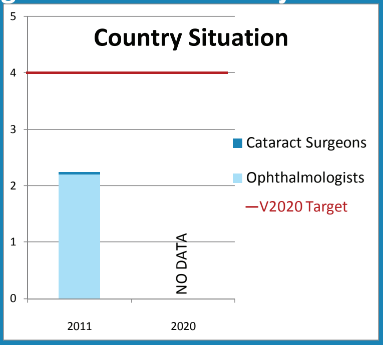

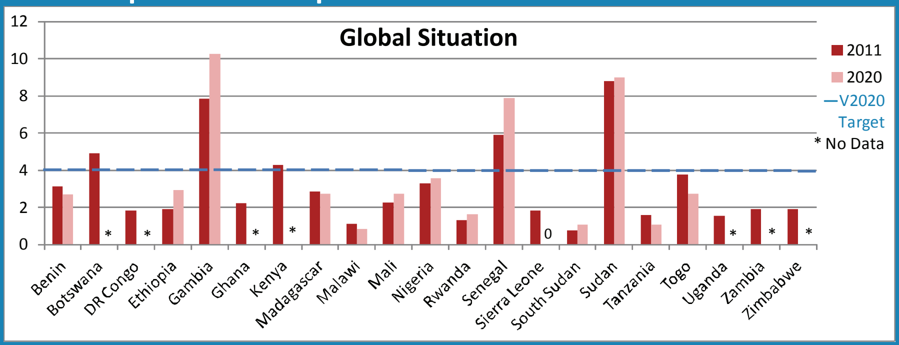

Practitioner Entry vs Exit: Over the past 3 years, 3 ophthalmologists and no cataract surgeons have entered the workforce. However, there is no information on how many have exited the workforce, so projections for the situation in 2020 cannot be made.

Practitioner Working Location: The overall practitioner to population ratio of 2.2, becomes 14.8 for those working in the capital compared with 0.8 for those working outside the capital.

Practitioner vs Population Growth: The number of surgeons will need to more than double to meet the VISION 2020 target by 2020, taking into account the expected 22% population increase over this period.

OCOs/Nurses: Current & Projected Workforce per Million Population

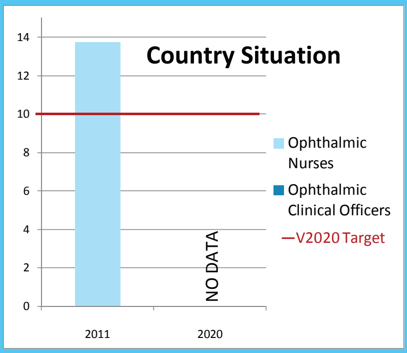

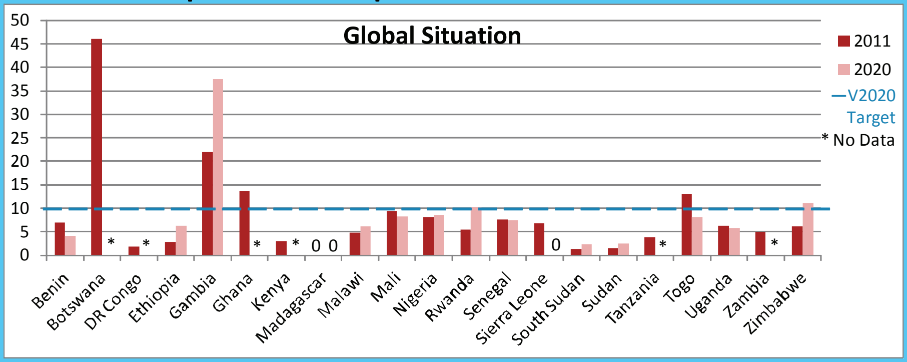

Practitioner Entry vs Exit: Over the past 3 years, 90 ophthalmic nurses have entered the workforce. However, there is no information on how many have exited the workforce, so projections for the situation in 2020 cannot be made.

Practitioner Working Location: With a ratio of 13.7, Ghana currently exceeds the VISION 2020 target for practitioner to population ratio. The ratio for those working in the capital is 50.5 and 9.5 for those working outside the capital.

Practitioner vs Population Growth: So long as no ophthalmic nurses exit the workforce, Ghana is set to meet this target by 2020.

Refractionists: Current & Projected Workforce per Million Population

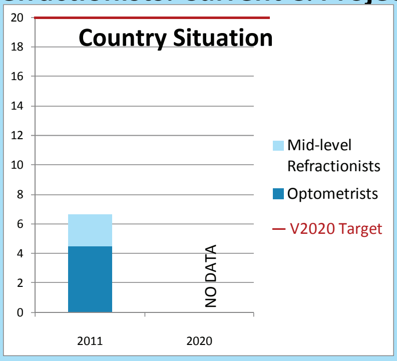

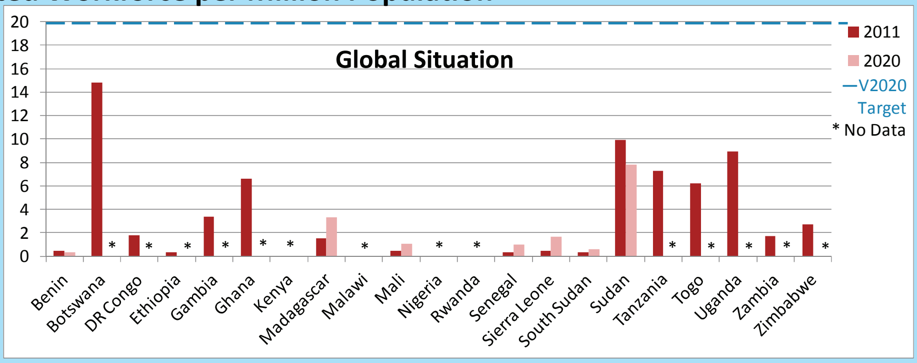

Practitioner Entry vs Exit: Nearly 50 optometrists join the workforce each year and only 2 have left over the past 3 years. However, there is no information on mid-level refractionists.

Practitioner Working Location: The refractionist to population ratio is 6.6 for the overall population and 36.9 and 3.1 respectively for those working inside and outside the capital.

Practitioner vs Population Growth: Projected growth data is only available for optometrists, not mid-level refractionists. Nearly 50 optometrists join the workforce each year, meaning the number of mid-level refractionists will need to increase 7-fold to meet the target by 2020.

Cataract Surgical Performance: Current & Projected Performance per Million Population

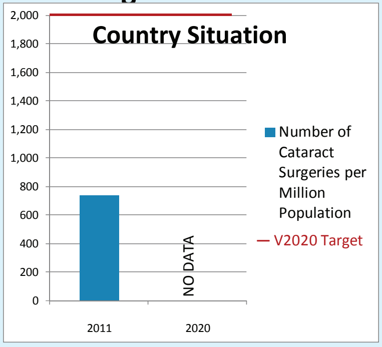

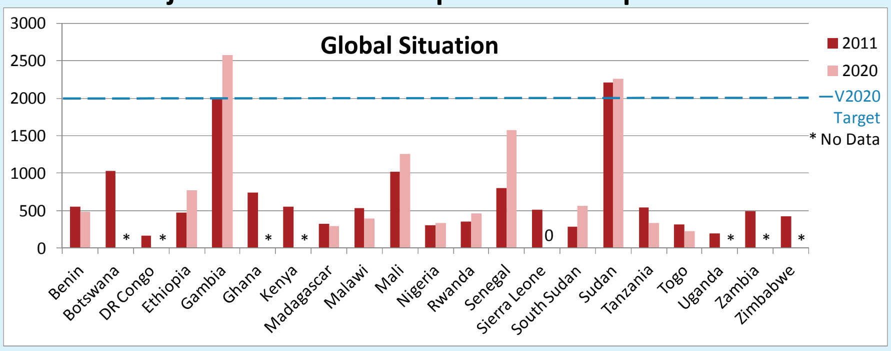

Ghana will need to perform more than three times as many annual cataract surgeries by 2020 in order to meet the VISION 2020 target, taking into account expected population growth.

Country Profile: Ghana

Current Situation: 2011

Total Population	Population Living in Capital City	% Population Living in Capital	Population Over 50	% Population Over 50
24,928,111	2,573,000	10%	2,714,000	11%

VISION 2020 Targets

Eye Care Cadre	# Practitioners per Million Population
Surgeons	4
OCOs/Nurses	10
Refractionists	20

Characteristics of the Active Eye Care Practitioner Workforce: 2011

Eye Care Cadre	Number of Practitioners in Active Workforce	Sector			Location		# Training Programmes available in Country
		Government	NGO/ Mission	Private for Profit*	Capital	Outside Capital	
Ophthalmologists	54	33	21	ND	38	16	2
Cataract Surgeons	1	0	1	ND	0	1	0
**Surgeons**	**55**	**33**	**22**	**ND**	**38**	**17**	**2**
Ophthalmic Clinical Officers	0	0	0	0	0	0	0
Ophthalmic Nurses	342	312	30	ND	130	212	2
**OCOs/Nurses**	**342**	**312**	**30**	**ND**	**130**	**212**	**2**
Optometrists	112	28	84	ND	47	65	2
Mid-level Refractionists	53	18	ND	44	48	5	1
**Refractionists**	**165**	**46**	**ND**	**ND**	**95**	**70**	**3**

Eye Care Practitioner Workforce Dynamics: 2011

Eye Care Cadre	Number of Practitioners in Active Workforce	Practitioners per Million Population			VISION 2020 Country Target # of Practitioners	Shortage in Practitioners to meet Target
		Countrywide	In Capital	Outside Capital		
Ophthalmologists	54	2.2	14.8	0.7		
Cataract Surgeons	1	0.04	0.0	0.0		
**Surgeons**	**55**	**2.2**	**14.8**	**0.8**	**100**	**45**
Ophthalmic Clinical Officers	0	0	0.0	0.0		
Ophthalmic Nurses	342	13.7	50.5	9.5		
**OCOs/Nurses**	**342**	**13.7**	**50.5**	**9.5**	**249**	**Target Met**
Optometrists	112	4.5	18.3	2.9		
Mid-level Refractionists	53	2.1	18.7	0.2		
**Refractionists**	**165**	**6.6**	**36.9**	**3.1**	**499**	**334**

Annual Cataract Surgical Performance

Number of Cataract Surgeries Performed (data from 2010)	18,405
Number of Cataract Surgeries per Surgeon (surgical performance ratio)	335
% Surgeries Performed by Ophthalmologists (estimate)	100%
Number of Cataract Surgeries per Million Population (CSR)	738
Target Number of Cataract Surgeries to meet VISION 2020 Target	49,856
Shortage in Cataract Surgeries to meet VISION 2020 Target	31,415

* Data for all except Mid-level Refractionists does not include Practitioners in the Private for Profit Sector.

ND: No Data

Projected Situation: 2020						
Projected Total Population	Projected Population Living in Capital City	% Projected Population Living in Capital	Projected Population Over 50	% Projected Population Over 50	Expected 9-year Population Increase	Expected 9-year Over 50 Population Increase
30,317,636	3,449,514	11%	3,485,000	11%	22%	28%

Projected Eye Care Practitioner Workforce Dynamics: 2020											
Eye Care Cadre	Number of Practitioners in Active Workforce (2011)	Over last 3 years			Projected Net Change over next 9 years	Projected Number of Practitioners in Active Workforce	Projected Practitioners per Million Population			VISION 2020 Country Target # of Practitioners	Projected Shortage in Practitioners to meet VISION 2020
		Number Entered Workforce	Number Exited Workforce	Net Change in Practitioner Numbers			Countrywide	In Capital	Outside Capital		
Ophthalmologists	54	3	ND	ND	ND	ND	ND	ND	ND		
Cataract Surgeons	1	0	ND	ND	ND	ND	ND	ND	ND		
**Surgeons**	**55**	**3**	**ND**	**ND**	**ND**	**ND**	**ND**	**ND**	**ND**	**121**	**ND**
Ophthalmic Clinical Officers	0	0	0	0	0	0	0.0	0.0	0.0		
Ophthalmic Nurses	342	90	ND	ND	ND	ND	ND	ND	ND		
**OCOs/Nurses**	**342**	**90**	**ND**	**ND**	**ND**	**ND**	**ND**	**ND**	**ND**	**303**	**ND**
Optometrists	112	50	2	48	144	256	8.4	18.3	2.9		
Mid-level Refractionists	53	0	ND	ND	ND	ND	ND	ND	ND		
**Refractionists**	**165**	**150**	**ND**	**ND**	**ND**	**ND**	**ND**	**ND**	**ND**	**606**	**ND**

Annual Projected Cataract Surgical Performance: 2020							
Eye Care Cadre	% Surgeries Performed (estimate)	Surgical Performance Ratio per Cadre (2011)	Projected Number of Surgeons in Active Workforce	Projected Number of Cataract Surgeries Performed	Projected Number of Cataract Surgeries per Million Population (CSR)	Target Number of Cataract Surgeries to meet VISION 2020 Target	Projected Shortage in Cataract Surgeries to meet VISION 2020 Target
Ophthalmologists	100%	341	ND	ND			
Cataract Surgeons	0%	0	ND	ND			
**Surgeons**	**100%**	**335**	**ND**	**ND**	**ND**	**60,635**	**ND**

ND: No Data

Country Profile: Kenya

Key Messages

- **Surgeons:** Kenya has already met this VISION 2020 target. Accounting for population growth, an additional 32 surgeons will need to be recruited by 2020 to still be above target.
- **OCOs/Nurses:** Kenya is a third of the way to meeting this VISION 2020 target. The country will need to recruit an additional 400 OCOs and/or nurses by 2020 to meet this target.
- **Refractionists:** Data was only available from the government sector, where no refractionists are employed.
- **Cataract Surgeries:** Kenya is a quarter of the way to meeting this target. An additional 37,000 surgeries will need to be carried out each year in order to meet this target, representing a 160% increase.

VISION 2020 Targets

	Eye Care Practitioners per Million Population			Cataract Surgeries Performed per Million Population	Cataract Surgeries Performed per Surgeon
	Surgeons*	Ophthalmic Clinical Officers /Nurses	Refrac-tionists		
VISION2020	4	10	20	2,000	500
2011 Situation	4.3	3.0	No Data	553	129
On Track					

* For the Africa region this includes Ophthalmologists and Cataract Surgeons

Eye Care Practitioners: % Working Inside/Outside Capital

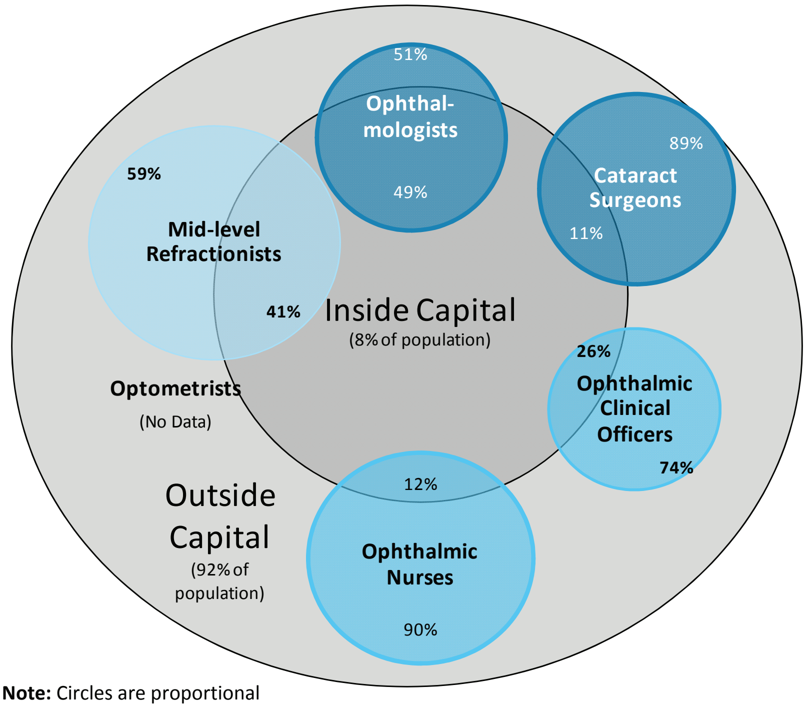

Note: Circles are proportional to numbers of eye care practitioners

In-Country Training Programmes

Surgeons	OCOs/Nurses	Refractionists
Total: 2	Total: 1	Total: 2

Distribution of Ophthalmologists

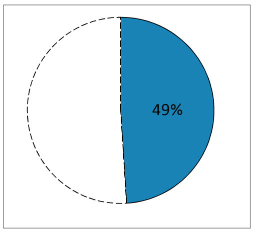

Ophthalmologists based in the capital

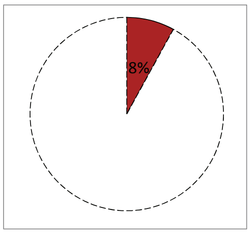

Population living in the capital

49% of Ophthalmologists treat 8% of the population

Eye Care Practitioners: Split between Sectors

Government | NGO/Mission | Private for Profit

Surgeons

Ophthalmologists

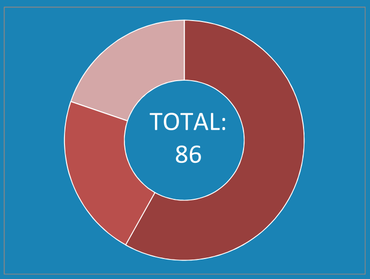

Cataract Surgeons

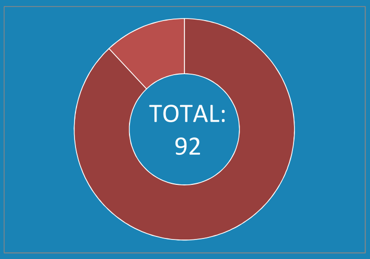

OCOs/Nurses

Ophthalmic Clinical Officers

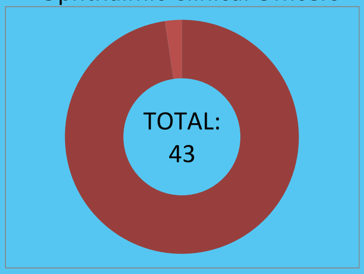

Ophthalmic Nurses

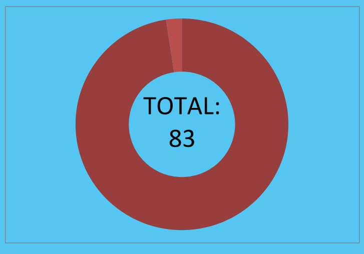

Refractionists

Optometrists

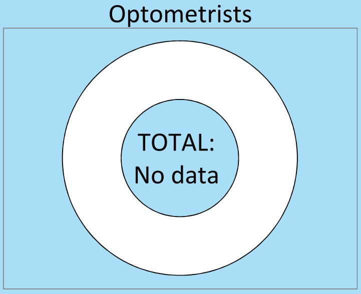

Mid-level Refractionists

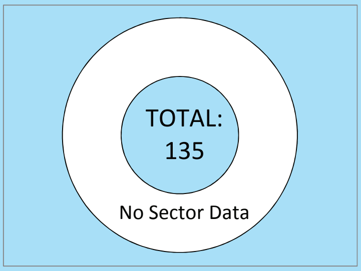

Surgeons: Current & Projected Workforce per Million Population

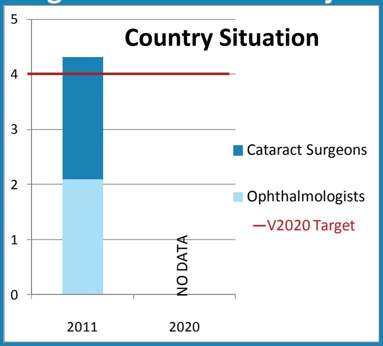

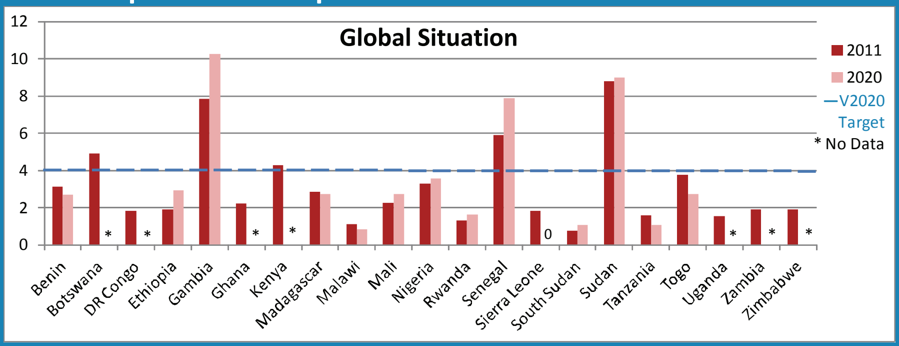

Practitioner Entry vs Exit: Over the past 3 years, 15 ophthalmologists and 24 cataract surgeons have entered the workforce. However, there is no information on how many have exited the workforce, so projections for the situation in 2020 cannot be made.

Practitioner Working Location: Although Kenya has currently met the VISION 2020 target, with a practitioner to population ratio of 4.3, this is not the case for practitioners based outside Nairobi, for which the ratio is 3.3. The surgeon to population ratio for those based in Nairobi is 15.5.

Practitioner vs Population Growth: Taking into account the expected 26% population growth between 2011 and 2020, the surgeon population needs to increase by 18% to continue to meet the target by 2020.

OCOs/Nurses: Current & Projected Workforce per Million Population

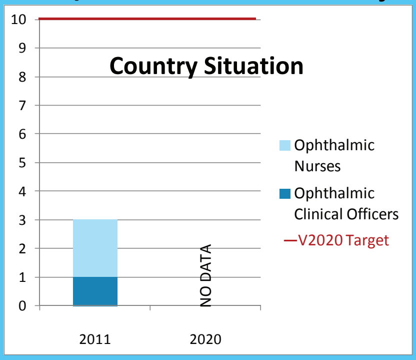

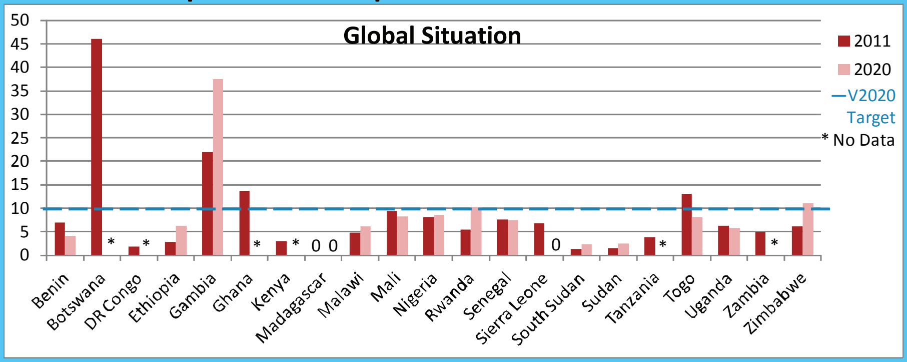

Practitioner Entry vs Exit: Over the past 3 years, no ophthalmic clinical officers and 31 ophthalmic nurses have entered the workforce. However, there is no information on how many have exited the workforce, so projections for the situation in 2020 cannot be made.

Practitioner Working Location: The practitioner to population ratio for those working in the capital is 6.2 compared to 2.8 for those working outside the capital.

Practitioner vs Population Growth: The number of practitioners will need to more than quadruple to meet the target by 2020, taking into account the expected 26% population growth rate over this period.

Refractionists: Current & Projected Workforce per Million Population

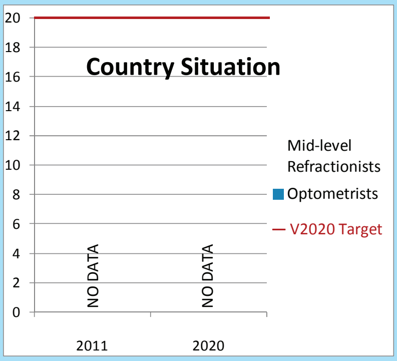

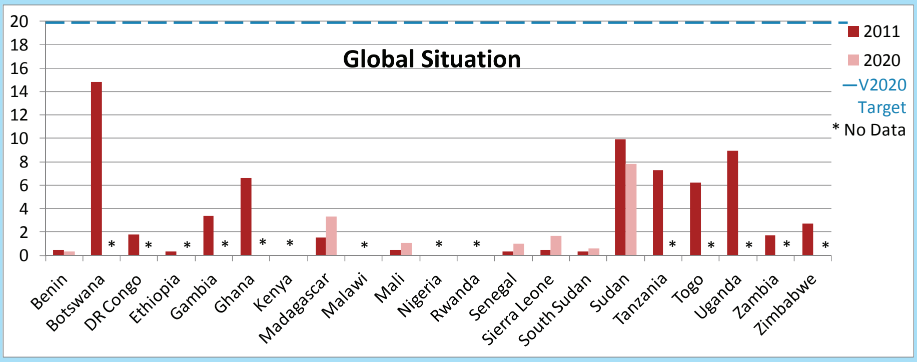

Practitioner Entry vs Exit: The number of optometrists, all of whom work outside the government sector, is not known. There are 135 mid-level refractionists currently working outside the government sector, but no information is available on entry or exit from the workforce.

Practitioner Working Location: The mid-level refractionist to population ratio is 3.2 for the overall population and 16.4 and 2.1 respectively for those working inside and outside the capital.

Practitioner vs Population Growth: Taking into account population growth, Kenya will need a total of 1,051 refractionists by 2020 to meet the target. There are currently 135 mid-level refractionists.

Cataract Surgical Performance: Current & Projected Performance per Million Population

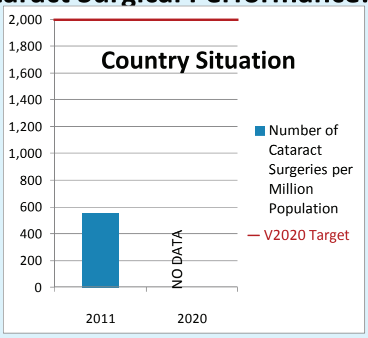

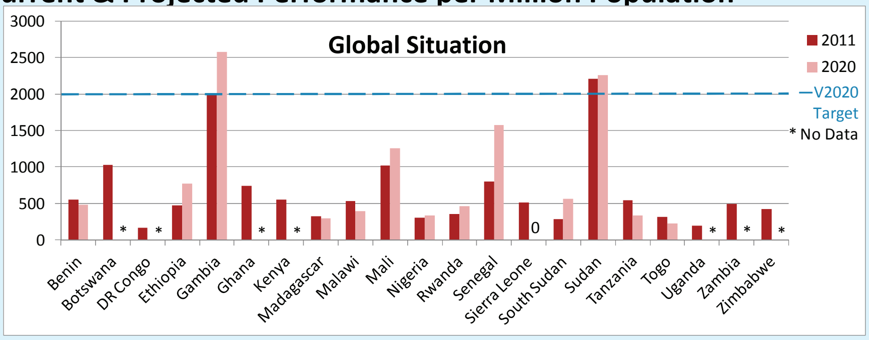

Kenya will need to perform nearly five times as many annual cataract surgeries by 2020 in order to meet the VISION 2020 target, taking into account expected population growth.

Country Profile: Kenya

Current Situation: 2011

Total Population	Population Living in Capital City	% Population Living in Capital	Population Over 50	% Population Over 50
41,580,218	3,363,000	8%	3,763,000	9%

VISION 2020 Targets

Eye Care Cadre	# Practitioners per Million Population
Surgeons	4
OCOs/Nurses	10
Refractionists	20

Characteristics of the Active Eye Care Practitioner Workforce: 2011

Eye Care Cadre	Number of Practitioners in Active Workforce	Sector			Location		# Training Programmes available in Country
		Government	NGO/ Mission	Private for Profit	Capital	Outside Capital	
Ophthalmologists	86	50	19	17	42	44	1
Cataract Surgeons	92	81	11	0	10	82	1
**Surgeons**	**178**	**131**	**30**	**17**	**52**	**126**	**2**
Ophthalmic Clinical Officers	43	42	1	0	11	32	0
Ophthalmic Nurses	83	81	2	0	10	75	1
**OCOs/Nurses**	**126**	**123**	**3**	**0**	**21**	**107**	**1**
Optometrists	ND	0	0	ND	ND	ND	1
Mid-level Refractionists	135	0	ND	ND	55	80	1
**Refractionists**	**ND**	**0**	**ND**	**ND**	**ND**	**ND**	**2**

Eye Care Practitioner Workforce Dynamics: 2011

Eye Care Cadre	Number of Practitioners in Active Workforce	Practitioners per Million Population			VISION 2020 Country Target # of Practitioners	Shortage in Practitioners to meet Target
		Countrywide	In Capital	Outside Capital		
Ophthalmologists	86	2.1	12.5	1.2		
Cataract Surgeons	92	2.2	3.0	2.1		
**Surgeons**	**178**	**4.3**	**15.5**	**3.3**	**166**	**Target Met**
Ophthalmic Clinical Officers	43	1	3.3	0.8		
Ophthalmic Nurses	83	2	3.0	2.0		
**OCOs/Nurses**	**126**	**3**	**6.2**	**2.8**	**416**	**290**
Optometrists	ND	ND	ND	ND		
Mid-level Refractionists	135	3.2	16.4	2.1		
**Refractionists**	**ND**	**ND**	**ND**	**ND**	**832**	**ND**

Annual Cataract Surgical Performance

Number of Cataract Surgeries Performed (data from 2010)	23,006
Number of Cataract Surgeries per Surgeon (surgical performance ratio)	129
% Surgeries Performed by Ophthalmologists (estimate)	28%
Number of Cataract Surgeries per Million Population (CSR)	553
Target Number of Cataract Surgeries to meet VISION 2020 Target	83,160
Shortage in Cataract Surgeries to meet VISION 2020 Target	60,155

ND: No Data

Projected Situation: 2020

Projected Total Population	Projected Population Living in Capital City	% Projected Population Living in Capital	Projected Population Over 50	% Projected Population Over 50	Expected 9-year Population Increase	Expected 9-year Over 50 Population Increase
52,547,084	4,907,122	9%	5,209,000	10%	26%	38%

Projected Eye Care Practitioner Workforce Dynamics: 2020

Eye Care Cadre	Number of Practitioners in Active Workforce (2011)	Over last 3 years			Projected Net Change over next 9 years	Projected Number of Practitioners in Active Workforce	Projected Practitioners per Million Population			VISION 2020 Country Target # of Practitioners	Projected Shortage in Practitioners to meet VISION 2020
		Number Entered Workforce	Number Exited Workforce	Net Change in Practitioner Numbers			Countrywide	In Capital	Outside Capital		
Ophthalmologists	86	15	ND	ND	ND	ND	ND	ND	ND		
Cataract Surgeons	92	24	ND	ND	ND	ND	ND	ND	ND		
**Surgeons**	**178**	**39**	**ND**	**ND**	**ND**	**ND**	**ND**	**ND**	**ND**	**210**	**ND**
Ophthalmic Clinical Officers	43	0	ND	ND	ND	ND	ND	ND	ND		
Ophthalmic Nurses	83	31	ND	ND	ND	ND	ND	ND	ND		
**OCOs/Nurses**	**126**	**31**	**ND**	**ND**	**ND**	**ND**	**ND**	**ND**	**ND**	**525**	**ND**
Optometrists	ND	ND	ND	ND	ND	ND	ND	ND	ND		
Mid-level Refractionists	135	ND	ND	ND	ND	ND	ND	ND	ND		
**Refractionists**	**ND**	**ND**	**ND**	**ND**	**ND**	**ND**	**ND**	**ND**	**ND**	**1,051**	**ND**

Annual Projected Cataract Surgical Performance: 2020

Eye Care Cadre	% Surgeries Performed (estimate)	Surgical Performance Ratio per Cadre (2011)	Projected Number of Surgeons in Active Workforce	Projected Number of Cataract Surgeries Performed	Projected Number of Cataract Surgeries per Million Population (CSR)	Target Number of Cataract Surgeries to meet VISION 2020 Target	Projected Shortage in Cataract Surgeries to meet VISION 2020 Target
Ophthalmologists	28%	75	ND	ND			
Cataract Surgeons	72%	180	ND	ND			
**Surgeons**	**100%**	**129**	**ND**	**ND**	**ND**	**105,094**	**ND**

ND: No Data

Country Profile: Madagascar

International Centre for Eye Health | AVRI African Vision Research Institute | Sightsavers

Key Messages

- **Surgeons:** The ophthalmologist workforce is currently decreasing. Madagascar is less than a third of the way to meeting this target and will be even further away by 2020.
- **OCOs/Nurses:** Madagascar has no ophthalmic clinical officers or ophthalmic nurses working in the country and currently has no training programme for this cadre.
- **Refractionists:** There are no optometrists in Madagascar, although two are currently being trained abroad. Madagascar will need to recruit 450 refractionsists to meet this target.
- **Cataract Surgeries:** Cataract surgeries are decreasing relative to population growth. To meet this target by 2020 the country needs to perform 7 times as many surgeries each year.

VISION 2020 Targets

	Eye Care Practitioners per Million Population			Cataract Surgeries Performed per Million Population	Cataract Surgeries Performed per Surgeon
	Surgeons*	Ophthalmic Clinical Officers /Nurses	Refrac-tionists		
VISION2020 Target	4	10	20	2,000	500
2011 Situation	2.8	0.0	1.5	325	115
On Track					

* For the Africa region this includes Ophthalmologists and Cataract Surgeons

Eye Care Practitioners: % Working Inside/Outside Capital

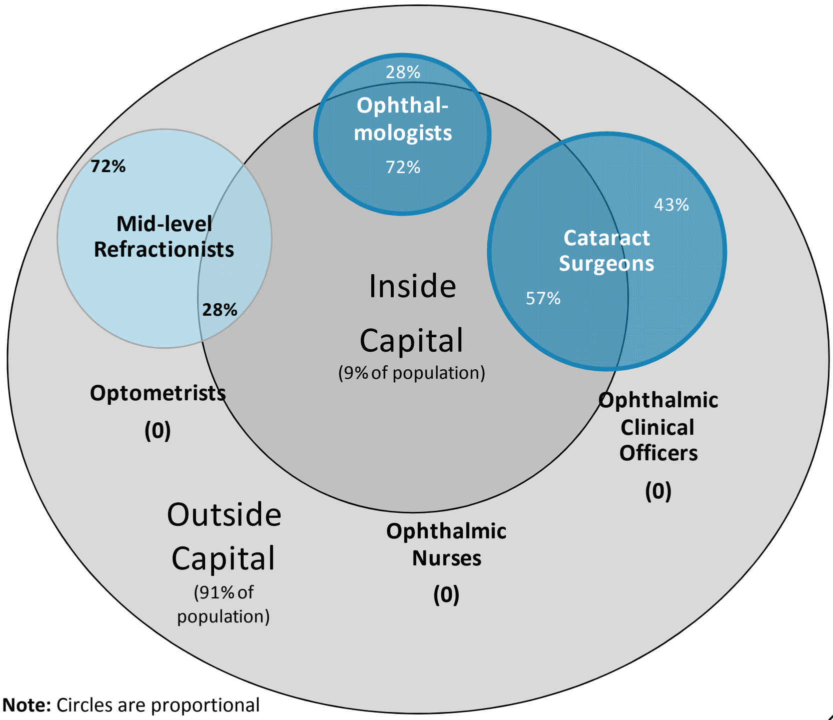

Note: Circles are proportional to numbers of eye care practitioners

In-Country Training Programmes

Surgeons	OCOs/Nurses	Refractionists
Total: 3	Total: 0	Total: 4

Distribution of Surgeons

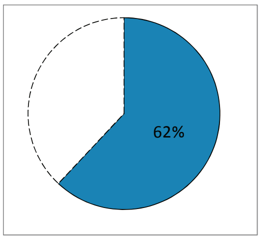

Surgeons based in the capital

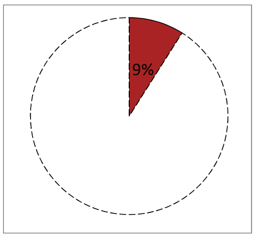

Population living in the capital

62% of Surgeons treat 9% of the population

Eye Care Practitioners: Split between Sectors

Government | NGO/Mission | Private for Profit

Surgeons

Ophthalmologists

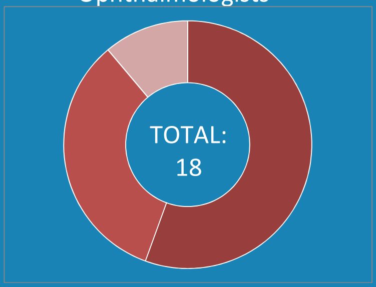

Cataract Surgeons

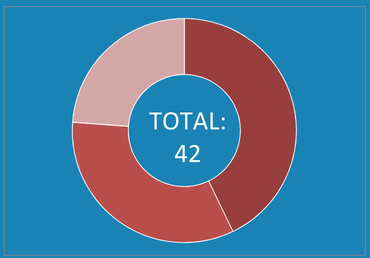

OCOs/Nurses

Ophthalmic Clinical Officers

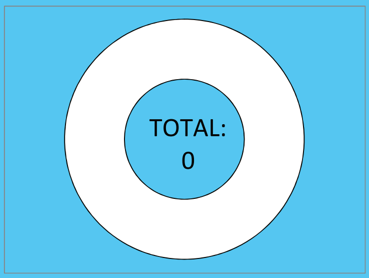

Ophthalmic Nurses

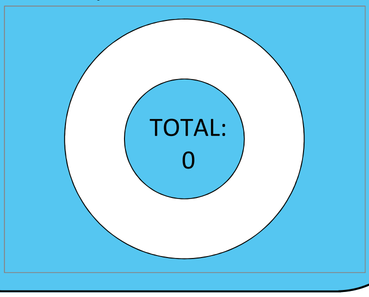

Refractionists

Optometrists

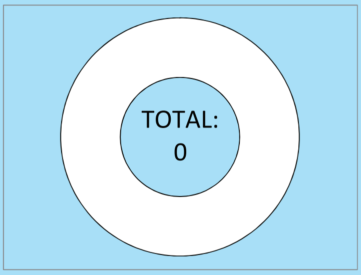

Mid-level Refractionists

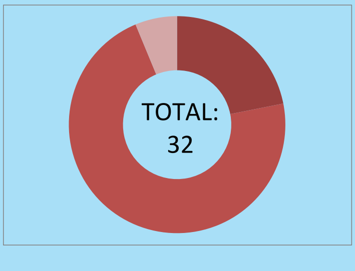

Surgeons: Current & Projected Workforce per Million Population

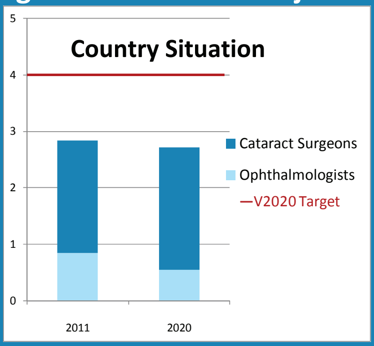

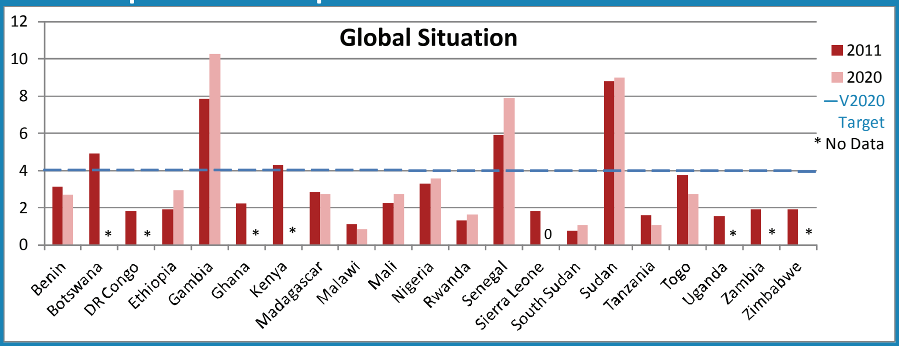

Practitioner Entry vs Exit: Over the past 3 years more ophthalmologists have exited the workforce than have entered.

Practitioner Working Location: Madagascar is more than half way to meeting the VISION 2020 target with a surgeon to population ratio of 2.8. This ratio is 18.6 and 1.2 respectively for surgeons working inside and outside the capital.

Practitioner vs Population Growth: If current trends continue, the general population will increase slightly faster than the number of surgeons, at rates of 29% and 25% respectively, meaning that unless trends change, Madagascar will never meet this target.

OCOs/Nurses: Current & Projected Workforce per Million Population

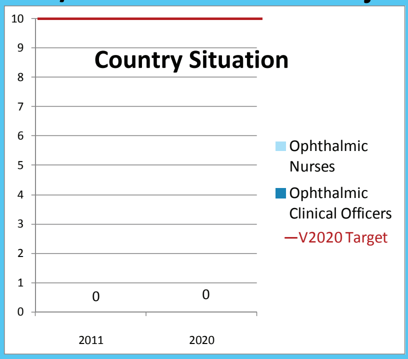

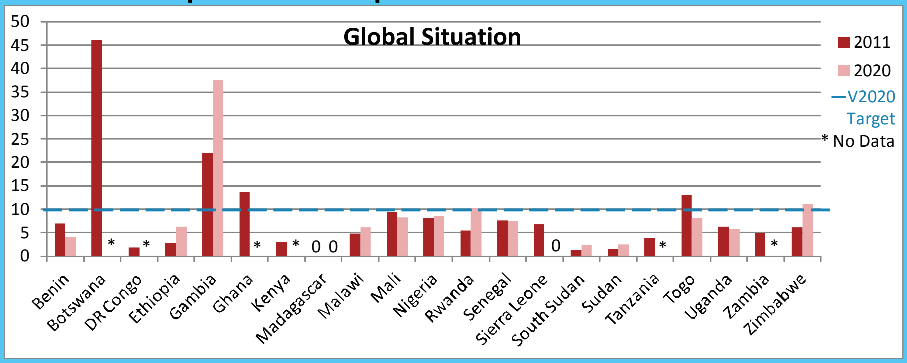

Madagascar has no ophthalmic clinical officers or ophthalmic nurses working in the country and currently has no training programme for this cadre.

Refractionists: Current & Projected Workforce per Million Population

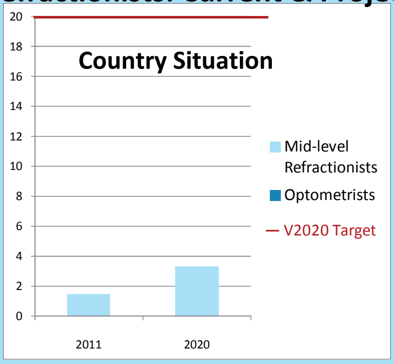

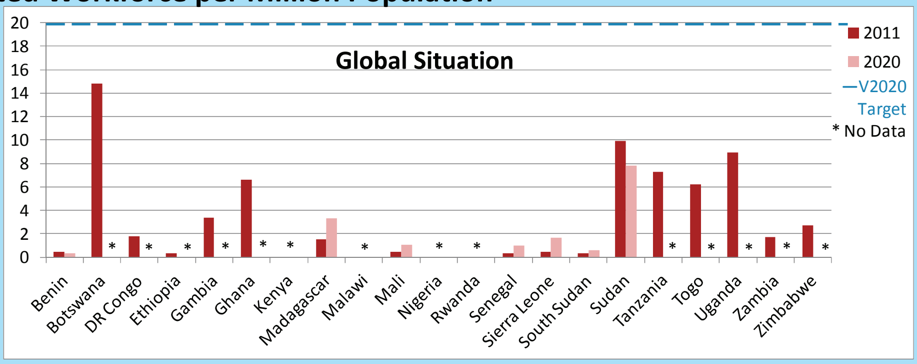

Practitioner Entry vs Exit: Although the number of refractionists is expected to nearly triple between 2011 and 2020 this is still not enough growth to meet the VISION 2020 target.

Practitioner Working Location: The mid-level refractionist to population ratio is 1.5 for the overall population and 4.5 and 1.2 respectively for those working inside and outside the capital.

Practitioner vs Population Growth: Taking into account the expected 29% population growth over this period, Madagascar will need to recruit more than 17 times as many refractionists as are currently employed in order to meet the target by 2020.

Cataract Surgical Performance: Current & Projected Performance per Million Population

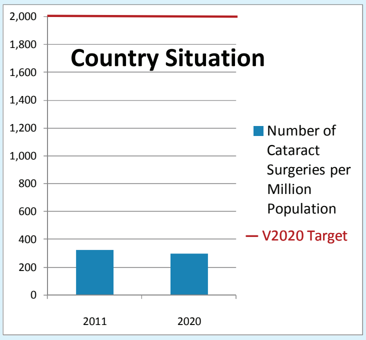

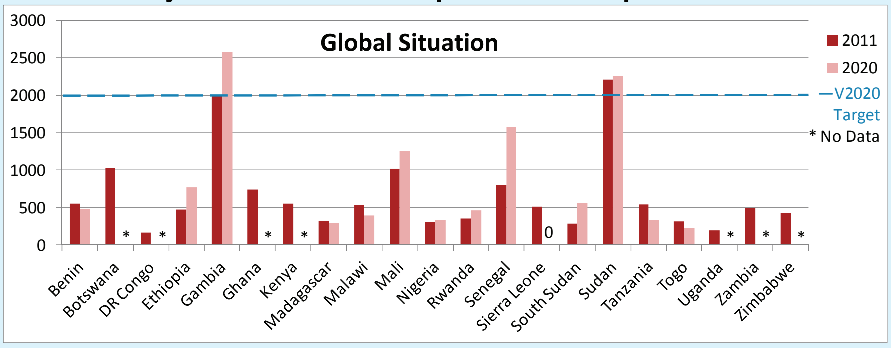

Taking into account the expected population growth, Madagascar will need a 700% increase in the annual number of cataract surgeries in order to meet this target by 2020.

Country Profile: Madagascar

VISION 2020 Targets

Eye Care Cadre	# Practitioners per Million Population
Surgeons	4
OCOs/Nurses	10
Refractionists	20

Current Situation: 2011

Total Population	Population Living in Capital City	% Population Living in Capital	Population Over 50	% Population Over 50
21,311,411	1,987,000	9%	2,088,000	10%

Characteristics of the Active Eye Care Practitioner Workforce: 2011

Eye Care Cadre	Number of Practitioners in Active Workforce	Sector			Location		# Training Programmes available in Country
		Government	NGO/ Mission	Private for Profit	Capital	Outside Capital	
Ophthalmologists	18	10	6	2	13	5	2
Cataract Surgeons	42	18	14	10	24	18	1
**Surgeons**	**60**	**28**	**20**	**12**	**37**	**23**	**3**
Ophthalmic Clinical Officers	0	0	0	0	0	0	0
Ophthalmic Nurses	0	0	0	0	0	0	0
**OCOs/Nurses**	**0**	**0**	**0**	**0**	**0**	**0**	**0**
Optometrists	0	0	0	0	0	0	0
Mid-level Refractionists	32	7	23	2	9	23	4
**Refractionists**	**32**	**7**	**23**	**2**	**9**	**23**	**4**

Eye Care Practitioner Workforce Dynamics: 2011

Eye Care Cadre	Number of Practitioners in Active Workforce	Practitioners per Million Population			VISION 2020 Country Target # of Practitioners	Shortage in Practitioners to meet Target
		Countrywide	In Capital	Outside Capital		
Ophthalmologists	18	0.8	6.5	0.3		
Cataract Surgeons	42	2.0	12.1	0.9		
**Surgeons**	**60**	**2.8**	**18.6**	**1.2**	**85**	**25**
Ophthalmic Clinical Officers	0	0.0	0.0	0.0		
Ophthalmic Nurses	0	0.0	0.0	0.0		
**OCOs/Nurses**	**0**	**0.0**	**0.0**	**0.0**	**213**	**213**
Optometrists	0	0.0	0.0	0.0		
Mid-level Refractionists	32	1.5	4.5	1.2		
**Refractionists**	**32**	**1.5**	**4.5**	**1.2**	**426**	**394**

Annual Cataract Surgical Performance

Number of Cataract Surgeries Performed (data from 2010)	6,919
Number of Cataract Surgeries per Surgeon (surgical performance ratio)	115
% Surgeries Performed by Ophthalmologists (estimate)	40%
Number of Cataract Surgeries per Million Population (CSR)	325
Target Number of Cataract Surgeries to meet VISION 2020 Target	42,623
Shortage in Cataract Surgeries to meet VISION 2020 Target	35,704

Projected Situation: 2020

Projected Total Population	Projected Population Living in Capital City	% Projected Population Living in Capital	Projected Population Over 50	% Projected Population Over 50	Expected 9-year Population Increase	Expected 9-year Over 50 Population Increase
27,528,364	2,978,954	11%	2,924,000	11%	29%	40%

Projected Eye Care Practitioner Workforce Dynamics: 2020

Eye Care Cadre	Number of Practitioners in Active Workforce (2011)	Over last 3 years			Projected Net Change over next 9 years	Projected Number of Practitioners in Active Workforce	Projected Practitioners per Million Population			VISION 2020 Country Target # of Practitioners	Projected Shortage in Practitioners to meet VISION 2020
		Number Entered Workforce	Number Exited Workforce	Net Change in Practitioner Numbers			Countrywide	In Capital	Outside Capital		
Ophthalmologists	18	2	3	-1	-3	15	0.5	3.6	0.2		
Cataract Surgeons	42	8	2	6	18	60	2.2	11.5	1.0		
**Surgeons**	**60**	**10**	**5**	**5**	**15**	**75**	**2.7**	**15.5**	**1.2**	**110**	**35**
Ophthalmic Clinical Officers	0	0	0	0	0	0	0.0	0.0	0.0		
Ophthalmic Nurses	0	0	0	0	0	0	0.0	0.0	0.0		
**OCOs/Nurses**	**0**	**0**	**0**	**0**	**0**	**0**	**0.0**	**0.0**	**0.0**	**275**	**275**
Optometrists	0	0	0	0	0	0	0.0	0.0	0.0		
Mid-level Refractionists	32	21	1	20	60	92	3.3	8.7	2.7		
**Refractionists**	**32**	**21**	**1**	**20**	**60**	**92**	**3.3**	**8.7**	**2.7**	**551**	**459**

Annual Projected Cataract Surgical Performance: 2020

Eye Care Cadre	% Surgeries Performed (estimate)	Surgical Performance Ratio per Cadre (2011)	Projected Number of Surgeons in Active Workforce	Projected Number of Cataract Surgeries Performed	Projected Number of Cataract Surgeries per Million Population (CSR)	Target Number of Cataract Surgeries to meet VISION 2020 Target	Projected Shortage in Cataract Surgeries to meet VISION 2020 Target
Ophthalmologists	40%	154	15	2,306			
Cataract Surgeons	60%	99	60	5,931			
**Surgeons**	**100%**	**115**	**75**	**8,237**	**299**	**55,057**	**46,820**

Country Profile: Malawi

International Centre for Eye Health | AVRI African Vision Research Institute | Sightsavers

Key Messages

- **Surgeons:** Malawi is a quarter of the way to meeting this target. There is no growth in this cadre, meaning that with population growth, Malawi will be even further from meeting this target by 2020.
- **OCOs/Nurses:** Malawi has no ophthalmic nurses. The country is currently half way to meeting this target with the presence of ophthalmic clinical officers.
- **Refractionists:** There was insufficient data on this cadre.
- **Cataract Surgeries:** Malawi is a quarter of the way to meeting this target and will be even further from the target as cataract surgeries are currently decreasing relative to population growth.

VISION 2020 Targets

	Eye Care Practitioners per Million Population			Cataract Surgeries Performed per Million Population	Cataract Surgeries Performed per Surgeon
	Surgeons*	Ophthalmic Clinical Officers /Nurses	Refrac-tionists		
VISION2020 Target	4	10	20	2,000	500
2011 Situation	1.1	4.8	No Data	533	483
On Track					

* For the Africa region this includes Ophthalmologists and Cataract Surgeons

Eye Care Practitioners: % Working Inside/Outside Capital

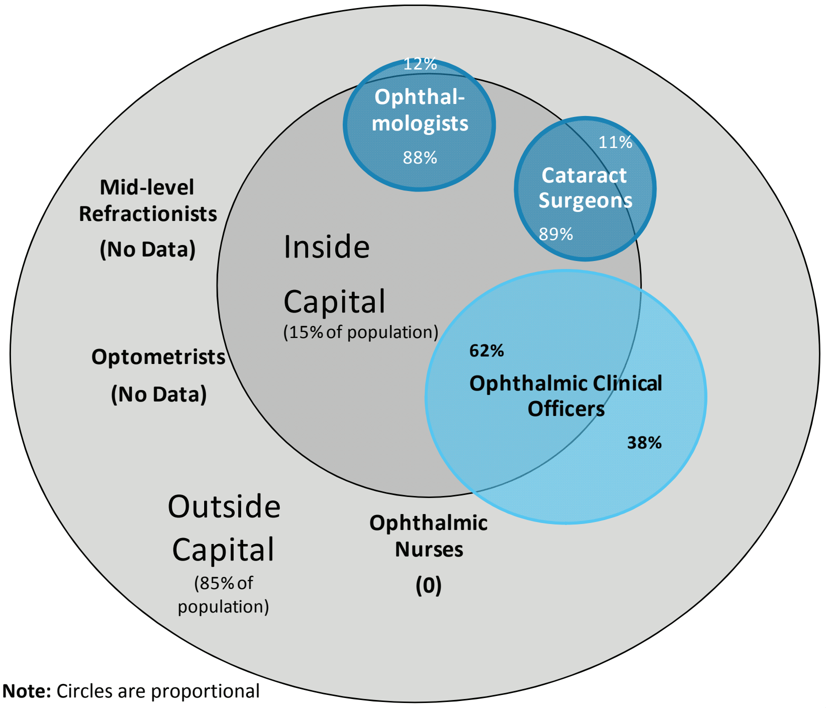

Note: Circles are proportional to numbers of eye care practitioners

In-Country Training Programmes

Surgeons — Total: 2

OCOs/Nurses — Total: 1

Refractionists — Total: No Data

Distribution of Surgeons

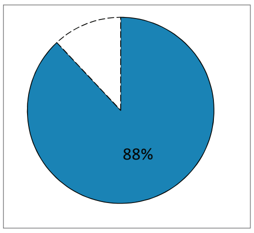

Surgeons based in the capital

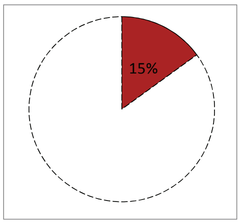

Population living in the capital

88% of Surgeons treat 15% of the population

Eye Care Practitioners: Split between Sectors

Government | NGO/Mission | Private for Profit

Surgeons

Ophthalmologists

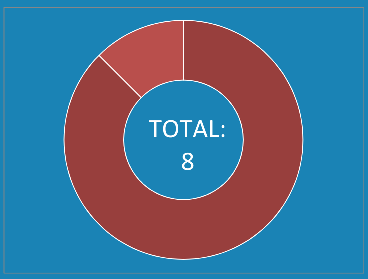

Cataract Surgeons

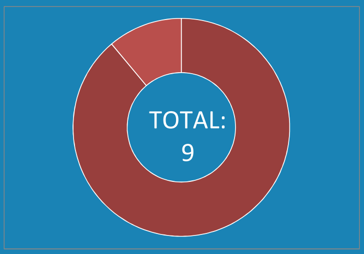

OCOs/Nurses

Ophthalmic Clinical Officers

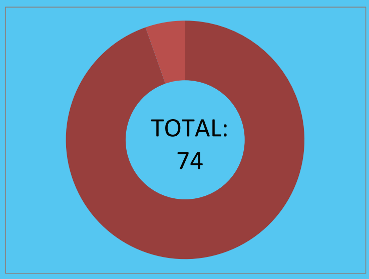

Ophthalmic Nurses

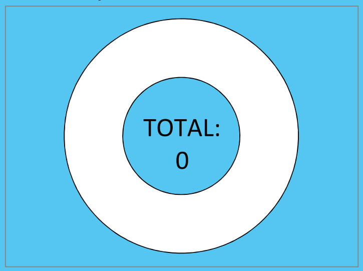

Refractionists

Optometrists

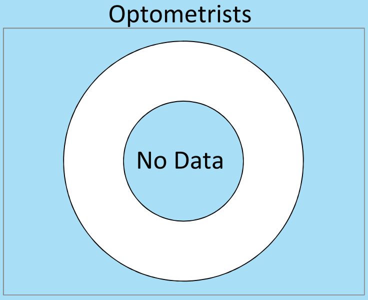

Mid-level Refractionists

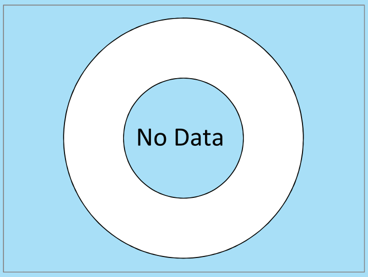

Surgeons: Current & Projected Workforce per Million Population

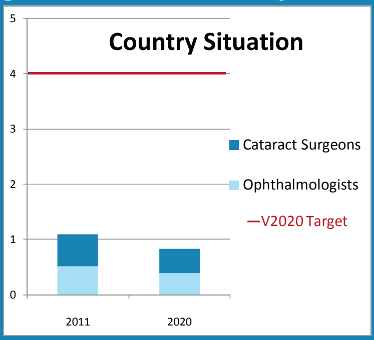

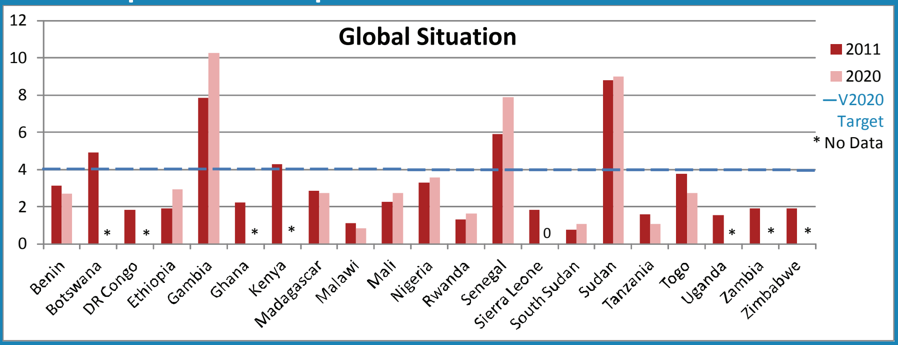

Practitioner Entry vs Exit: As many surgeons have exited the workforce as have entered over the past 3 years, meaning the current surgeon to population ratio of 1.1 will decrease to 0.83 by 2020 taking into account population growth.

Practitioner Working Location: Given that 88% of surgeons work in the 3 largest cities, the surgeon to population ratio is 6.5 for those working in these cities and 0.2 for those working outside these cities.

Practitioner vs Population Growth: To account for the expected 34% population growth over this period, Malawi needs to increase the number of surgeons by nearly 400% to meet this target by 2020.

OCOs/Nurses: Current & Projected Workforce per Million Population

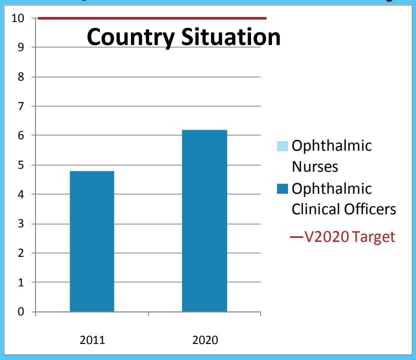

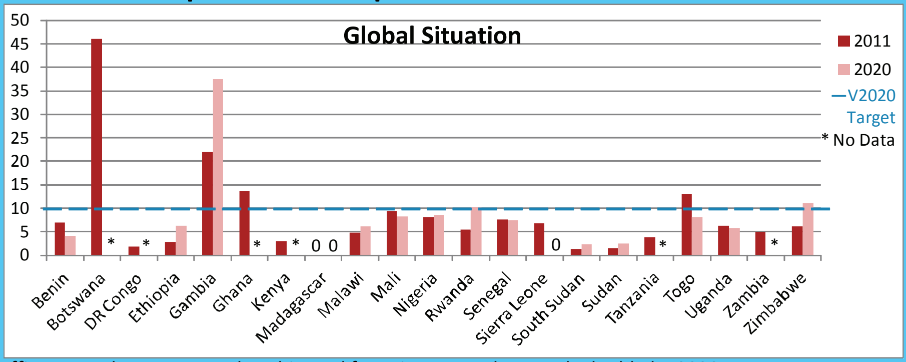

Practitioner Entry vs Exit: The high ophthalmic clinical officer growth rate means that this workforce is expected to nearly double by 2020.

Practitioner Working Location: The ophthalmic clinical officer to population ratio is 4.8 for the overall population and 19.9 and 2.1 respectively for those working inside and outside the three largest cities.

Practitioner vs Population Growth: Although the number of nurses is increasing, the proportional increase in the number of nurses will eventually be expected to be outpaced by proportional general population increases. Therefore, without additional intervention the target will never be met.

Refractionists: Current & Projected Workforce per Million Population

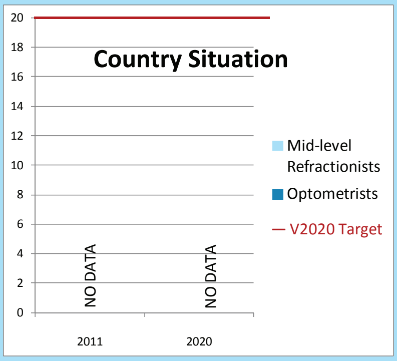

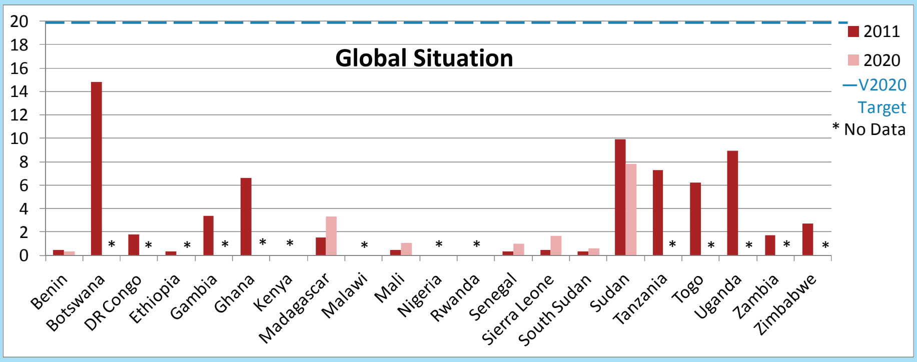

There is no data on the refractionist population of Malawi.

Cataract Surgical Performance: Current & Projected Performance per Million Population

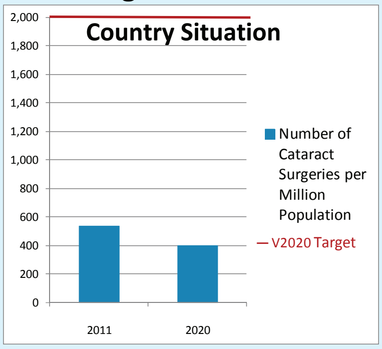

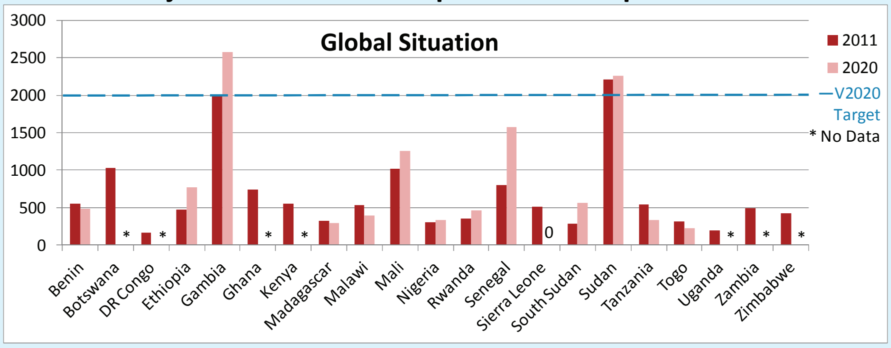

Given the increase in population, the annual number of cataract surgeries needs to more than quadruple in order to meet the VISION 2020 target by 2020.

Country Profile: Malawi

Current Situation: 2011

Total Population	Population Living in Capital City*	% Population Living in Capital	Population Over 50	% Population Over 50
15,397,121	2,316,000	15%	1,439,000	9%

VISION 2020 Targets

Eye Care Cadre	# Practitioners per Million Population
Surgeons	4
OCOs/Nurses	10
Refractionists	20

Characteristics of the Active Eye Care Practitioner Workforce: 2011

Eye Care Cadre	Number of Practitioners in Active Workforce	Sector			Location		# Training Programmes available in Country
		Government	NGO/ Mission	Private for Profit	Capital*	Outside Capital	
Ophthalmologists	8	7	1	0	7	1	1
Cataract Surgeons	9	8	1	0	8	1	1
**Surgeons**	**17**	**15**	**2**	**0**	**15**	**2**	**2**
Ophthalmic Clinical Officers	74	70	4	0	46	28	1
Ophthalmic Nurses	0	0	0	0	0	0	0
**OCOs/Nurses**	**74**	**70**	**4**	**0**	**46**	**28**	**1**
Optometrists	ND	3	1	ND	4	0	1
Mid-level Refractionists	ND	ND	ND	ND	ND	ND	ND
**Refractionists**	**ND**	**ND**	**ND**	**ND**	**ND**	**ND**	**ND**

Eye Care Practitioner Workforce Dynamics: 2011

Eye Care Cadre	Number of Practitioners in Active Workforce	Practitioners per Million Population			VISION 2020 Country Target # of Practitioners	Shortage in Practitioners to meet Target
		Countrywide	In Capital	Outside Capital		
Ophthalmologists	8	0.5	3.0	0.1		
Cataract Surgeons	9	0.6	3.5	0.1		
**Surgeons**	**17**	**1.1**	**6.5**	**0.2**	**62**	**45**
Ophthalmic Clinical Officers	74	4.8	19.9	2.1		
Ophthalmic Nurses	0	0	0.0	0.0		
**OCOs/Nurses**	**74**	**4.8**	**19.9**	**2.1**	**154**	**80**
Optometrists	ND	ND	1.7	0.0		
Mid-level Refractionists	ND	ND	ND	ND		
**Refractionists**	**ND**	**ND**	**ND**	**ND**	**298**	**ND**

Annual Cataract Surgical Performance

Number of Cataract Surgeries Performed (estimate from IAPB)	8,208
Number of Cataract Surgeries per Surgeon (surgical performance ratio)	483
% Surgeries Performed by Ophthalmologists (estimate)	75%
Number of Cataract Surgeries per Million Population (CSR)	533
Target Number of Cataract Surgeries to meet VISION 2020 Target	30,794
Shortage in Cataract Surgeries to meet VISION 2020 Target	22,586

* 'Capital' interpreted as 3 largest cities in the country

ND: No Data

Projected Situation: 2020

Projected Total Population	Projected Population Living in Capital City	% Projected Population Living in Capital	Projected Population Over 50	% Projected Population Over 50	Expected 9-year Population Increase	Expected 9-year Over 50 Population Increase
20,677,512	3,401,842	16%	1,765,000	9%	34%	23%

Projected Eye Care Practitioner Workforce Dynamics: 2020

Eye Care Cadre	Number of Practitioners in Active Workforce (2011)	Over last 3 years			Projected Net Change over next 9 years	Projected Number of Practitioners in Active Workforce	Projected Practitioners per Million Population			VISION 2020 Country Target # of Practitioners	Projected Shortage in Practitioners to meet VISION 2020
		Number Entered Workforce	Number Exited Workforce	Net Change in Practitioner Numbers			Countrywide	In Capital	Outside Capital		
Ophthalmologists	8	2	2	0	0	8	0.4	2.1	0.1		
Cataract Surgeons	9	2	2	0	0	9	0.4	2.4	0.1		
**Surgeons**	**17**	**4**	**4**	**0**	**0**	**17**	**0.8**	**4.4**	**0.1**	**83**	**66**
Ophthalmic Clinical Officers	74	25	7	18	54	128	6.2	23.4	2.8		
Ophthalmic Nurses	0	0	0	0	0	0	0.0	0.0	0.0		
**OCOs/Nurses**	**74**	**25**	**7**	**18**	**54**	**128**	**6.2**	**23.4**	**2.8**	**207**	**79**
Optometrists	ND	0	ND	ND	ND	ND	ND	ND	ND		
Mid-level Refractionists	ND	ND	ND	ND	ND	ND	ND	ND	ND		
**Refractionists**	**ND**	**ND**	**ND**	**ND**	**ND**	**ND**	**ND**	**ND**	**ND**	**414**	**ND**

Annual Projected Cataract Surgical Performance: 2020

Eye Care Cadre	% Surgeries Performed (estimate)	Surgical Performance Ratio per Cadre (2011)	Projected Number of Surgeons in Active Workforce	Projected Number of Cataract Surgeries Performed	Projected Number of Cataract Surgeries per Million Population (CSR)	Target Number of Cataract Surgeries to meet VISION 2020 Target	Projected Shortage in Cataract Surgeries to meet VISION 2020 Target
Ophthalmologists	75%	ND	8	ND			
Cataract Surgeons	25%	ND	9	ND			
**Surgeons**	**100%**	**ND**	**17**	**ND**	**364**	**41,355**	**33,831**

ND: No Data

Country Profile: Mali

Key Messages

- **Surgeons:** The current ophthalmologist growth rate is not enough to account for the overall population growth. Unless trends change, Mali will never meet the VISION 2020 target.
- **OCOs/Nurses:** This target had almost been met in Mali in 2011. Due to practitioner growth being slower than population growth, Mali is projected to never meet this target.
- **Refractionists:** There are 7 optometrists and no mid-level refractionsists. Mali needs to recruit nearly 400 staff in this cadre.
- **Cataract Surgeries:** Cataract surgeries are increasing relative to population growth. However, to meet the VISION 2020 target, these will need to increase by a further 60%.

VISION 2020 Targets

	Eye Care Practitioners per Million Population			Cataract Surgeries Performed per Million Population	Cataract Surgeries Performed per Surgeon
	Surgeons*	Ophthalmic Clinical Officers /Nurses	Refrac-tionists		
VISION2020 Target	4	10	20	2,000	500
2011 Situation	2.3	9.5	0.4	1,016	446
On Track					

* For the Africa region this includes Ophthalmologists and Cataract Surgeons

Eye Care Practitioners: % Working Inside/Outside Capital

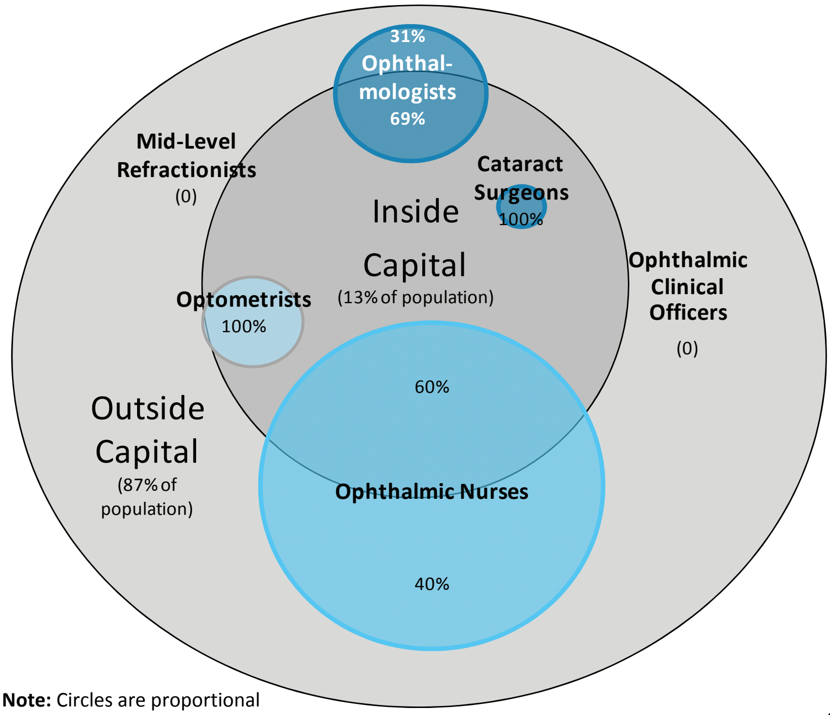

Note: Circles are proportional to numbers of eye care practitioners

In-Country Training Programmes

Surgeons	OCOs/Nurses	Refractionists
Total: 1	Total: 1	Total: 3

Distribution of Ophthalmic Nurses

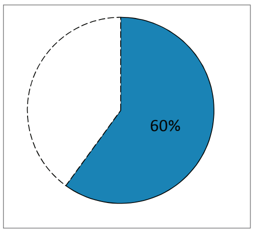

Ophthalmic Nurses based in the capital

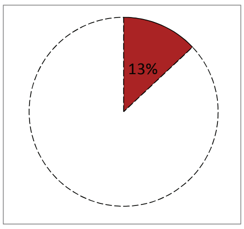

Population living in the capital

60% of Ophthalmic Nurses treat 13% of the population

Eye Care Practitioners: Split between Sectors

Government · NGO/Mission · Private for Profit

Surgeons

Ophthalmologists

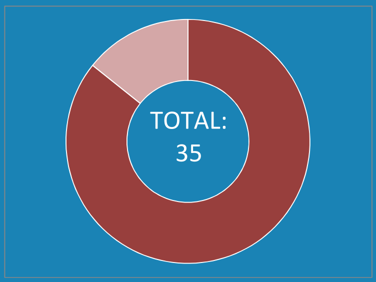

Cataract Surgeons

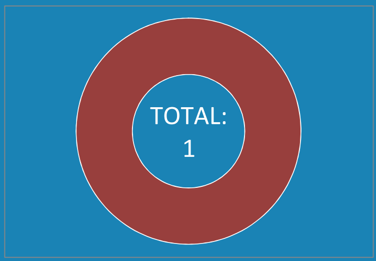

OCOs/Nurses

Ophthalmic Clinical Officers

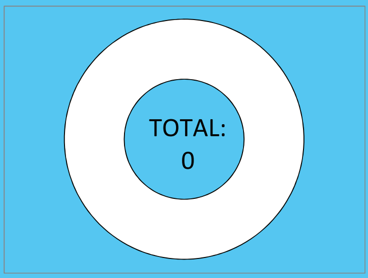

Ophthalmic Nurses

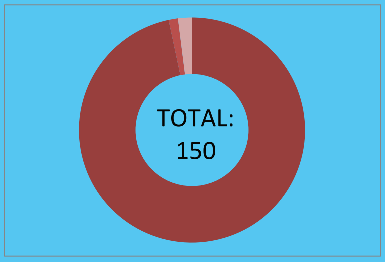

Refractionists

Optometrists

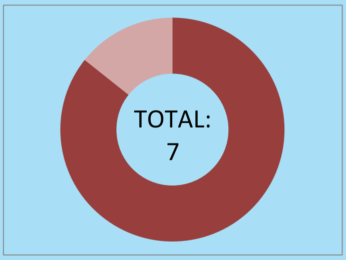

Mid-level Refractionists

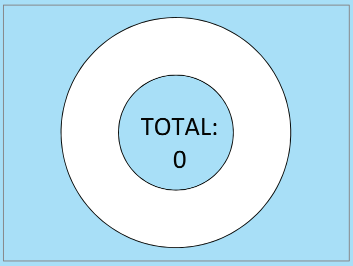

Surgeons: Current & Projected Workforce per Million Population

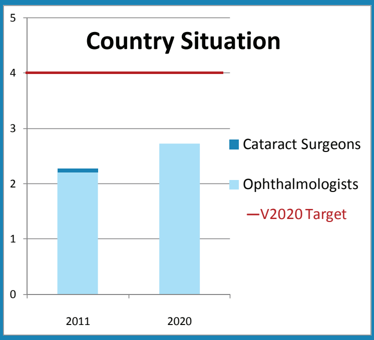

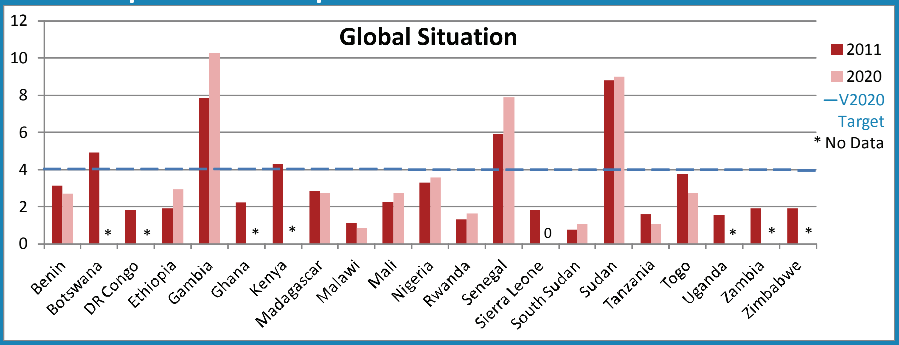

Practitioner Entry vs Exit: The number of surgeons is expected to increase by 60% between 2011 and 2020.
Practitioner Working Location: The surgeon to population ratio is 2.3 for the overall population and 12.3 and 0.8 respectively for those working inside and outside the capital.
Practitioner vs Population Growth: Although the number of surgeons is increasing, the proportional increase in number of surgeons will eventually be expected to be outpaced by proportional general population increases. Therefore, without additional intervention the target will never be met.

OCOs/Nurses: Current & Projected Workforce per Million Population

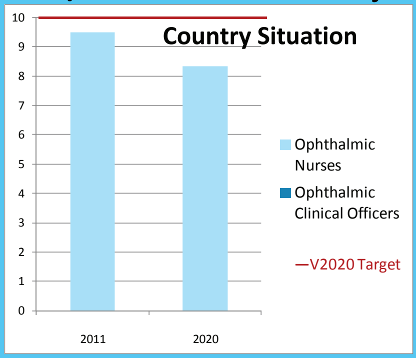

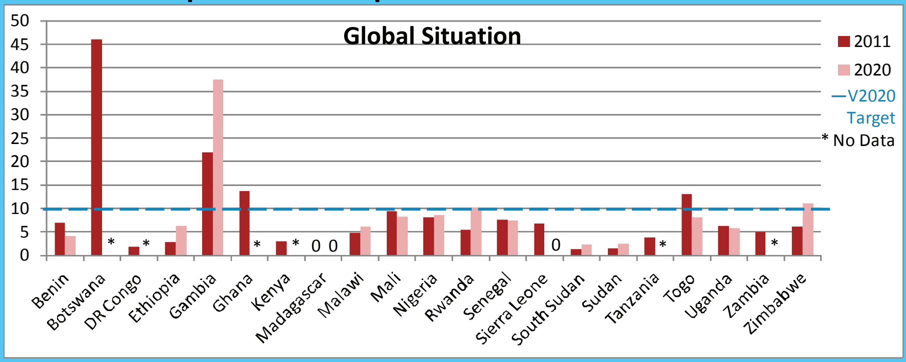

Practitioner Entry vs Exit: The number of ophthalmic nurses is increasing. An additional 34 nurses will be needed by 2020 in order to meet the VISION 2020 target.
Practitioner Working Location: Although Mali has currently almost met the VISION 2020 target, with a practitioner to population ratio of 9.5, this is not the case for practitioners based outside the capital, for which the ratio is 4.4, compared with those working inside the capital where the ratio is 44.2.
Practitioner vs Population Growth: The number of ophthalmic nurses is growing at a slower rate than the general population, 14% compared with 30% over this 9-year period, meaning that the practitioner to population ratio is expected to decrease by 2020.

Refractionists: Current & Projected Workforce per Million Population

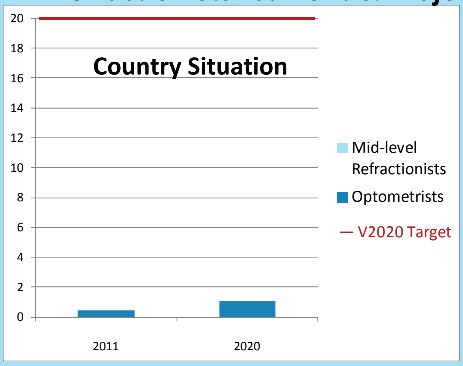

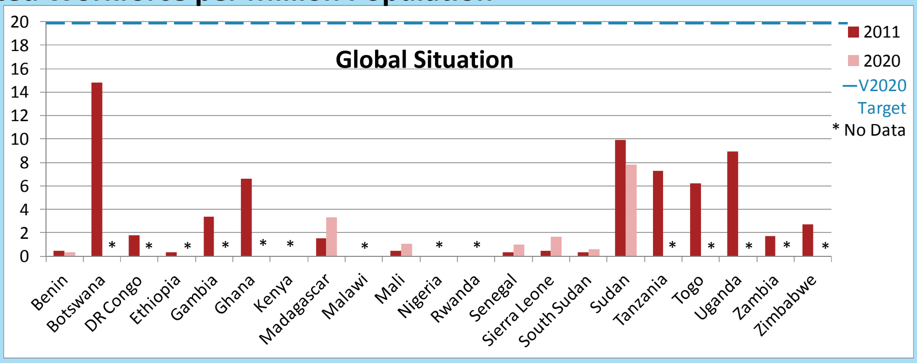

Practitioner Entry vs Exit: Over the past 3 years 5 optometrists have joined the workforce and none have left. This increase in refractionist numbers means that by 2020 Mali will have a practitioner to population ratio of 1.1, still far from the VISION 2020 target of 20.
Practitioner Working Location: All of the 7 optometrists work in the capital, where 15% of the population live.
Practitioner vs Population Growth: Accounting for the projected 30% population growth between 2011 and 2020, Mali will need 411 refractionists to meet the VISION 2020 target; there are currently 7.

Cataract Surgical Performance: Current & Projected Performance per Million Population

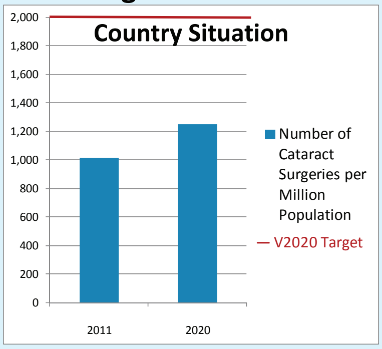

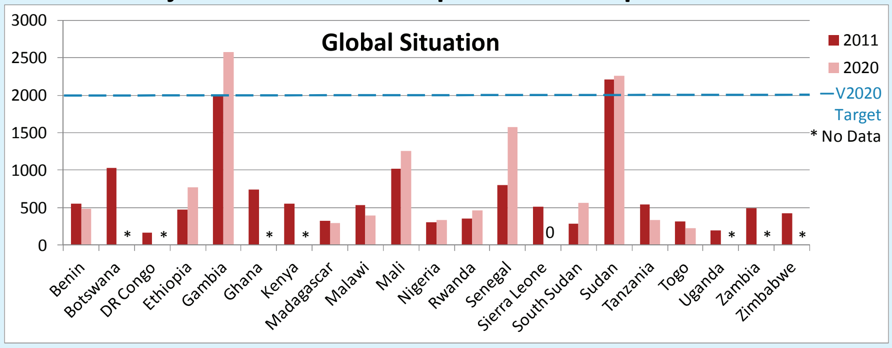

Although the number of cataract surgeries is increasing, the proportional surgeon population increases will eventually be expected to be outpaced by proportional general population increases. Therefore, without additional intervention the target will never be met.

Country Profile: Mali

Current Situation: 2011

Total Population	Population Living in Capital City	% Population Living in Capital	Population Over 50	% Population Over 50
15,821,545	2,037,000	13%	1,217,000	8%

VISION 2020 Targets

Eye Care Cadre	# Practitioners per Million Population
Surgeons	4
OCOs/Nurses	10
Refractionists	20

Characteristics of the Active Eye Care Practitioner Workforce: 2011

Eye Care Cadre	Number of Practitioners in Active Workforce	Sector			Location		# Training Programmes available in Country
		Government	NGO/ Mission	Private for Profit	Capital	Outside Capital	
Ophthalmologists	35	30	0	5	24	11	1
Cataract Surgeons	1	1	0	0	1	0	0
**Surgeons**	**36**	**31**	**0**	**5**	**25**	**11**	**1**
Ophthalmic Clinical Officers	0	0	0	0	0	0	0
Ophthalmic Nurses	150	145	2	3	90	60	1
**OCOs/Nurses**	**150**	**145**	**2**	**3**	**90**	**60**	**1**
Optometrists	7	6	0	1	7	0	1
Mid-level Refractionists	0	0	0	0	0	0	2
**Refractionists**	**7**	**6**	**0**	**1**	**7**	**0**	**3**

Eye Care Practitioner Workforce Dynamics: 2011

Eye Care Cadre	Number of Practitioners in Active Workforce	Practitioners per Million Population			VISION 2020 Country Target # of Practitioners	Shortage in Practitioners to meet Target
		Countrywide	In Capital	Outside Capital		
Ophthalmologists	35	2.2	11.8	0.8		
Cataract Surgeons	1	0.1	0.5	0.0		
**Surgeons**	**36**	**2.3**	**12.3**	**0.8**	**63**	**27**
Ophthalmic Clinical Officers	0	0	0.0	0.0		
Ophthalmic Nurses	150	9.5	44.2	4.4		
**OCOs/Nurses**	**150**	**9.5**	**44.2**	**4.4**	**158**	**8**
Optometrists	7	0.4	3.4	0.0		
Mid-level Refractionists	0	0	0.0	0.0		
**Refractionists**	**7**	**0.4**	**3.4**	**0.0**	**316**	**309**

Annual Cataract Surgical Performance

Number of Cataract Surgeries Performed (data from 2010)	16,073
Number of Cataract Surgeries per Surgeon (surgical performance ratio)	446
% Surgeries Performed by Ophthalmologists (estimate)	100%
Number of Cataract Surgeries per Million Population (CSR)	1,016
Target Number of Cataract Surgeries to meet VISION 2020 Target	31,643
Shortage in Cataract Surgeries to meet VISION 2020 Target	15,570

Projected Situation: 2020

Projected Total Population	Projected Population Living in Capital City	% Projected Population Living in Capital	Projected Population Over 50	% Projected Population Over 50	Expected 9-year Population Increase	Expected 9-year Over 50 Population Increase
20,533,964	3,058,583	15%	1,471,000	7%	30%	21%

Projected Eye Care Practitioner Workforce Dynamics: 2020

Eye Care Cadre	Number of Practitioners in Active Workforce (2011)	Over last 3 years			Projected Net Change over next 9 years	Projected Number of Practitioners in Active Workforce	Projected Practitioners per Million Population			VISION 2020 Country Target # of Practitioners	Projected Shortage in Practitioners to meet VISION 2020
		Number Entered Workforce	Number Exited Workforce	Net Change in Practitioner Numbers			Countrywide	In Capital	Outside Capital		
Ophthalmologists	35	9	2	7	21	56	2.7	12.6	1.0		
Cataract Surgeons	1	0	1	-1	-3	0	0.0	0.0	0.0		
**Surgeons**	**36**	**9**	**3**	**6**	**18**	**56**	**2.7**	**12.6**	**1.0**	**82**	**28**
Ophthalmic Clinical Officers	0	0	0	0	0	0	0.0	0.0	0.0		
Ophthalmic Nurses	150	11	4	7	21	171	8.3	33.5	3.9		
**OCOs/Nurses**	**150**	**11**	**4**	**7**	**21**	**171**	**8.3**	**33.5**	**3.9**	**205**	**34**
Optometrists	7	5	0	5	15	22	1.1	7.2	0.0		
Mid-level Refractionists	0	0	0	0	0	0	0.0	0.0	0.0		
**Refractionists**	**7**	**5**	**0**	**5**	**15**	**22**	**1.1**	**7.2**	**0.0**	**411**	**389**

Annual Projected Cataract Surgical Performance: 2020

Eye Care Cadre	% Surgeries Performed (estimate)	Surgical Performance Ratio per Cadre (2011)	Projected Number of Surgeons in Active Workforce	Projected Number of Cataract Surgeries Performed	Projected Number of Cataract Surgeries per Million Population (CSR)	Target Number of Cataract Surgeries to meet VISION 2020 Target	Projected Shortage in Cataract Surgeries to meet VISION 2020 Target
Ophthalmologists	100	459	56	25,717			
Cataract Surgeons	0	0	0	0			
**Surgeons**	**100%**	**446**	**56**	**25,717**	**1,252**	**41,068**	**15,351**

Country Profile: Nigeria

Key Messages

- **Surgeons:** The current ophthalmologist growth rate is not enough to account for the current overall population growth, meaning that unless trends change, Nigeria will never meet the VISION 2020 target.
- **OCOs/Nurses:** There are no ophthalmic clinical officers working in Nigeria. At the current ophthalmic nurse growth rate, Nigeria will never meet the VISION 2020 target, given the expected population growth rate.
- **Refractionists:** No data was available for this cadre.
- **Cataract Surgeries**: The number of cataract surgeries is increasing at a faster rate than population growth. By 2020 Nigeria will be a third of the way to meeting this target.

VISION 2020 Targets

	Eye Care Practitioners per Million Population			Cataract Surgeries Performed per Million Population	Cataract Surgeries Performed per Surgeon
	Surgeons*	Ophthalmic Clinical Officers /Nurses	Refrac-tionists		
VISION2020 Target	4	10	20	2,000	500
2011 Situation	3.3	8.1	No Data	309	95
On Track					

* For the Africa region this includes Ophthalmologists and Cataract Surgeons

Eye Care Practitioners: % Working Inside/Outside Capital

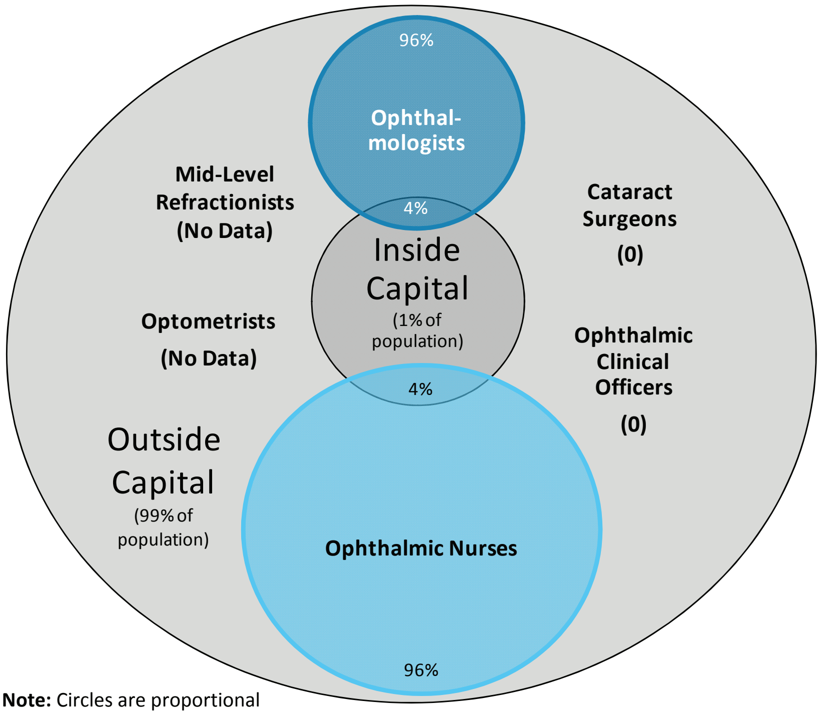

Note: Circles are proportional to numbers of eye care practitioners

In-Country Training Programmes

Surgeons	OCOs/Nurses	Refractionists
No Data	No Data	No Data

Distribution of Practitioners

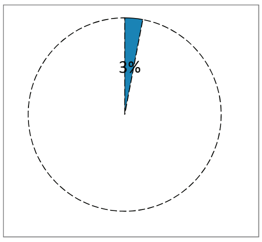

Practitioners based in the capital

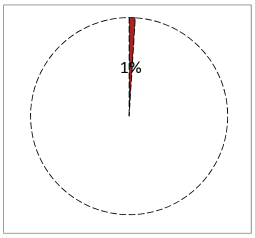

Population living in the capital

3% of all Eye Care Practitioners treat 1% of the population

Eye Care Practitioners: Split between Sectors

Government | NGO/Mission | Private for Profit

Surgeons

Ophthalmologists

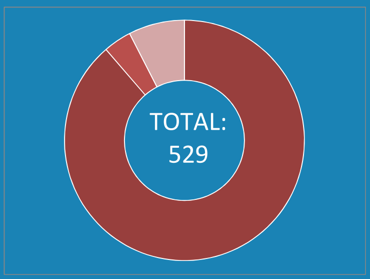

Cataract Surgeons

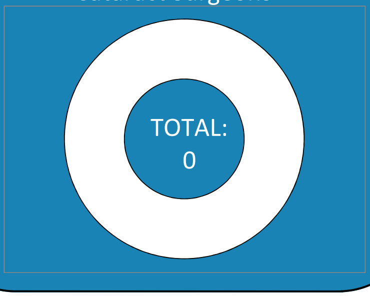

OCOs/Nurses

Ophthalmic Clinical Officers

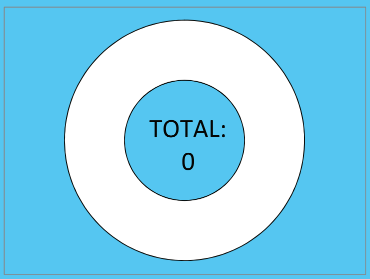

Ophthalmic Nurses

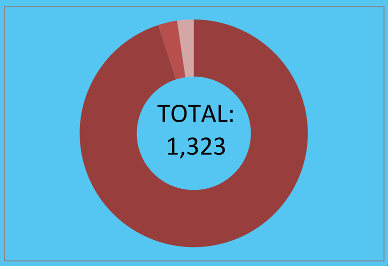

Refractionists

Optometrists

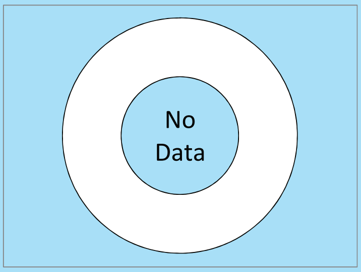

Mid-level Refractionists

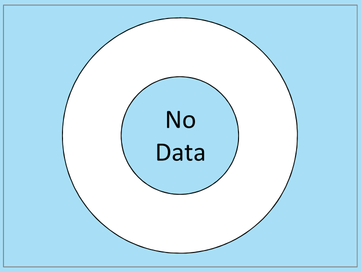

Surgeons: Current & Projected Workforce per Million Population

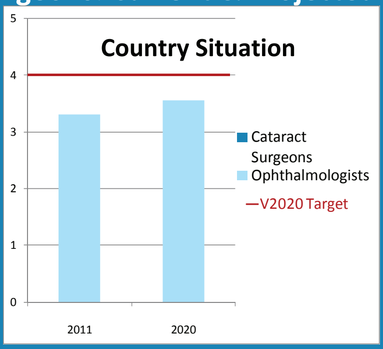

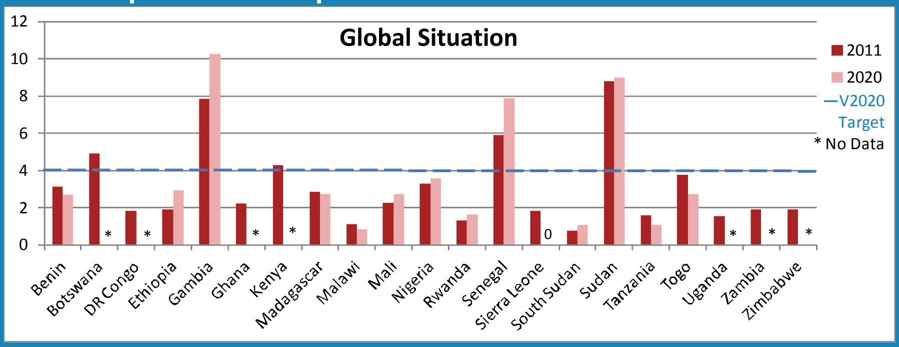

Practitioner Entry vs Exit: Proportional estimates for ophthalmologist entry and exit were used based on data taken from 10 out of 37 states. These estimates project an increasing ophthalmologist workforce.

Practitioner Working Location: 96% of ophthalmologists work outside of Abuja. The surgeon to population ratio is 8.8 for those working in Abuja and 3.2 for those working outside Abuja.

Practitioner vs Population Growth: Nigeria is projected to almost meet the VISION 2020 target by 2020. However, the proportional increases in surgeon numbers will eventually be expected to be outpaced by proportional general population increases. Therefore, without additional intervention the target will never be met.

OCOs/Nurses: Current & Projected Workforce per Million Population

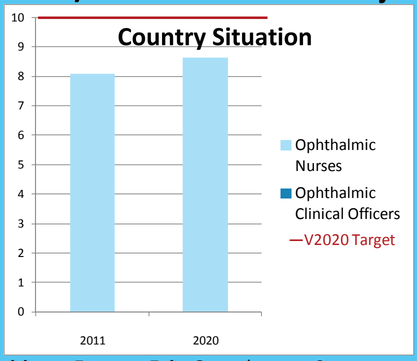

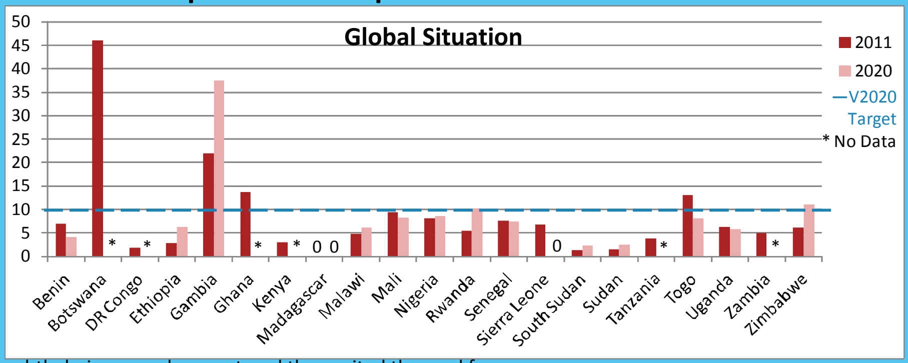

Practitioner Entry vs Exit: Over the past 3 years more ophthalmic nurses have entered than exited the workforce.

Practitioner Working Location: The practitioner to population ratio is 8.1 for the overall population and 20.9 and 8.0 respectively for those working inside and outside the capital.

Practitioner vs Population Growth: Nigeria is projected to be close to the VISION 2020 target by 2020. However, the proportional practitioner population increases will eventually be expected to be outpaced by proportional general population increases. Therefore, without additional intervention the target will never be met.

Refractionists: Current & Projected Workforce per Million Population

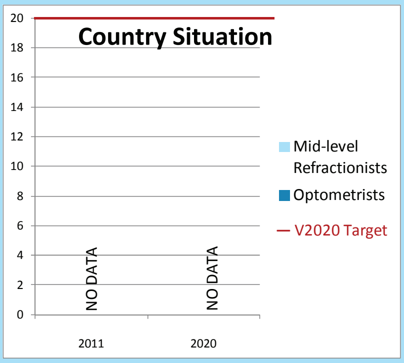

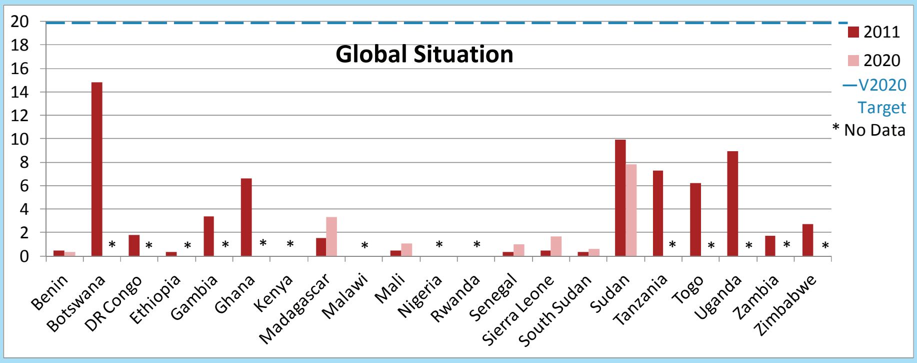

There is no data on the refractionist population in Nigeria.

Cataract Surgical Performance: Current & Projected Performance per Million Population

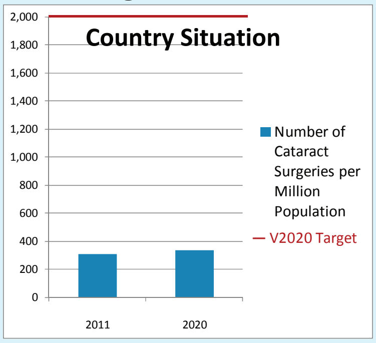

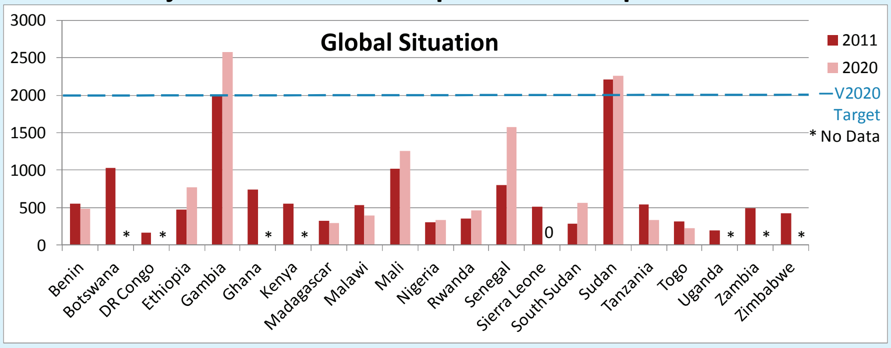

Nigeria needs to carry out more than 8 times as many cataract surgeries each year to meet the VISION 2020 target by 2020.

Country Profile: Nigeria

Current Situation: 2011

Total Population	Population Living in Capital City *	% Population Living in Capital	Population Over 50	% Population Over 50
162,470,261	2,153,000	1%	15,796,000	10%

VISION 2020 Targets

Eye Care Cadre	# Practitioners per Million Population
Surgeons	4
OCOs/Nurses	10
Refractionists	20

Characteristics of the Active Eye Care Practitioner Workforce: 2011

Eye Care Cadre	Number of Practitioners in Active Workforce	Sector			Location		# Training Programmes available in Country
		Government	NGO/ Mission	Private for Profit	Capital	Outside Capital	
Ophthalmologists	529	469	20	40	19	510	ND
Cataract Surgeons	0	0	0	0	0	0	ND
**Surgeons**	**529**	**469**	**20**	**40**	**19**	**510**	**ND**
Ophthalmic Clinical Officers	0	0	0	0	0	0	ND
Ophthalmic Nurses	1,323	1,256	36	31	45	1,278	ND
**OCOs/Nurses**	**1,323**	**1,256**	**36**	**31**	**45**	**1,278**	**ND**
Optometrists	ND	ND	ND	ND	ND	ND	ND
Mid-level Refractionists	ND	ND	ND	ND	ND	ND	ND
**Refractionists**	**ND**	**ND**	**ND**	**ND**	**ND**	**ND**	**ND**

Eye Care Practitioner Workforce Dynamics: 2011

Eye Care Cadre	Number of Practitioners in Active Workforce	Practitioners per Million Population			VISION 2020 Country Target # of Practitioners	Shortage in Practitioners to meet Target
		Countrywide	In Capital	Outside Capital		
Ophthalmologists	529	3.3	8.8	3.2		
Cataract Surgeons	0	0	0.0	0.0		
**Surgeons**	**529**	**3.3**	**8.8**	**3.2**	**650**	**121**
Ophthalmic Clinical Officers	0	0	0.0	0.0		
Ophthalmic Nurses	1,323	8.1	20.9	8.0		
**OCOs/Nurses**	**1,323**	**8.1**	**20.9**	**8.0**	**1,625**	**302**
Optometrists	ND	ND	ND	ND		
Mid-level Refractionists	ND	ND	ND	ND		
**Refractionists**	**ND**	**ND**	**ND**	**ND**	**3,249**	**ND**

Annual Cataract Surgical Performance

Number of Cataract Surgeries Performed (data from 2011)**	48,962
Number of Cataract Surgeries per Surgeon (surgical performance ratio)	95
% Surgeries Performed by Ophthalmologists (estimate)	100%
Number of Cataract Surgeries per Million Population (CSR)	309
Target Number of Cataract Surgeries to meet VISION 2020 Target	324,941
Shortage in Cataract Surgeries to meet VISION 2020 Target	274,728

* Abuja figures were used for "Capital City"

** National Estimates of 2011 Cataract Surgery Performance based on Proportional Data from 13/37 States

ND: No Data

Projected Situation: 2020

Projected Total Population	Projected Population Living in Capital City	% Projected Population Living in Capital	Projected Population Over 50	% Projected Population Over 50	Expected 9-year Population Increase	Expected 9-year Over 50 Population Increase
203,877,920	2,978,549	1%	19,930,000	10%	25%	26%

Projected Eye Care Practitioner Workforce Dynamics: 2020

Eye Care Cadre	Number of Practitioners in Active Workforce (2011)	Over last 3 years **			Projected Net Change over next 9 years	Projected Number of Practitioners in Active Workforce	Projected Practitioners per Million Population			VISION 2020 Country Target # of Practitioners	Projected Shortage in Practitioners to meet VISION 2020
		Number Entered Workforce	Number Exited Workforce	Net Change in Practitioner Numbers			Countrywide	In Capital	Outside Capital		
Ophthalmologists	529	89	24	65	195	724	3.6	8.7	3.5		
Cataract Surgeons	0	0	0	0	0	0	0.0	0.0	0.0		
**Surgeons**	**529**	**89**	**24**	**65**	**195**	**724**	**3.6**	**8.7**	**3.5**	**816**	**92**
Ophthalmic Clinical Officers	0	0	0	0	0	0	0.0	0.0	0.0		
Ophthalmic Nurses	1,323	222	76	146	439	1,762	8.6	20.1	8.5		
**OCOs/Nurses**	**1,323**	**222**	**76**	**146**	**439**	**1,762**	**8.6**	**20.1**	**8.5**	**2,039**	**277**
Optometrists	ND	ND	ND	ND	ND	ND	ND	ND	ND		
Mid-level Refractionists	ND	ND	ND	ND	ND	ND	ND	ND	ND		
**Refractionists**	**ND**	**ND**	**ND**	**ND**	**ND**	**ND**	**ND**	**ND**	**ND**	**4,078**	**ND**

Annual Projected Cataract Surgical Performance: 2020 ***

Eye Care Cadre	% Surgeries Performed (estimate)	Surgical Performance Ratio per Cadre (2011)	Projected Number of Surgeons in Active Workforce	Projected Number of Cataract Surgeries Performed	Projected Number of Cataract Surgeries per Million Population (CSR)	Target Number of Cataract Surgeries to meet VISION 2020 Target	Projected Shortage in Cataract Surgeries to meet VISION 2020 Target
Ophthalmologists	100%	93	724	67,015			
Cataract Surgeons	0	0	0	0			
**Surgeons**	**100%**	**93**	**724**	**67,015**	**337**	**407,756**	**339,029**

** Entry and Exit data for Ophthalmologists and Optometrists are proportional estimates based on data taken from 10 out 37 States ND: No Data

*** Projected National Estimates of 2020 Cataract Surgery Performance also based on Proportional Workforce Net Change from 10/37 States

Country Profile: Rwanda

Key Messages

- **Surgeons:** The current ophthalmologist growth rate is not enough to account for the current overall population growth. Unless trends change, Rwanda will never meet this target.
- **OCOs/Nurses:** Rwanda is currently half-way to meeting the target and with the current increasing growth rate of ophthalmic clinical officers, the target is set to be met by 2020.
- **Refractionists:** Rwanda has 6 optometrists, all working in the private sector in Kigali. There is no information on mid-level refractionists.
- **Cataract Surgeries:** The number of cataract surgeries is increasing at a faster rate than population growth; by 2020 Rwanda will be a quarter of the way to meeting this target.

VISION 2020 Targets

	Eye Care Practitioners per Million Population			Cataract Surgeries Performed per Million Population	Cataract Surgeries Performed per Surgeon
	Surgeons*	Ophthalmic Clinical Officers /Nurses	Refractionists		
VISION2020 Target	4	10	20	2,000	500
2011 Situation	1.3	5.4	No Data	358	279
On Track					

* For the Africa region this includes Ophthalmologists and Cataract Surgeons

Eye Care Practitioners: % Working Inside/Outside Capital

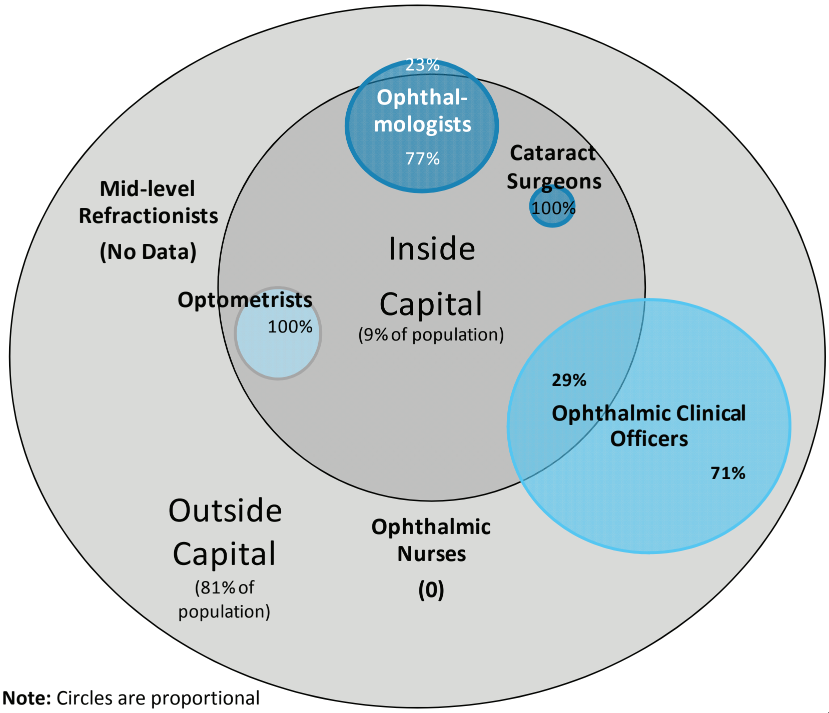

Note: Circles are proportional to numbers of eye care practitioners

In-Country Training Programmes

Surgeons	OCOs/Nurses	Refractionists
Total: 1	Total: 1	No Data

Distribution of Surgeons

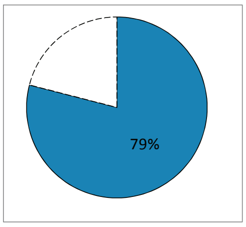

Surgeons based in the capital

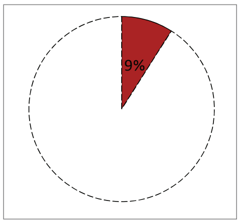

Population living in the capital

79% of Surgeons treat 9% of the population

Eye Care Practitioners: Split between Sectors

Government | NGO/Mission | Private for Profit

Surgeons

Ophthalmologists

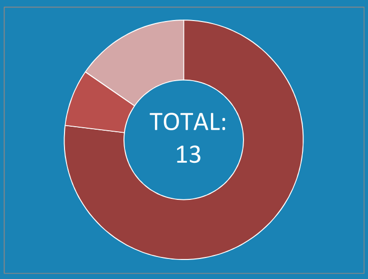

Cataract Surgeons

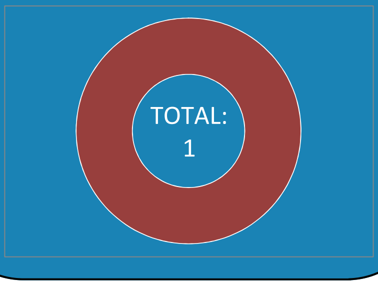

OCOs/Nurses

Ophthalmic Clinical Officers

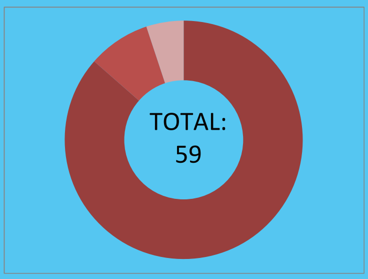

Ophthalmic Nurses

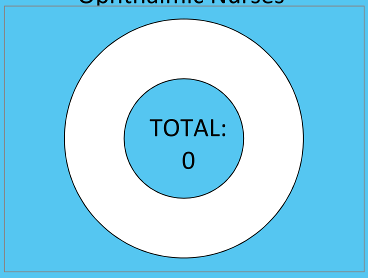

Refractionists

Optometrists

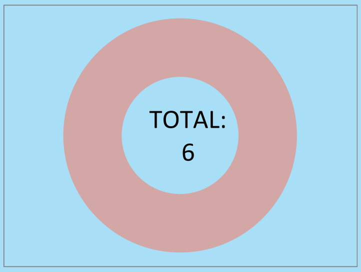

Mid-level Refractionists

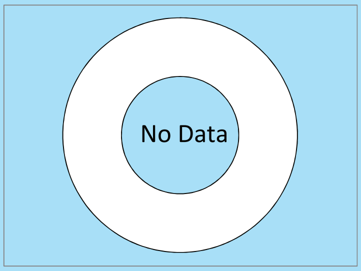

Surgeons: Current & Projected Workforce per Million Population

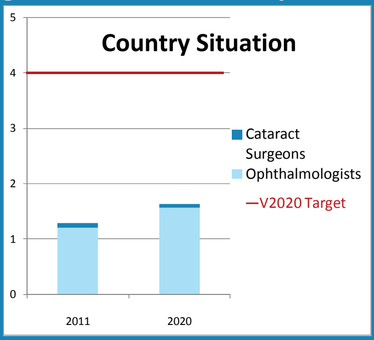

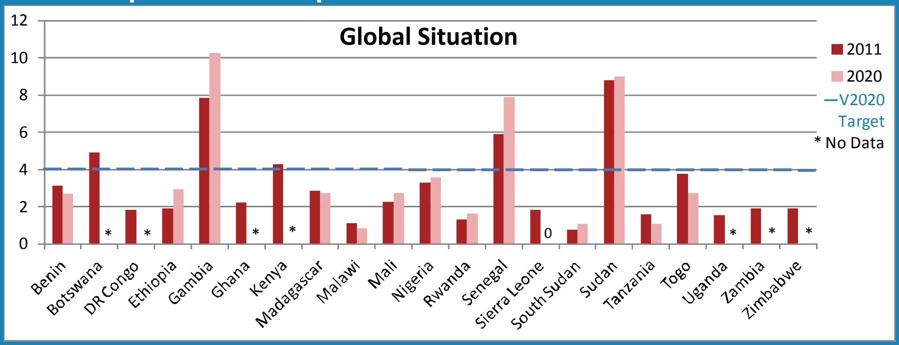

Practitioner Entry vs Exit: Over the past 3 years, 5 ophthalmologists have entered the workforce and 3 have left.
Practitioner Working Location: 80% of surgeons work in the capital. The overall surgeon to population ratio is 1.3. This ratio is 11.0 and 0.3 respectively for those working inside and outside the capital.
Practitioner vs Population Growth: Although the number of surgeons is increasing, the proportional increase in surgeon numbers will eventually be expected to be outpaced by proportional general population increases. Therefore, without additional intervention the target will never be met.

OCOs/Nurses: Current & Projected Workforce per Million Population

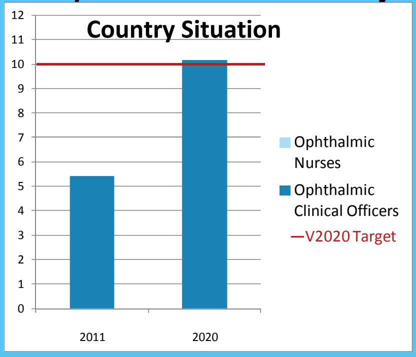

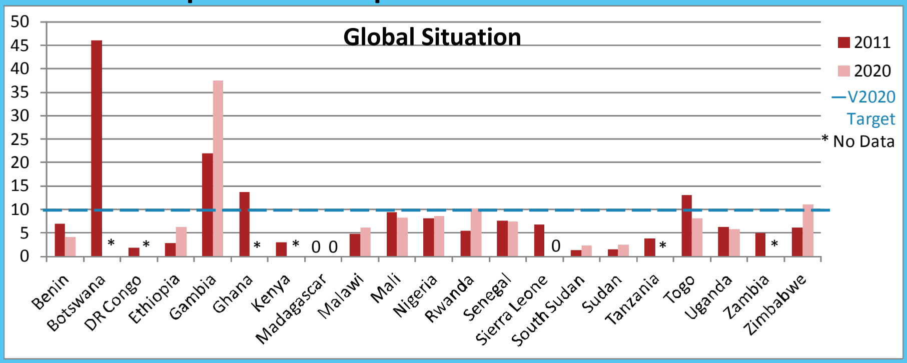

Practitioner Entry vs Exit: Over the past 3 years, 31 ophthalmic clinical officers have entered the workforce and 3 have left.
Practitioner Working Location: Although Rwanda is projected to meet this target, with a projected practitioner to population ratio of 10.2 by 2020, this is not the case for practitioners working outside the capital where the ratio is projected to be 8.1 in 2020, compared with 27.7 for those working inside the capital.
Practitioner vs Population Growth: The increase in practitioner to population ratio, means that Rwanda is projected to meet the VISION 2020 target by 2020.

Refractionists: Current & Projected Workforce per Million Population

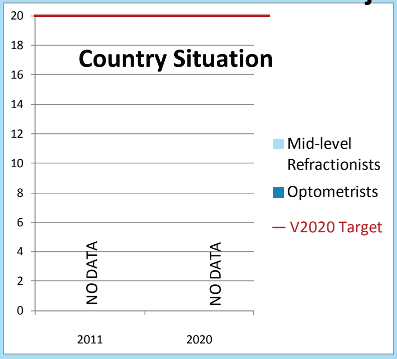

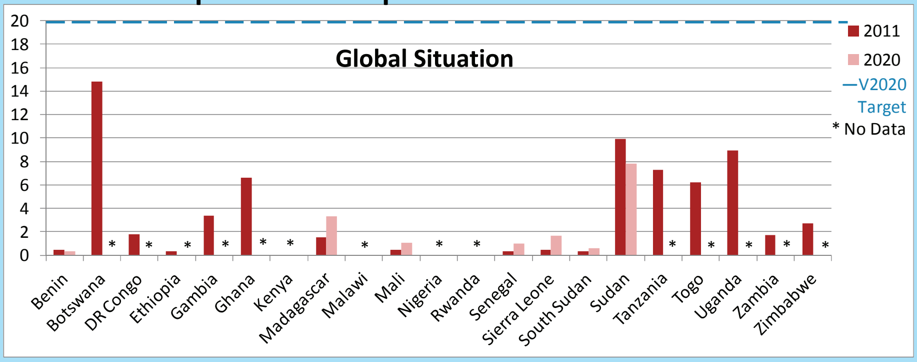

Practitioner Entry vs Exit: Over the past 3 years 2 optometrists have joined the workforce and none have left. There is no information on mid-level refractionists.
Practitioner Working Location: All 6 optometrists work in Kigali and are employed by the private for profit sector.
Practitioner vs Population Growth: The number of optometrists is projected to double by 2020, which is a larger increase than the expected 29% population increase over this period. However, at a projected optometrist to population ratio of 0.85 by 2020 this is far from the target of 20.0.

Cataract Surgical Performance: Current & Projected Performance per Million Population

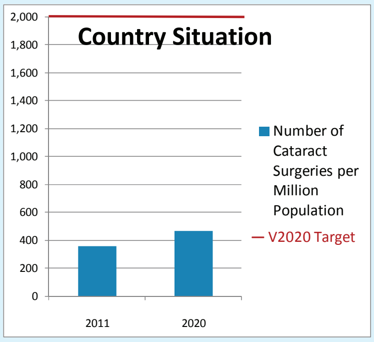

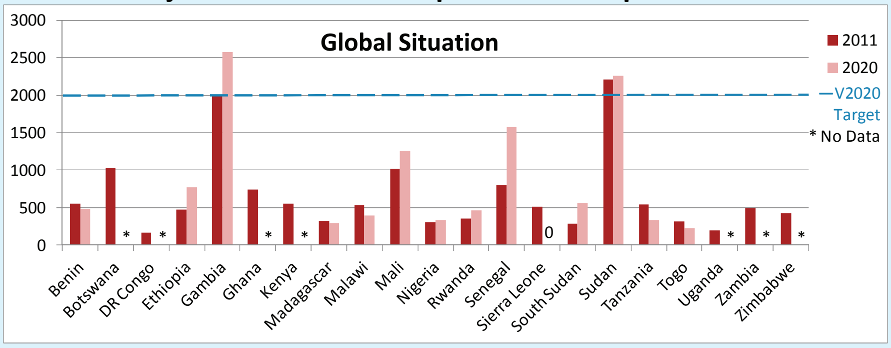

Rwanda is projected to be a quarter of the way to the VISION 2020 target by 2020. Rwanda needs to carry out 7 times as many cataract surgeries each year in order to meet this target by 2020.

Country Profile: Rwanda

Current Situation: 2011

Total Population	Population Living in Capital City	% Population Living in Capital	Population Over 50	% Population Over 50
10,924,703	1,004,000	9%	959,000	9%

VISION 2020 Targets

Eye Care Cadre	# Practitioners per Million Population
Surgeons	4
OCOs/Nurses	10
Refractionists	20

Characteristics of the Active Eye Care Practitioner Workforce: 2011

Eye Care Cadre	Number of Practitioners in Active Workforce	Sector			Location		# Training Programmes available in Country
		Government	NGO/ Mission	Private for Profit	Capital	Outside Capital	
Ophthalmologists	13	10	1	2	10	3	1
Cataract Surgeons	1	1	0	0	1	0	0
**Surgeons**	**14**	**11**	**1**	**2**	**11**	**3**	**1**
Ophthalmic Clinical Officers	59	51	5	3	17	42	1
Ophthalmic Nurses	0	0	0	0	0	0	0
**OCOs/Nurses**	**59**	**51**	**5**	**3**	**17**	**42**	**1**
Optometrists	6	0	0	6	6	0	1
Mid-level Refractionists	ND	ND	ND	ND	ND	ND	ND
**Refractionists**	**ND**	**ND**	**ND**	**ND**	**ND**	**ND**	**ND**

Eye Care Practitioner Workforce Dynamics: 2011

Eye Care Cadre	Number of Practitioners in Active Workforce	Practitioners per Million Population			VISION 2020 Country Target # of Practitioners	Shortage in Practitioners to meet Target
		Countrywide	In Capital	Outside Capital		
Ophthalmologists	13	1.2	10.0	0.3		
Cataract Surgeons	1	0.1	1.0	0.0		
**Surgeons**	**14**	**1.3**	**11.0**	**0.3**	**44**	**30**
Ophthalmic Clinical Officers	59	5.4	16.9	4.2		
Ophthalmic Nurses	0	0.0	0.0	0.0		
**OCOs/Nurses**	**59**	**5.4**	**16.9**	**4.2**	**109**	**50**
Optometrists	6	0.6	6.0	0.0		
Mid-level Refractionists	ND	ND	ND	ND		
**Refractionists**	**ND**	**ND**	**ND**	**ND**	**218**	**ND**

ND: No Data

Annual Cataract Surgical Performance

Number of Cataract Surgeries Performed (data from 2010)	3,910
Number of Cataract Surgeries per Surgeon (surgical performance ratio)	279
% Surgeries Performed by Ophthalmologists (estimate)	99%
Number of Cataract Surgeries per Million Population (CSR)	358
Target Number of Cataract Surgeries to meet VISION 2020 Target	21,849
Shortage in Cataract Surgeries to meet VISION 2020 Target	17,939

Projected Situation: 2020

Projected Total Population	Projected Population Living in Capital City	% Projected Population Living in Capital	Projected Population Over 50	% Projected Population Over 50	Expected 9-year Population Increase	Expected 9-year Over 50 Population Increase
14,044,365	1,484,914	11%	1,409,000	10%	29%	47%

Projected Eye Care Practitioner Workforce Dynamics: 2020

Eye Care Cadre	Number of Practitioners in Active Workforce (2011)	Over last 3 years			Projected Net Change over next 9 years	Projected Number of Practitioners in Active Workforce	Projected Practitioners per Million Population			VISION 2020 Country Target # of Practitioners	Projected Shortage in Practitioners to meet VISION 2020
		Number Entered Workforce	Number Exited Workforce	Net Change in Practitioner Numbers			Countrywide	In Capital	Outside Capital		
Ophthalmologists	13	5	2	3	9	22	1.6	11.4	0.4		
Cataract Surgeons	1	0	0	0	0	1	0.1	0.7	0.0		
**Surgeons**	**14**	**5**	**2**	**3**	**9**	**23**	**1.6**	**12.2**	**0.4**	**56**	**33**
Ophthalmic Clinical Officers	59	31	3	28	84	143	10.2	27.7	8.1		
Ophthalmic Nurses	0	0	0	0	0	0	0.0	0.0	0.0		
**OCOs/Nurses**	**59**	**31**	**3**	**28**	**84**	**143**	**10.2**	**27.7**	**8.1**	**140**	**Target Met**
Optometrists	6	2	0	2	6	12	0.9	8.1	0.0		
Mid-level Refractionists	ND	ND	ND	ND	ND	ND	ND	ND	ND		
**Refractionists**	**ND**	**ND**	**ND**	**ND**	**ND**	**ND**	**ND**	**ND**	**ND**	**281**	**ND**

Annual Projected Cataract Surgical Performance: 2020

Eye Care Cadre	% Surgeries Performed (estimate)	Surgical Performance Ratio per Cadre (2011)	Projected Number of Surgeons in Active Workforce	Projected Number of Cataract Surgeries Performed	Projected Number of Cataract Surgeries per Million Population (CSR)	Target Number of Cataract Surgeries to meet VISION 2020 Target	Projected Shortage in Cataract Surgeries to meet VISION 2020 Target
Ophthalmologists	99%	298	22	6,551			
Cataract Surgeons	1%	39	1	39			
**Surgeons**	**100%**	**279**	**23**	**6,590**	**469**	**28,089**	**21,499**

ND: No Data

Country Profile: Senegal

Key Messages

- **Surgeons:** Senegal is set to meet this VISION 2020 target, largely due to a high projected increase of cataract surgeons.
- **OCOs/Nurses:** The rate of ophthalmic nurses per million population is projected to decrease. Senegal is not set to meet this VISION 2020 target.
- **Refractionists:** Senegal has no optometrists and only 4 mid-level refractionists. To meet this VISION 2020 target, Senegal will need to recruit more than 300 refractionists.
- **Cataract Surgeries:** Cataract surgeries are increasing relative to population growth. If current trends continue, Senegal is set to meet this target by 2028.

VISION 2020 Targets

	Eye Care Practitioners per Million Population			Cataract Surgeries Performed per Million Population	Cataract Surgeries Performed per Surgeon
	Surgeons*	Ophthalmic Clinical Officers /Nurses	Refrac-tionists		
VISION2020 Target	4	10	20	2,000	500
2011 Situation	5.9	7.6	0.3	803	137
On Track					

* For the Africa region this includes Ophthalmologists and Cataract Surgeons

Eye Care Practitioners: % Working Inside/Outside Capital

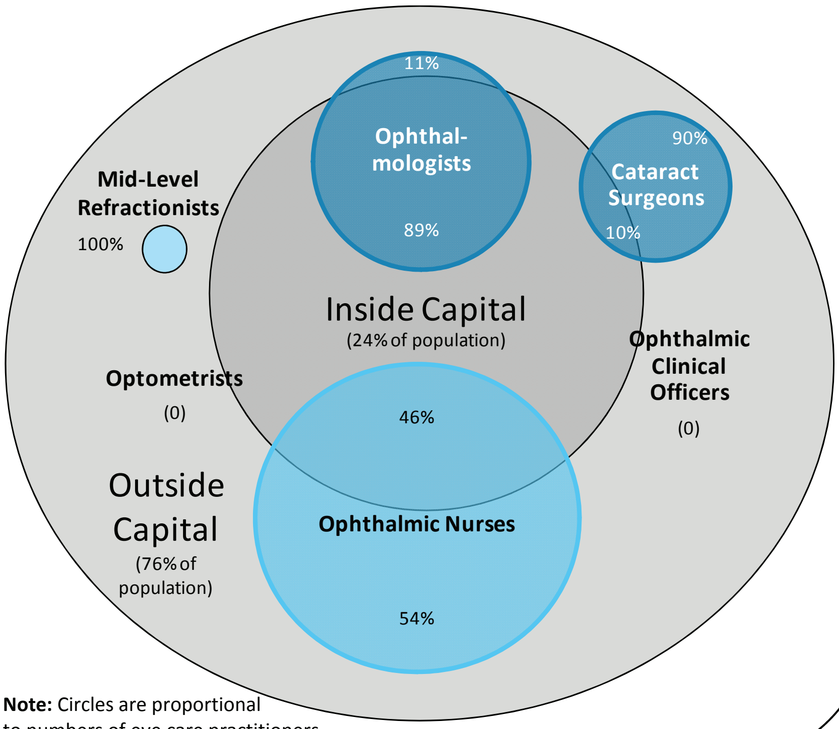

Note: Circles are proportional to numbers of eye care practitioners

In-Country Training Programmes

Surgeons	OCOs/Nurses	Refractionists
Total: 1	Total: 1	Total: 0

Distribution of Ophthalmologists

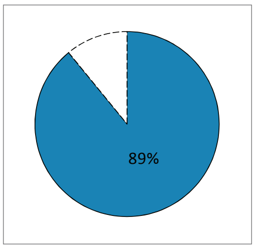

Ophthalmologists based in the capital

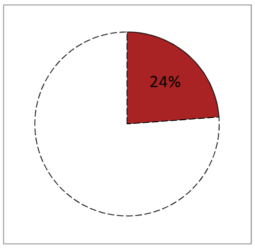

Population living in the capital

89% of Ophthalmologists treat 24% of the population

Eye Care Practitioners: Split between Sectors

Government · NGO/Mission · Private for Profit

Surgeons

Ophthalmologists

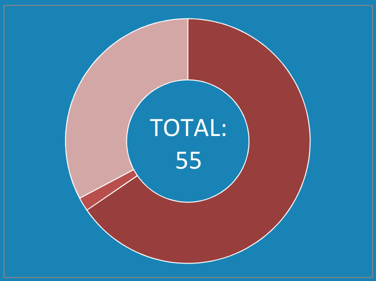

Cataract Surgeons

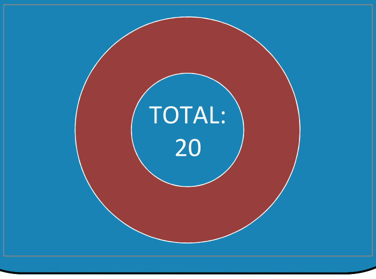

OCOs/Nurses

Ophthalmic Clinical Officers

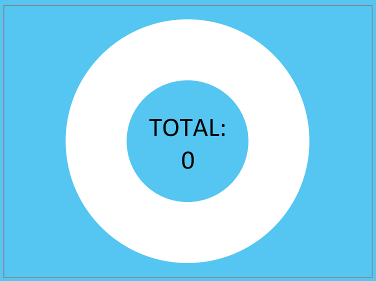

Ophthalmic Nurses

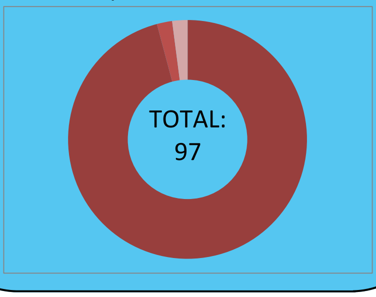

Refractionists

Optometrists

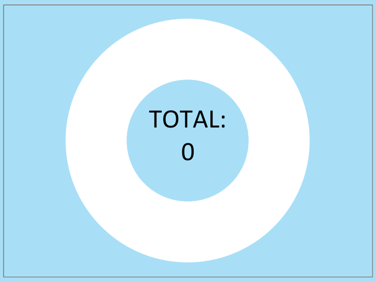

Mid-level Refractionists

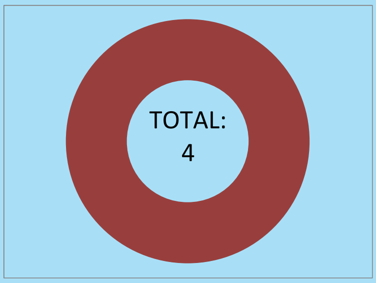

Surgeons: Current & Projected Workforce per Million Population

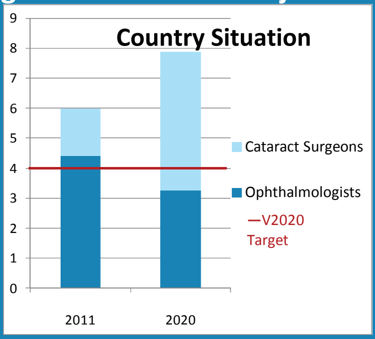

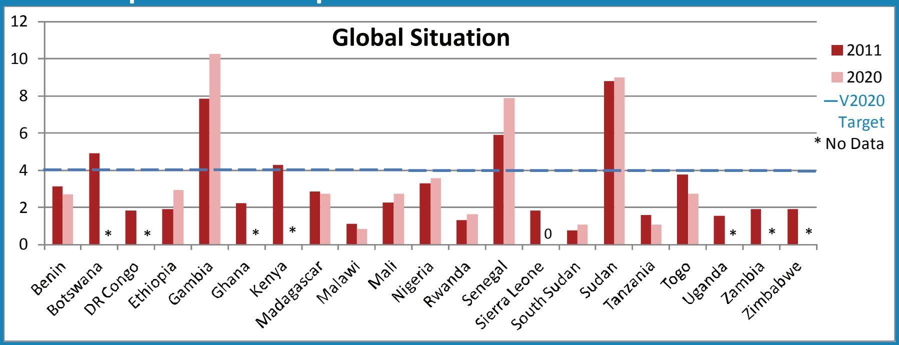

Practitioner Entry vs Exit: Between 2011 and 2020, the number of surgeons is expected to grow and meet the VISION 2020 target, mainly because of a high workforce entry rate of cataract surgeons; ophthalmologists are expected to decrease in numbers over this period.

Practitioner Working Location: Although Senegal meets the VISION 2020 target for practitioner to population ratio of 4, this is not the case for practitioners based outside the capital, for which the ratio is 2.5 and projected to be 3.4 in 2020.

Practitioner vs Population Growth: The number of surgeons is expected to outpace general population growth, mainly due to the high growth rate of cataract surgeons, which is expected to more than triple. The overall surgeon growth rate of 68% is above the general population growth rate of 29%.

OCOs/Nurses: Current & Projected Workforce per Million Population

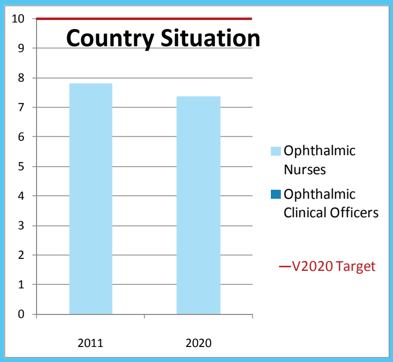

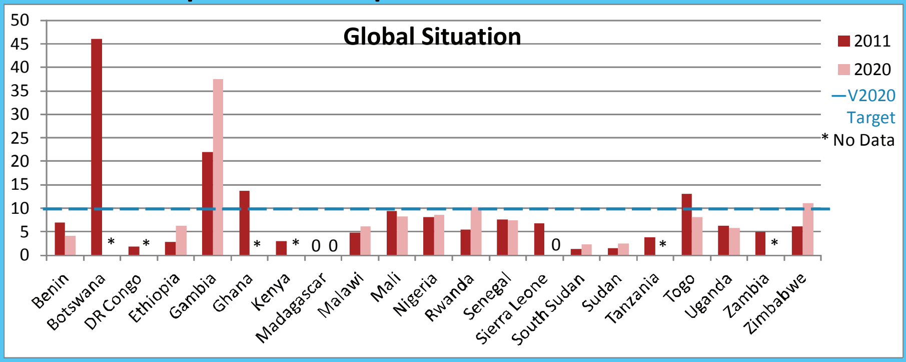

Practitioner Entry vs Exit: Senegal has no ophthalmic clinical officers. Nearly half as many ophthalmic nurses have left the workforce as have entered over the past 3 years.

Practitioner Working Location: The ratio of ophthalmic nurses to population is 5.4 for those working outside the capital, compared to a ratio of 15 inside the capital.

Practitioner vs Population Growth: The projected ophthalmic nurse growth rate is 7%, below the general population growth rate of 29%, meaning that Senegal will be further from the VISION 2020 target by 2020 if trends continue.

Refractionists: Current & Projected Workforce per Million Population

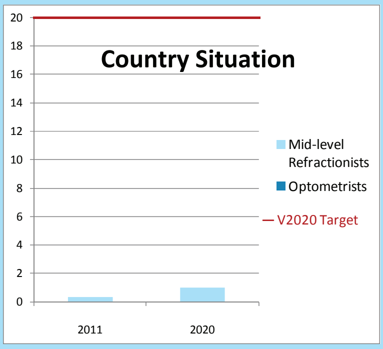

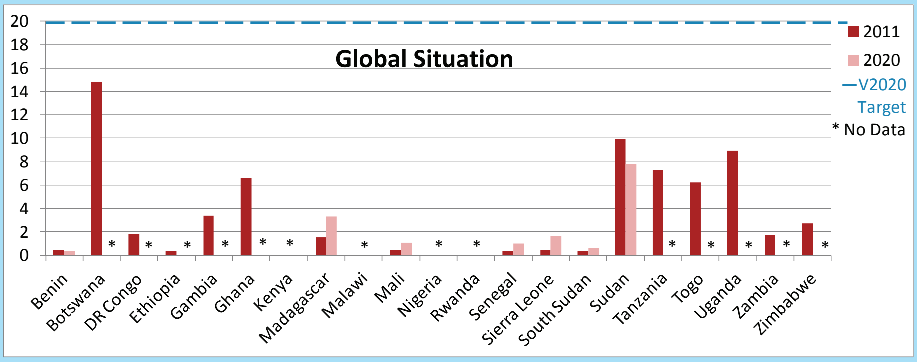

Practitioner Entry vs Exit: Senegal has no optometrists working in the country and only 4 mid-level refractionists. Although the number of mid-level refractionists is expected to quadruple by 2020, this still represents a shortage of 304 refractionists to meet the VISION 2020 target.

Practitioner Working Location: All of the 4 mid-level refractionists are based outside the capital, with a corresponding practitioner to population ratio of 0.4, still far below the VISION 2020 target of 20.

Practitioner vs Population Growth: The overall refractionist practitioner growth rate is 100%, which is above the general population growth rate of 29%, but still far below the 8,000% increase needed.

Cataract Surgical Performance: Current & Projected Performance per Million Population

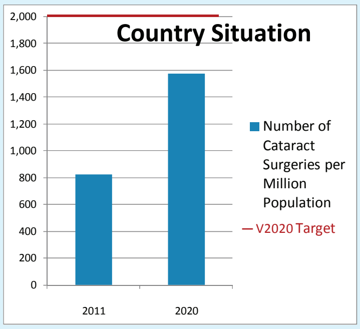

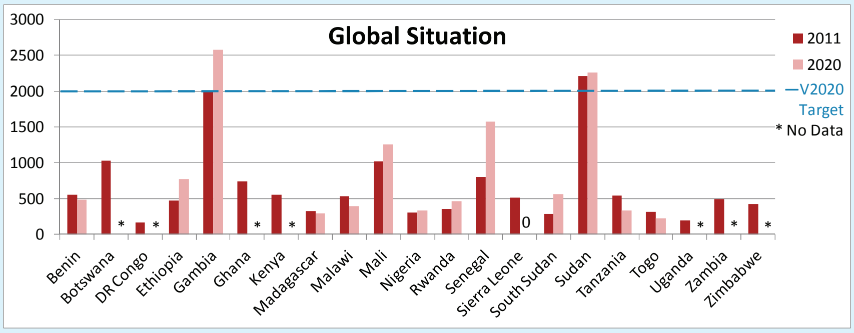

Although Senegal has a projected 96% increase in cataract surgeries by 2020, which is largely due to the projected growth in Cataract Surgeons, this increase is still below the required 149% to reach VISION 2020 target by 2020. If current trends continue, Senegal is expected to meet this target by 2028.

Country Profile: Senegal

Current Situation: 2011

Total Population	Population Living in Capital City	% Population Living in Capital	Population Over 50	% Population Over 50
12,751,652	3,035,000	24%	1,224,000	10%

VISION 2020 Targets

Eye Care Cadre	# Practitioners per Million Population
Surgeons	4
OCOs/Nurses	10
Refractionists	20

Characteristics of the Active Eye Care Practitioner Workforce: 2011

Eye Care Cadre	Number of Practitioners in Active Workforce	Sector			Location		# Training Programmes available in Country
		Government	NGO/ Mission	Private for Profit	Capital	Outside Capital	
Ophthalmologists	55	36	1	18	49	6	1
Cataract Surgeons	20	20	0	0	2	18	0
**Surgeons**	**75**	**56**	**1**	**18**	**51**	**24**	**1**
Ophthalmic Clinical Officers	0	0	0	0	0	0	0
Ophthalmic Nurses	97	93	2	2	45	52	1
**OCOs/Nurses**	**97**	**93**	**2**	**2**	**45**	**52**	**1**
Optometrists	0	0	0	0	0	0	0
Mid-level Refractionists	4	4	0	0	0	4	0
**Refractionists**	**4**	**4**	**0**	**0**	**0**	**4**	**0**

Eye Care Practitioner Workforce Dynamics: 2011

Eye Care Cadre	Number of Practitioners in Active Workforce	Practitioners per Million Population			VISION 2020 Country Target # of Practitioners	Shortage in Practitioners to meet Target
		Countrywide	In Capital	Outside Capital		
Ophthalmologists	55	4.3	16.1	0.6		
Cataract Surgeons	20	1.6	0.7	1.9		
**Surgeons**	**75**	**5.9**	**16.8**	**2.5**	**51**	**Target Met**
Ophthalmic Clinical Officers	0	0.0	0.0	0.0		
Ophthalmic Nurses	97	7.6	14.8	5.4		
**OCOs/Nurses**	**97**	**7.6**	**14.8**	**5.4**	**128**	**31**
Optometrists	0	0.0	0.0	0.0		
Mid-level Refractionists	4	0.3	0.0	0.4		
**Refractionists**	**4**	**0.3**	**0.0**	**0.4**	**255**	**251**

Annual Cataract Surgical Performance

Number of Cataract Surgeries Performed (data from 2010)	10,241
Number of Cataract Surgeries per Surgeon (surgical performance ratio)	137
% Surgeries Performed by Ophthalmologists (estimate)	45%
Number of Cataract Surgeries per Million Population (CSR)	803
Target Number of Cataract Surgeries to meet VISION 2020 Target	25,503
Shortage in Cataract Surgeries to meet VISION 2020 Target	15,262

Projected Situation: 2020

Projected Total Population	Projected Population Living in Capital City	% Projected Population Living in Capital	Projected Population Over 50	% Projected Population Over 50	Expected 9-year Population Increase	Expected 9-year Over 50 Population Increase
16,004,873	4,070,128	25%	1,583,000	10%	26%	29%

Projected Eye Care Practitioner Workforce Dynamics: 2020

Eye Care Cadre	Number of Practitioners in Active Workforce (2011)	Over last 3 years			Projected Net Change over next 9 years	Projected Number of Practitioners in Active Workforce	Projected Practitioners per Million Population			VISION 2020 Country Target # of Practitioners	Projected Shortage in Practitioners to meet VISION 2020
		Number Entered Workforce	Number Exited Workforce	Net Change in Practitioner Numbers			Countrywide	In Capital	Outside Capital		
Ophthalmologists	55	3	4	-1	-3	52	3.3	11.4	0.5		
Cataract Surgeons	20	18	0	18	54	74	4.6	1.8	5.6		
**Surgeons**	**75**	**21**	**4**	**17**	**51**	**126**	**7.9**	**21.1**	**3.4**	**64**	**Target Met**
Ophthalmic Clinical Officers	0	0	0	0	0	0	0.0	0.0	0.0		
Ophthalmic Nurses	97	12	5	7	21	118	7.4	13.4	5.3		
**OCOs/Nurses**	**97**	**12**	**5**	**7**	**21**	**118**	**7.4**	**13.4**	**5.3**	**160**	**42**
Optometrists	0	0	0	0	0	0	0.0	0.0	0.0		
Mid-level Refractionists	4	4	0	4	12	16	1.0	0.0	1.3		
**Refractionists**	**4**	**4**	**0**	**4**	**12**	**16**	**1.0**	**0.0**	**1.3**	**320**	**304**

Annual Projected Cataract Surgical Performance: 2020

Eye Care Cadre	% Surgeries Performed (estimate)	Surgical Performance Ratio per Cadre (2011)	Projected Number of Surgeons in Active Workforce	Projected Number of Cataract Surgeries Performed	Projected Number of Cataract Surgeries per Million Population (CSR)	Target Number of Cataract Surgeries to meet VISION 2020 Target	Projected Shortage in Cataract Surgeries to meet VISION 2020 Target
Ophthalmologists	45%	84	52	4,357			
Cataract Surgeons	55%	282	74	20,840			
**Surgeons**	**100%**	**137**	**126**	**25,198**	**1,574**	**32,010**	**6,812**

Country Profile: Sierra Leone

Key Messages

- **Surgeons:** More surgeons are exiting the workforce than entering. If this trend does not change, there will be no surgeons working in Sierra Leone by 2020.
- **OCOs/Nurses:** There are more nurses exiting the workforce than entering. If this trend continues, there will be no ophthalmic nurses working in Sierra Leone by 2020.
- **Refractionists:** There are no optometrists currently working in Sierra Leone. The mid-level refractionist workforce is increasing, but the country needs to recruit 140 to meet the target.
- **Cataract Surgeries:** Sierra Leone is a quarter of the way to meeting this target. With the decreasing surgeon numbers, there are projected to be no cataract surgeries by 2020.

VISION 2020 Targets

	Eye Care Practitioners per Million Population			Cataract Surgeries Performed per Million Population	Cataract Surgeries Performed per Surgeon
	Surgeons*	Ophthalmic Clinical Officers /Nurses	Refractionists		
VISION2020 Target	4	10	20	2,000	500
2011 Situation	1.8	6.8	0.5	509	277
On Track					

* For the Africa region this includes Ophthalmologists and Cataract Surgeons

Eye Care Practitioners: % Working Inside/Outside Capital

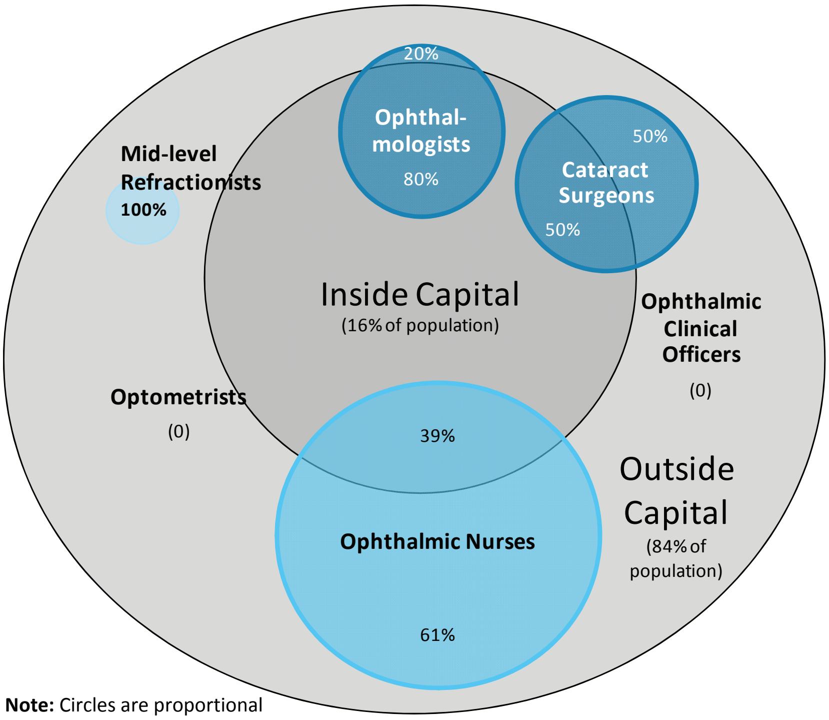

Note: Circles are proportional to numbers of eye care practitioners

In-Country Training Programmes

Surgeons	OCOs/Nurses	Refractionists
Total: 0	Total: 2	Total: 1

Distribution of Surgeons

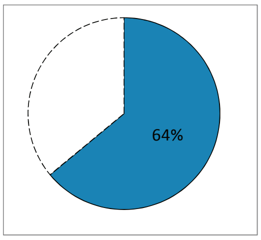

Surgeons based in the capital

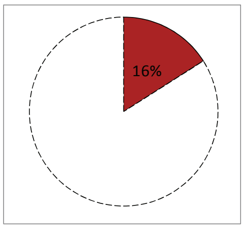

Population living in the capital

64% of Surgeons treat 16% of the population

Eye Care Practitioners: Split between Sectors

Government NGO/Mission Private for Profit

Surgeons

Ophthalmologists

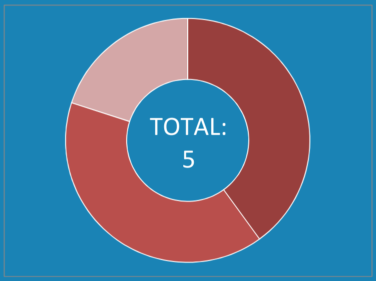

Cataract Surgeons

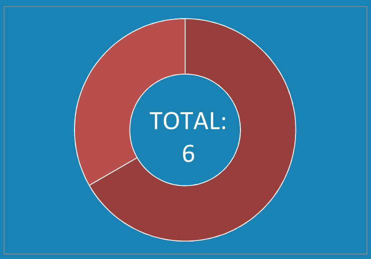

OCOs/Nurses

Ophthalmic Clinical Officers

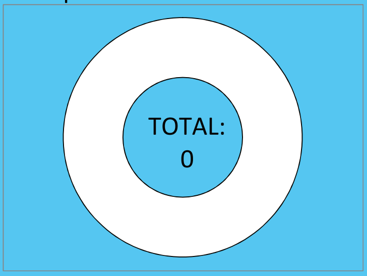

Ophthalmic Nurses

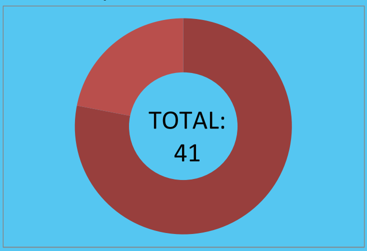

Refractionists

Optometrists

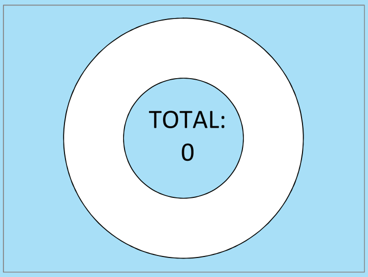

Mid-level Refractionists

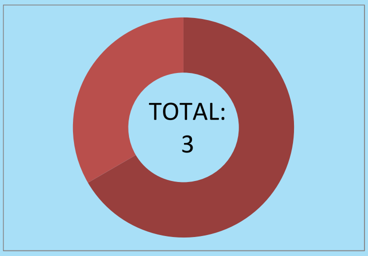

Surgeons: Current & Projected Workforce per Million Population

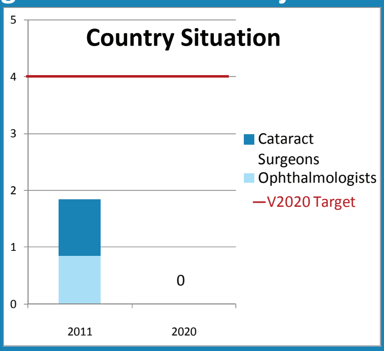

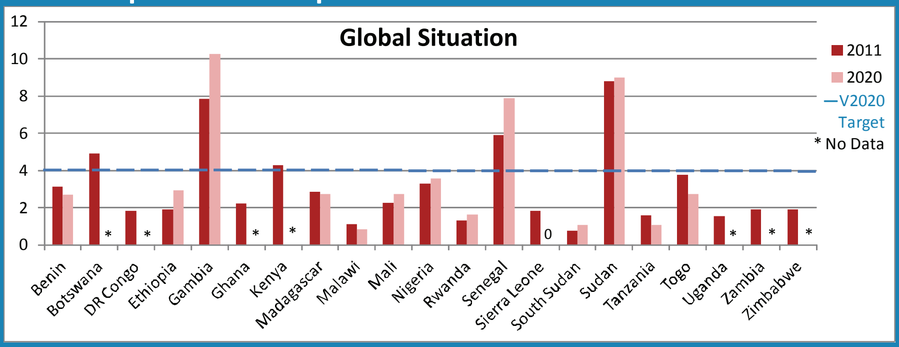

Practitioner Entry vs Exit: Over the past 3 years, more surgeons have exited the workforce than have entered. If this trend continues, Sierra Leone is not expected to have any surgeons in the active workforce by 2020.

Practitioner Working Location: 64% of surgeons work in the capital. The ratio of surgeons to population is 7.4 for those working inside the capital, compared to a ratio of 0.8 outside the capital.

Practitioner vs Population Growth: Surgeon numbers are currently decreasing in Sierra Leone, compared to the population which is projected to increase by 20% between 2011 and 2020.

OCOs/Nurses: Current & Projected Workforce per Million Population

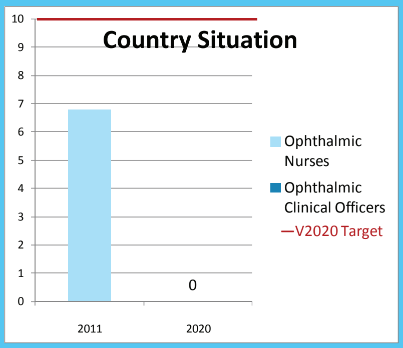

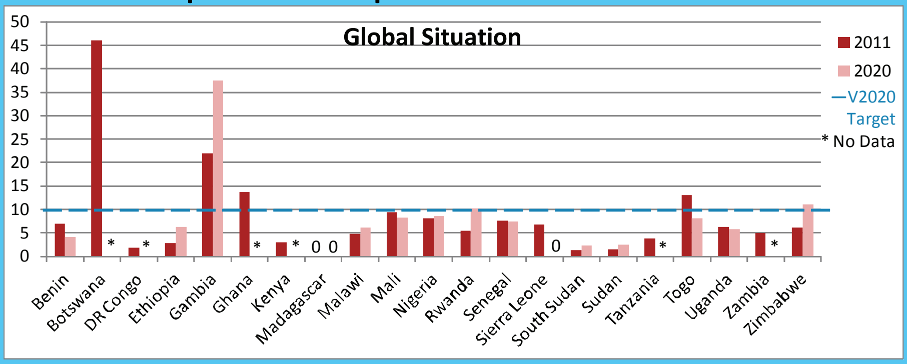

Practitioner Entry vs Exit: There are no ophthalmic clinical officers in Sierra Leone. Over the past 3 years more ophthalmic nurses have exited the workforce than have entered. If this trend continues, Sierra Leone is not expected to have any ophthalmic nurses in the active workforce by 2020.

Practitioner Working Location: The overall practitioner to population ratio is 6.8. This ratio is 17.0 and 5.0 respectively for those working inside and outside the capital.

Practitioner vs Population Growth: At the current decreasing rate, Sierra Leone will have no ophthalmic nurses by 2020, unless current trends change.

Refractionists: Current & Projected Workforce per Million Population

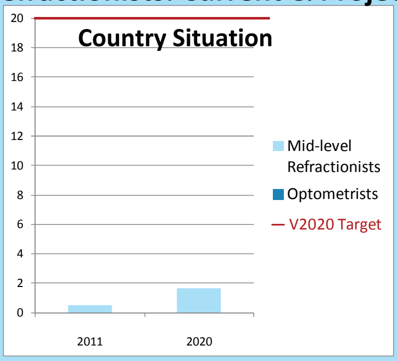

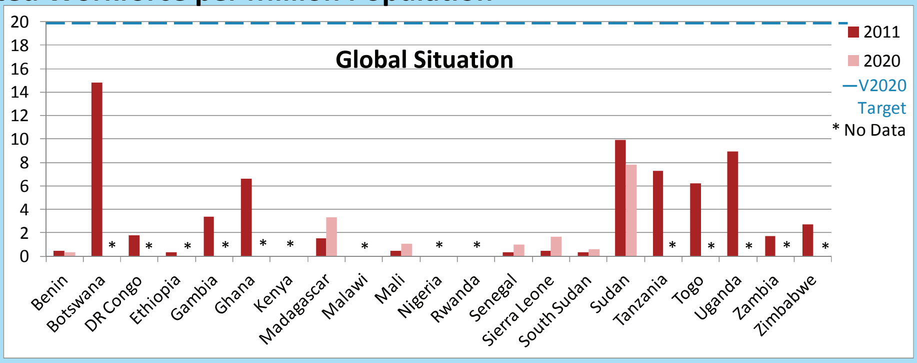

Practitioner Entry vs Exit: There are no optometrists working in Sierra Leone. There are 3 mid-level refractionists in the active workforce and over the past 3 years, 3 have entered and none have exited.

Practitioner Working Location: All 3 mid-level refractionists work outside the capital.

Practitioner vs Population Growth: Although the number of refractionists is increasing at a faster rate than general population growth, in order to meet the VISION 2020 target by 2020, Sierra Leone will need to recruit 141 refractionists.

Cataract Surgical Performance: Current & Projected Performance per Million Population

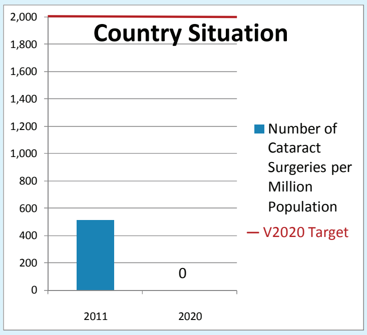

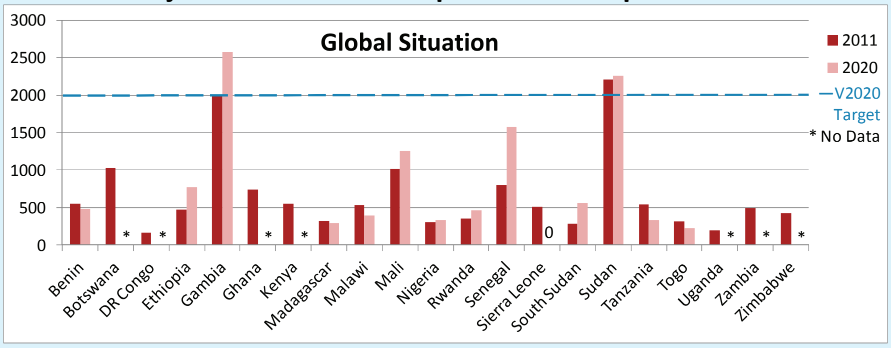

With the decreasing surgeon population, Sierra Leone is not expected to carry out any cataract surgeries by 2020.

Country Profile: Sierra Leone

Current Situation: 2011

Total Population	Population Living in Capital City	% Population Living in Capital	Population Over 50	% Population Over 50
5,987,240	941,000	16%	552,000	9%

VISION 2020 Targets

Eye Care Cadre	# Practitioners per Million Population
Surgeons	4
OCOs/Nurses	10
Refractionists	20

Characteristics of the Active Eye Care Practitioner Workforce: 2011

Eye Care Cadre	Number of Practitioners in Active Workforce	Sector			Location		# Training Programmes available in Country
		Government	NGO/ Mission	Private for Profit	Capital	Outside Capital	
Ophthalmologists	5	2	2	1	4	1	0
Cataract Surgeons	6	4	2	0	3	3	0
**Surgeons**	**11**	**6**	**4**	**1**	**7**	**4**	**0**
Ophthalmic Clinical Officers	0	0	0	0	0	0	0
Ophthalmic Nurses	41	32	9	0	16	25	2
**OCOs/Nurses**	**41**	**32**	**9**	**0**	**16**	**25**	**2**
Optometrists	0	0	0	0	0	0	0
Mid-level Refractionists	3	2	1	0	0	3	1
**Refractionists**	**3**	**2**	**1**	**0**	**0**	**3**	**1**

Eye Care Practitioner Workforce Dynamics: 2011

Eye Care Cadre	Number of Practitioners in Active Workforce	Practitioners per Million Population			VISION 2020 Country Target # of Practitioners	Shortage in Practitioners to meet Target
		Countrywide	In Capital	Outside Capital		
Ophthalmologists	5	0.8	4.3	0.2		
Cataract Surgeons	6	1.0	3.2	0.6		
**Surgeons**	**11**	**1.8**	**7.4**	**0.8**	**24**	**13**
Ophthalmic Clinical Officers	0	0.0	0.0	0.0		
Ophthalmic Nurses	41	6.8	17.0	5.0		
**OCOs/Nurses**	**41**	**6.8**	**17.0**	**5.0**	**60**	**19**
Optometrists	0	0.0	0.0	0.0		
Mid-level Refractionists	3	0.5	0.0	0.6		
**Refractionists**	**3**	**0.5**	**0.0**	**0.6**	**120**	**117**

Annual Cataract Surgical Performance

Number of Cataract Surgeries Performed (data from 2010)	3,049
Number of Cataract Surgeries per Surgeon (surgical performance ratio)	277
% Surgeries Performed by Ophthalmologists (estimate)	ND
Number of Cataract Surgeries per Million Population (CSR)	509
Target Number of Cataract Surgeries to meet VISION 2020 Target	11,974
Shortage in Cataract Surgeries to meet VISION 2020 Target	8,925

ND: No Data

Projected Situation: 2020						
Projected Total Population	Projected Population Living in Capital City	% Projected Population Living in Capital	Projected Population Over 50	% Projected Population Over 50	Expected 9-year Population Increase	Expected 9-year Over 50 Population Increase
7,180,666	1,229,103	17%	663,000	9%	20%	20%

Projected Eye Care Practitioner Workforce Dynamics: 2020											
Eye Care Cadre	Number of Practitioners in Active Workforce (2011)	Over last 3 years			Projected Net Change over next 9 years	Projected Number of Practitioners in Active Workforce	Projected Practitioners per Million Population			VISION 2020 Country Target # of Practitioners	Projected Shortage in Practitioners to meet VISION 2020
		Number Entered Workforce	Number Exited Workforce	Net Change in Practitioner Numbers			Countrywide	In Capital	Outside Capital		
Ophthalmologists	5	1	5	-4	-12	0	0.0	0.0	0.0		
Cataract Surgeons	6	1	6	-5	-15	0	0.0	0.0	0.0		
**Surgeons**	**11**	**2**	**11**	**-9**	**-27**	**0**	**0.0**	**0.0**	**0.0**	**29**	**29**
Ophthalmic Clinical Officers	0	0	0	0	0	0	0.0	0.0	0.0		
Ophthalmic Nurses	41	22	38	-16	-48	0	0.0	0.0	0.0		
**OCOs/Nurses**	**41**	**22**	**38**	**-16**	**-48**	**0**	**0.0**	**0.0**	**0.0**	**72**	**72**
Optometrists	0	0	0	0	0	0	0.0	0.0	0.0		
Mid-level Refractionists	3	3	0	3	9	12	1.7	0.0	2.0		
**Refractionists**	**3**	**3**	**0**	**3**	**9**	**12**	**1.7**	**0.0**	**2.0**	**144**	**132**

Annual Projected Cataract Surgical Performance: 2020							
Eye Care Cadre	% Surgeries Performed (estimate)	Surgical Performance Ratio per Cadre (2011)	Projected Number of Surgeons in Active Workforce	Projected Number of Cataract Surgeries Performed	Projected Number of Cataract Surgeries per Million Population (CSR)	Target Number of Cataract Surgeries to meet VISION 2020 Target	Projected Shortage in Cataract Surgeries to meet VISION 2020 Target
Ophthalmologists	0	0	0	0			
Cataract Surgeons	0	0	0	0			
**Surgeons**	**0**	**0**	**0**	**0**	**0**	**14,361**	**14,361**

Country Profile: Republic of South Sudan

International Centre for Eye Health | AVRI African Vision Research Institute | Sightsavers

Key Messages

- **Surgeons:** The ophthalmologist workforce is decreasing and cataract surgeon numbers increasing. South Sudan will be a quarter of the way to meeting this target by 2020.
- **OCOs/Nurses:** South Sudan is projected to be a quarter of the way to meeting this target by 2020. In order to meet the target 150 practitioners need to be recruited.
- **Refractionists:** There is 1 optometrist and 2 mid-level refractionists currently working in South Sudan. In order to meet the target, South Sudan needs to recruit over 300 of this cadre.
- **Cataract Surgeries:** Cataract surgeries are increasing relative to population growth. However, to meet the VISION 2020 target, these will need to increase by a further 250%.

VISION 2020 Targets

	Eye Care Practitioners per Million Population			Cataract Surgeries Performed per Million Population	Cataract Surgeries Performed per Surgeon
	Surgeons*	Ophthalmic Clinical Officers /Nurses	Refractionists		
VISION2020 Target	4	10	20	2,000	500
2011 Situation	0.8	1.3	0.3	289	377
On Track					

* For the Africa region this includes Ophthalmologists and Cataract Surgeons

Eye Care Practitioners: % Working Inside/Outside Capital

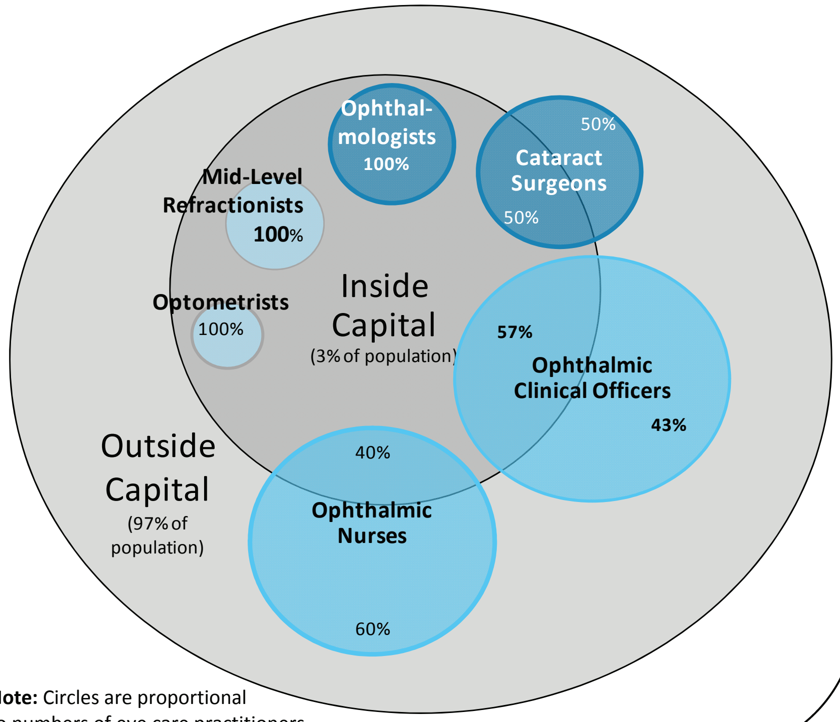

Note: Circles are proportional to numbers of eye care practitioners

In-Country Training Programmes

Surgeons	OCOs/Nurses	Refractionists
Total: 1	Total: 2	Total: 1

Distribution of Surgeons

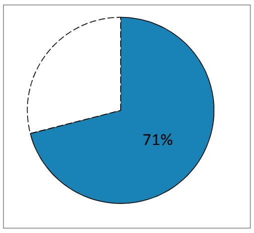

Surgeons based in the capital

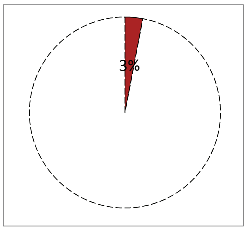

Population living in the capital

71% of Surgeons treat 3% of the population

Eye Care Practitioners: Split between Sectors

Government | NGO/Mission | Private for Profit

Surgeons

Ophthalmologists

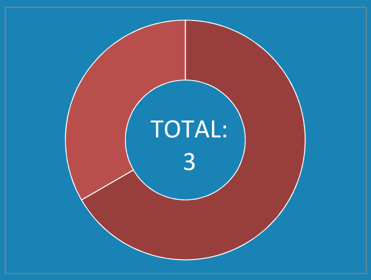

Cataract Surgeons

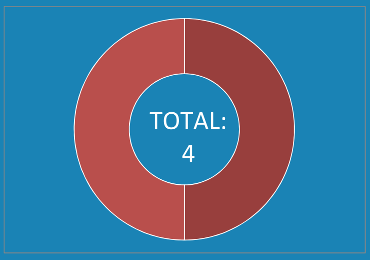

OCOs/Nurses

Ophthalmic Clinical Officers

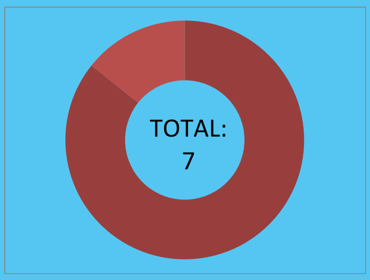

Ophthalmic Nurses

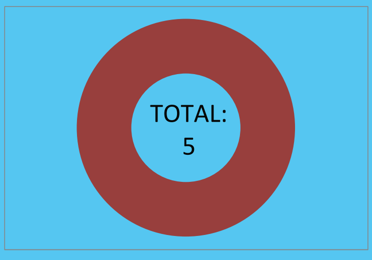

Refractionists

Optometrists

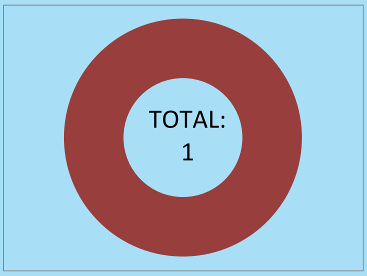

Mid-level Refractionists

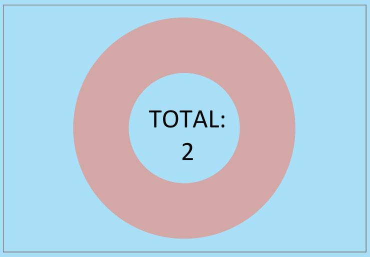

Surgeons: Current & Projected Workforce per Million Population

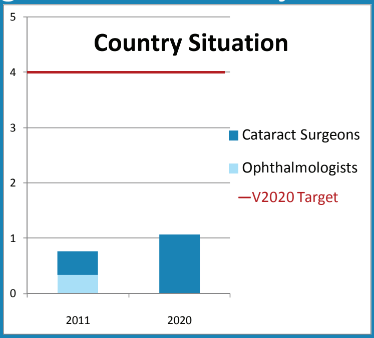

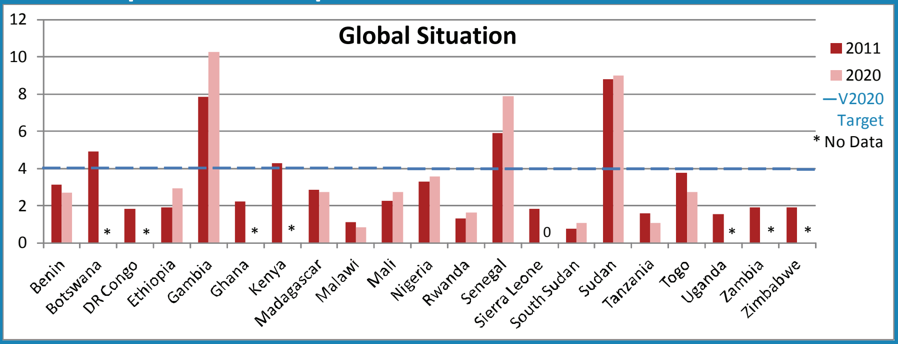

Practitioner Entry vs Exit: Over the past 3 years more ophthalmologists have exited the workforce than have entered and more cataract surgeons have entered the workforce than have exited.

Practitioner Working Location: All of the 3 ophthalmologists in South Sudan work in the capital, as well as half of the 4 cataract surgeons. The overall surgeon to population ratio is 0.8. This ratio is 18.6 and 0.2 respectively for those working inside and outside the capital.

Practitioner vs Population Growth: Although the number of surgeons is increasing faster than the general population, in order to meet the VISION 2020 target by 2020 South Sudan will need to recruit nearly 9 times as many surgeons as are currently in the workforce.

OCOs/Nurses: Current & Projected Workforce per Million Population

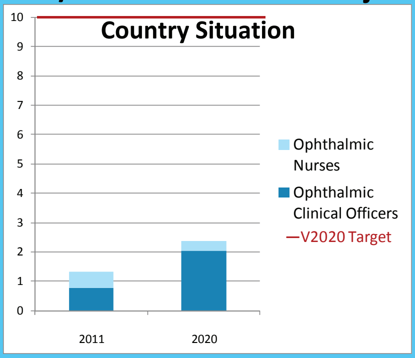

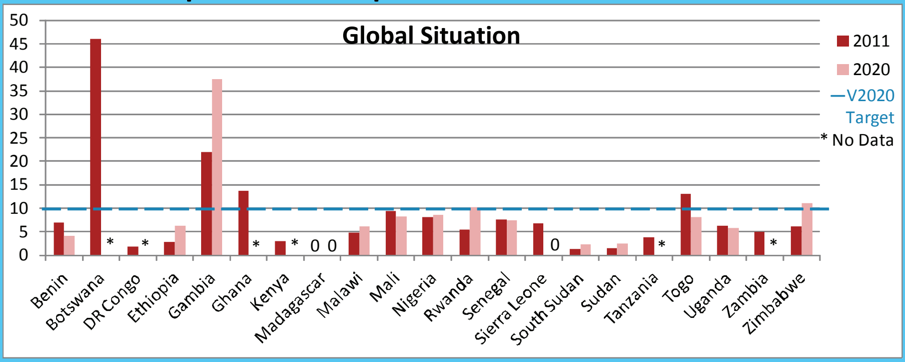

Practitioner Entry vs Exit: Over the past 3 years more ophthalmic clinical officers have entered the workforce than have exited. No ophthalmic nurses have entered or exited.

Practitioner Working Location: The overall practitioner to population ratio is 1.3. This ratio is 22.3 and 0.7 respectively for those working inside and outside the capital.

Practitioner vs Population Growth: Although the practitioner population is growing faster than population growth, by 2020 South Sudan will only be a quarter of the way to meeting this target and will need nearly 13 times as large a workforce in order to meet the target.

Refractionists: Current & Projected Workforce per Million Population

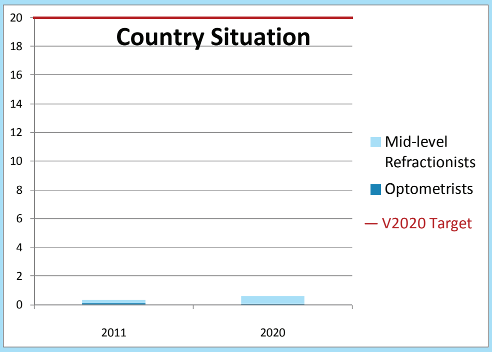

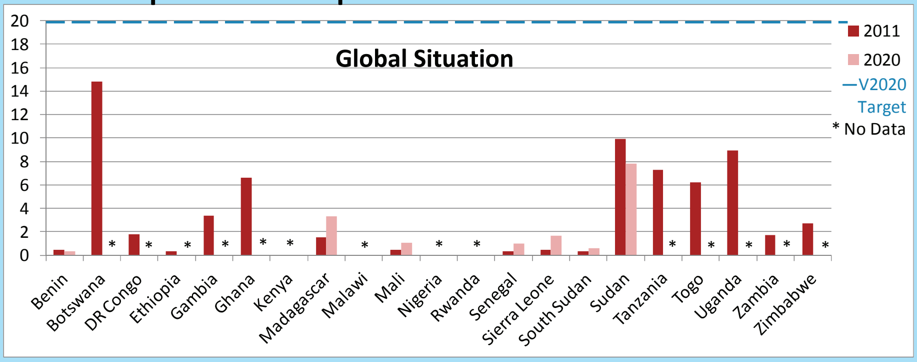

Practitioner Entry vs Exit: South Sudan has 1 optometrist and 2 mid-level refractionists. Over the past 3 years there has been no change in optometrist numbers and 2 mid-level refractionists have entered the workforce. South Sudan therefore has a projected growth rate of less than one additional refractionist each year.

Practitioner Working Location: All 3 refractionists work in the capital. The optometrist is employed by the government and both mid-level refractionists work in the private for profit sector.

Practitioner vs Population Growth: South Sudan currently has 3 refractionists and is projected to have 9 in the active workforce by 2020. In order to meet the VISION 2020 target by 2020, South Sudan need to have 302 refractionists in the active workforce.

Cataract Surgical Performance: Current & Projected Performance per Million Population

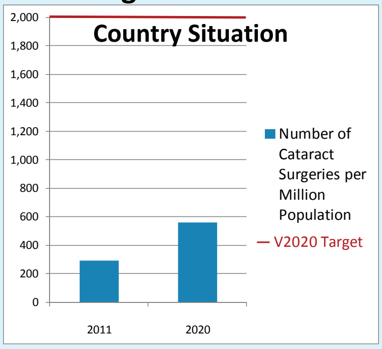

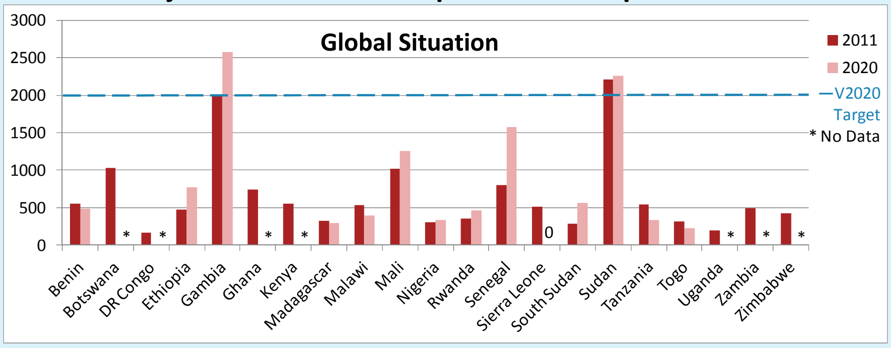

With the increasing number of cataract surgeons, South Sudan is expected to be just over a quarter of the way to meeting this target by 2020. In order to meet the target South Sudan needs to carry out 11 times as many cataract surgeries each year as they currently do.

Country Profile: South Sudan

Current Situation: 2011

Total Population	Population Living in Capital City	% Population Living in Capital	Population Over 50	% Population Over 50
9,135,362	269,000	3%	1,057,000	12%

VISION 2020 Targets

Eye Care Cadre	# Practitioners per Million Population
Surgeons	4
OCOs/Nurses	10
Refractionists	20

Characteristics of the Active Eye Care Practitioner Workforce: 2011

Eye Care Cadre	Number of Practitioners in Active Workforce	Sector			Location		# Training Programmes available in Country
		Government	NGO/ Mission	Private for Profit	Capital	Outside Capital	
Ophthalmologists	3	2	1	0	3	0	0
Cataract Surgeons	4	2	2	0	2	2	1
**Surgeons**	**7**	**4**	**3**	**0**	**5**	**2**	**1**
Ophthalmic Clinical Officers	7	6	1	0	4	3	1
Ophthalmic Nurses	5	5	0	0	2	3	1
**OCOs/Nurses**	**12**	**11**	**1**	**0**	**6**	**6**	**2**
Optometrists	1	1	0	0	1	0	0
Mid-level Refractionists	2	0	0	2	2	0	1
**Refractionists**	**3**	**1**	**0**	**2**	**3**	**0**	**1**

Eye Care Practitioner Workforce Dynamics: 2011

Eye Care Cadre	Number of Practitioners in Active Workforce	Practitioners per Million Population			VISION 2020 Country Target # of Practitioners	Shortage in Practitioners to meet Target
		Countrywide	In Capital	Outside Capital		
Ophthalmologists	3	0.3	11.2	0.0		
Cataract Surgeons	4	0.4	7.4	0.2		
**Surgeons**	**7**	**0.8**	**18.6**	**0.2**	**35**	**28**
Ophthalmic Clinical Officers	7	0.8	14.9	0.3		
Ophthalmic Nurses	5	0.5	7.4	0.3		
**OCOs/Nurses**	**12**	**1.3**	**22.3**	**0.7**	**86**	**74**
Optometrists	1	0.1	3.7	0.0		
Mid-level Refractionists	2	0.2	7.4	0.0		
**Refractionists**	**3**	**0.3**	**11.2**	**0.0**	**173**	**170**

Annual Cataract Surgical Performance

Number of Cataract Surgeries Performed (data from 2010)	2,639
Number of Cataract Surgeries per Surgeon (surgical performance ratio)	377
% Surgeries Performed by Ophthalmologists (estimate)	20%
Number of Cataract Surgeries per Million Population (CSR)	289
Target Number of Cataract Surgeries to meet VISION 2020 Target	18,271
Shortage in Cataract Surgeries to meet VISION 2020 Target	15,632

Projected Situation: 2020

Projected Total Population	Projected Population Living in Capital City	% Projected Population Living in Capital	Projected Population Over 50	% Projected Population Over 50	Expected 9-year Population Increase	Expected 9-year Over 50 Population Increase
15,088,901	380,546	3%	1,583,000	10%	65%	50%

Projected Eye Care Practitioner Workforce Dynamics: 2020

Eye Care Cadre	Number of Practitioners in Active Workforce (2011)	Over last 3 years			Projected Net Change over next 9 years	Projected Number of Practitioners in Active Workforce	Projected Practitioners per Million Population			VISION 2020 Country Target # of Practitioners	Projected Shortage in Practitioners to meet VISION 2020 Target
		Number Entered Workforce	Number Exited Workforce	Net Change in Practitioner Numbers			Countrywide	In Capital	Outside Capital		
Ophthalmologists	3	0	2	-2	-6	0	0.0	0.0	0.0		
Cataract Surgeons	4	4	0	4	12	16	1.1	21.0	0.5		
**Surgeons**	**7**	**4**	**2**	**2**	**6**	**16**	**1.1**	**30.0**	**0.3**	**60**	**44**
Ophthalmic Clinical Officers	7	8	0	8	24	31	2.1	46.5	0.9		
Ophthalmic Nurses	5	0	0	0	0	5	0.3	5.3	0.2		
**OCOs/Nurses**	**12**	**8**	**0**	**8**	**24**	**36**	**2.4**	**47.3**	**1.2**	**151**	**115**
Optometrists	1	0	0	0	0	1	0.1	2.6	0.0		
Mid-level Refractionists	2	2	0	2	6	8	0.5	21.0	0.0		
**Refractionists**	**3**	**2**	**0**	**2**	**6**	**9**	**0.6**	**23.7**	**0.0**	**302**	**293**

Annual Projected Cataract Surgical Performance: 2020

Eye Care Cadre	% Surgeries Performed (estimate)	Surgical Performance Ratio per Cadre (2011)	Projected Number of Surgeons in Active Workforce	Projected Number of Cataract Surgeries Performed	Projected Number of Cataract Surgeries per Million Population (CSR)	Target Number of Cataract Surgeries to meet VISION 2020 Target	Projected Shortage in Cataract Surgeries to meet VISION 2020 Target
Ophthalmologists	20%	176	0	0			
Cataract Surgeons	80%	528	16	8,445			
**Surgeons**	**100%**	**377**	**16**	**8,445**	**560**	**30,178**	**21,733**

Country Profile: Sudan

Key Messages

- **Surgeons:** Sudan is well above this VISION 2020 target, which is projected to further increase by 2020.
- **OCOs/Nurses:** By 2020 Sudan is projected to being a quarter of the way towards meeting this target and taking into account general population growth, is never expected to meet this target.
- **Refractionists:** Sudan is currently half-way to meeting this target. As the number of refractionists is set to increase at a slower rate than population growth, Sudan will be further from this target by 2020.
- **Cataract Surgeries:** Sudan is above this target, which is projected to further increase by 2020.

VISION 2020 Targets

	Eye Care Practitioners per Million Population			Cataract Surgeries Performed per Million Population	Cataract Surgeries Performed per Surgeon
	Surgeons*	Ophthalmic Clinical Officers /Nurses	Refraction-ists		
VISION2020 Target	4	10	20	2,000	500
2011 Situation	8.8	1.5	9.9	2,210	252
On Track					

* For the Africa region this includes Ophthalmologists and Cataract Surgeons

Eye Care Practitioners: % Working Inside/Outside Capital

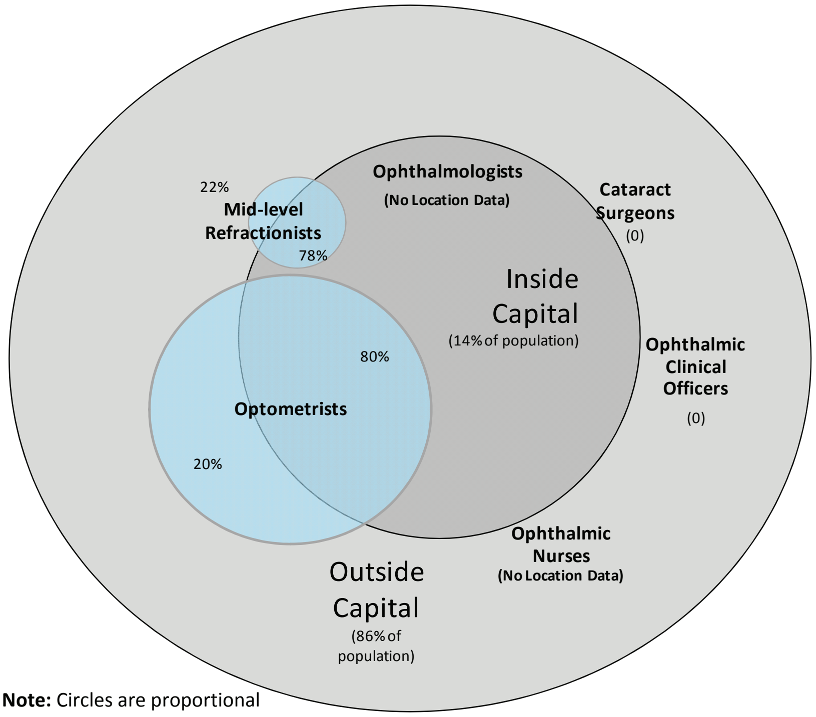

Note: Circles are proportional to numbers of eye care practitioners

In-Country Training Programmes

Surgeons
Total: 8

OCOs/Nurses
Total: 2

Refractionists
Total: 3

Distribution of Refractionists

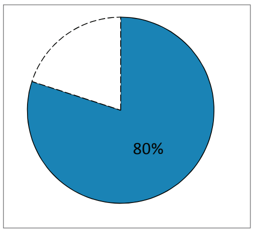

Refractionists based in the capital

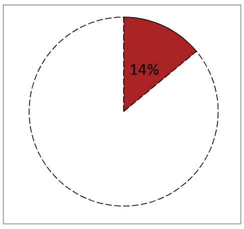

Population living in the capital

80% of all Refractionists treat 14% of the population

Eye Care Practitioners: Split between Sectors

Government | NGO/Mission | Private for Profit

Surgeons

Ophthalmologists

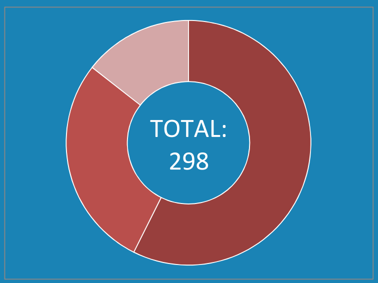

Cataract Surgeons

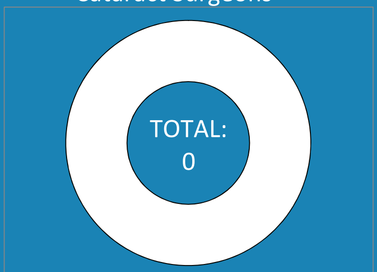

OCOs/Nurses

Ophthalmic Clinical Officers

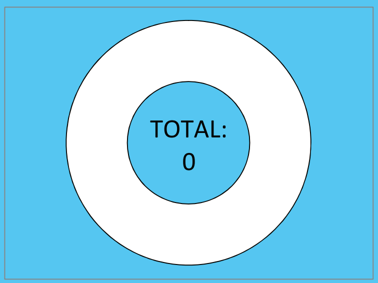

Ophthalmic Nurses

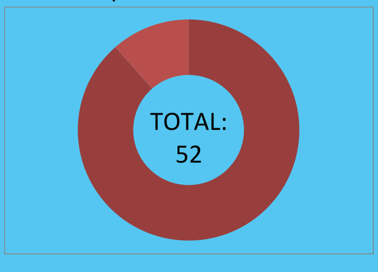

Refractionists

Optometrists

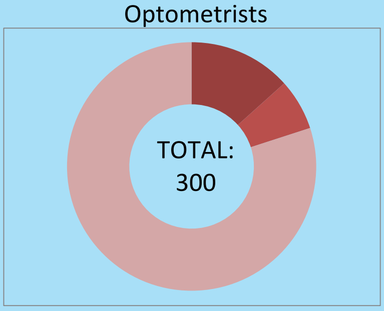

Mid-level Refractionists

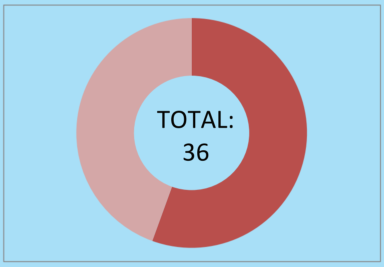

Surgeons: Current & Projected Workforce per Million Population

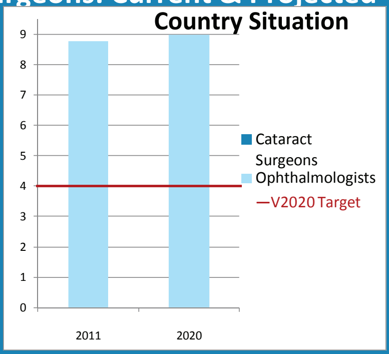

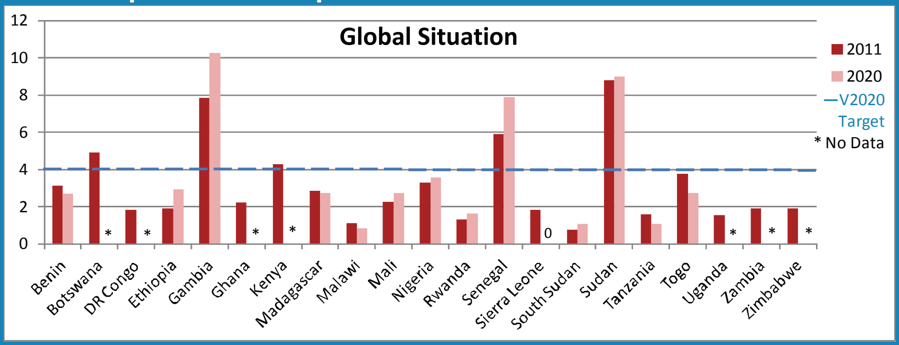

Practitioner Entry vs Exit: Only ophthalmologists work in Sudan. Over the past 3 years, there has been an increase in ophthalmologist numbers.

Practitioner Working Location: There is no location data for ophthalmologists.

Practitioner vs Population Growth: Between 2011 and 2020 there is expected to be a 31% increase in the general population. The ophthalmologist population is expected to increase at a slightly faster rate.

OCOs/Nurses: Current & Projected Workforce per Million Population

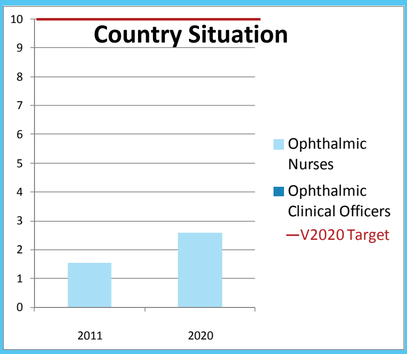

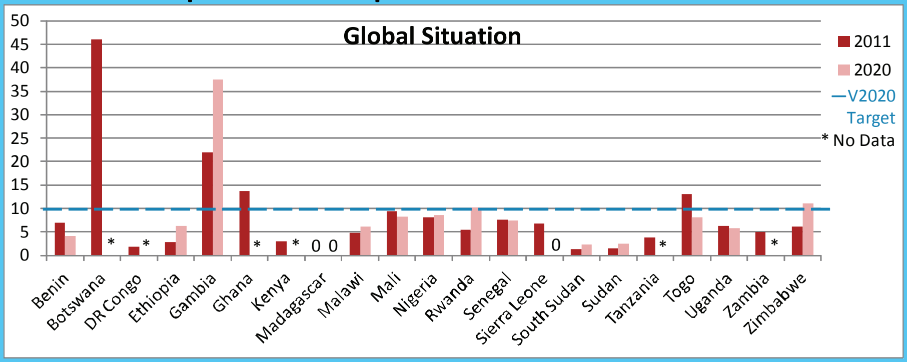

Practitioner Entry vs Exit: There are no ophthalmic clinical officers in Sudan. The number of ophthalmic nurses is expected to more than double between 2011 and 2020.

Practitioner Working Location: There is no location data for ophthalmic nurses. 88% of ophthalmic nurses are employed by the government.

Practitioner vs Population Growth: The number of ophthalmic nurses is expected to increase at a faster rate than the general population, which is expected to increase by 31% between 2011 and 2020.

Refractionists: Current & Projected Workforce per Million Population

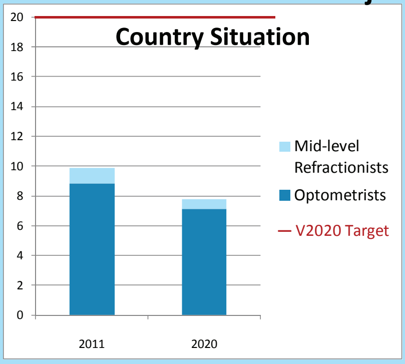

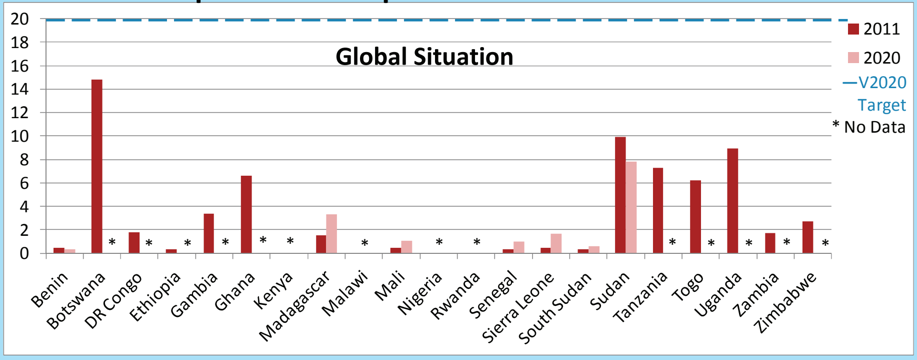

Practitioner Entry vs Exit: Over the past 3 years more mid-level refractionists have exited the workforce than have entered over the past 3 years and 6 more optometrists have entered than exited the workforce.

Practitioner Working Location: Overall Sudan is nearly half way to meeting the VISION 2020 target with a practitioner to population ratio of 9.9. This is not the case for practitioners working outside the capital where the ratio is 2.3, compared with 57.9 for those working inside the capital.

Practitioner vs Population Growth: The general population is expected to grow at a faster rate than the number of practitioners. Sudan is expected to be further from the VISION 2020 target by 2020 unless current trends change.

Cataract Surgical Performance: Current & Projected Performance per Million Population

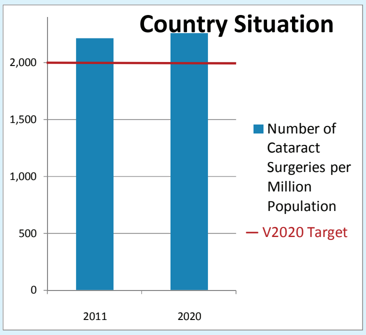

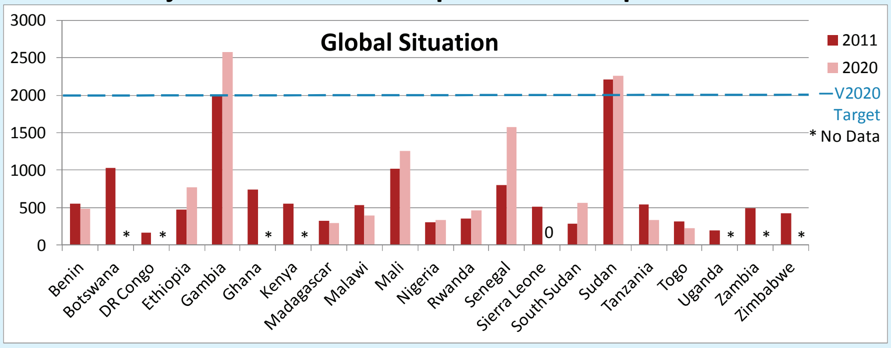

Sudan is currently above this VISION 2020 target and is expected to further increase this ratio by 2020.

Country Profile: Sudan

Current Situation: 2011

Total Population	Population Living in Capital City	% Population Living in Capital	Population Over 50	% Population Over 50
33,933,255	4,632,000	14%	3,686,000	11%

VISION 2020 Targets

Eye Care Cadre	# Practitioners per Million Population
Surgeons	4
OCOs/Nurses	10
Refractionists	20

Characteristics of the Active Eye Care Practitioner Workforce: 2011

Eye Care Cadre	Number of Practitioners in Active Workforce	Sector			Location		# Training Programmes available in Country
		Government	NGO/ Mission	Private for Profit	Capital	Outside Capital	
Ophthalmologists	298	171	84	43	ND	ND	8
Cataract Surgeons	0	0	0	0	0	0	0
**Surgeons**	**298**	**171**	**84**	**43**	**ND**	**ND**	**8**
Ophthalmic Clinical Officers	0	0	0	0	0	0	0
Ophthalmic Nurses	52	46	6	0	ND	ND	2
**OCOs/Nurses**	**52**	**46**	**6**	**0**	**ND**	**ND**	**2**
Optometrists	300	40	20	240	240	60	1
Mid-level Refractionists	36	0	20	16	28	8	2
**Refractionists**	**336**	**40**	**40**	**256**	**268**	**68**	**3**

Eye Care Practitioner Workforce Dynamics: 2011

Eye Care Cadre	Number of Practitioners in Active Workforce	Practitioners per Million Population			VISION 2020 Country Target # of Practitioners	Shortage in Practitioners to meet Target
		Countrywide	In Capital	Outside Capital		
Ophthalmologists	298	8.8	40.8	1.7		
Cataract Surgeons	0	0.0	0.0	0.0		
**Surgeons**	**298**	**8.8**	**40.8**	**1.7**	**132**	**Target Met**
Ophthalmic Clinical Officers	0	0.0	0.0	0.0		
Ophthalmic Nurses	52	1.5	5.0	0.1		
**OCOs/Nurses**	**52**	**1.5**	**5.0**	**0.1**	**329**	**277**
Optometrists	300	8.8	51.8	2.0		
Mid-level Refractionists	36	1.1	6.0	0.3		
**Refractionists**	**336**	**9.9**	**57.9**	**2.3**	**658**	**322**

Annual Cataract Surgical Performance

Number of Cataract Surgeries Performed (data from 2010)	75,000
Number of Cataract Surgeries per Surgeon (surgical performance ratio)	252
% Surgeries Performed by Ophthalmologists (estimate)	100%
Number of Cataract Surgeries per Million Population (CSR)	2,210
Target Number of Cataract Surgeries to meet VISION 2020 Target	67,867
Shortage in Cataract Surgeries to meet VISION 2020 Target	Target Met

ND: No Data

Projected Situation: 2020						
Projected Total Population	Projected Population Living in Capital City	% Projected Population Living in Capital	Projected Population Over 50	% Projected Population Over 50	Expected 9-year Population Increase	Expected 9-year Over 50 Population Increase
44,541,000	5,938,827	13%	4,887,000	11%	31%	33%

Projected Eye Care Practitioner Workforce Dynamics: 2020											
Eye Care Cadre	Number of Practitioners in Active Workforce (2011)	Over last 3 years			Projected Net Change over next 9 years	Projected Number of Practitioners in Active Workforce	Projected Practitioners per Million Population			VISION 2020 Country Target # of Practitioners	Projected Shortage in Practitioners to meet VISION 2020
		Number Entered Workforce	Number Exited Workforce	Net Change in Practitioner Numbers			Countrywide	In Capital	Outside Capital		
Ophthalmologists	298	48	14	34	102	400	9.0	ND	ND		
Cataract Surgeons	0	0	0	0	0	0	0.0	0.0	0.0		
**Surgeons**	**298**	**48**	**14**	**34**	**102**	**400**	**9.0**	**ND**	**ND**	**178**	**Target Met**
Ophthalmic Clinical Officers	0	0	0	0	0	0	0.0	0.0	0.0		
Ophthalmic Nurses	52	25	4	21	63	115	2.6	ND	ND		
**OCOs/Nurses**	**52**	**25**	**4**	**21**	**63**	**115**	**2.6**	**ND**	**ND**	**445**	**330**
Optometrists	300	40	34	6	18	318	7.1	42.8	1.6		
Mid-level Refractionists	36	18	20	-2	-6	30	0.7	3.9	0.2		
**Refractionists**	**336**	**58**	**54**	**4**	**12**	**348**	**7.8**	**46.7**	**1.8**	**891**	**543**

Annual Projected Cataract Surgical Performance: 2020							
Eye Care Cadre	% Surgeries Performed (estimate)	Surgical Performance Ratio per Cadre (2011)	Projected Number of Surgeons in Active Workforce	Projected Number of Cataract Surgeries Performed	Projected Number of Cataract Surgeries per Million Population (CSR)	Target Number of Cataract Surgeries to meet VISION 2020 Target	Projected Shortage in Cataract Surgeries to meet VISION 2020 Target
Ophthalmologists	100%	252	400	100,671			
Cataract Surgeons	0%	0	0	0			
**Surgeons**	**100%**	**252**	**400**	**100,671**	**2,260**	**89,082**	**Target Met**

Country Profile: United Republic of Tanzania

International Centre for Eye Health | AVRI African Vision Research Institute | Sightsavers

Key Messages

- **Surgeons:** The rate of surgeons per million population is set to decrease. Tanzania will need to more than triple its surgeon workforce to meet this target by 2020.
- **OCOs/Nurses:** Tanzania is currently a third of the way to meeting this VISION 2020 target. In order to meet it by 2020, the workforce will need to be more than tripled.
- **Refractionists:** Tanzania is currently a third of the way to meeting this VISION 2020 target. In order to meet it by 2020, the workforce will need to more than quadruple.
- **Cataract Surgeries:** Cataract surgeries are decreasing relative to population growth, meaning that by 2020 Tanzania will be a sixth of the way towards this target.

VISION 2020 Targets

	Eye Care Practitioners per Million Population			Cataract Surgeries Performed per Million Population	Cataract Surgeries Performed per Surgeon
	Surgeons*	Ophthalmic Clinical Officers /Nurses	Refractionists		
VISION2020 Target	4	10	20	2,000	500
2011 Situation	1.6	3.8	7.3	545	340
On Track					

* For the Africa region this includes Ophthalmologists and Cataract Surgeons

Eye Care Practitioners: % Working Inside/Outside Capital

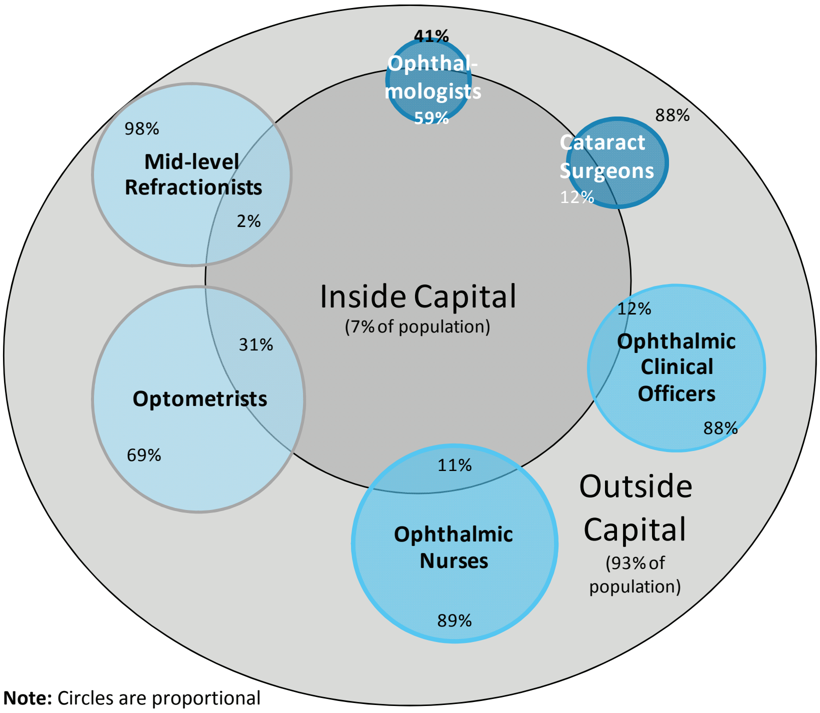

Note: Circles are proportional to numbers of eye care practitioners

In-Country Training Programmes

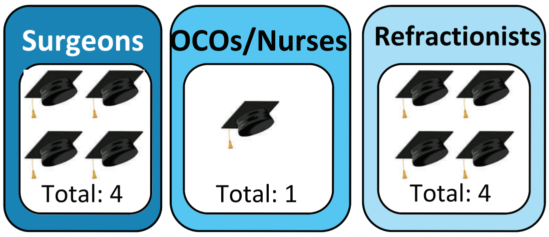

Distribution of Ophthalmologists

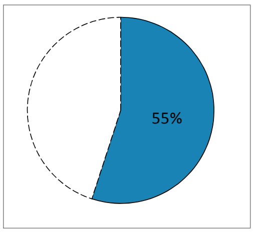

Ophthalmologists based in the capital

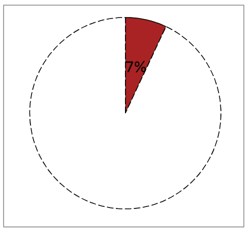

Population living in the capital

59% of Ophthalmologists treat 7% of the population

Eye Care Practitioners: Split between Sectors

Government | NGO/Mission | Private for Profit

Surgeons

Ophthalmologists

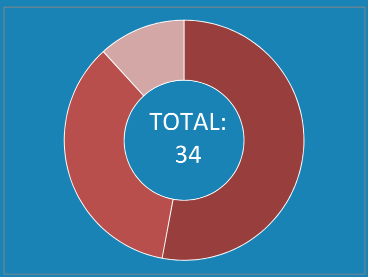

Cataract Surgeons

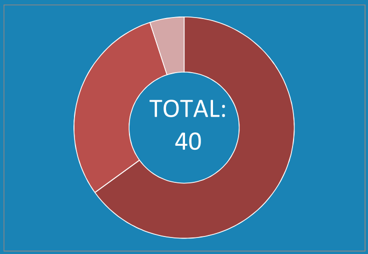

OCOs/Nurses

Ophthalmic Clinical Officers

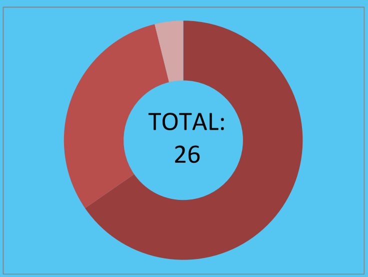

Ophthalmic Nurses

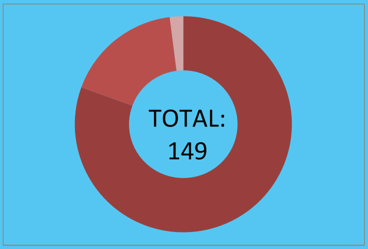

Refractionists

Optometrists

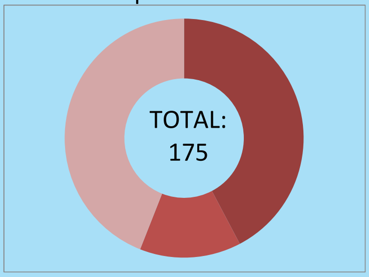

Mid-level Refractionists

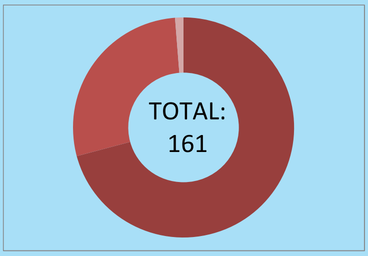

Surgeons: Current & Projected Workforce per Million Population

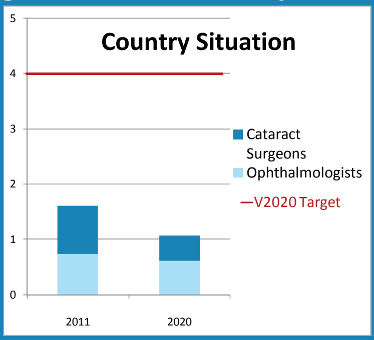

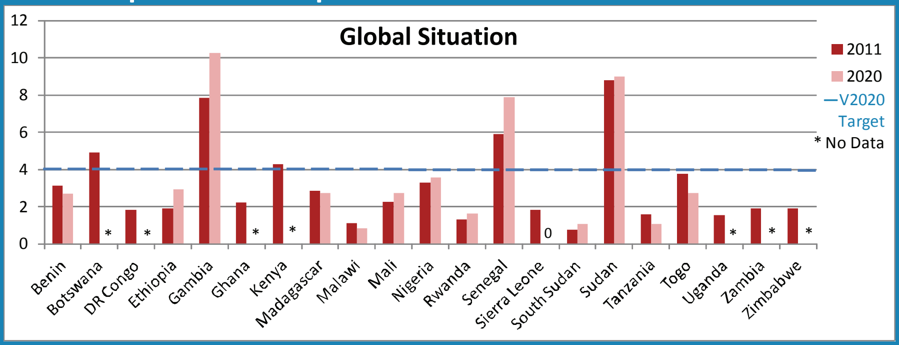

Practitioner Entry vs Exit: Over the past 3 years, more cataract surgeons have exited the workforce than have entered and only 1 more ophthalmologist has entered than those who exited the workforce. Tanzania is projected to have 3 more surgeons in the active workforce in 2020 than in 2011.
Practitioner Working Location: The overall surgeon to population ratio is 1.6. This ratio is 7.3 and 1.1 respectively for those working inside and outside Dar es Salaam.
Practitioner vs Population Growth: The number of surgeons is expected to grow at a slower rate than the general population, meaning Tanzania will be further from the target by 2020 unless current trends change.

OCOs/Nurses: Current & Projected Workforce per Million Population

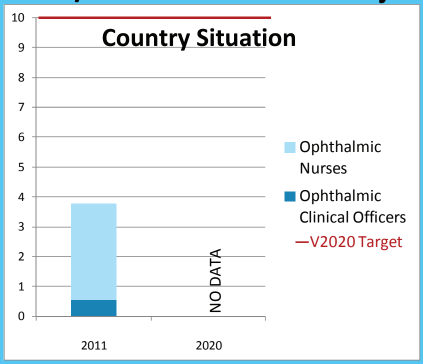

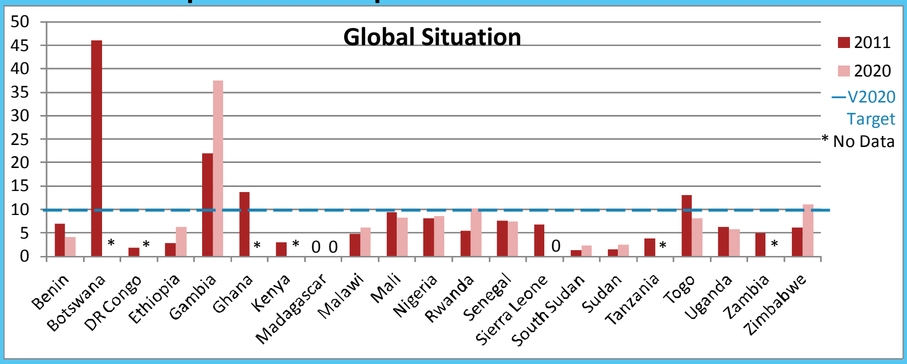

Practitioner Entry vs Exit: Over the past 3 years, 12 more ophthalmic clinical officers have entered the workforce than have exited. 38 ophthalmic nurses have entered the workforce, but exit data is not available so workforce projections are unavailable for 2020.
Practitioner Working Location: The overall practitioner to population ratio is 3.8. This ratio is 5.6 and 3.6 respectively for those working inside and outside Dar es Salaam.
Practitioner vs Population Growth: To account for population growth, the number of practitioners will need to more than triple by 2020 in order to meet the VISION 2020 target.

Refractionists: Current & Projected Workforce per Million Population

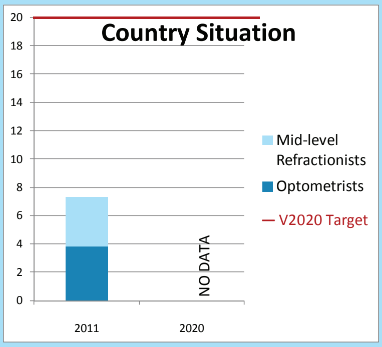

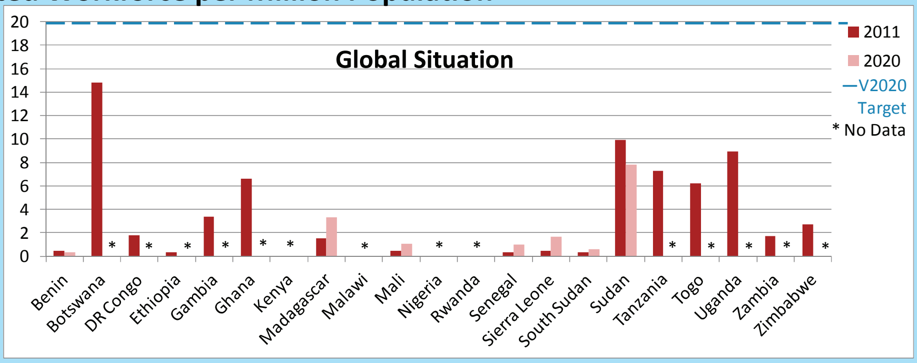

Practitioner Entry vs Exit: Entry and exit data is not available for refractionists.
Practitioner Working Location: The overall practitioner to population ratio is 7.3. This ratio is 17.0 and 6.5 respectively for those working inside and outside Dar es Salaam.
Practitioner vs Population Growth: There needs to be a 230% increase in practitioners numbers in order to meet the VISION 2020 target by 2020.

Cataract Surgical Performance: Current & Projected Performance per Million Population

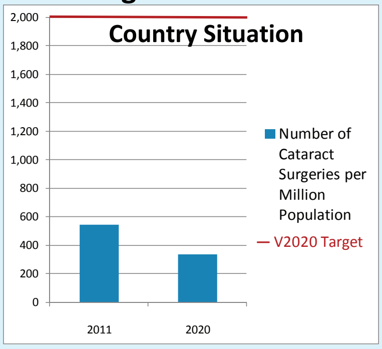

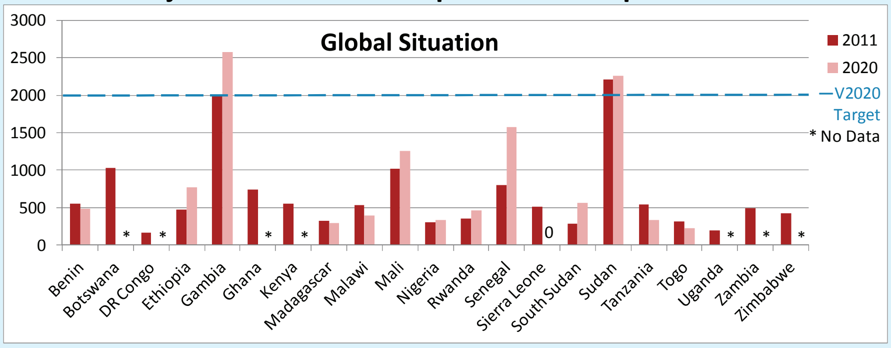

Given the number of surgeons is growing at a slower rate than the general population. Tanzania will be further from this target in 2020 unless current trends change.

Country Profile: United Republic of Tanzania

Current Situation: 2011

Total Population	Population Living in Capital City*	% Population Living in Capital	Population Over 50	% Population Over 50
46,250,317	3,415,000	7%	4,391,000	9%

VISION 2020 Targets

Eye Care Cadre	# Practitioners per Million Population
Surgeons	4
OCOs/Nurses	10
Refractionists	20

Characteristics of the Active Eye Care Practitioner Workforce: 2011

Eye Care Cadre	Number of Practitioners in Active Workforce	Sector			Location		# Training Programmes available in Country
		Government	NGO/ Mission	Private for Profit	Capital	Outside Capital	
Ophthalmologists	34	18	12	4	20	14	3
Cataract Surgeons **	40	26	12	2	5	35	1
**Surgeons**	**74**	**44**	**24**	**6**	**25**	**49**	**4**
Ophthalmic Clinical Officers	26	17	8	1	3	23	0
Ophthalmic Nurses	149	120	26	3	16	133	1
**OCOs/Nurses**	**175**	**137**	**34**	**4**	**19**	**156**	**1**
Optometrists	175	74	24	77	55	120	1
Mid-level Refractionists	161	114	45	2	3	158	3
**Refractionists**	**336**	**188**	**69**	**79**	**58**	**278**	**4**

Eye Care Practitioner Workforce Dynamics: 2011

Eye Care Cadre	Number of Practitioners in Active Workforce	Practitioners per Million Population			VISION 2020 Country Target # of Practitioners	Shortage in Practitioners to meet Target
		Countrywide	In Capital	Outside Capital		
Ophthalmologists	34	0.7	5.9	0.3		
Cataract Surgeons **	40	0.9	1.4	0.8		
**Surgeons**	**74**	**1.6**	**7.3**	**1.1**	**185**	**111**
Ophthalmic Clinical Officers	26	0.6	0.9	0.5		
Ophthalmic Nurses	149	3.2	4.7	3.1		
**OCOs/Nurses**	**175**	**3.8**	**5.6**	**3.6**	**463**	**288**
Optometrists	175	3.8	16.1	2.8		
Mid-level Refractionists	161	3.5	0.9	3.7		
**Refractionists**	**336**	**7.3**	**17.0**	**6.5**	**925**	**589**

Annual Cataract Surgical Performance

Number of Cataract Surgeries Performed (data from 2010)	25,188
Number of Cataract Surgeries per Surgeon (surgical performance ratio)	340
% Surgeries Performed by Ophthalmologists (estimate)	30%
Number of Cataract Surgeries per Million Population (CSR)	545
Target Number of Cataract Surgeries to meet VISION 2020 Target	92,501
Shortage in Cataract Surgeries to meet VISION 2020 Target	67,313

* Dar Es Salaam figures were used for “Capital City”

** Sector and location data for Assistant Medical Officers in Ophthalmology (AMOOs) has been reported in the same category as AMOO-Cataract Surgeons. Sector and location data has been divided proportionally between Cataract Surgeons and OCOs

Projected Situation: 2020

Projected Total Population	Projected Population Living in Capital City	% Projected Population Living in Capital	Projected Population Over 50	% Projected Population Over 50	Expected 9-year Population Increase	Expected 9-year Over 50 Population Increase
61,101,208	5,452,417	9%	5,703,000	9%	32%	30%

Projected Eye Care Practitioner Workforce Dynamics: 2020

Eye Care Cadre	Number of Practitioners in Active Workforce (2011)	Over last 3 years			Projected Net Change over next 9 years	Projected Number of Practitioners in Active Workforce	Projected Practitioners per Million Population			VISION 2020 Country Target # of Practitioners	Projected Shortage in Practitioners to meet VISION 2020
		Number Entered Workforce	Number Exited Workforce	Net Change in Practitioner Numbers			Countrywide	In Capital	Outside Capital		
Ophthalmologists	34	8	7	1	3	37	0.6	4.0	0.3		
Cataract Surgeons +	40	3	7	-4	-12	28	0.5	0.6	0.4		
**Surgeons**	**74**	**11**	**14**	**-3**	**-9**	**65**	**1.1**	**4.0**	**0.8**	**244**	**179**
Ophthalmic Clinical Officers	26	17	5	12	36	62	1.0	1.4	1.0		
Ophthalmic Nurses	149	38	ND	ND	ND	ND	ND	ND	ND		
**OCOs/Nurses**	**175**	**55**	**ND**	**ND**	**ND**	**ND**	**ND**	**ND**	**ND**	**611**	**ND**
Optometrists	175	37	ND	ND	ND	ND	ND	ND	ND		
Mid-level Refractionists	161	9	ND	ND	ND	ND	ND	ND	ND		
**Refractionists**	**336**	**46**	**ND**	**ND**	**ND**	**ND**	**ND**	**ND**	**ND**	**1,222**	**ND**

Annual Projected Cataract Surgical Performance: 2020

Eye Care Cadre	% Surgeries Performed (estimate)	Surgical Performance Ratio per Cadre (2011)	Projected Number of Surgeons in Active Workforce	Projected Number of Cataract Surgeries Performed	Projected Number of Cataract Surgeries per Million Population (CSR)	Target Number of Cataract Surgeries to meet VISION 2020 Target	Projected Shortage in Cataract Surgeries to meet VISION 2020 Target
Ophthalmologists	30%	222	37	8,223			
Cataract Surgeons	70%	441	28	12,342			
**Surgeons**	**100%**	**340**	**65**	**20,565**	**337**	**122,202**	**101,637**

+ Entry/Exit data for Assistant Medical Officers in Ophthalmology (AMOOs) has been reported in the same category as AMOO-Cataract Surgeons   ND: No Data

Country Profile: Togo

Key Messages

- **Surgeons:** Togo is currently almost at this VISION 2020 target. However, due to decreasing surgeon numbers, this practitioner to population ratio is projected to decrease over time.
- **OCOs/Nurses:** Togo has already met this VISION 2020 target. The number of ophthalmic nurses is decreasing, meaning that unless this trend changes, Togo will be under target by 2020.
- **Refractionists:** In order to meet this VISION 2020 target by 2020, Togo will need to nearly quadruple their workforce.
- **Cataract Surgeries:** With the decreasing number of surgeons , cataract surgeries are also decreasing. By 2020 Togo will be 10% of the way towards meeting this target, requiring an additional 13,000 surgeries per year.

International Centre for Eye Health | AVRI African Vision Research Institute | Sightsavers

VISION 2020 Targets

	Eye Care Practitioners per Million Population			Cataract Surgeries Performed per Million Population	Cataract Surgeries Performed per Surgeon
	Surgeons*	Ophthalmic Clinical Officers /Nurses	Refractionists		
VISION2020 Target	4	10	20	2,000	500
2011 Situation	3.8	13.0	6.2	318	85
On Track					

* For the Africa region this includes Ophthalmologists and Cataract Surgeons

Eye Care Practitioners: % Working Inside/Outside Capital

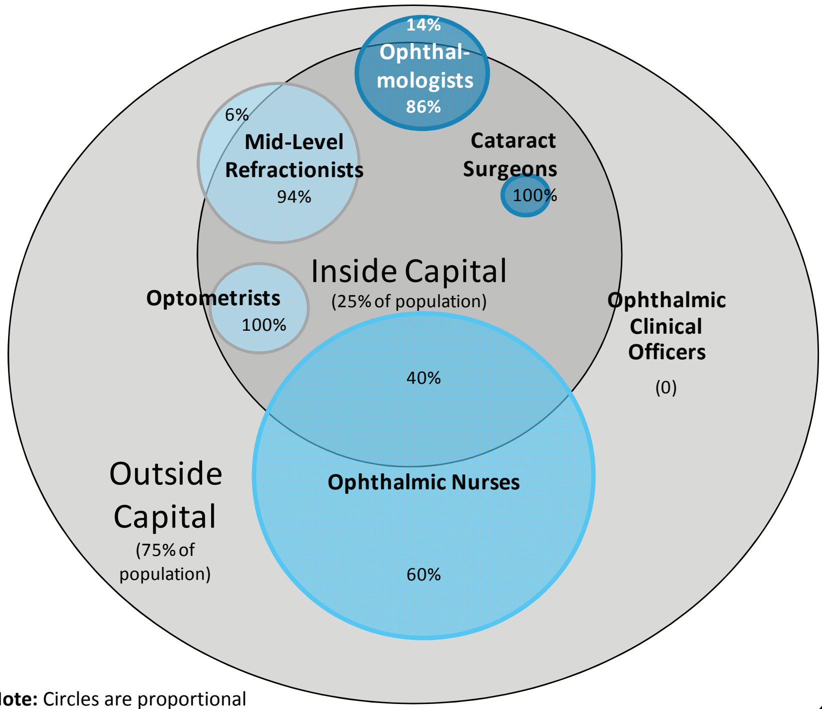

Note: Circles are proportional to numbers of eye care practitioners

In-Country Training Programmes

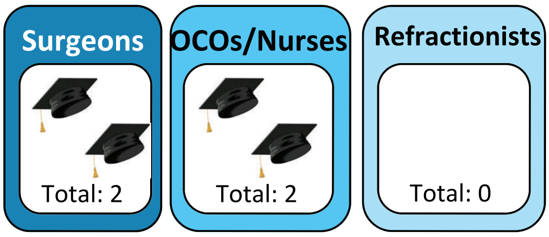

Distribution of Refractionists

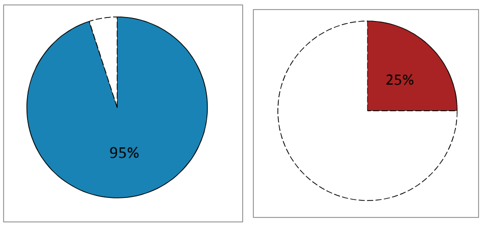

Refractionists based in the capital | Population living in the capital

95% of all Refractionists treat 25% of the population

Eye Care Practitioners: Split between Sectors

Government | NGO/Mission | Private for Profit

Surgeons

Ophthalmologists

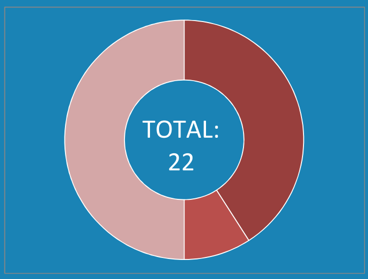

Cataract Surgeons

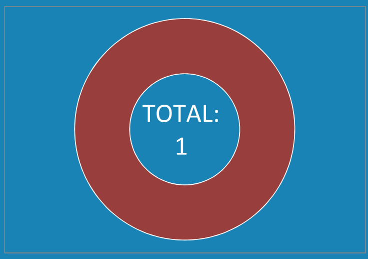

OCOs/Nurses

Ophthalmic Clinical Officers

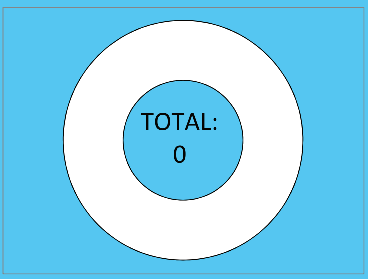

Ophthalmic Nurses

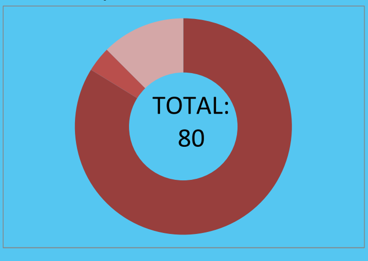

Refractionists

Optometrists

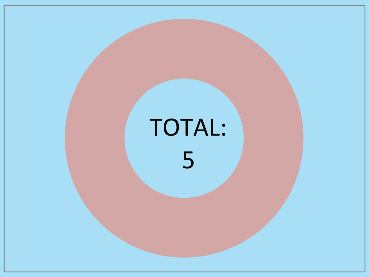

Mid-level Refractionists

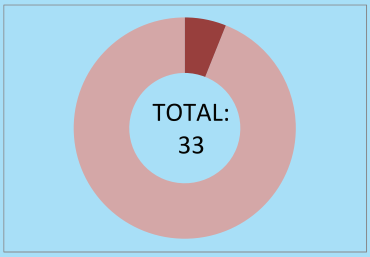

Surgeons: Current & Projected Workforce per Million Population

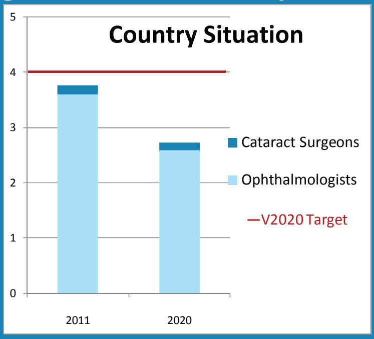

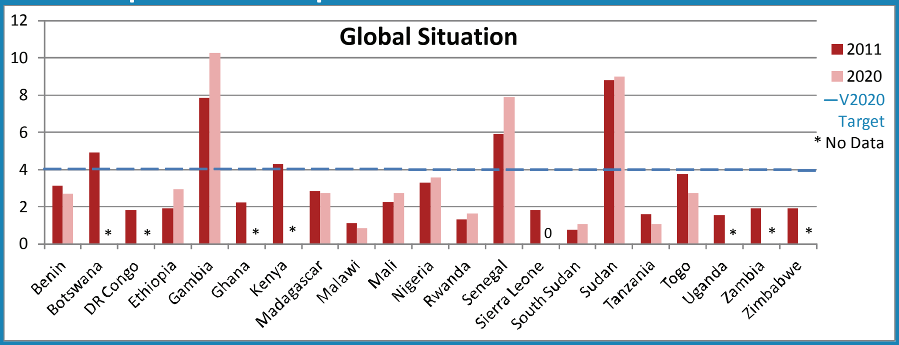

Practitioner Entry vs Exit: Over the past 3 years more surgeons exited the workforce than entered.
Practitioner Working Location: The overall surgeon to population ratio is 3.8. This ratio is 13.1 and 0.6 respectively for those working inside and outside the capital.
Practitioner vs Population Growth: With the decreasing number of surgeons, Togo will be further from the VISION 2020 target by 2020. If Togo were able to increase the surgeon workforce by 23%, they would achieve this target by 2020.

OCOs/Nurses: Current & Projected Workforce per Million Population

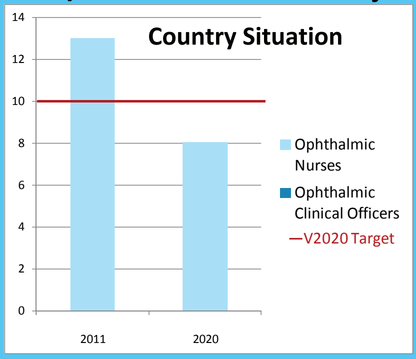

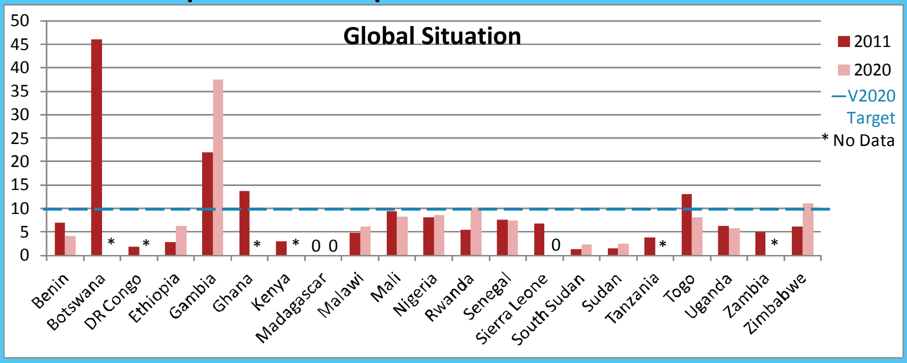

Practitioner Entry vs Exit: There are no ophthalmic clinical officers in Togo. Over the past 3 years more ophthalmic nurses exited the workforce than entered.
Practitioner Working Location: The overall practitioner to population ratio is 13.0. This ratio is 21.0 and 10.4 respectively for those working inside and outside the capital.
Practitioner vs Population Growth: Ophthalmic nurse numbers are expected to decrease, meaning that although Togo exceeded the VISION 2020 target in 2011, it is projected to be under target by 2020.

Refractionists: Current & Projected Workforce per Million Population

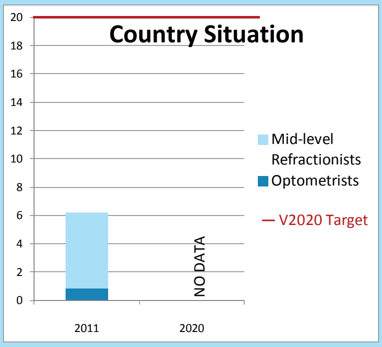

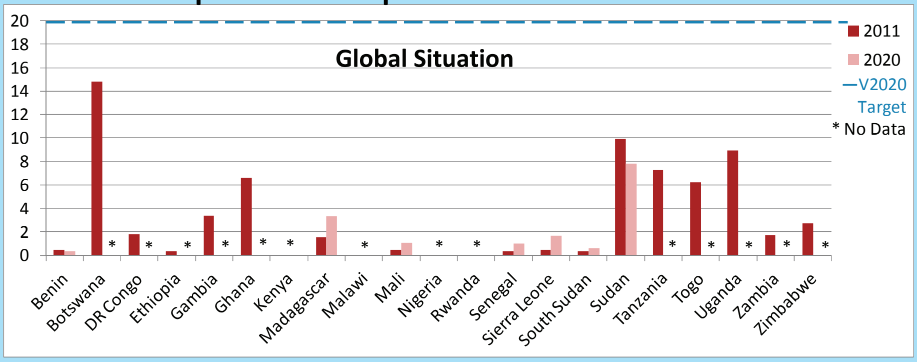

Practitioner Entry vs Exit: There are 5 optometrists working in Togo. None have entered or exited the workforce in the past 3 years. There are 33 mid-level refractionists, but no information on their movement into or out of the workforce.
Practitioner Working Location: All of the optometrists and all but 2 of the mid-level refractionists work in the private sector. The overall refractionist to population ratio is 6.21. This ratio is 23.6 and 0.4 respectively for those working inside and outside the capital.
Practitioner vs Population Growth: There needs to be a 287% increase in the refractionists workforce to meet the target by 2020, accounting for the expected 19% population growth between 2011 and 2020.

Cataract Surgical Performance: Current & Projected Performance per Million Population

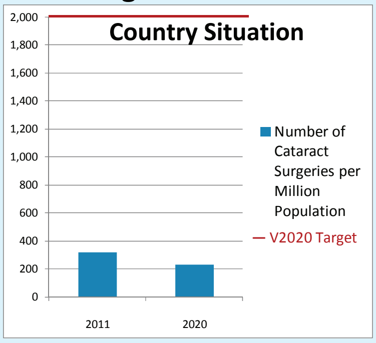

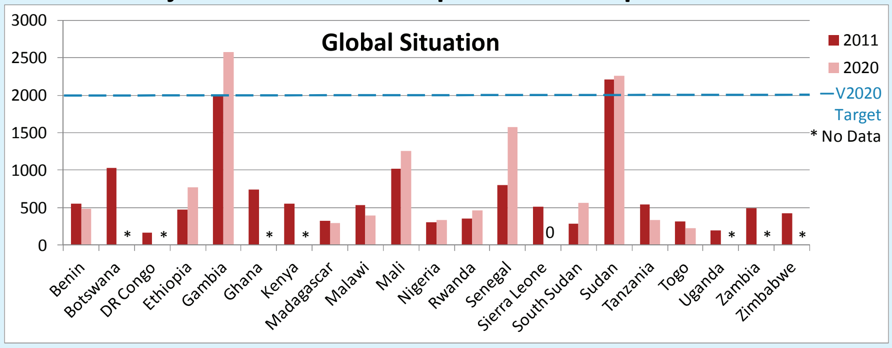

With the decreasing number of surgeons, the number of cataract surgeries is also expected to decrease. Togo will need to perform nearly 7 times as many cataract surgeries each year in order to meet the target by 2020.

Country Profile: Togo

Current Situation: 2011

Total Population	Population Living in Capital City	% Population Living in Capital	Population Over 50	% Population Over 50
6,147,889	1,524,000	25%	603,000	10%

VISION 2020 Targets

Eye Care Cadre	# Practitioners per Million Population
Surgeons	4
OCOs/Nurses	10
Refractionists	20

Characteristics of the Active Eye Care Practitioner Workforce: 2011

Eye Care Cadre	Number of Practitioners in Active Workforce	Sector			Location		# Training Programmes available in Country
		Government	NGO/ Mission	Private for Profit	Capital	Outside Capital	
Ophthalmologists	22	9	2	11	19	3	2
Cataract Surgeons	1	1	0	0	1	0	0
**Surgeons**	**23**	**10**	**2**	**11**	**20**	**3**	**2**
Ophthalmic Clinical Officers	0	0	0	0	0	0	0
Ophthalmic Nurses	80	67	3	10	32	48	2
**OCOs/Nurses**	**80**	**67**	**3**	**10**	**32**	**48**	**2**
Optometrists	5	0	0	5	5	0	0
Mid-level Refractionists	33	2	0	31	31	2	0
**Refractionists**	**38**	**2**	**0**	**36**	**36**	**2**	**0**

Eye Care Practitioner Workforce Dynamics: 2011

Eye Care Cadre	Number of Practitioners in Active Workforce	Practitioners per Million Population			VISION 2020 Country Target # of Practitioners	Shortage in Practitioners to meet Target
		Countrywide	In Capital	Outside Capital		
Ophthalmologists	22	3.6	12.5	0.6		
Cataract Surgeons	1	0.2	0.7	0.0		
**Surgeons**	**23**	**3.8**	**13.1**	**0.6**	**25**	**2**
Ophthalmic Clinical Officers	0	0	0.0	0.0		
Ophthalmic Nurses	80	13	21.0	10.4		
**OCOs/Nurses**	**80**	**13**	**21.0**	**10.4**	**61**	**Target Met**
Optometrists	5	0.8	3.3	0.0		
Mid-level Refractionists	33	5.4	20.3	0.4		
**Refractionists**	**38**	**6.2**	**23.6**	**0.4**	**123**	**85**

Annual Cataract Surgical Performance

Number of Cataract Surgeries Performed (data from 2010)	1,952
Number of Cataract Surgeries per Surgeon (surgical performance ratio)	85
% Surgeries Performed by Ophthalmologists (estimate)	100%
Number of Cataract Surgeries per Million Population (CSR)	318
Target Number of Cataract Surgeries to meet VISION 2020 Target	12,296
Shortage in Cataract Surgeries to meet VISION 2020 Target	10,344

Projected Situation: 2020						
Projected Total Population	Projected Population Living in Capital City	% Projected Population Living in Capital	Projected Population Over 50	% Projected Population Over 50	Expected 9-year Population Increase	Expected 9-year Over 50 Population Increase
7,342,292	2,026,434	28%	782,000	11%	19%	30%

Projected Eye Care Practitioner Workforce Dynamics: 2020											
Eye Care Cadre	Number of Practitioners in Active Workforce (2011)	Over last 3 years			Projected Net Change over next 9 years	Projected Number of Practitioners in Active Workforce	Projected Practitioners per Million Population			VISION 2020 Country Target # of Practitioners	Projected Shortage in Practitioners to meet VISION 2020
		Number Entered Workforce	Number Exited Workforce	Net Change in Practitioner Numbers			Countrywide	In Capital	Outside Capital		
Ophthalmologists	22	3	4	-1	-3	19	2.6	8.1	0.5		
Cataract Surgeons	1	1	1	0	0	1	0.1	0.5	0.0		
**Surgeons**	**23**	**4**	**5**	**-1**	**-3**	**20**	**2.7**	**8.6**	**0.5**	**29**	**9**
Ophthalmic Clinical Officers	0	0	0	0	0	0	0.0	0.0	0.0		
Ophthalmic Nurses	80	3	10	-7	-21	59	8.0	11.6	6.7		
**OCOs/Nurses**	**80**	**3**	**10**	**-7**	**-21**	**59**	**8.0**	**11.6**	**6.7**	**73**	**14**
Optometrists	5	0	0	0	0	5	0.7	2.5	0.0		
Mid-level Refractionists	33	0	33	-33	-99	0	0.0	0.0	0.0		
**Refractionists**	**38**	**0**	**33**	**-33**	**-99**	**5**	**0.7**	**2.3**	**0.0**	**147**	**142**

Annual Projected Cataract Surgical Performance: 2020							
Eye Care Cadre	% Surgeries Performed (estimate)	Surgical Performance Ratio per Cadre (2011)	Projected Number of Surgeons in Active Workforce	Projected Number of Cataract Surgeries Performed	Projected Number of Cataract Surgeries per Million Population (CSR)	Target Number of Cataract Surgeries to meet VISION 2020 Target	Projected Shortage in Cataract Surgeries to meet VISION 2020 Target
Ophthalmologists	100%	89	19	1,686			
Cataract Surgeons	0%	0	1	0			
**Surgeons**	**100%**	**85**	**20**	**1,686**	**230**	**14,685**	**12,999**

Country Profile: Uganda

Key Messages

- **Surgeons:** Uganda is currently less than half-way to meeting this target and will need to recruit an additional 130 surgeons by 2020 to meet this target.
- **OCOs/Nurses:** As the practitioner to population ratio is increasing at a slower rate than population growth, by 2020 Uganda will be further from meeting this target.
- **Refractionists:** Uganda has 4 optometrists and a decreasing number of mid-level refractionists. The country is currently less than half-way to meeting this target and will need to recruit an additional 600 practitioners.
- **Cataract Surgeries:** Uganda is currently less than 10% of the way towards meeting this target.

International Centre for Eye Health | AVRI African Vision Research Institute | Sightsavers

VISION 2020 Targets

	Eye Care Practitioners per Million Population			Cataract Surgeries Performed per Million Population	Cataract Surgeries Performed per Surgeon
	Surgeons*	Ophthalmic Clinical Officers /Nurses	Refraction-ists		
VISION2020 Target	4	10	20	2,000	500
2011 Situation	1.6	6.3	8.9	197	130
On Track					

* For the Africa region this includes Ophthalmologists and Cataract Surgeons

Eye Care Practitioners: % Working Inside/Outside Capital

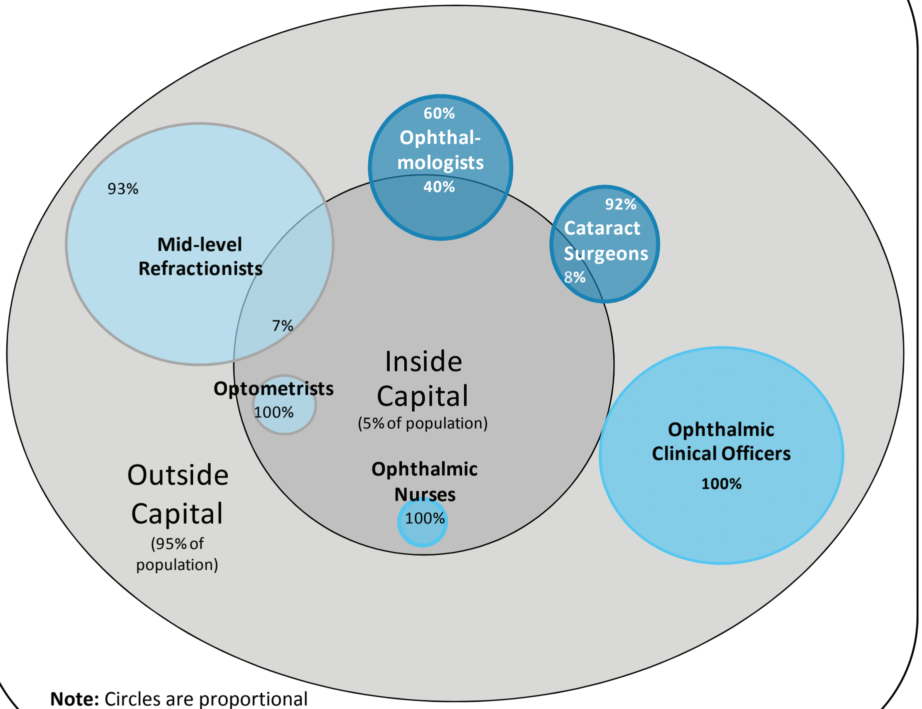

Note: Circles are proportional to numbers of eye care practitioners

In-Country Training Programmes

Surgeons	OCOs/Nurses	Refractionists
Total: 2	Total: 2	Total: 3

Distribution of Ophthalmologists

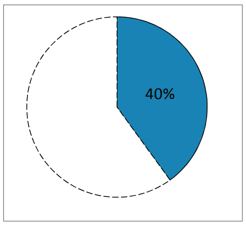

Ophthalmologists based in the capital

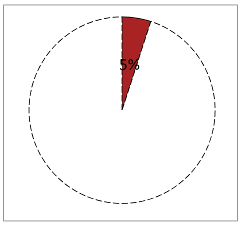

Population living in the capital

40% of Ophthalmologists treat 5% of the population

Eye Care Practitioners: Split between Sectors

Government | NGO/Mission | Private for Profit

Surgeons

Ophthalmologists

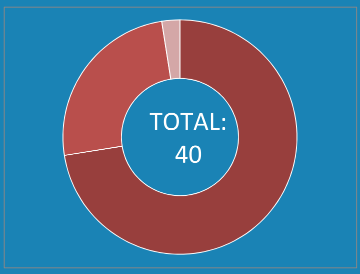

Cataract Surgeons

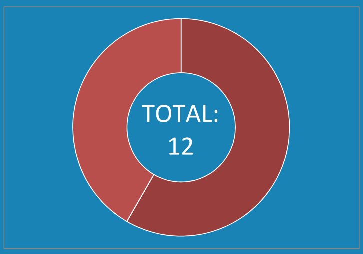

OCOs/Nurses

Ophthalmic Clinical Officers

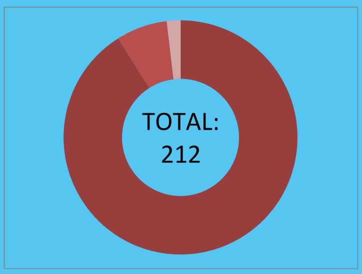

Ophthalmic Nurses

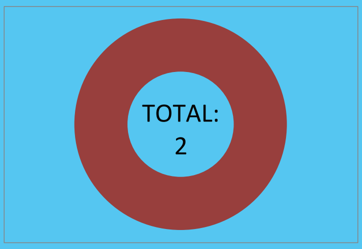

Refractionists

Optometrists

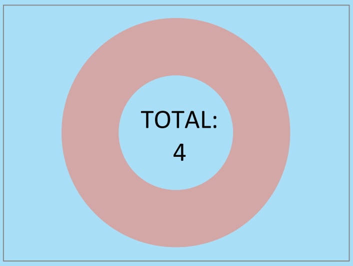

Mid-level Refractionists

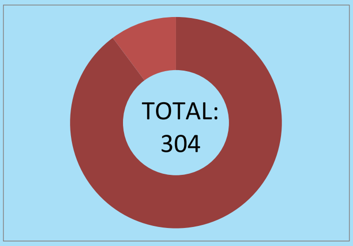

Surgeons: Current & Projected Workforce per Million Population

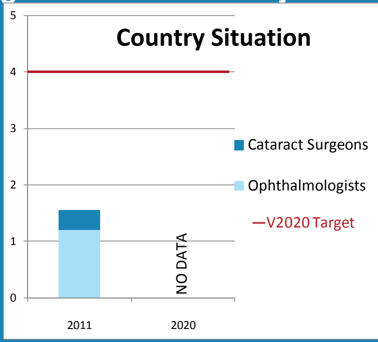

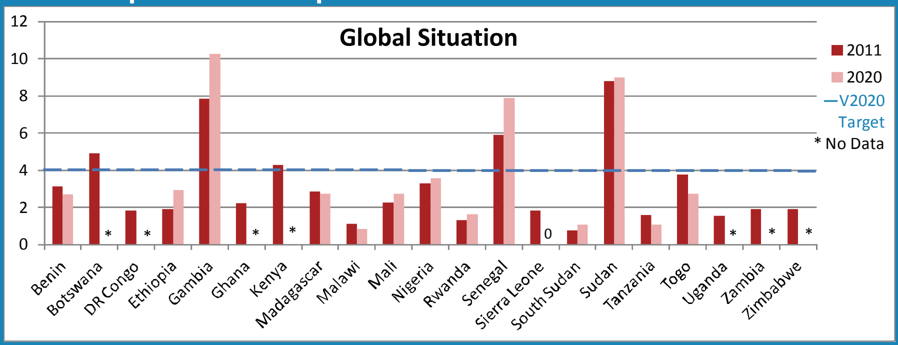

Practitioner Entry vs Exit: Over the past 3 years more ophthalmologists have exited the workforce than have entered. As there is no entry and exit data for cataract surgeons, projections for the situation in 2020 is not available.

Practitioner Working Location: The overall surgeon to population ratio is 1.56. This ratio is 10.2 and 1.1 respectively for those working inside and outside the capital.

Practitioner vs Population Growth: Surgeon numbers will need to more than triple by 2020 in order to meet the target, accounting for the expected 32% population increase between 2011 and 2020.

OCOs/Nurses: Current & Projected Workforce per Million Population

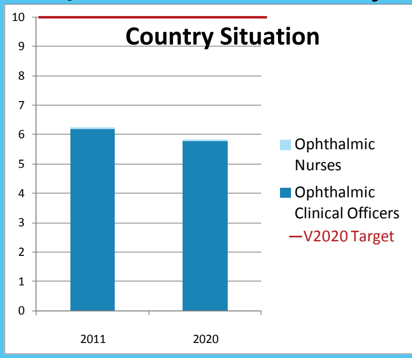

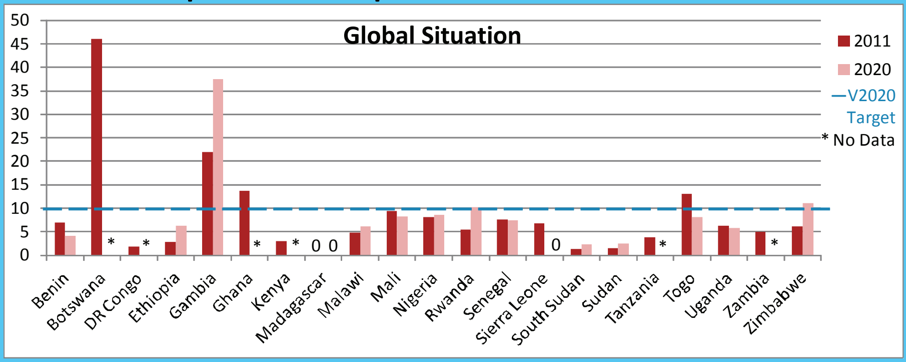

Practitioner Entry vs Exit: Over the past 3 years more ophthalmic clinical officers have entered the workforce than have exited.

Practitioner Working Location: All of the ophthalmic clinical officers work outside the capital. The overall practitioner to population ratio is 6.3. This ratio is 1.2 and 6.5 respectively for those working inside and outside the capital.

Practitioner vs Population Growth: Between 2011 and 20202, the number of practitioners is expected to grow at a slower rate than the general population, 24% compared with 32%, meaning that Uganda will be further from the target in 2020.

Refractionists: Current & Projected Workforce per Million Population

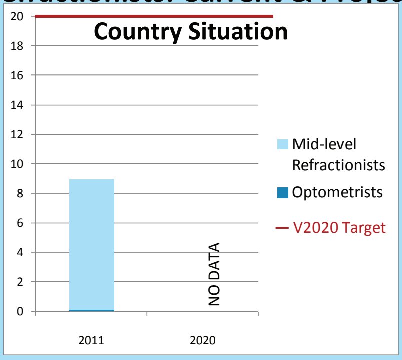

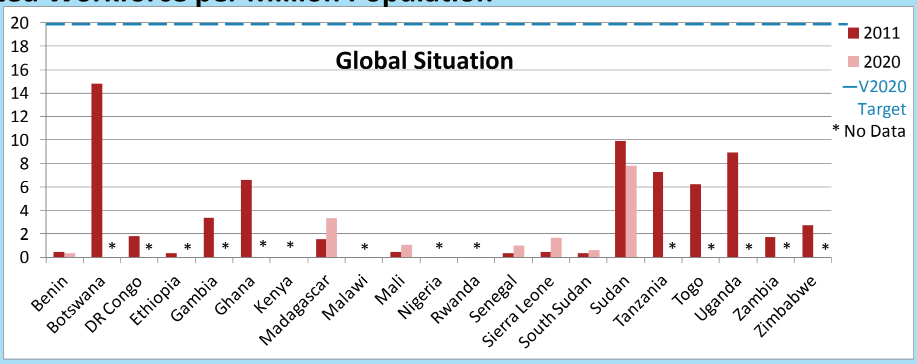

Practitioner Entry vs Exit: More mid-level refractionists left the workforce than entered over the past 3 years. There are 4 optometrists working in Uganda, but no available information on their movement into and out of the workforce.

Practitioner Working Location: All 4 optometrists work in the capital. The overall refractionist to population ratio is 8.9. This ratio is 15.7 and 8.6 respectively for those working inside and outside the capital.

Practitioner vs Population Growth: The number of refractionists will need to triple by 2020 to meet the target, accounting for the expected population growth.

Cataract Surgical Performance: Current & Projected Performance per Million Population

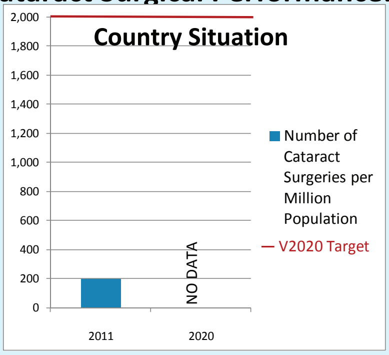

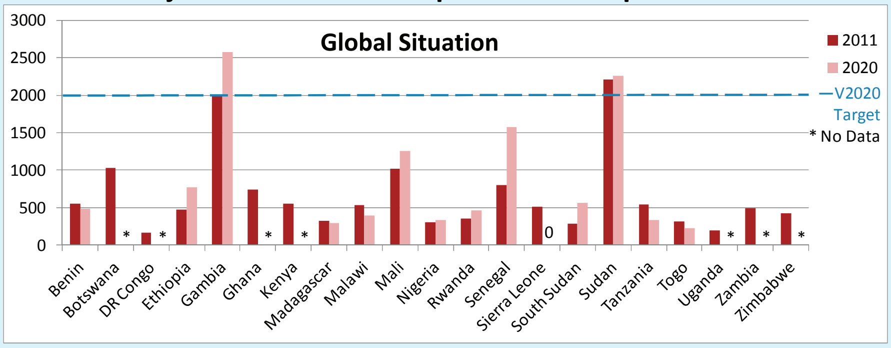

Uganda will need to carry out more than 13 times as many cataract surgeries each year in order to meet the target by 2020, accounting for population growth.

Country Profile: Uganda

Current Situation: 2011

Total Population	Population Living in Capital City	% Population Living in Capital	Population Over 50	% Population Over 50
34,466,105	1,659,000	5%	2,620,000	8%

VISION 2020 Targets

Eye Care Cadre	# Practitioners per Million Population
Surgeons	4
OCOs/Nurses	10
Refractionists	20

Characteristics of the Active Eye Care Practitioner Workforce: 2011

Eye Care Cadre	Number of Practitioners in Active Workforce	Sector			Location		# Training Programmes available in Country
		Government	NGO/ Mission	Private for Profit	Capital	Outside Capital	
Ophthalmologists	40	29	10	1	16	24	2
Cataract Surgeons	12	7	5	0	1	11	0
**Surgeons**	**52**	**36**	**15**	**1**	**17**	**35**	**2**
Ophthalmic Clinical Officers	212	193	15	4	0	212	1
Ophthalmic Nurses	2	2	0	0	2	0	1
**OCOs/Nurses**	**214**	**195**	**15**	**4**	**2**	**212**	**2**
Optometrists	4	0	0	4	4	0	0
Mid-level Refractionists *	304	273	31	ND	22	282	3
**Refractionists**	**308**	**273**	**31**	**ND**	**26**	**282**	**3**

Eye Care Practitioner Workforce Dynamics: 2011

Eye Care Cadre	Number of Practitioners in Active Workforce	Practitioners per Million Population			VISION 2020 Country Target # of Practitioners	Shortage in Practitioners to meet Target
		Country-wide	In Capital	Outside Capital		
Ophthalmologists	40	1.2	9.6	0.7		
Cataract Surgeons	12	0.35	0.6	0.3		
**Surgeons**	**52**	**1.55**	**10.2**	**1.1**	**138**	**86**
Ophthalmic Clinical Officers	212	6.2	0.0	6.5		
Ophthalmic Nurses	2	0.1	1.2	0.0		
**OCOs/Nurses**	**214**	**6.3**	**1.2**	**6.5**	**345**	**131**
Optometrists	4	0.1	2.4	0.0		
Mid-level Refractionists	304	8.8	13.3	8.6		
**Refractionists**	**308**	**8.9**	**15.7**	**8.6**	**689**	**381**

Annual Cataract Surgical Performance

Number of Cataract Surgeries Performed (data from 2010)	6,780
Number of Cataract Surgeries per Surgeon (surgical performance ratio)	130
% Surgeries Performed by Ophthalmologists (estimate)	94%
Number of Cataract Surgeries per Million Population (CSR)	197
Target Number of Cataract Surgeries to meet VISION 2020 Target	68,932
Shortage in Cataract Surgeries to meet VISION 2020 Target	62,152

* Mid-level Refractionsists excludes MLEPs who may be working in the Private for Profit Sector

ND: No Data

Projected Situation: 2020

Projected Total Population	Projected Population Living in Capital City	% Projected Population Living in Capital	Projected Population Over 50	% Projected Population Over 50	Expected 9-year Population Increase	Expected 9-year Over 50 Population Increase
45,427,240	2,708,035	6%	3,485,000	8%	32%	33%

Projected Eye Care Practitioner Workforce Dynamics: 2020

Eye Care Cadre	Number of Practitioners in Active Workforce (2011)	Over last 3 years			Projected Net Change over next 9 years	Projected Number of Practitioners in Active Workforce	Projected Practitioners per Million Population			VISION 2020 Country Target # of Practitioners	Projected Shortage in Practitioners to meet VISION 2020
		Number Entered Workforce	Number Exited Workforce	Net Change in Practitioner Numbers			Countrywide	In Capital	Outside Capital		
Ophthalmologists	40	5	6	-1	-3	37	0.8	5.5	0.5		
Cataract Surgeons	12	ND	ND	ND	ND	ND	ND	ND	ND		
**Surgeons**	**52**	**ND**	**ND**	**ND**	**ND**	**ND**	**ND**	**ND**	**ND**	**182**	**ND**
Ophthalmic Clinical Officers	212	26	9	17	51	263	5.8	0.0	6.2		
Ophthalmic Nurses	2	0	0	0	0	2	0.0	0.7	0.0		
**OCOs/Nurses**	**214**	**26**	**9**	**17**	**51**	**265**	**5.8**	**0.9**	**6.1**	**454**	**189**
Optometrists	4	ND	ND	ND	ND	ND	ND	ND	ND		
Mid-level Refractionists	304	72	73	-1	-3	301	6.6	8.0	6.5		
**Refractionists**	**308**	**ND**	**ND**	**ND**	**ND**	**ND**	**ND**	**ND**	**ND**	**909**	**ND**

Annual Projected Cataract Surgical Performance: 2020

Eye Care Cadre	% Surgeries Performed (estimate)	Surgical Performance Ratio per Cadre (2011)	Projected Number of Surgeons in Active Workforce	Projected Number of Cataract Surgeries Performed	Projected Number of Cataract Surgeries per Million Population (CSR)	Target Number of Cataract Surgeries to meet VISION 2020 Target	Projected Shortage in Cataract Surgeries to meet VISION 2020 Target
Ophthalmologists	94%	159	37	5,895			
Cataract Surgeons	6%	34	ND	ND			
**Surgeons**	**100%**	**130**	**ND**	**ND**	**ND**	**90,854**	**ND**

ND: No Data

Country Profile: Zambia

Key Messages

- **Surgeons:** Zambia is currently less than half-way to meeting this target and will need to triple its workforce by 2020 in order to meet the target.
- **OCOs/Nurses:** Zambia is half-way to meeting this target and will need to recruit an additional 100 people by 2020 in order to meet the target.
- **Refractionists:** Zambia is less than 10% of the way to meeting this target.
- **Cataract Surgeries:** Zambia is a quarter of the way to meeting this target and will need to triple the number of annual surgeries performed in order to meet the target.

VISION 2020 Targets

	Eye Care Practitioners per Million Population			Cataract Surgeries Performed per Million Population	Cataract Surgeries Performed per Surgeon
	Surgeons*	Ophthalmic Clinical Officers /Nurses	Refraction-ists		
VISION2020 Target	4	10	20	2,000	500
2011 Situation	1.9	5.0	1.7	499	259
On Track					

* For the Africa region this includes Ophthalmologists and Cataract Surgeons

Eye Care Practitioners: % Working Inside/Outside Capital

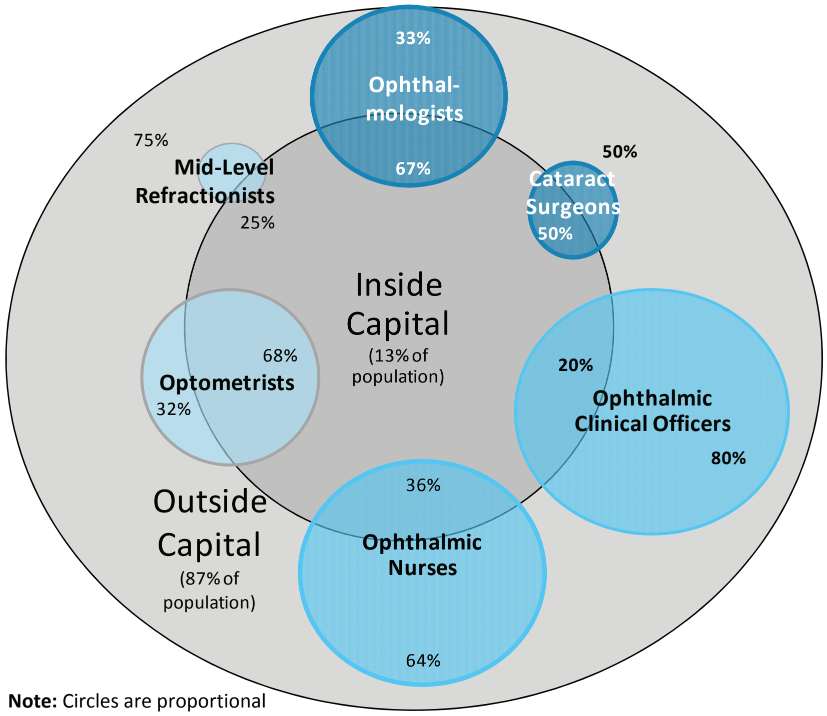

Note: Circles are proportional to numbers of eye care practitioners

In-Country Training Programmes

Surgeons	OCOs/Nurses	Refractionists
Total: 1	Total: 1	Total: 1

Distribution of Refractionists

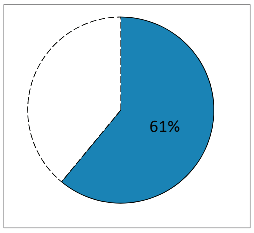

Refractionists based in the capital

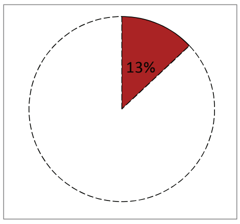

Population living in the capital

61% of all Refractionists treat 13% of the population

Eye Care Practitioners: Split between Sectors

Government · NGO/Mission · Private for Profit

Surgeons

Ophthalmologists

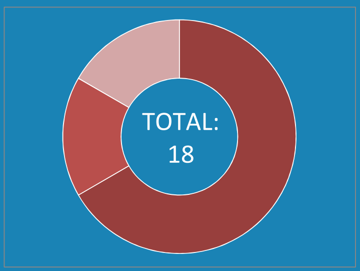

Cataract Surgeons

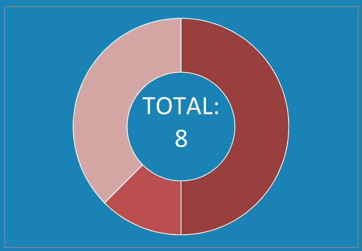

OCOs/Nurses

Ophthalmic Clinical Officers

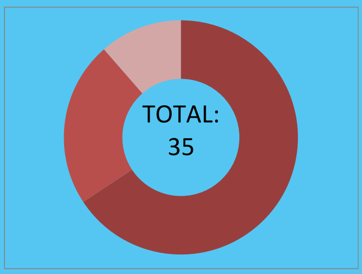

Ophthalmic Nurses

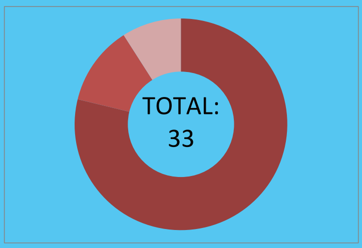

Refractionists

Optometrists

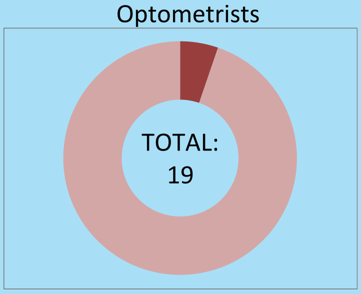

Mid-level Refractionists

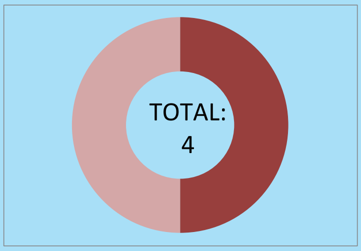

Surgeons: Current & Projected Workforce per Million Population

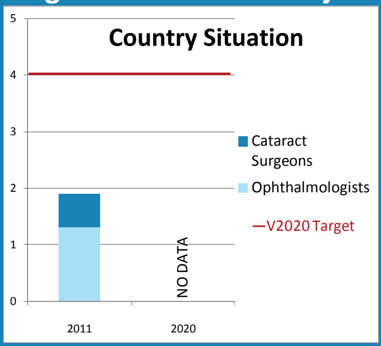

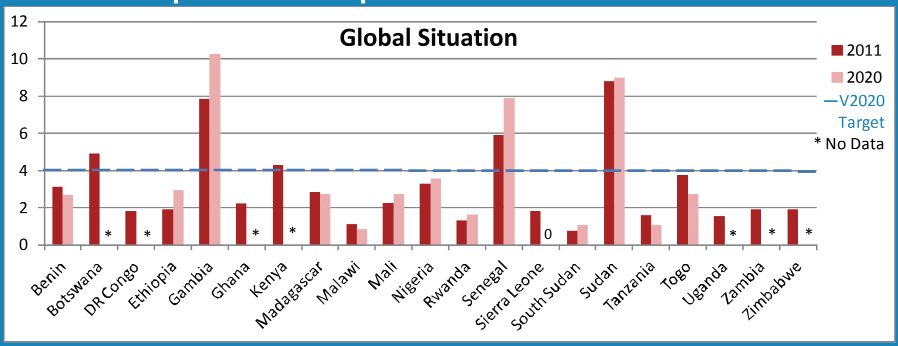

Practitioner Entry vs Exit: Over the past 3 years, 2 ophthalmologists have entered the workforce and no cataract surgeons. There is no information on how many have left the workforce, so 2020 projections are not available.

Practitioner Working Location: The overall surgeon to population ratio is 1.9. This ratio is 5.5 and 1.4 respectively for those working inside and outside the capital.

Practitioner vs Population Growth: Accounting for the expected 33% population growth between 2011 and 2020, surgeon numbers will need to triple in order to meet the target by 2020.

OCOs/Nurses: Current & Projected Workforce per Million Population

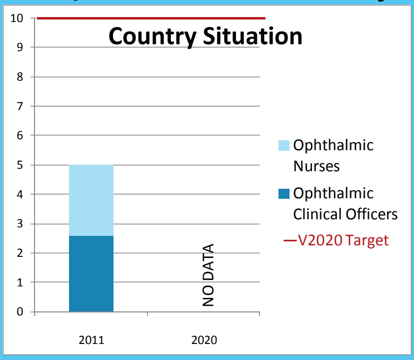

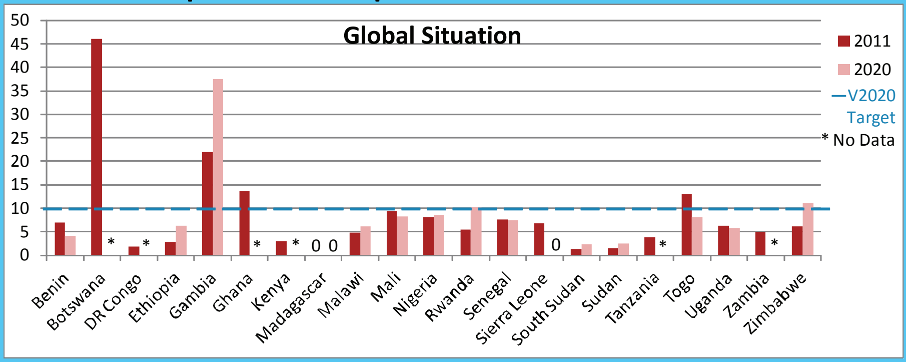

Practitioner Entry vs Exit: Over the past 3 years, 23 ophthalmic clinical officers and 22 ophthalmic nurses have entered the workforce. There is no information on how many have left the workforce, so 2020 projections are not available.

Practitioner Working Location: Although Zambia is halfway to meeting this target, with an overall practitioner to population ratio is 5.0, this is not the case for practitioners based outside the capital. This ratio is 10.5 and 4.2 respectively for those working inside and outside the capital.

Practitioner vs Population Growth: Accounting for population growth, practitioner numbers need to increase by 163% by 2020 in order to meet the target.

Refractionists: Current & Projected Workforce per Million Population

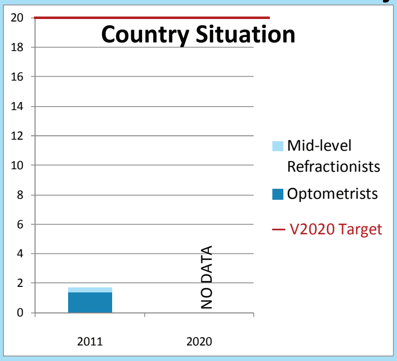

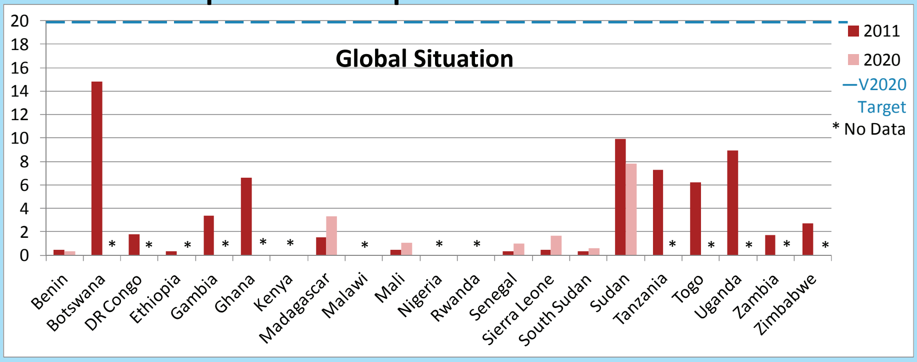

Practitioner Entry vs Exit: There is no information on entry and exit from the workforce for refractionists. All but 3 of the refractionists work in the public sector.

Practitioner Working Location: The overall refractionist to population ratio is 1.7. This ratio is 7.8 and 0.8 respectively for those working inside and outside the capital.

Practitioner vs Population Growth: Accounting for population growth, Zambia will need more than 15 times as many refractionists in the workforce by 2020 to meet this target.

Cataract Surgical Performance: Current & Projected Performance per Million Population

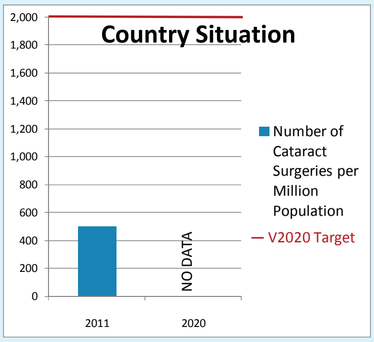

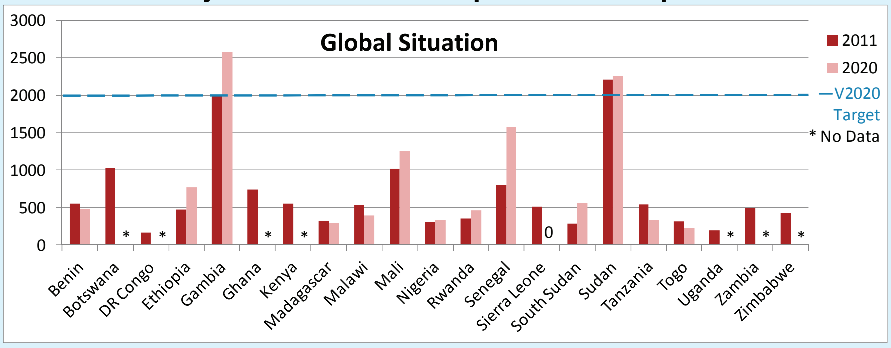

Zambia will need to perform more than 5 times as many cataract surgeries each year in order to meet this target by 2020, accounting for population growth.

Country Profile: Zambia

Current Situation: 2011				
Total Population	Population Living in Capital City	% Population Living in Capital	Population Over 50	% Population Over 50
13,506,196	1,802,000	13%	1,033,000	8%

VISION 2020 Targets	
Eye Care Cadre	# Practitioners per Million Population
Surgeons	4
OCOs/Nurses	10
Refractionists	20

Characteristics of the Active Eye Care Practitioner Workforce: 2011

Eye Care Cadre	Number of Practitioners in Active Workforce	Sector			Location		# Training Programmes available in Country
		Government	NGO/ Mission	Private for Profit	Capital	Outside Capital	
Ophthalmologists	18	12	3	3	6	12	1
Cataract Surgeons	8	4	1	3	4	4	0
**Surgeons**	**26**	**16**	**4**	**6**	**10**	**16**	**1**
Ophthalmic Clinical Officers	35	23	8	4	7	28	0
Ophthalmic Nurses	33	26	4	3	12	21	1
**OCOs/Nurses**	**68**	**49**	**12**	**7**	**19**	**49**	**1**
Optometrists	19	1	0	18	13	6	0
Mid-level Refractionists	4	2	0	2	1	3	1
**Refractionists**	**23**	**3**	**0**	**20**	**14**	**9**	**1**

Eye Care Practitioner Workforce Dynamics: 2011

Eye Care Cadre	Number of Practitioners in Active Workforce	Practitioners per Million Population			VISION 2020 Country Target # of Practitioners	Shortage in Practitioners to meet Target
		Countrywide	In Capital	Outside Capital		
Ophthalmologists	18	1.3	3.3	1.0		
Cataract Surgeons	8	0.6	2.2	0.3		
**Surgeons**	**26**	**1.9**	**5.5**	**1.4**	**54**	**28**
Ophthalmic Clinical Officers	35	2.6	3.9	2.4		
Ophthalmic Nurses	33	2.4	6.7	1.8		
**OCOs/Nurses**	**68**	**5.0**	**10.5**	**4.2**	**135**	**67**
Optometrists	19	1.4	7.2	0.5		
Mid-level Refractionists	4	0.3	0.6	0.3		
**Refractionists**	**23**	**1.7**	**7.8**	**0.8**	**270**	**247**

Annual Cataract Surgical Performance

Number of Cataract Surgeries Performed (data from 2010)*	6,734
Number of Cataract Surgeries per Surgeon (surgical performance ratio)	259
% Surgeries Performed by Ophthalmologists (estimate)	ND
Number of Cataract Surgeries per Million Population (CSR)	499
Target Number of Cataract Surgeries to meet VISION 2020 Target	27,012
Shortage in Cataract Surgeries to meet VISION 2020 Target	20,278

* Estimate provided by IAPB

ND: No Data

Projected Situation: 2020

Projected Total Population	Projected Population Living in Capital City	% Projected Population Living in Capital	Projected Population Over 50	% Projected Population Over 50	Expected 9-year Population Increase	Expected 9-year Over 50 Population Increase
17,918,464	2,623,337	15%	1,346,000	8%	33%	30%

Projected Eye Care Practitioner Workforce Dynamics: 2020

Eye Care Cadre	Number of Practitioners in Active Workforce (2011)	Over last 3 years			Projected Net Change over next 9 years	Projected Number of Practitioners in Active Workforce	Projected Practitioners per Million Population			VISION 2020 Country Target # of Practitioners	Projected Shortage in Practitioners to meet VISION 2020
		Number Entered Workforce	Number Exited Workforce	Net Change in Practitioner Numbers			Countrywide	In Capital	Outside Capital		
Ophthalmologists	18	2	ND	ND	ND	ND	ND	ND	ND		
Cataract Surgeons	8	0	ND	ND	ND	ND	ND	ND	ND		
**Surgeons**	**26**	**2**	**ND**	**ND**	**ND**	**ND**	**ND**	**ND**	**ND**	**72**	**ND**
Ophthalmic Clinical Officers	35	23	ND	ND	ND	ND	ND	ND	ND		
Ophthalmic Nurses	33	22	ND	ND	ND	ND	ND	ND	ND		
**OCOs/Nurses**	**68**	**45**	**ND**	**ND**	**ND**	**ND**	**ND**	**ND**	**ND**	**179**	**ND**
Optometrists	19	ND	ND	ND	ND	ND	ND	ND	ND		
Mid-level Refractionists	4	0	ND	ND	ND	ND	ND	ND	ND		
**Refractionists**	**23**	**ND**	**ND**	**ND**	**ND**	**ND**	**ND**	**ND**	**ND**	**358**	**ND**

Annual Projected Cataract Surgical Performance: 2020

Eye Care Cadre	% Surgeries Performed (estimate)	Surgical Performance Ratio per Cadre (2011)	Projected Number of Surgeons in Active Workforce	Projected Number of Cataract Surgeries Performed	Projected Number of Cataract Surgeries per Million Population (CSR)	Target Number of Cataract Surgeries to meet VISION 2020 Target	Projected Shortage in Cataract Surgeries to meet VISION 2020 Target
Ophthalmologists	ND	ND	ND	ND			
Cataract Surgeons	ND	ND	ND	ND			
**Surgeons**	**ND**	**259**	**ND**	**ND**	**ND**	**35,837**	**ND**

ND: No Data

Country Profile: Zimbabwe

Key Messages

- **Surgeons:** Zimbabwe is less than half-way towards meeting this target.
- **OCOs/Nurses:** Zimbabwe has no ophthalmic clinical officers. The projected growth rate of ophthalmic nurses means that Zimbabwe is set to meet this target by 2020.
- **Refractionists:** In order to meet this target by 2020, Zimbabwe will need to recruit nearly 300 refractionists. There are currently 35 working in the country.
- **Cataract Surgeries:** Zimbabwe will need to quadruple the number of cataract surgeries performed each year in order to meet this target.

VISION 2020 Targets

	Eye Care Practitioners per Million Population			Cataract Surgeries Performed per Million Population	Cataract Surgeries Performed per Surgeon
	Surgeons*	Ophthalmic Clinical Officers /Nurses	Refrac-tionists		
VISION2020 Target	4	10	20	2,000	500
2011 Situation	1.9	6.2	2.8	428	220
On Track					

* For the Africa region this includes Ophthalmologists and Cataract Surgeons

Eye Care Practitioners: % Working Inside/Outside Capital

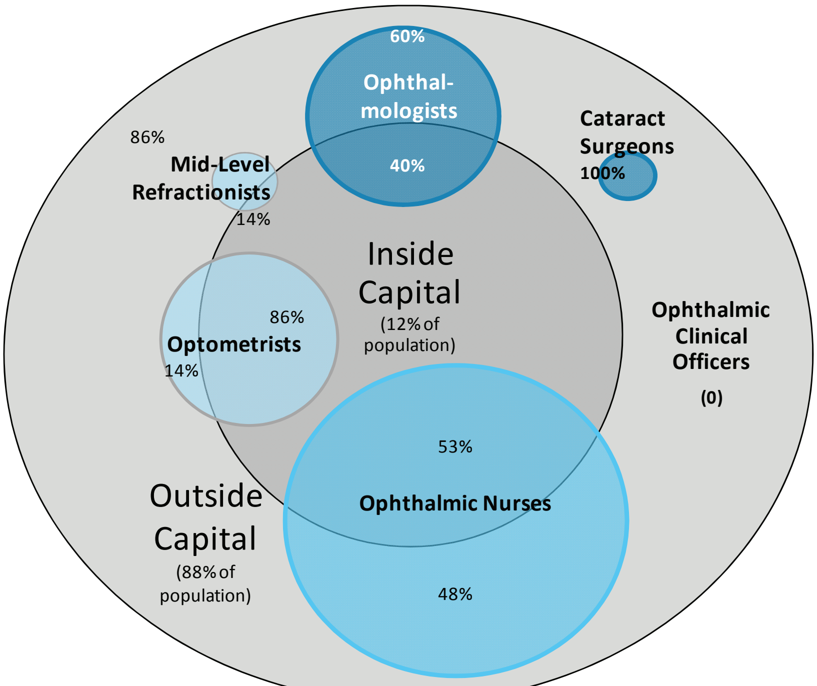

Note: Circles are proportional to numbers of eye care practitioners

In-Country Training Programmes

Surgeons — Total: 4

OCOs/Nurses — Total: 1

Refractionists — Total: 2

Distribution of Optometrists

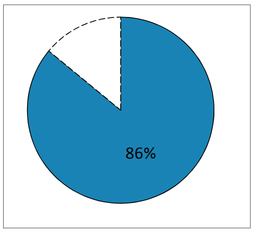

Optometrists based in the capital

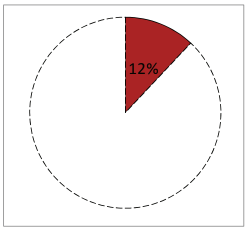

Population living in the capital

86% of optometrists treat 12% of the population

Eye Care Practitioners: Split between Sectors

Government | NGO/Mission | Private for Profit

Surgeons

Ophthalmologists

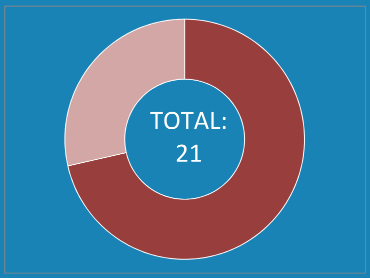

Cataract Surgeons

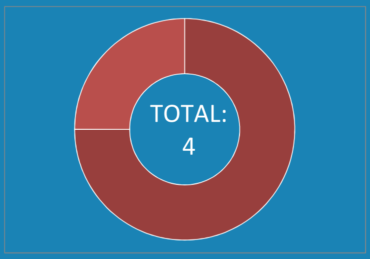

OCOs/Nurses

Ophthalmic Clinical Officers

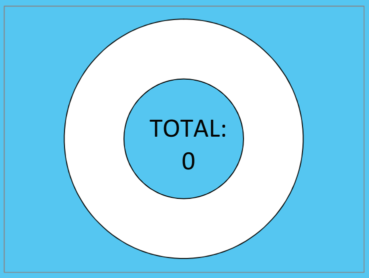

Ophthalmic Nurses

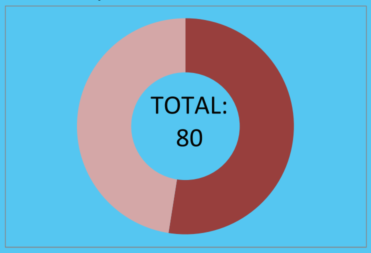

Refractionists

Optometrists

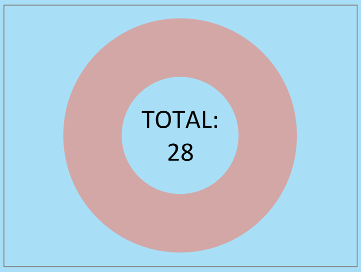

Mid-level Refractionists

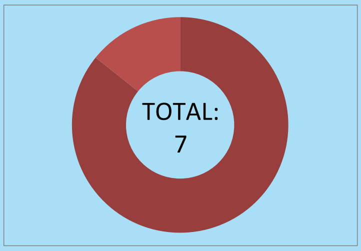

Surgeons: Current & Projected Workforce per Million Population

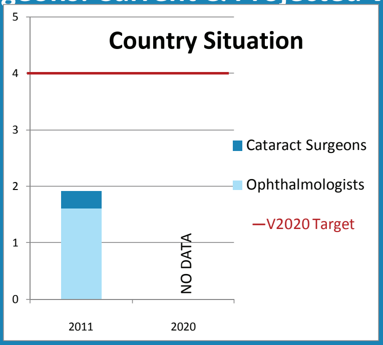

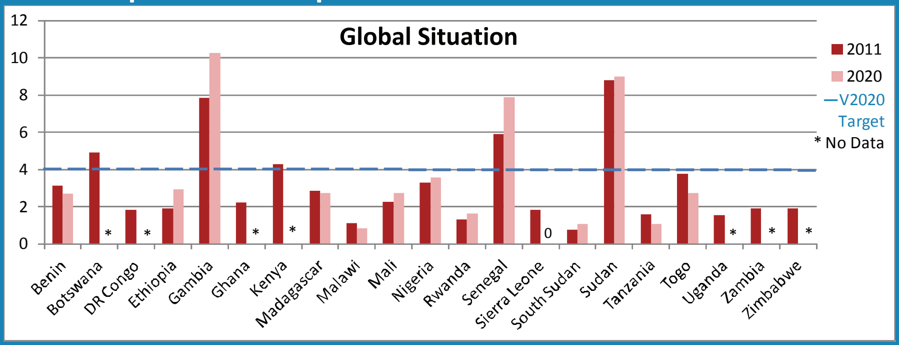

Practitioner Entry vs Exit: There is no information on how many surgeons have entered and exited the workforce over the past 3 years, so 2020 projections are not available.

Practitioner Working Location: The overall surgeon to population ratio is 1.9. This ratio is 7.8 and 1.1 respectively for those working inside and outside the capital.

Practitioner vs Population Growth: Accounting for the expected 21% population growth between 2011 and 2020, there will need to be a 150% increase in surgeon numbers in order to meet this target by 2020.

OCOs/Nurses: Current & Projected Workforce per Million Population

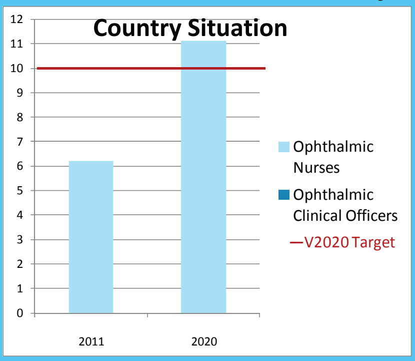

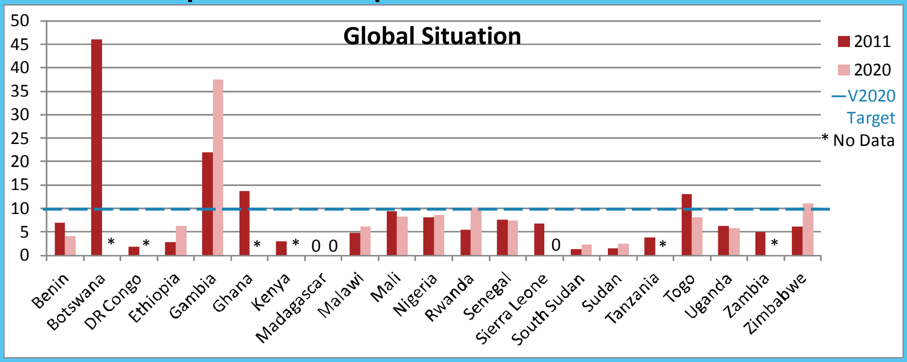

Practitioner Entry vs Exit: There are no ophthalmic clinical officers working in Zimbabwe. More ophthalmic nurses have entered the workforce than have exited over the past 3 years.

Practitioner Working Location: The overall practitioner to population ratio is 6.2. This ratio is 27.2 and 3.4 respectively for those working inside and outside the capital.

Practitioner vs Population Growth: The number of ophthalmic nurses is expected to grow faster than the general population. If current trends continue, Zimbabwe will meet this target by 2020.

Refractionists: Current & Projected Workforce per Million Population

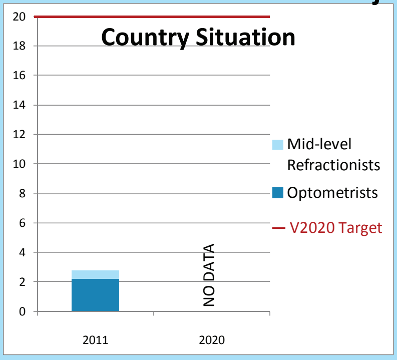

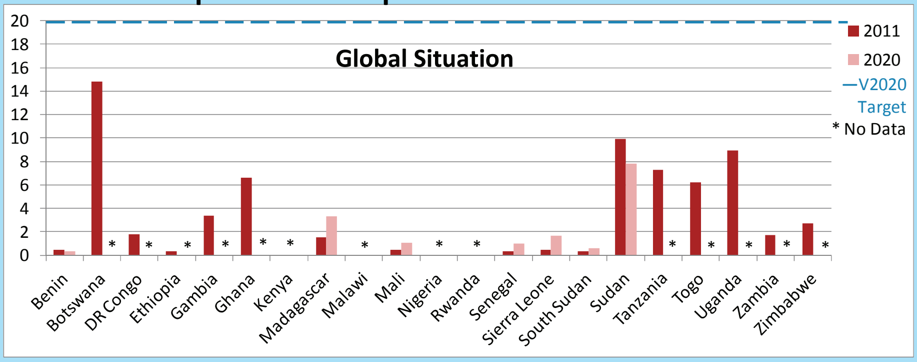

Practitioner Entry vs Exit: There is no information on how many refractionists have entered and exited the workforce over the past 3 years, so 2020 projections are not available.

Practitioner Working Location: The overall refractionist to population ratio is 2.3. This ratio is 16.2 and 0.9 respectively for those working inside and outside the capital.

Practitioner vs Population Growth: Accounting for population growth, Zimbabwe will need nearly 9 times as many refractionists in the workforce by 2020 to meet this target.

Cataract Surgical Performance: Current & Projected Performance per Million Population

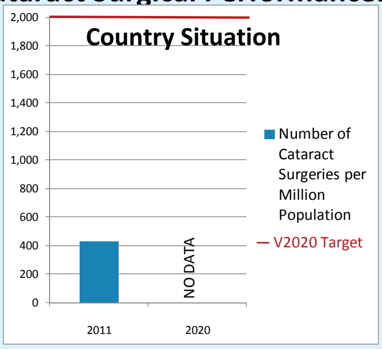

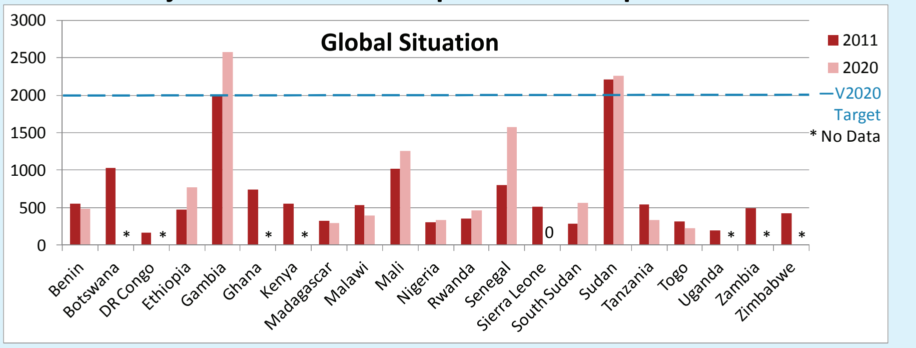

Accounting for population growth, Zimbabwe will need to perform more than 5 times as many cataract surgeries each year to meet this target by 2020.

Country Profile: Zimbabwe

Current Situation: 2011

Total Population	Population Living in Capital City	% Population Living in Capital	Population Over 50	% Population Over 50
12,841,284	1,542,000	12%	1,347,000	10%

VISION 2020 Targets

Eye Care Cadre	# Practitioners per Million Population
Surgeons	4
OCOs/Nurses	10
Refractionists	20

Characteristics of the Active Eye Care Practitioner Workforce: 2011

Eye Care Cadre	Number of Practitioners in Active Workforce	Sector			Location		# Training Programmes available in Country
		Government	NGO/ Mission	Private for Profit	Capital	Outside Capital	
Ophthalmologists *	21	15	0	6	12	8	2
Cataract Surgeons	4	3	1	0	0	4	2
**Surgeons**	**25**	**18**	**1**	**6**	**12**	**12**	**4**
Ophthalmic Clinical Officers	0	0	0	0	0	0	0
Ophthalmic Nurses	80	42	0	38	42	38	1
**OCOs/Nurses**	**80**	**42**	**0**	**38**	**42**	**38**	**1**
Optometrists	28	0	0	28	24	4	0
Mid-level Refractionists	7	6	1	0	1	6	2
**Refractionists**	**35**	**6**	**1**	**28**	**25**	**10**	**2**

Eye Care Practitioner Workforce Dynamics: 2011

Eye Care Cadre	Number of Practitioners in Active Workforce	Practitioners per Million Population			VISION 2020 Country Target # of Practitioners	Shortage in Practitioners to meet Target
		Countrywide	In Capital	Outside Capital		
Ophthalmologists	21	1.6	7.8	0.7		
Cataract Surgeons	4	0.3	0.0	0.4		
**Surgeons**	**25**	**1.9**	**7.8**	**1.1**	**51**	**26**
Ophthalmic Clinical Officers	0	0	0.0	0.0		
Ophthalmic Nurses	80	6.2	27.2	3.4		
**OCOs/Nurses**	**80**	**6.2**	**27.2**	**3.4**	**128**	**48**
Optometrists	28	2.2	15.6	0.4		
Mid-level Refractionists	7	0.56	0.6	0.5		
**Refractionists**	**35**	**2.8**	**16.2**	**0.9**	**257**	**222**

Annual Cataract Surgical Performance

Number of Cataract Surgeries Performed (data from 2010)	5,500
Number of Cataract Surgeries per Surgeon (surgical performance ratio)	220
% Surgeries Performed by Ophthalmologists (estimate)	100%
Number of Cataract Surgeries per Million Population (CSR)	428
Target Number of Cataract Surgeries to meet VISION 2020 Target	25,683
Shortage in Cataract Surgeries to meet VISION 2020 Target	20,183

* Some location data is missing, but as this represents less than 5% of active workforce it has been used in the analysis

Projected Situation: 2020

Projected Total Population	Projected Population Living in Capital City	% Projected Population Living in Capital	Projected Population Over 50	% Projected Population Over 50	Expected 9-year Population Increase	Expected 9-year Over 50 Population Increase
15,545,865	2,078,351	13%	1,539,000	10%	21%	14%

Projected Eye Care Practitioner Workforce Dynamics: 2020

Eye Care Cadre	Number of Practitioners in Active Workforce (2011)	Over last 3 years			Projected Net Change over next 9 years	Projected Number of Practitioners in Active Workforce	Projected Practitioners per Million Population			VISION 2020 Country Target # of Practitioners	Projected Shortage in Practitioners to meet VISION 2020
		Number Entered Workforce	Number Exited Workforce	Net Change in Practitioner Numbers			Countrywide	In Capital	Outside Capital		
Ophthalmologists	21	ND	7	ND	ND	ND	ND	ND	ND		
Cataract Surgeons	4	ND	2	ND	ND	ND	ND	ND	ND		
**Surgeons**	**25**	**ND**	**9**	**ND**	**ND**	**ND**	**ND**	**ND**	**ND**	**62**	**ND**
Ophthalmic Clinical Officers	0	0	0	0	0	0	0.0	0.0	0.0		
Ophthalmic Nurses	80	42	11	31	93	173	11.1	43.7	6.1		
**OCOs/Nurses**	**80**	**42**	**11**	**31**	**93**	**173**	**11.1**	**43.7**	**6.1**	**155**	**Target Met**
Optometrists	28	4	ND	ND	ND	ND	ND	ND	ND		
Mid-level Refractionists	7	ND	28	ND	ND	ND	ND	ND	ND		
**Refractionists**	**35**	**ND**	**ND**	**ND**	**ND**	**ND**	**ND**	**ND**	**ND**	**311**	**ND**

Annual Projected Cataract Surgical Performance: 2020

Eye Care Cadre	% Surgeries Performed (estimate)	Surgical Performance Ratio per Cadre (2011)	Projected Number of Surgeons in Active Workforce	Projected Number of Cataract Surgeries Performed	Projected Number of Cataract Surgeries per Million Population (CSR)	Target Number of Cataract Surgeries to meet VISION 2020 Target	Projected Shortage in Cataract Surgeries to meet VISION 2020 Target
Ophthalmologists	100%	262	ND	ND			
Cataract Surgeons	0	0	ND	ND			
**Surgeons**	**100%**	**220**	**ND**	**ND**	**ND**	**31,092**	**ND**

ND: No Data
